# CD163^+^ macrophages restrain vascular calcification, promoting the development of high-risk plaque

**DOI:** 10.1172/jci.insight.154922

**Published:** 2023-03-08

**Authors:** Atsushi Sakamoto, Rika Kawakami, Masayuki Mori, Liang Guo, Ka Hyun Paek, Jose Verdezoto Mosquera, Anne Cornelissen, Saikat Kumar B. Ghosh, Kenji Kawai, Takao Konishi, Raquel Fernandez, Daniela T. Fuller, Weili Xu, Aimee E. Vozenilek, Yu Sato, Hiroyuki Jinnouchi, Sho Torii, Adam W. Turner, Hirokuni Akahori, Salome Kuntz, Craig C. Weinkauf, Parker J. Lee, Robert Kutys, Kathryn Harris, Alfred Lawrence Killey, Christina M. Mayhew, Matthew Ellis, Leah M. Weinstein, Neel V. Gadhoke, Roma Dhingra, Jeremy Ullman, Armella Dikongue, Maria E. Romero, Frank D. Kolodgie, Clint L. Miller, Renu Virmani, Aloke V. Finn

**Affiliations:** 1 CVPath Institute, Inc., Gaithersburg, Maryland, USA.; 2 Department of Public Health Sciences, Center for Public Health Genomics, University of Virginia School of Medicine, Charlottesville, Virginia, USA.; 3 Department of Biochemistry and Molecular Genetics, University of Virginia, Charlottesville, Virginia, USA.; 4 Department of Cardiovascular and Renal Medicine, Hyogo Medical University, Nishinomiya, Hyogo, Japan.; 5 Division of Vascular and Endovascular Surgery, University of Arizona, Tucson, Arizona, USA.; 6 University of Maryland School of Medicine, Baltimore, Maryland, USA.

**Keywords:** Cell Biology, Vascular Biology, Atherosclerosis, Macrophages, Plaque formation

## Abstract

Vascular calcification (VC) is concomitant with atherosclerosis, yet it remains uncertain why rupture-prone high-risk plaques do not typically show extensive calcification. Intraplaque hemorrhage (IPH) deposits erythrocyte-derived cholesterol, enlarging the necrotic core and promoting high-risk plaque development. Pro-atherogenic CD163^+^ alternative macrophages engulf hemoglobin:haptoglobin (HH) complexes at IPH sites. However, their role in VC has never been examined to our knowledge. Here we show, in human arteries, the distribution of CD163^+^ macrophages correlated inversely with VC. In vitro experiments using vascular smooth muscle cells (VSMCs) cultured with HH-exposed human macrophage — M(Hb) — supernatant reduced calcification, while arteries from *ApoE^–/–^* *CD163^–/–^* mice showed greater VC. M(Hb) supernatant–exposed VSMCs showed activated NF-κB, while blocking NF-κB attenuated the anticalcific effect of M(Hb) on VSMCs. CD163^+^ macrophages altered VC through NF-κB–induced transcription of hyaluronan synthase (HAS), an enzyme that catalyzes the formation of the extracellular matrix glycosaminoglycan, hyaluronan, within VSMCs. M(Hb) supernatants enhanced HAS production in VSMCs, while knocking down HAS attenuated its anticalcific effect. NF-κB blockade in *ApoE^–/–^* mice reduced hyaluronan and increased VC. In human arteries, hyaluronan and HAS were increased in areas of CD163^+^ macrophage presence. Our findings highlight an important mechanism by which CD163^+^ macrophages inhibit VC through NF-κB–induced HAS augmentation and thus promote the high-risk plaque development.

## Graphical abstract

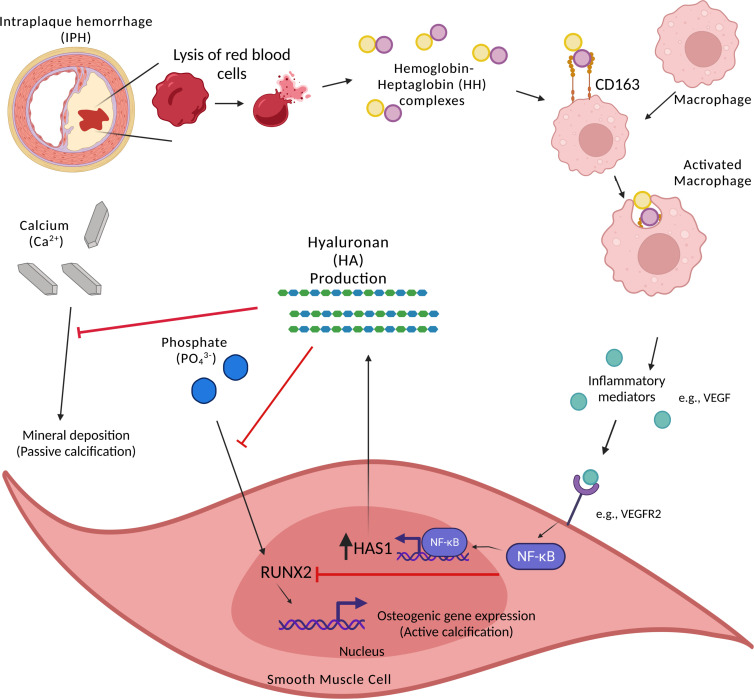

## Introduction

The presence of vascular calcification (VC) has been recognized in atherosclerotic coronary arteries for more than 100 years. Calcification suggests the presence of atherosclerotic disease and correlates with disease burden (1, 2). VC involves the deposition and crystallization of calcium/phosphate in the form of hydroxyapatite within the arterial wall. This is a highly regulated process with cells of the vascular wall assuming an osteoblast-like phenotype, which upregulates specific transcription factors, resulting in expression of extracellular matrix (ECM) and incorporation of hydroxyapatite crystals (3, 4). Yet there are still important aspects of VC that are not completely understood. One of these is the complex relationship of VC with plaque instability. Coronary calcium is greater in stable than unstable plaques, and there is a negative correlation between necrotic core (NC) and calcification areas (5, 6). How and why this divergence in the process of calcification occurs remain poorly understood.

Macrophages are involved in various stages of atherosclerosis and are thought to play important roles in the process of VC, either directly or indirectly, through their influence on other cell types. The increased recognition of the macrophage phenotypic heterogeneity within the process of atherosclerosis has made understanding their roles in atherosclerosis and VC increasingly complex (7, 8). As yet, little is understood about the role of macrophage polarization in human atherosclerotic VC.

One major cause of coronary plaque progression into high-risk unstable plaques, and macrophage polarization, is intraplaque hemorrhage (IPH), which is thought to result from plaque neovascularization and microvascular permeability (9, 10). IPH is considered a critical event in the advancement of atherosclerotic plaques by causing the accumulation of erythrocyte membranes, which are rich in free cholesterol and promote NC enlargement (11). Within areas of IPH, free hemoglobin released from lysed erythrocyte is bound by the plasma haptoglobin and hemoglobin:haptoglobin (HH) complexes are formed. HH is engulfed by macrophages via the CD163 scavenger receptor exclusively expressed on cells of this lineage (12). We recently reported CD163^+^ alternative macrophage response to IPH provokes angiogenesis and microvascular permeability by releasing vascular endothelial growth factor (VEGF), which promotes plaque destabilization by initiating a vicious cycle that likely leads to further IPH, inflammation, and NC expansion (10). However, the role of CD163^+^ macrophages in VC has never been explored to our knowledge.

Recently, it was suggested that IPH promotes VC through lysis of erythrocytes (13, 14). Erythrocyte membranes dose-dependently enhance calcification in murine aortic rings through a mechanism that involved nitric oxide (13). However, these experiments did not take into account the macrophage inflammatory response to IPH, which might itself alter this response. In addition, these findings seem to be inconsistent with the known role of IPH in promoting high-risk plaque phenotypes, which usually contain less calcium (15).

Here, we revealed an important role for CD163^+^ macrophages in preventing VC. We demonstrated a strong inverse correlation between VC and CD163^+^ macrophage presence in atherosclerotic plaques and that genetic deletion of CD163 in a mouse model enhanced plaque calcification. CD163^+^ macrophages reduced plaque calcification through a mechanism involving stimulation of NF-κB signaling within vascular smooth muscle cells (VSMCs), which enhanced production of the anticalcific ECM glycosaminoglycan, hyaluronan (HA). These data suggest the formation of unstable plaques by IPH occurs concomitantly with protection against VC by CD163^+^ macrophage-dependent effects on VSMCs and help explain the relationship between plaque instability and the development of VC.

## Results

### An inverse correlation between VC and CD163^+^ macrophages in human atherosclerotic plaques.

A total of 60 atherosclerotic carotid plaque samples removed during surgical carotid endarterectomy (CEA) were available from the CVPath Registry (16). After excluding 28 samples, which were missing detailed patient history, we included 32 patient samples in the analysis. Pathologic characteristics were assessed based on adjacent sections of hematoxylin & eosin (H&E) staining and immunohistochemical staining against CD68 (a general macrophage marker) and CD163. Patient clinical information and histomorphometric findings are shown in Table 1. Each patient’s clinical background and pathologic plaque characteristics, obtained from the average of the 3 most diseased sections in each case, were compared between symptomatic (Sym) and asymptomatic (Asy) cases as reported in clinical record (details are provided in Supplemental Methods; supplemental material available online with this article; https://doi.org/10.1172/jci.insight.154922DS1). Overall, age, sex, and underlying comorbidities including hypertension, diabetes mellitus, current smoking, obesity, and prior history of CAD were not different between Sym and Asy groups. IEL area, lumen area, plaque area, and percentage area stenosis were similar in Sym versus Asy cases. Calcification area and percentage calcification in the plaque were significantly smaller in Sym versus Asy cases (median 3.01, IQR [1–5.78] mm^2^ vs. 6.61 [5–11.79] mm^2^, *P* < 0.01; and 8.29 [2.17–12.79]% vs. 15.64 [7.97–27.41]%, *P* = 0.04, respectively). In contrast, CD163-positive area was relatively larger and percentage CD163-positive area in the plaque was significantly greater in Sym versus Asy cases (0.59 [0.19–1.99] mm^2^ vs. 0.23 [0.05–0.43] mm^2^, *P* = 0.06; and 1.59 [0.51–4.80]% vs. 0.47 [0.15–1.48]%, *P* = 0.03, respectively). CD68-positive area showed a similar tendency. These results indicate that Sym plaques have less calcium but greater CD163^+^ macrophages, suggesting a role for CD163 in plaque calcification.

**Figure 1:**
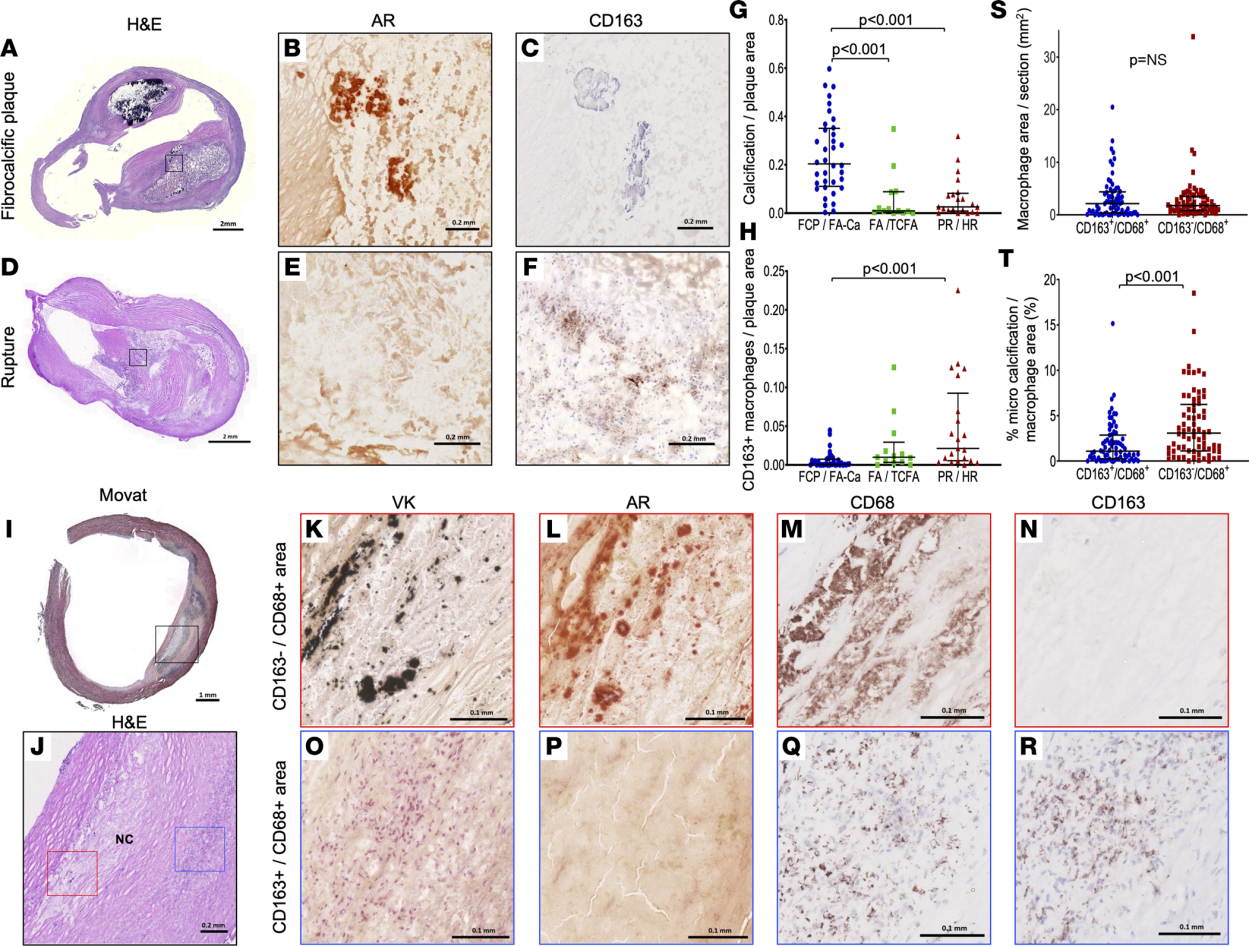
CD163^+^ macrophages are associated with reduced vascular calcification in human carotid artery advanced atheromas. (**A**–**C**) FCP sections from 82-year-old male. Low-power H&E image (**A**). High-power AR (**B**) and CD163 immunostaining (**C**) from the corresponding black rectangle in **A**. No CD163^+^ cells were observed around calcification stained as red in AR. (**D**–**F**) PR sections from 46-year-old male. Low-power H&E image (**D**). High-power images of AR (**E**) and CD163 immunostaining (**F**) from the corresponding black rectangle in **D**. Multiple CD163^+^ cells were observed around hemorrhagic area without any AR-positive lesions. (**G** and **H**) Seventy atheroma sections from 32 patients were classified into FCP or FA-Ca (*n* = 36, blue), FA or TCFA (*n* = 13, green), and PR or HR (*n* = 21, red), with the corresponding percentage of calcification (**G**) and CD163^+^ macrophages (**H**) per plaque area. (**I**–**R**) FA sections from 62-year-old male. Low-power Movat image (**I**). Mid-power image of H&E (**J**) of the corresponding black rectangle in **I**. High-power images of VK (**K** and **O**), AR (**L** and **P**), as well as immunostaining for CD68 (a general macrophage marker) (**M** and **Q**) and CD163 (**N** and **R**) of the corresponding red (**K**–**N**) and blue rectangles (**O**–**R**) in **J**. Note the absence of calcification in areas of CD163^+^ macrophages but its presence in CD163^–^ areas. (**S** and **T**) CD163^+^CD68^+^ and CD163^–^CD68^+^ areas were determined in 70 sections (the method of outlining was in Supplemental Figure 1). Each CD163^+^CD68^+^ and CD163^–^CD68^+^ area was put in adjacent AR sections. Then AR-positive microcalcification areas in each field were digitally analyzed. The total area of CD163^+^CD68^+^ and CD163^–^CD68^+^ was similar (**S**); however, % microcalcification in CD163^–^CD68^+^ was greater than in CD163^+^CD68^+^ (**T**). Results are presented as median and interquartile range (**G**, **H**, **S**, and **T**). Kruskal-Wallis test followed by post hoc Dunn’s test (**G** and **H**) or Wilcoxon’s matched-pair signed-rank test (**S** and **T**) was applied. Data normality was tested by Shapiro-Wilk test. Scale bars: 2 mm (**A** and **D**), 0.2 mm (**B**, **C**, **E**, and **F**) 1 mm (**I**), 0.2 mm (**J**), 0.1 mm (**K**–**R**). AR, Alizarin Red; Ca, calcification; FA, fibroatheroma; FA-Ca, fibroatheroma with calcification; FCP, fibrocalcific plaque; HR, healed plaque rupture; PR, plaque rupture; TCFA, thin-cap fibroatheroma; VK, von Kossa.

To elucidate the spatial correlation between calcification and macrophage subtype, a total of 70 sections (32 patients) from the advanced plaque phenotypes described above were selected and classified into 3 groups; fibrocalcific or fibroatheroma with calcification (FCP/FA-Ca) (*n* = 36), fibroatheroma or thin-cap fibroatheroma (FA/TCFA) (*n* = 13), and plaque rupture or healed rupture (PR/HR) (*n* = 21). The relationship between calcification, as assessed by Alizarin Red (AR) staining, and CD163-positive area was examined by the histology of adjacent sections (Figure 1, A–H). AR-positive area was greater in FCP/FA-Ca versus FA/TCFA (*P* < 0.001) and PR/HR (*P* < 0.001) (Figure 1G), while the CD163-positive area was greater in PR/HR versus FCP/FA-Ca (*P* < 0.001) (Figure 1H). Since plaque macrophages have been reported as an important source of calcification, a detailed spatial distribution of microcalcifications and CD163^+^CD68^+^ and CD163^–^CD68^+^ macrophages was conducted (Figure 1, I–T). CD163^+^CD68^+^ and CD163^–^CD68^+^ areas were digitally outlined, and percentage AR-positive area in the 2 different macrophage areas was compared as shown in Supplemental Figure 1. CD163^–^CD68^+^ areas were mainly found in the plaque NC as previously reported (10). Although the total area of CD163^+^CD68^+^ and CD163^–^CD68^+^ areas was equivalent (Figure 1S), microcalcifications assessed by AR were significantly greater in CD163^–^CD68^+^ areas (Figure 1T). These findings suggest an inverse correlation between CD163^+^ macrophage presence and plaque calcification.

### Distribution patterns of calcification and CD163^+^ macrophages in chronic total occlusions within human coronary arteries.

**Figure 2:**
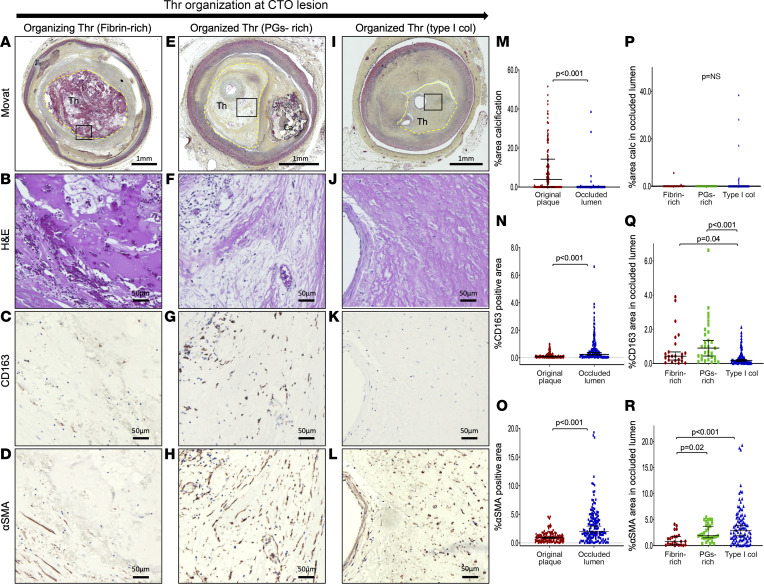
The distribution pattern of calcification and CD163^+^ macrophages in the underlying plaque and thrombus occluded lumen of human coronary CTOs. (**A**–**D**) Section with thrombotic total occlusion of RCA from 39-year-old man who died of recent inferior myocardial infarction. Low-power Movat image showing fibrin-rich organizing thrombus (**A**). High-power images of H&E (**B**) and immunostaining for CD163 (**C**) and α-SMA (**D**) (corresponding black rectangle in **A**). Occluded lumen with fibrin-rich thrombus (yellow-dot outline in **A**) was observed. CD163^+^ cells and small amount of α-SMA^+^ cells were found without visible calcification at occluded lumen. (**E**–**H**) CTO section from 92-year-old man who died of a subdural hemorrhage. Low-power Movat image showing PG-rich organized thrombus (**E**). High-power images of H&E (**F**) and immunostaining for CD163 (**G**) and α-SMA (**H**) (corresponding black rectangle in **E**). Sheet Ca was observed in the original plaque. Multiple CD163^+^ cells and α-SMA^+^ cells were found in PG-rich thrombus at occluded lumen (yellow-dot outline in **E**) without visible calcification. (**I**–**L**) CTO section from 56-year-old man with triple-vessel disease. Low-power Movat image with type I collagen–rich organized thrombus (**I**). High-power images of H&E (**J**) and immunostaining for CD163 (**K**) and α-SMA (**L**) (corresponding black rectangle in **I**). Occluded thrombus was replaced by type I collagen over time. No CD163^+^ cells or calcification was observed at occluded lumen (yellow-dot outline in **I**). (**M**–**R**) We obtained 145 CTO sections from 21 vessels (19 patients). Percentage area calcification (**M**), % CD163^+^ area (**N**), and % α-SMA^+^ area (**O**) were compared between original plaque and occluded lumen. Sections were classified into fibrin-rich organizing thrombus (*n* = 23), PG-rich organized thrombus (*n* = 31), and type I collagen–rich organized thrombus (*n* = 91). Percentage area calcification (**P**), % CD163^+^ area (**Q**), and % α-SMA^+^ area (**R**) in occluded lumen were compared. Results are presented as the median and interquartile range (**M**–**R**). Wilcoxon’s matched-pairs signed-rank test (**M**–**O**) or Kruskal-Wallis test followed by post hoc Dunn’s test (**P**–**R**) was applied. Data normality was tested by Shapiro-Wilk test. Scale bars: 1 mm (**A**, **E**, and **I**), 50 μm (**B**–**D**, **F**–**H**, and **J**–**L**). RCA, right coronary artery; α-SMA, α–smooth muscle actin; col, collagen; PG, proteoglycan; Th, thrombus.

The etiology of chronic total occlusion (CTO) is primarily due to thrombotic occlusion. These areas are rich in erythrocytes, which are entrapped in the clot. Subsequent erythrocyte lysis attracts CD163^+^ macrophages during thrombus organization. We used this phenomenon to understand whether erythrocytes in the presence of CD163^+^ macrophages promote or inhibit vascular calcification within the CTO itself. A total of 149 CTO sections from 21 vessels (19 patients) were obtained from CVPath Registry, and section-based analysis was done to examine the distribution pattern of CD163^+^ macrophages, VSMCs, and calcification (Figure 2, A–R). Patient clinical information is shown in Supplemental Table 1. The area of the original plaque and the occluded lumen were determined. Greater percentage area calcification and smaller percentage area CD163^+^ macrophages were observed in the underlying plaque versus the occluded lumen (both *P* < 0.001) (Figure 2, M and N). CTO sections were divided into 3 phases of luminal thrombus organization, 1) fibrin-rich organizing thrombus, 2) PG-rich organized thrombus, and 3) type I collagen–rich organized thrombus as previously described (17). Percentage CD163^+^ area in the occluded lumen was greater in fibrin-rich and PG-rich occluded lumen than type I collagen–rich occlusion (Figure 2Q) accompanied by the presence of α-SMA–positive VSMCs in the lumen (Figure 2, O and R), suggesting the presence of CD163^+^ macrophages in the initial but not the later phases of thrombus organization and that possibly the interaction between CD163^+^ macrophages and VSMCs exists at the site of thrombus organization. However, almost no macrocalcifications were observed in the occluded lumen, with the exception of 3 sections in which it was difficult to distinguish the occluded lumen from the original plaque (Figure 2P). These findings also suggest an inverse relationship between CD163^+^ macrophages and VC.

### Genetic deletion of CD163 in mice promotes VC.

**Figure 3:**
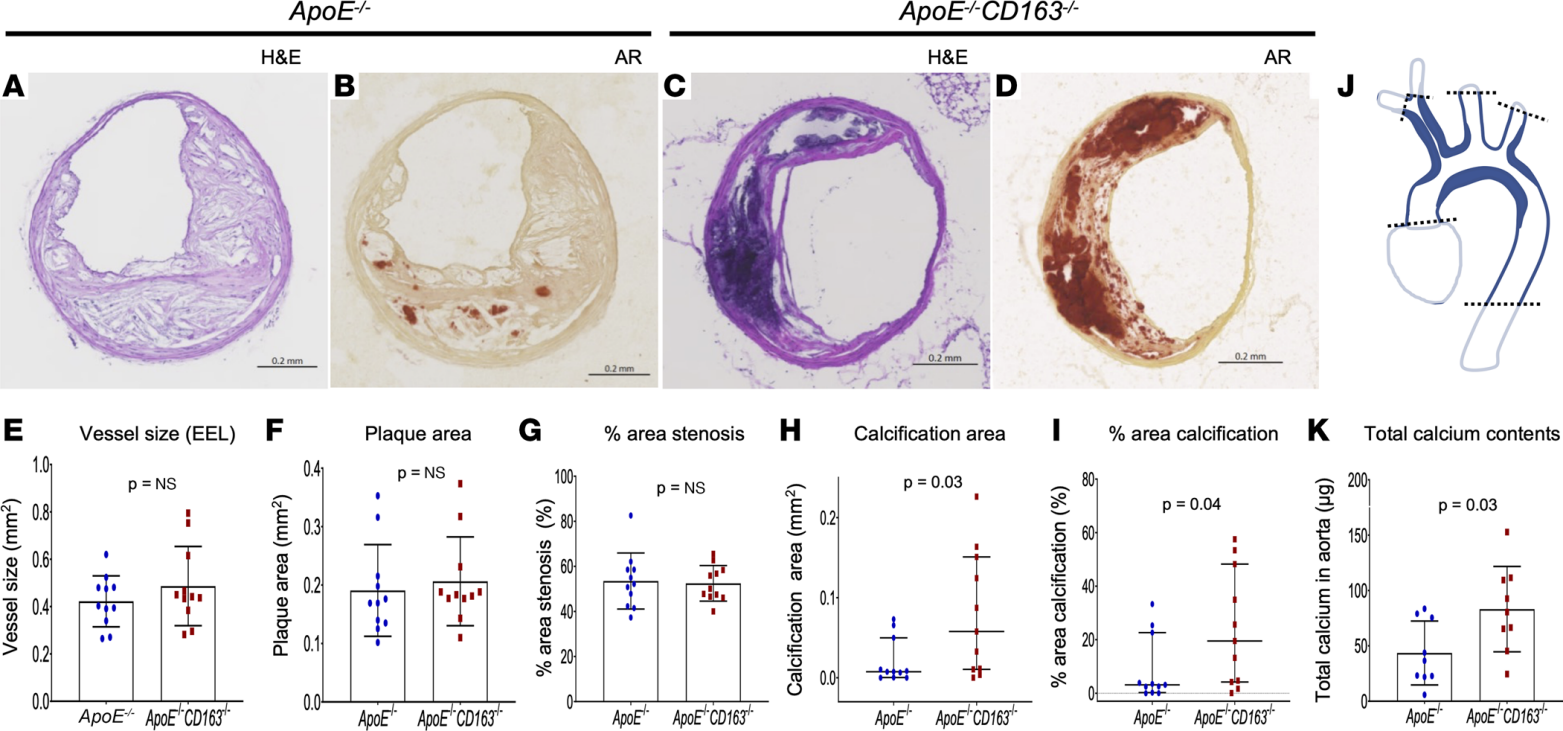
BCA and aortic calcification in aged *ApoE^–/–^* versus *ApoE^–/–^* *CD163^–/–^* mice. (**A**–**D**) Representative BCA sections with H&E and AR staining obtained from *ApoE^–/–^* (**A** and **B**) and *ApoE^–/–^* *CD163^–/–^* (**C** and **D**) mice (1.5 years old). (**E**–**I**) Vessel size (EEL) (**E**), plaque area (**F**), % area stenosis (**G**), calcification area assessed by AR (**H**), and % calcification area (**I**) at the most stenotic BCA lesion were compared between *ApoE^–/–^* and *ApoE^–/–^* *CD163^–/–^* mice (*n* = 11 per group). (**J**) Schematic diagram of a mouse aorta from which total calcium contents were extracted for colorimetric analysis. Aortic tissues from ascending (cut just above aortic valve) to descending (cut at the level of diaphragm) were obtained for further calcium extraction process. BCA (main branch only), left common carotid, and subclavian arteries with the equivalent length with BCA were also included in the specimens. (**K**) Total aortic calcium contents assessed by colorimetric assay with the comparison of *ApoE^–/–^* and *ApoE^–/–^* *CD163^–/–^* mice (*n* = 9 per group). Results are presented as the mean ± standard deviation (**E**–**G** and **K**) or median and interquartile range (**H** and **I**). *T* test (**E**–**G** and **K**) or Mann-Whitney test (**H** and **I**) was applied. Data normality was tested by Shapiro-Wilk test. EEL, external elastic lamina.

To explore the role of CD163 in the process of VC, we crossed atherosclerosis-prone apolipoprotein E–deficient (*ApoE^–/–^*) mice with *CD163^–/–^* mice (*ApoE^–/–^* *CD163^–/–^*) (both on a C57BL/6J background) (10) and compared the features of atherosclerosis with *ApoE^–/–^* mice. To obtain more calcified plaque development, we examined the brachiocephalic artery (BCA) from aged mice (i.e., 1.5 years old with normal chow feeding). BCAs were chosen because they frequently develop hemorrhage in mice of this age (18). Representative BCA sections from *ApoE^–/–^* and *ApoE^–/–^* *CD163^–/–^* mice are shown in Figure 3, A–D. At the most stenotic sections, equivalent vessel size, plaque area, and percentage area stenosis were observed between *ApoE^–/–^* and *ApoE^–/–^* *CD163^–/–^* mice (Figure 3, E–G), while calcification area and percentage area calcification were significantly greater in *ApoE^–/–^* *CD163^–/–^* versus *ApoE^–/–^* mice (Figure 3, H and I). Moreover, total calcium content extracted from the thoracic aorta (Figure 3J) was significantly greater in *ApoE^–/–^* *CD163^–/–^* versus *ApoE^–/–^* mice (Figure 3K), suggesting a role for CD163 in preventing VC.

### Supernatant from CD163^+^ macrophages attenuates VSMC calcification in vitro.

**Figure 4:**
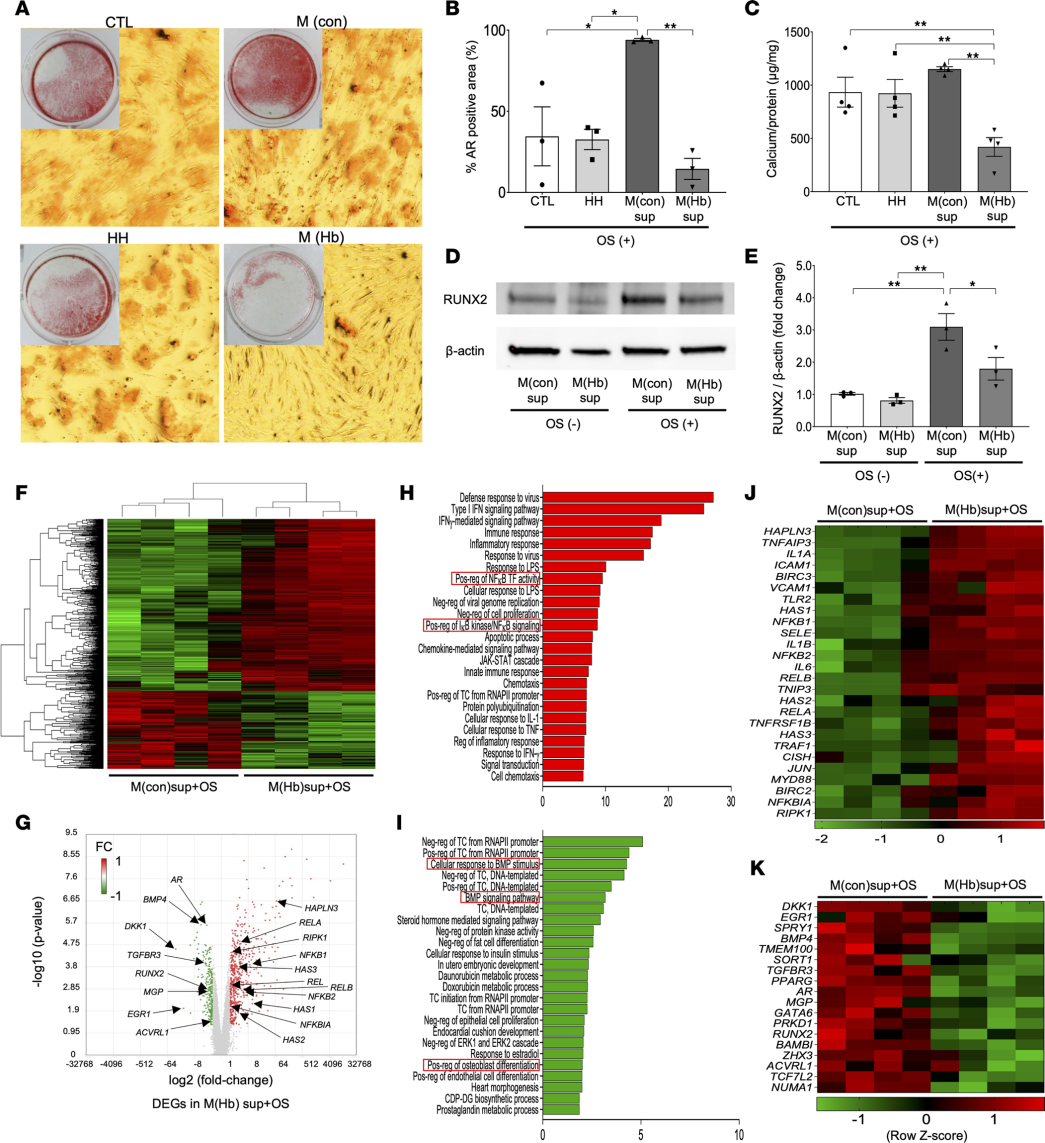
Effect of M(Hb) supernatant on HASMC calcification in vitro. (**A**–**C**) HASMCs were exposed for 2 days to control basic culture media with OS (containing CaCl_2_, β-glycerophosphate, l-ascorbic acid, insulin, and dexamethasone) with or without HH, or with M(con)sup or M(Hb)sup. Representative macro- (inset) and microscopic AR findings (4× original magnification) in each condition (**A**). Summary of % AR-positive area in each condition (**B**) (*n* = 3 per group). Amount of calcium examined by colorimetric assay adjusted by protein amount (**C**) (*n* = 4 per group). (**D** and **E**) HASMCs were cultured for 24 hours in M(con) or M(Hb) with or without OS. Representative immunoblotting image of RUNX2 and β-actin (**D**). Summary of densitometry analysis (**E**) (*n* = 3 per group). (**F**–**K**) HASMCs were cultured for 6 hours in M(con) or M(Hb) with OS. Extracted RNA samples were analyzed by Affymetrix Clariom S microarray (*n* = 4 per group). We demonstrated 548 upregulated and 249 downregulated DEGs in M(Hb)+OS versus M(con)+OS condition (threshold: FC ≥ 2.0 or ≤ –2.0 with *P* ≤ 0.05). Heatmap of 797 DEGs visualized by row *z* score scaling (**F**). Volcano plots detailing the magnitude of expression difference (**G**). GO enrichment analysis of unbiased DEGs was conducted by DAVID v6.8 bioinformatics resources. Top 25 GO BP terms with highest statistical significance in up- and downregulated DEGs (**H** and **I**). Selected genes related to inflammation and NF-κB signaling from upregulated genes (**J**) and these related to calcification/osteogenic differentiation-related genes from downregulated DEGs (**K**) were visualized by heatmap with row *z* score scaling of log_2_ FC. *P < 0.05, **P < 0.01. Results are presented as the mean ± standard error and ANOVA followed by post hoc Tukey’s test was applied (**B**, **C**, and **E**). Data normality was tested by Shapiro-Wilk test. The experiments of **A**–**E** were performed at least 3 times to confirm their reproducibility. AR, Alizarin Red; BP, biological process; CTL, control; DEGs, differentially expressed genes; FC, fold change; GO, gene ontology; HH, hemoglobin-haptoglobin complex; M(con)sup, control macrophage supernatant; M(Hb)sup, HH-differentiated macrophage supernatant; Neg-reg, negative regulation; OS, osteogenic components supplementation; Pos-reg, positive regulation; TC, transcription; TF, transcription factor.

To verify the effect of CD163^+^ macrophages on VC, we conducted an in vitro calcification assay. Human aortic smooth muscle cells (HASMCs) were exposed to basic culture media with osteogenic component supplementation (OS; containing CaCl_2_, β-glycerophosphate, l-ascorbic acid, insulin, and dexamethasone) for 6, 24, and 48 hours. The results of AR-positive staining area, calcium deposition as assessed by colorimetric assay, and Western blot analysis for runt-related transcription factor 2 (RUNX2), a well-known transcriptional factor for osteogenic differentiation of HASMCs, were all upregulated in a time-dependent manner (Supplemental Figure 2). CD163^+^ cells, termed HH-exposed human macrophages —M(Hb) — can be reproduced in vitro by differentiation of human monocytes over 5 to 7 days in HH-enriched media (9). Control macrophages — M(con) — are differentiated in a similar manner but without HH. The supernatant of M(con) and M(Hb) was used to understand the effect of these cells on HASMCs. HASMCs were cultured with M(con) or M(Hb) supernatants, control media, or HH-supplemented media, in the presence or absence of OS for 48 hours. HASMCs exposed to control media, HH-supplemented media, and M(con) supernatant with OS showed areas of large calcium deposition represented by AR staining. AR staining area was significantly suppressed by M(Hb) supernatant exposure (*P* < 0.01) (Figure 4, A and B). Calcium was also assessed by colorimetric analysis, which revealed less calcium deposition in M(Hb)-exposed cells (*P* < 0.01) (Figure 4C). Further, RUNX2 protein expression level in HASMCs was equivalent after M(con) and M(Hb) exposure for 24 hours under non-OS condition. Under OS condition, RUNX2 was elevated in M(con), but it was significantly suppressed by M(Hb) supernatant (Figure 4, D and E). These results demonstrate the inhibitory effect of CD163^+^ macrophages on mineral deposition within HASMCs.

To further explore the molecular mechanisms underlying these results, we performed microarray analysis of RNA samples extracted from HASMCs exposed to M(con) or M(Hb) with OS (6 hours). Differentially expressed gene (DEG) analysis showed 548 genes upregulated and 249 downregulated in M(Hb)+OS–exposed HASMCs versus M(con)+OS (threshold: fold change [FC] ≥ 2.0 or ≤ –2.0 with *P* ≤ 0.05) (Figure 4, F and G, and Supplemental Table 2). To understand the biological significance of these transcriptional changes in M(Hb)+OS–exposed HASMCs, unbiased gene ontology (GO) enrichment analysis was conducted using the Database for Annotation, Visualization, and Integrated Discovery (DAVID) v6.8 online bioinformatics resources. The top 25 GO biological process (BP) terms with the highest statistical significance in up- and downregulated DEGs are shown in Figure 4, H and I. We found that the upregulated DEGs were enriched for inflammation, such as immune response (GO:0006955), inflammatory response (GO:006954), positive regulation of NF-κB transcription factor activity (GO:0051092), and positive regulation of IκB kinase/NF-κB signaling (GO:0043123) (Figure 4H). On the other hand, GO BP terms related to HASMC calcification (i.e., positive regulation of osteoblast differentiation [GO:0045669], cellular response to bone morphogenic protein [BMP] stimulus [GO:0071773], and BMP signaling pathway [GO:0030509]) were found in downregulated DEGs of M(Hb)+OS–exposed HASMCs (Figure 4I). Selected genes from up- and downregulated DEGs were visualized by heatmap with row *z* score scaling of log_2_ FC (Figure 4, J and K). As shown, transcripts related to inflammation and NF-κB signaling showed greater expression in the M(Hb) group (Figure 4J), while prior-reported calcification/osteogenic differentiation-related mRNA levels including *RUNX2* were downregulated in M(Hb)+OS–exposed HASMCs versus M(con)+OS group (Figure 4K). These results were validated by real-time PCR (Supplemental Figure 3). Since cell death is known as one of the principal causes of subsequent calcification, the effect of M(con) and M(Hb) supernatant for cell viability and apoptotic cell death was tested using in vitro assay. Viability of HASMCs was measured with PrestoBlue HS assay, and HASMC apoptosis was tested by TUNEL staining after exposing to M(con) or M(Hb) with or without OS. As shown in Supplemental Figure 4, no remarkable differences for cell viability and the degree of HASMC apoptosis were found between M(con) versus M(Hb) condition regardless of the presence of OS components.

### Activation of NF-κB and hyaluronan synthase with HASMCs is critical to the anticalcific mechanisms of CD163^+^ macrophages.

**Figure 5:**
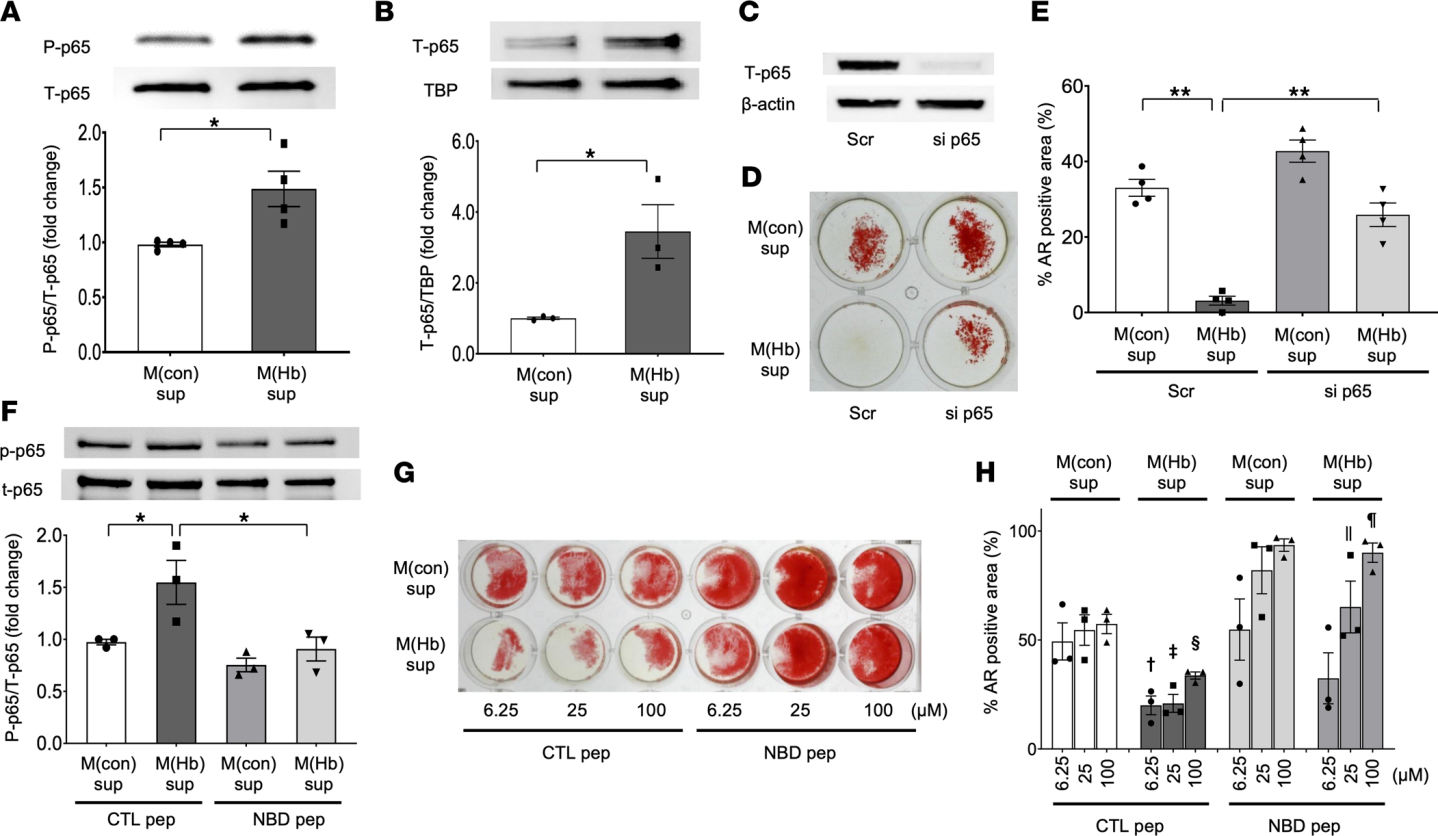
Stimulation of NF-κB signaling by M(Hb) supernatants directs its anticalcific effect on HASMCs. (**A** and **B**) Representative immunoblotting and summary of densitometry analysis for HASMCs after exposure to M(con) or M(Hb) supernatants for 24 hours without OS: p-p65/t-p65 (whole-cell lysate, *n* = 4 per group) (**A**) and t-p65/TBP (nuclear fraction, *n* = 3 per group) (**B**). (**C**–**E**) Immunoblotting image of t-p65/β-actin for HASMC whole-cell lysate transfected with the scrambled control siRNA or p65-siRNA (**C**). M(con)sup or M(Hb)sup with OS exposure (48 hours) was performed. Representative AR staining images (**D**) and summary of % AR-positive area (*n* = 4 per group) (**E**). (**F**) HASMC were exposed to M(con)sup or M(Hb)sup with CTLpep or NBDpep (both 25 μM) without OS for 24-hours. Representative WB images and summary of densitometry analysis for p-p65/t-p65 (whole-cell lysate, *n* = 3 per group). (**G** and **H**) HASMCs were exposed to M(con)sup or M(Hb)sup with OS and CTLpep or NBDpep (6.25, 25, and 100 μM) for 48 hours. Representative AR staining images (**G**) and summary of % AR-positive area (*n* = 3 per group) (**H**). *P < 0.05, **P < 0.01, ^†^*P* < 0.05 vs. M(con) CTLpep 6.25 μM, ^‡^*P* < 0.05 vs. M(con) CTLpep 25 μM, ^§^*P* 0.05 vs. CTLpep 100 μM, ^||^*P* < 0.05 vs. M(Hb) CTLpep 25 μM, ^¶^*P* < 0.05 vs. M(Hb) CTLpep 100 μM. Results are presented as the mean ± standard error (**A**, **B**, **E**, **F**, and **H**). *T* test was applied to **A** and **B**. ANOVA followed by post hoc Tukey’s test was applied to **E**, **F**, and **H**. Data normality was tested by Shapiro-Wilk test. All experiments were performed at least 3 times to confirm the reproducibility. CTLpep, IKK-NBD control peptide; NBDpep, NF-κB inhibitor NBD peptide; OS, osteogenic medium; p-p65, phosphorylated p65 (Ser536); t-p65, total-p65; Scr, scrambled control siRNA; si p65, p65-siRNA; TBP, TATA-binding protein; WB, Western blotting.

The relationship of NF-κB to VC is complex and incompletely understood. Al-Huseini et al. recently showed knockout of IKKβ, a catalytic subunit of NF-κB, which phosphorylates IκBα causing its degradation via the ubiquitin pathway, in VSMCs promoted VC through activation of β-catenin and RUNX2 (19). We previously demonstrated in cultured endothelial cells that exposure of M(Hb) supernatants increased canonical NF-κB signaling in part through VEGF (10). Given the results of our microarray analysis (Figure 4, H and J), we examined whether NF-κB was activated in HASMCs exposed to M(Hb) supernatants by Western blot analysis. Ser536 phosphorylation of p65 in whole-cell lysates and total p65 in nuclear fraction were significantly elevated in M(Hb) versus M(con) group (24-hour exposure) (Figure 5, A and B). Knocking down p65 via siRNA in HASMC significantly attenuated the anticalcific effect of M(Hb) supernatant as assessed by percentage AR-positive areas at a 48-hour time point as shown in Figure 5, C–E. Pharmacological suppression of NF-κB signaling using the NF-κB inhibitor NBDpep attenuated the activation of p65 phosphorylation (Figure 5F) and the anticalcific effect of M(Hb) under OS condition assessed by percentage AR-positive staining in a dose-dependent fashion (Figure 5, G and H). These data suggest that the anticalcific effect of M(Hb) supernatants is mediated by NF-κB activation in HASMCs.

**Figure 6:**
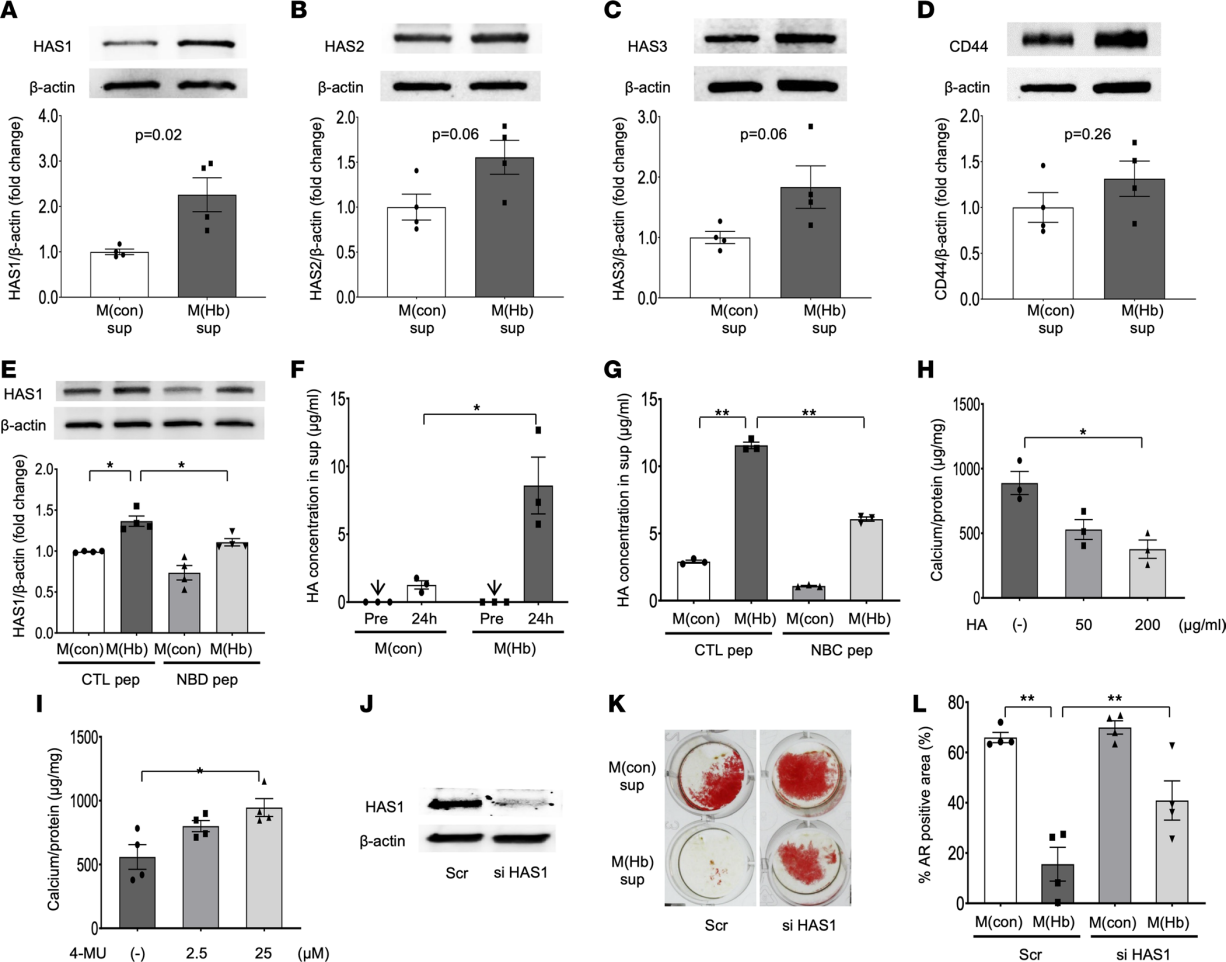
Augmented HA synthesis via NF-κB signaling by M(Hb) supernatants is responsible for its anticalcific effect on HASMCs. (**A**–**D**) Representative WB images and summary of densitometry analysis for HASMCs after exposing to M(con)sup or M(Hb)sup without OS for 24 hours (whole-cell lysate); HAS1/β-actin (**A**), HAS2/β-actin (**B**), HAS3/β-actin (**C**), and CD44/β-actin (**D**) (*n* = 4 in each). (**E**) HASMCs were exposed to M(con)sup or M(Hb)sup with CTLpep or NBDpep (both 25 μM) without OS for 24 hours. Representative WB images and summary of densitometry analysis for HAS1/β-actin (whole-cell lysate, *n* = 4 per group). (**F**) Summary of HA concentration in M(con) and M(Hb) supernatant pre- and post-exposing (24 hours) to HASMCs (ELISA, *n* = 3 per group). (**G**) Summary of HA concentration in M(con) and M(Hb) supernatant post-exposing (24 hours) to HASMCs with CTLpep or NBDpep (both 100 μM) without OS for 24 hours (*n* = 3 per group). (**H**) Calcium content in HASMCs after 48 hours’ exposure in OS to HA supplementation (0, 50, and 200 μg/mL) (colorimetric assay, *n* = 3 per group, adjusted by protein amount). (**I**) Calcium content in HASMCs after 24 hours’ exposure to basal growth medium with OS and 4-MU, an HAS inhibitor, (0, 2.5, and 25 μM) (colorimetric assay, *n* = 4 per group, adjusted by protein amount). (**J**–**L**) HASMCs were transfected with Scr or siHAS1. The effect of siHAS1 was confirmed by WB (**J**). M(con) or M(Hb) supernatant with OS was exposed for 48 hours. Representative AR images (**K**) and summary of % AR-positive area (*n* = 4 per group) (**L**). *P < 0.05, **P < 0.01. Results are presented as the mean ± standard error (**A**–**H** and **K**). *T* test was applied to **A**–**D**. ANOVA followed by post hoc Tukey’s test was applied to **E**–**I** and **L**. Data normality was tested by Shapiro-Wilk test. All experiments were performed at least 3 times to confirm their reproducibility. HAS, hyaluronan synthase; siHAS1, HAS1 siRNA; 4-MU, 4-methylumbelliferone.

HA is an ECM glycosaminoglycan released from VSMCs with potential anticalcific effects (20). Prior studies reported that HA synthase (HAS1, -2, and -3) expression is regulated by NF-κB signaling with evidence of a p65 binding site in the HAS promoter (21). Since our microarray data also suggest upregulation of HAS in HASMCs by M(Hb) exposure (Figure 4, G and J), protein expression of HASs was examined by Western blot analysis. Upregulation of all HAS subtypes was found in M(Hb)-exposed HASMCs (24 hours), and this effect was greatest for the HAS1 isoform (Figure 6, A–C). Changes in CD44 expression, a membrane protein associated with HA degradation, were relatively small after M(Hb) supernatant exposure (Figure 6D). Pharmacological suppression of NF-κB signaling by NBDpep, which blocks the interaction of NF-κB essential modulator with the IκB kinase complex, resulted in HAS1 downregulation as compared with CTLpep in M(Hb) supernatant–exposed HASMCs (Figure 6E). M(con) and M(Hb) supernatant alone contained quite low levels of HA as recognized by ELISA, but after exposure to HASMCs, the level of HA in M(Hb) supernatant dramatically increased after 24 hours (Figure 6F), suggesting HASMCs released HA into the media. Augmented HA production by M(Hb) supernatant was suppressed by the presence of NBDpep (Figure 6G). HA attenuated calcium deposition when directly exposed to HASMCs under OS conditions while pharmacological HAS inhibition by 4-methylumbelliferone, a well-known HAS inhibitor by depletion of cellular UDP-glucuronic acid (22), exacerbated HASMC calcium deposition in a dose-dependent fashion (0, 2.5, and 25 μM for 24-hour exposure) (Figure 6, H and I). Knockdown of HAS1 in HASMCs by siRNA significantly attenuated the anticalcific effect of M(Hb) supernatant assessed by percentage AR-positive area (Figure 6, J–L). These results suggest the anticalcific effect of M(Hb) supernatant on HASMCs is derived from NF-κB stimulation and subsequent HA production by HASMCs. In our prior work, we found that M(Hb) supernatant contains a large amount of VEGF released from macrophages exposed to HH (10). HA is transcriptionally regulated by NF-κB (23). We examined whether HA production within M(Hb)-exposed HASMCs was due to the effect of VEGF. The effects of 2 biological VEGF receptor inhibitors (i.e., axitinib [inhibitor of VEGF receptors 1, 2, and 3] and cabozantinib [inhibitor of VEGF receptor 2]) were tested. M(Hb) supernatant–induced HA production was attenuated by both reagents in a dose-dependent fashion (Supplemental Figure 5).

### Deletion of CD163 in mice attenuates NF-κB signal stimulation and HA production in atherosclerotic lesions.

**Figure 7:**
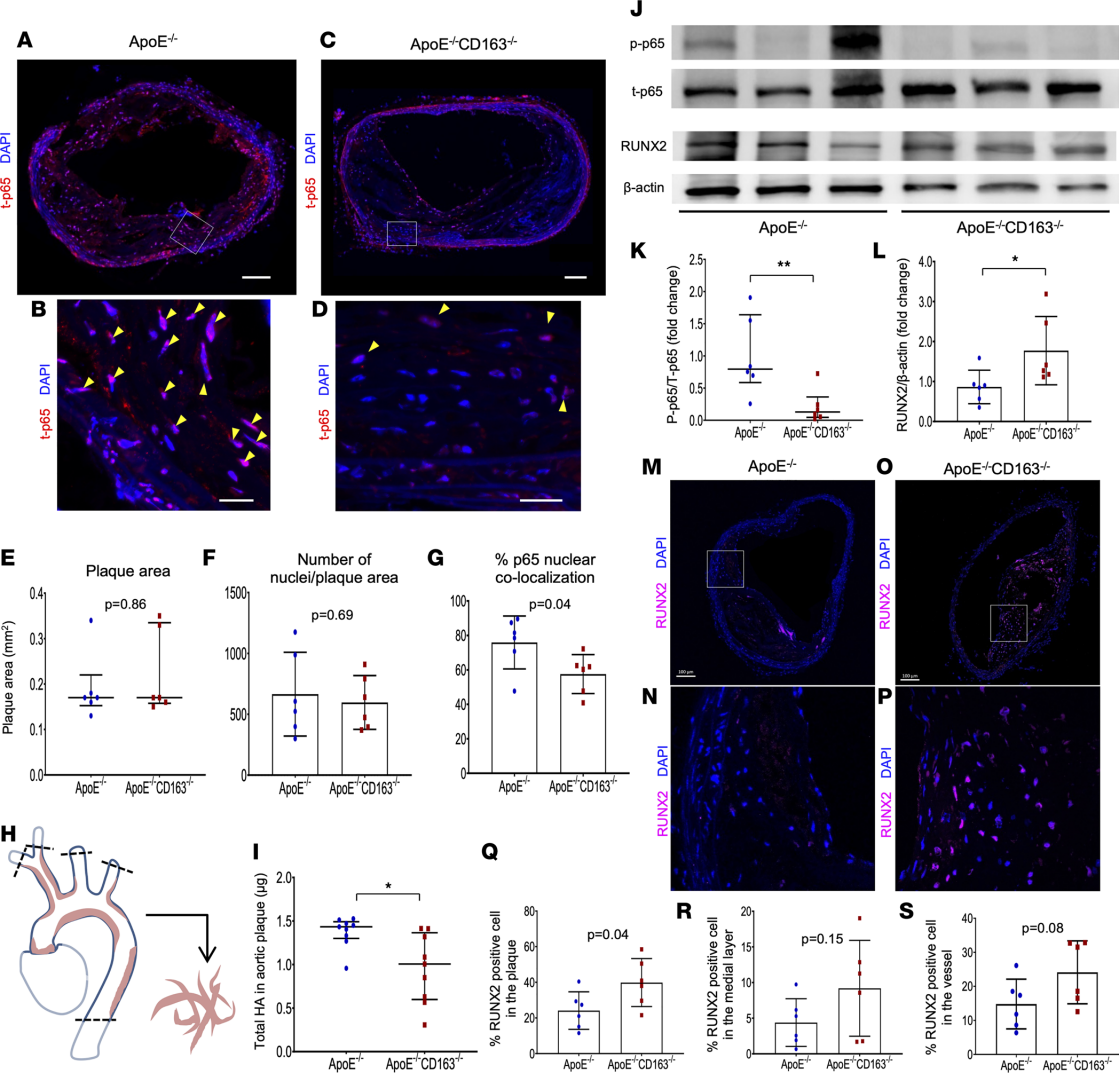
NF-κB signaling, HA, and RUNX2 expression in plaques of aged *ApoE^–/–^* versus *ApoE^–/–^* *CD163^–/–^* mice. (**A**–**D**) Representative low- and high-power images of BCA sections with total p-65 immunofluorescence (red) and DAPI counterstaining obtained from aged *ApoE^–/–^* (**A** and **B**) and *ApoE^–/–^* *CD163^–/–^* (**C** and **D**) mice (1.5-year-old). Nuclei in the plaque showing t-p65 colocalization are indicated by yellow arrowheads. (**E**–**G**) Summary of plaque area (**E**), number of nuclei/plaque area (**F**), and % p65 nuclear colocalization (**G**) in the most stenotic BCA lesions were compared between *ApoE^–/–^* and *ApoE^–/–^* *CD163^–/–^* mice (*n* = 6 per group). (**H**) Schematic diagram of the methodology for plaque extraction from mouse aortas. Whole visible plaques from ascending (including aortic root) to descending (cut at the level of diaphragm) aorta were peeled out under dissecting microscope. Plaques in BCA, left common carotid, and subclavian arteries with the equivalent length with BCA were also included in the samples. Plaques were mechanically ground in equivalent volumes of PBS, and further ELISA and WB analyses were performed. (**I**) HA content in aortic plaque assessed by ELISA (*n* = 9 per group). (**J**–**L**) Representative WB images for mouse plaque samples including p-p65, t-p65, RUNX2, and β-actin (**J**). Summary of densitometry analysis is also shown in **K** (p-p65/t-p65) and **L** (RUNX2/β-actin) (*n* = 6 per group). (**M**–**P**) Representative low- and high-power images of BCA sections with RUNX2 staining (purple) obtained from aged *ApoE^–/–^* (**M** and **N**) and *ApoE^–/–^* *CD163^–/–^* (**O** and **P**) mice (1.5-year-old). (**Q**–**S**) Summary of % RUNX2^+^ cells in the plaque (**Q**), % RUNX2^+^ cells in the medial layer (**R**), and % RUNX2^+^ cells in the vessel (plaque+medial layer) (**S**) in the most stenotic BCA lesion were compared between *ApoE^–/–^* and *ApoE^–/–^* *CD163^–/–^* mice (*n* = 6 per group). *P < 0.05, **P < 0.01. Results are presented as the mean ± standard deviation (**F**, **G**, **L**, and **Q**–**S**) or median and interquartile range (**E**, **I**, and **K**). *T* test (**F**, **G**, **L**, and **Q**–**S**) or Mann-Whitney test (**E**, **I**, and **K**) was conducted for statistical analysis. Data normality was tested by Shapiro-Wilk test. Scale bars: 100 μm (**A** and **C**), 20 μm (**B** and **D**), and 100 μm (**M**–**P**).

To further demonstrate the role of CD163 on NF-κB signaling, HA, and calcification in the plaque formation, BCA plaque samples from aged *ApoE^–/–^* and *ApoE^–/–^* *CD163^–/–^* mice (1.5 years old with normal chow feeding) were examined. Immunofluorescence staining of total p65 in BCA sections were compared between the 2 groups using the most stenotic section (Figure 7, A–D). Although plaque area and number of nuclei/plaque area were equivalent between the 2 groups, percentage p65 nuclear colocalization was significantly greater in *ApoE^–/–^* compared with *ApoE^–/–^* *CD163^–/–^* (Figure 7, E–G). Next, the luminal side of the aorta was exposed by longitudinal cutting of the aortic wall, and the whole visible plaque from ascending to descending aorta were peeled out using a dissecting microscopic (Figure 7H). Plaques were mechanically ground in equivalent volumes of PBS. The amount of HA in the plaque was assessed by ELISA and demonstrated significantly greater HA in *ApoE^–/–^* versus *ApoE^–/–^* *CD163^–/–^* mice (Figure 7I). Immunoblotting analysis of samples peeled out from whole aortic plaque showed greater level of p65 phosphorylation in *ApoE^–/–^* mice while greater expression of RUNX2 in *ApoE^–/–^* *CD163^–/–^* was observed (Figure 7, J–L). Further, immunofluorescence staining of RUNX2 in BCA sections revealed greater number of RUNX2^+^ cells in the vessels of *ApoE^–/–^* *CD163^–/–^* versus *ApoE^–/–^* mice (Figure 7, M–S). These findings are consistent with the prior in vitro experimental results.

To explore the role of Hb in phenotypic modulation of macrophage behavior with respect to HA production by HASMCs, we isolated peritoneal macrophages from *CD163^–/–^* mice (10). Our prior work confirmed that exposure of peritoneal macrophages to mouse Hb (0.1 mg/mL) overnight resulted in significant release of VEGF-A in WT but not *CD163^–/–^* mice (10). (Ingestion of Hb by mouse CD163^+^ macrophages does not seem to be as dependent on haptoglobin binding as human CD163 is; ref. 24.) Moreover, treatment of HASMCs with supernatants from WT mouse peritoneal macrophages exposed to Hb resulted in significant enhancement of p65 phosphorylation, HAS (mainly HAS1) gene expression, and HA production in an in vitro assay (Supplemental Figure 6). There was no effect of supernatants from Hb-exposed, CD163-deficient macrophages in these assays with respect to these endpoints (Supplemental Figure 6). Collectively, these results suggest that CD163 mediates the NF-κB stimulation and subsequent HA production in smooth muscle cells (SMCs).

### Pharmacological suppression of NF-κB signaling in atherogenic ApoE^–/–^ mice suppresses plaque formation but exacerbates calcium deposition with a lower level of HA expression.

**Figure 8:**
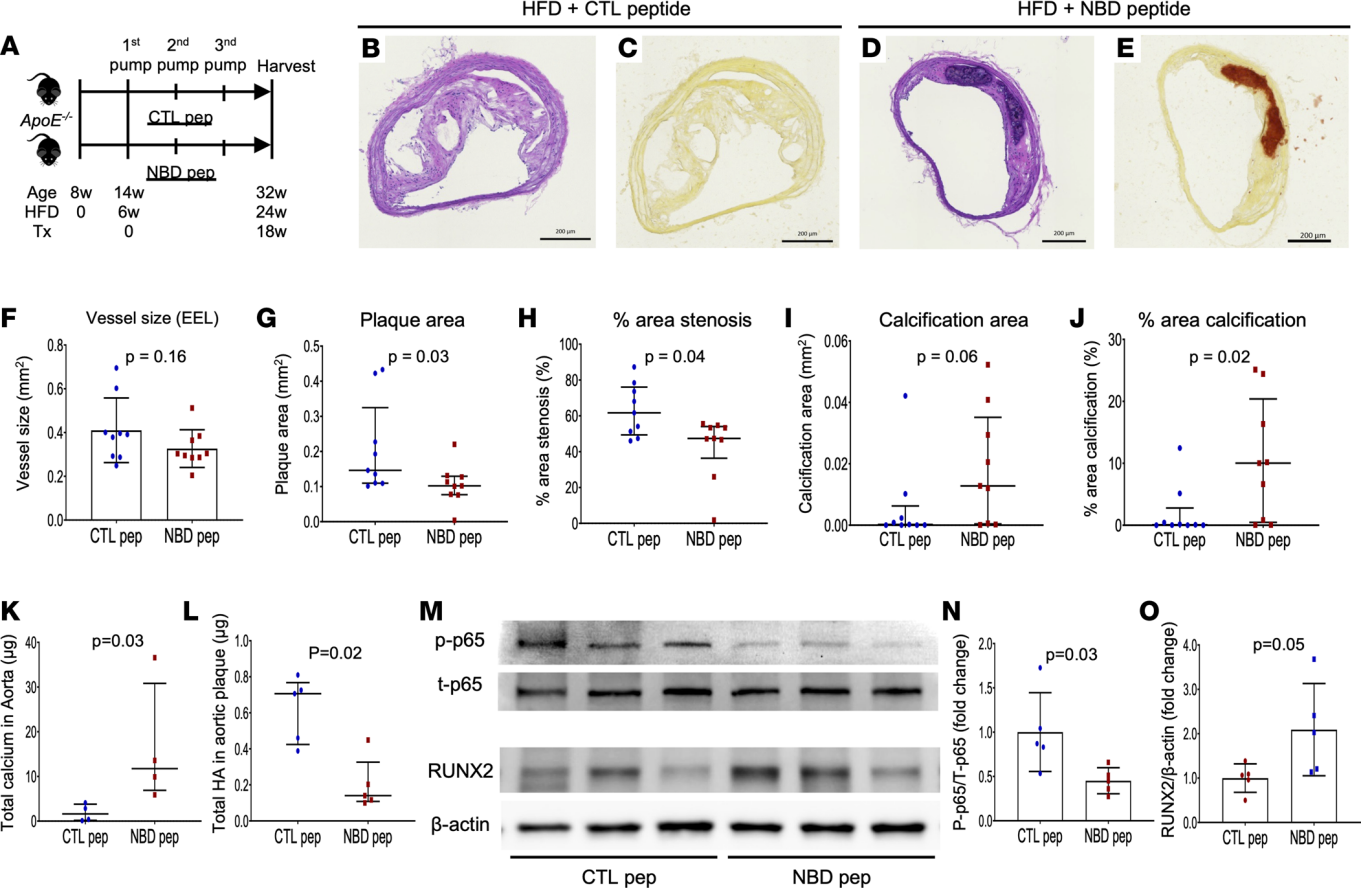
Effect of NF-κB inhibition by NBDpep on development of atheroma and VC in *ApoE^–/–^* mice with HFD feeding. (**A**) Schematic diagram of study design. HFD was started in *ApoE^–/–^* mice at the age of 8 weeks and continued to the end of the experiment. Treatment by continuous subcutaneous injection of CTLpep or NBDpep by Alzet osmotic pump (100 μg/kg/d) was started at the age of 14 weeks. Since the pump needed to be replaced every 6 weeks, pump replacing surgeries were performed, and Tx was continued for 18 weeks (by the age of 32 weeks). (**B**–**E**) Representative BCA sections with H&E and AR staining obtained from HFD feeding *ApoE^–/–^* mice with CTLpep (**B**; H&E, **C**; AR) or NBDpep Tx (**D**; H&E, **E**; AR). (**F**–**J**) Vessel size (EEL) (**F**), plaque area (**G**), % area stenosis (**H**), calcification area assessed by AR (**I**), and % area calcification (**J**) at the most stenotic BCA lesion were compared between CTLpep- and NBDpep-treated *ApoE^–/–^* mice (*n* = 9 per group). (**K** and **L**) Total aortic calcium contents assessed by colorimetric assay (*n* = 4 per group) (**K**) and HA content in aortic plaque assessed by ELISA (*n* = 5 per group) (**L**) with the comparison of CTLpep- and NBDpep-treated *ApoE^–/–^* mice. (**M**–**O**) Representative WB images of mouse plaque samples including p-p65 (Ser536), t-p65, RUNX2, and β-actin (**M**). Summary of densitometry analysis is shown in **N** (p-p65/t-p65) and **O** (RUNX2/β-actin) (*n* = 5 in each). Results are presented as the mean ± standard deviation (**F**, **N**, and **O**) or median and interquartile range (**G**–**L**). *T* test (**F**, **N**, and **O**) or Mann-Whitney test (**G**–**L**) was conducted for statistical analysis. Data normality was tested by Shapiro-Wilk test. Scale bars: 200 μm (**B**–**E**). Tx, treatment.

For further exploring the role of NF-κB signaling in plaque formation and calcification, we used the selective canonical NF-κB inhibitor i.e., NBDpep, in the *ApoE^–/–^* mouse model (25). High-fat diet (HFD) was started at the age of 8 weeks (6 weeks before initiating drug administration) and continued to the end of the experiment to develop adequate volume of plaque. Subcutaneous administration of IKK-NBD CTLpep or NBDpep (both 100 μg/kg/d, Enzo Life Sciences) by Alzet osmotic pump was started at the age of 14 weeks (6 weeks after initiating HFD). Drug administration was continued for 18 weeks (until the age of 32 weeks). A schematic diagram of study design is shown in Figure 8A. After euthanasia, BCA sections were stained with H&E and AR for the analysis of plaque morphology and calcification (Figure 8, B–E). Although vessel size was equivalent, plaque area and percentage area stenosis were significantly greater in the CTLpep than NBDpep treatment group (*P* = 0.03 and 0.04) (Figure 8, F–H). However, calcification area assessed by AR staining was relatively smaller with borderline significance (*P* = 0.06), and percentage area calcification of the plaque was significantly smaller in CTLpep- versus NBDpep-treated *ApoE^–/–^* mice (*P* = 0.02) (Figure 8, I and J). Total calcium extracted from thoracic aorta was significantly greater, and total HA contents in the plaque peeled out from aorta were markedly smaller in the NBDpep group (*P* = 0.03 and 0.02) (Figure 8, K and L). Moreover, immunoblotting data of mouse plaque showed significant suppression of p65 phosphorylation and greater expression of RUNX2 in NBDpep- versus CTLpep-treated mice (*P* = 0.03 and 0.05) (Figure 8, M–O). These results indicate suppression of NF-κB signaling in the plaque leads to plaque volume regression but increases calcium deposition, which is suggestive of a more stable atheroma phenotype and implies a disconnect between inflammation and calcification with atherosclerotic plaques.

### CD163 is associated with NF-κB signaling stimulation, HA production, and reduced calcification in human arteries.

**Figure 9:**
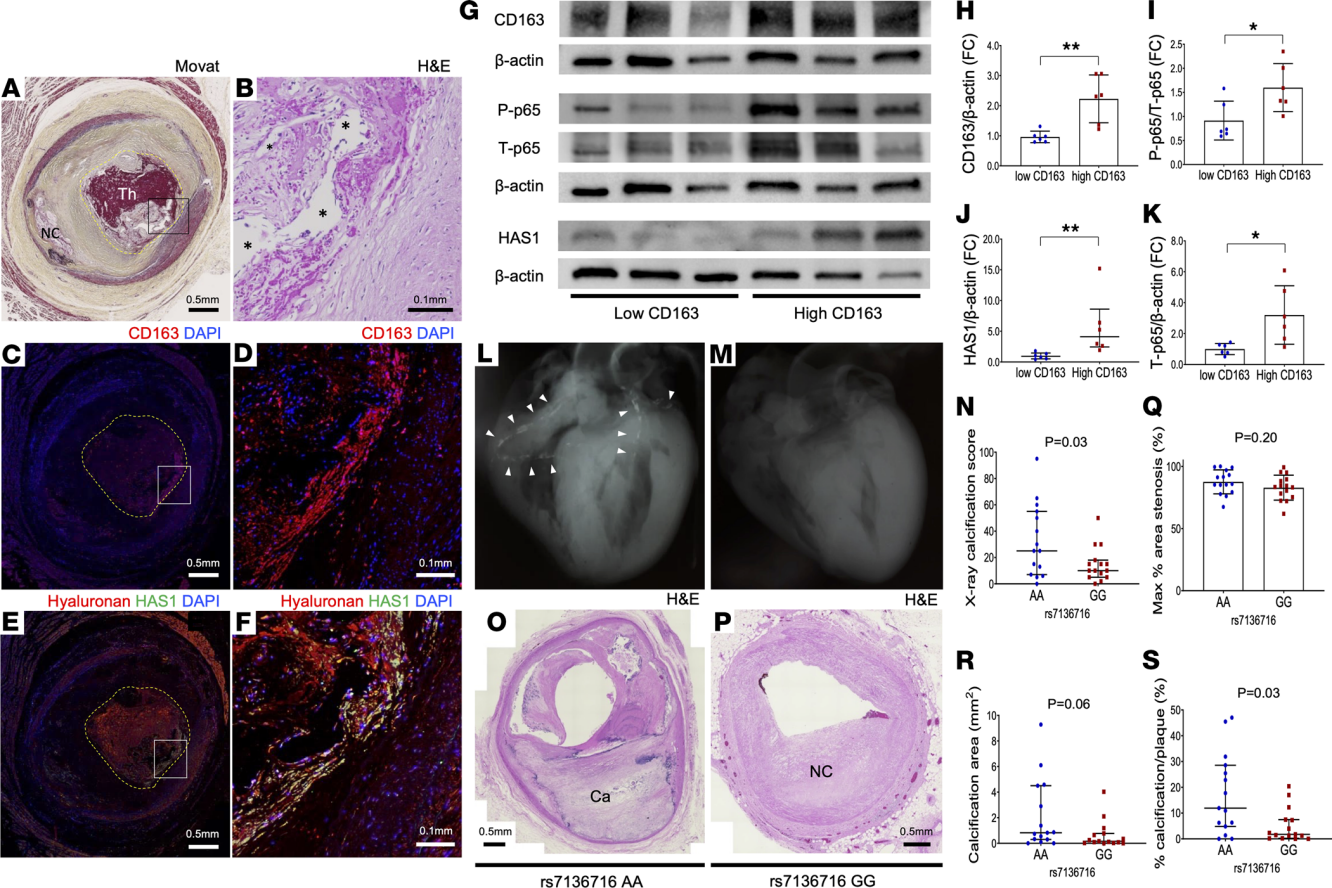
CD163 activity and its association with NF-κB signaling, HA synthesis, and calcification in human atherosclerotic arteries. (**A**–**F**) CTO section of left circumflex artery (LCX) from 37-year-old man who died from an acute coronary syndrome (culprit plaque rupture was found in Left anterior descending artery, LAD). Low-power Movat (**A**) and high-power H&E (**B**) image of the corresponding black rectangle in **A**. Yellow-dot border of occluded lumen with thrombus. Fibrin deposition and neo-angiogenesis (*) in the occluded lumen were observed. CD163 (red) (**C** and **D**) and HA (red)/HAS1 (green) (**E** and **F**) immunofluorescence images of the adjacent sections of **A**. (**G**–**K**) Representative immunoblotting (**G**) and summary of densitometry analysis (**H**–**K**) for CD163, p-p65 (Ser536), t-p65, HAS1, and β-actin of protein extracted from human carotid atheroma expressing high or low levels of CD163 (*n* = 6 per group). *P < 0.05, **P < 0.01. (**L**) X-ray image of postmortem heart from 41-year-old African American with WT allele of the rs7136716 (AA genotype). White arrowheads indicate severe calcification in the coronary tree. (**M**) X-ray image of postmortem heart from 44-year-old African American with 2 copies of the minor allele for rs7136716 (GG genotype) without visible coronary calcification. Pathology of coronary arteries revealed triple-vessel disease. (**N**) Summary of ex vivo x-ray–based calcification score in age-matched AA versus GG genotype carriers (*n* = 15 per group). (**O**–**S**) Representative histopathology images of coronary artery sections (H&E) from AA (**O**) and GG (**P**) genotype carriers. Percentage area stenosis (**Q**), total calcification area (**R**), and % calcification/plaque area (**S**) between 2 genotype carriers. Results are presented as the mean ± standard deviation (**H**, **I**, **K**, and **Q**) or median and interquartile range (**J**, **N**, **R**, and **S**). *T* test (**H**, **I**, **K**, and **Q**) or Mann-Whitney test (**J**, **N**, **R**, and **S**) was conducted for statistical analysis. Data normality was tested by Shapiro-Wilk test. Scale bars: 0.5 mm (**A**, **C**, and **E**), 0.1 mm (**B**, **D**, and **F**), 0.5 mm (**O** and **P**).

To support our results in human plaques, we examined human coronary CTO sections with thrombotic occlusion of the lumen with erythrocytes encased within organizing thrombus (Figure 9, A–F). Representative H&E; Movat; and CD163, HA, and HAS1 immunofluorescence images of human coronary CTO sections are shown in Figure 9, A–F. In areas of the occluded lumen (not original plaque), HA- and HAS1-expressing cells were abundantly observed (Figure 9, E and F). To verify these results, protein was extracted from CD163^hi^ plaques and compared with protein extracted from CD163^lo^ plaques (as previously described, ref. 10) in human frozen CEA samples. Immunoblotting for CD163, phosphorylated p65, total p65, and HAS1 revealed that plaques rich in CD163^+^ cells had greater phosphorylation of p65 and total p65 itself, as well as HAS1 expression, than CD163^lo^ plaques (Figure 9, G–K).

Next, a single nucleotide polymorphism (SNP) in the CD163 gene was examined in a cohort of 346 African Americans from CVPath Registry. We previously reported homozygous G allele carriers of rs7136716 being significantly associated with coronary plaque rupture with greater expression of CD163^+^ macrophages as well as microvessel density in the culprit ruptured site in African Americans (10). (Note, individuals of European descent have a lower prevalence of this SNP, with a minor allele frequency of 11%–12% vs. approximately 50% for individuals of African American descent.) To further explore the effect of the homozygous minor alleles on VC, we examined age-matched 30 victims of sudden coronary death with severe CAD carrying homozygous major (*n* = 15) versus minor allele (*n* = 15) genotypes. Case clinical background and pathologic characteristics are shown in Supplemental Table 3. The degree of coronary artery calcification by x-ray in the autopsy hearts was semiquantitated in a blinded manner for their rs7136716 genotypes. According to the x-ray, coronary arteries were divided into 10 segments (i.e., left main trunk and proximal, middle, and distal of each RCA, LAD, and LCX). The semiquantitative calcification score of each segment was evaluated on a 0 to 10 scale. Total calcium score in 10 segments was considered the score for each case (0 to 100). Representative ex vivo x-ray images of major AA and minor GG genotype carriers are shown in Figure 9, L and M, and Supplemental Figure 7. Semiquantitated coronary artery calcification by x-ray was significantly greater in AA versus GG genotype carriers (Figure 9N). Moreover, 3 sections from the most stenotic lesion were selected from the RCA, LAD, and LCX proximal sites (i.e., 9 sections/case). Representative pathology sections of 2 genotype carriers are shown in Figure 9, O and P. Although the plaque area and percentage stenosis were similar, calcification area and percentage calcification/plaque area were greater in AA versus GG genotype carriers (Figure 9, Q–S, and Supplemental Table 3).

**Figure 10:**
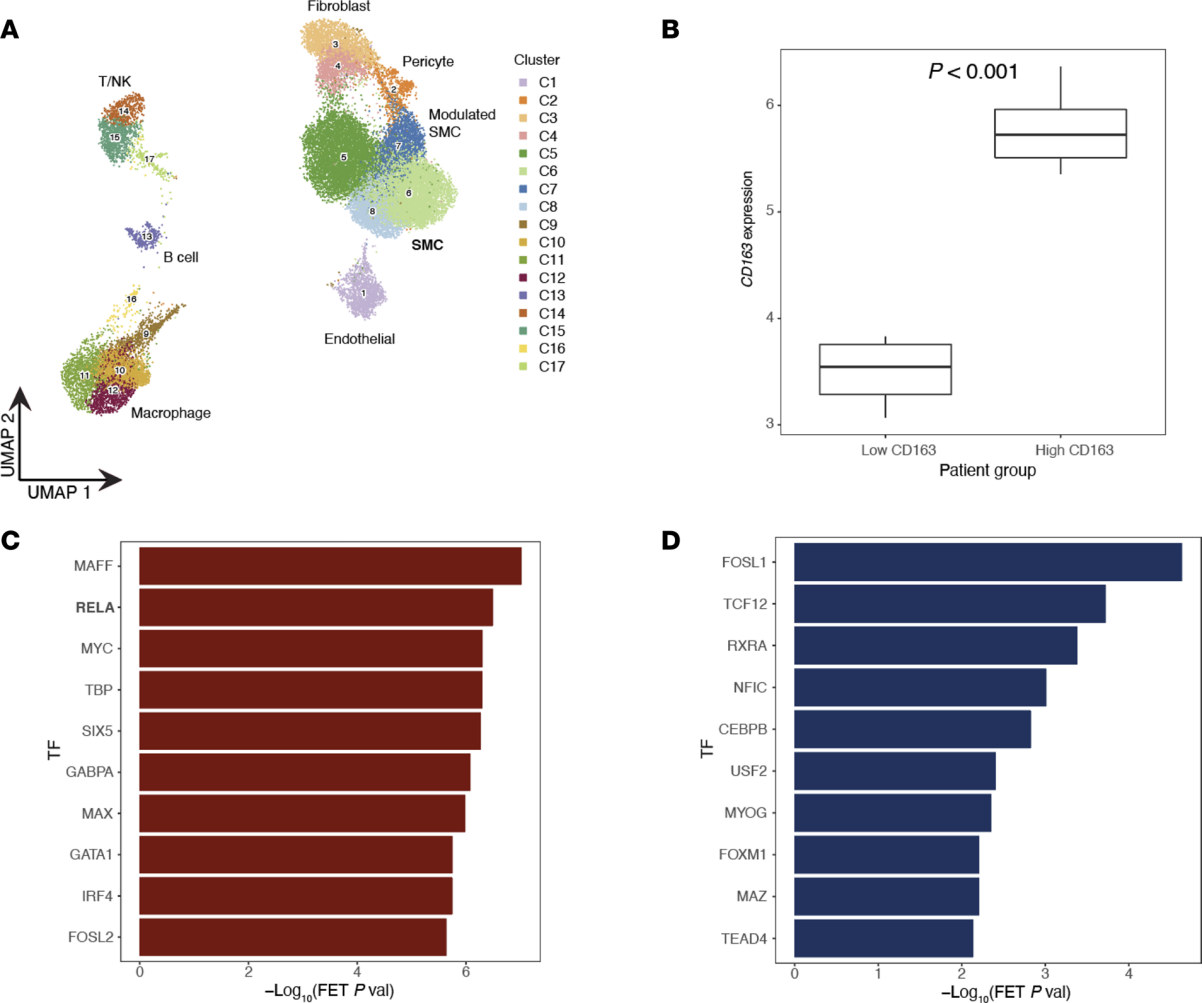
scRNA-Seq data analysis for human coronary artery samples. (**A**) UMAP embeddings of human coronary artery (HCA) lesion scRNA-Seq data (26) integration into HCA snATAC-Seq data of samples with varying clinical presentations of CAD (41 individuals, ~30,000 cells) (27). scRNA-Seq cell type labels and expression profiles were transferred to snATAC-Seq clusters using the ArchR implementation (29) of canonical correlation analysis (28) to maximize the number of cells available for gene expression statistical analyses. The UMAP plot shows 8 identified major cell types including macrophages, contractile/modulated SMCs, endothelial cells, fibroblasts, B cells, pericytes, and a mixed population of T/NK cells. (**B**) Box plot depicting stratification of individual patients based on the CD163 mean expression level of their corresponding macrophages. Individuals below the CD163 mean macrophage expression distribution 25% quartile were denoted as low CD163 (*n* = 11, norm expression < 3.87), whereas individuals above the distribution 75% quartile were denoted as high CD163 (*n* = 11, norm expression > 5.33). Box plots show the median and interquartile range with upper (75%) and lower (25%) quartiles shown. The difference in CD163 mean expression between low and high CD163 patients was statistically significant (*P* < 0.001) as calculated by an unpaired Student’s *t* test. (**C** and **D**) Bar plots depicting enrichment of TF in gene sets from a transcriptome-wide differential expression analysis of SMCs from high versus low CD163 patients. Genes were ranked based on the mean expression difference between the 2 conditions, and the top 100 genes upregulated in CD163^hi^ SMCs (**C**) as well as top 100 genes upregulated in CD163^lo^ groups (**D**) were used for TF target overrepresentation analyses using ChEA3 (30). The *x* axis of the plot denotes the negative log_10_ of the calculated Fisher Exact Test (FET) *P* value. Metadata for each patient within high and low CD163 groups can be found in Supplemental Table 5.

Last, to further corroborate the directionality between CD163, NF-κB signaling, and osteogenic marker expression at the single-cell level, we leveraged public human coronary lesion single-cell RNA-Seq (scRNA-Seq) data (26) and our recently published single-nucleus ATAC-Seq (snATAC-Seq) atlas of human coronary arteries with varying clinical presentations of CAD (41 individuals, ~30,000 cells) (27). To maximize the number of cells available for gene expression statistical analyses, cell type labels and gene expression profiles were transferred from scRNA-Seq data to snATAC-Seq clusters using the ArchR implementation of canonical correlation analysis (28, 29). After harmonizing these 2 data sets, uniform manifold approximation and projection (UMAP) plot showed 8 lesion major cell types including macrophages, SMCs (contractile and ECM-rich modulated), endothelial cells, fibroblasts, B cells, pericytes, and a mixed population of T and natural killer (T/NK) cells (Figure 10A). To begin investigating effects of CD163^+^ macrophage expression on calcification, we stratified individuals based on the CD163 mean expression level of their corresponding macrophages. Individuals below the CD163 mean macrophage expression distribution 25% quartile were denoted as CD163^lo^ (*n* = 11) whereas individuals above the distribution 75% quartile were denoted as CD163^hi^ (*n* = 11) (Supplemental Methods and Supplemental Table 5). Prior to downstream analyses, we confirmed that the difference in expression between low and high CD163 groups was statistically significant (*P* = 9.33 × 10^–14^) (Figure 10B). To obtain a more global view of CD163 and NF-κB signaling, we examined SMCs from both high (*n* = 1,725) and low (*n* = 1,303) CD163 groups and performed a *t* test–based differential expression analysis (Supplemental Table 6).

We then examined the top 100 genes upregulated in high and low CD163 SMCs for gene set enrichment analysis (GSEA) and TF overrepresentation analysis. Given the interest in the effect of CD163 on SMC-induced calcification, we performed GSEA of genes that were upregulated in CD163^lo^ SMCs and found consistent enrichment of ECM and calcification-related terms, such as extracellular matrix organization, ossification, and bone, across 3 different ontologies (Supplemental Methods and Supplemental Tables 7–9), supporting an inverse association between CD163 expression and SMC-induced calcification. Furthermore, to uncover underlying signaling across high and low CD163 SMCs more globally, we used these gene sets for TF target overrepresentation analysis using ChEA3 (30). Importantly, we found *RELA* (NF-κB p65 subunit) as one of the most highly enriched TFs within the gene set upregulated in CD163^hi^ SMCs (Figure 10C), supporting activation of NF-κB signaling. Among TFs enriched in CD163^lo^ SMCs, we found factors previously shown to be active in human contractile and ECM-rich SMCs such as TEAD4 and FOSL1 (Figure 10D) (27). These results further support an anticalcific effect of CD163^+^ macrophages in atherosclerotic lesions through activation of NF-κB signaling in SMCs.

## Discussion

Macrophages located in atherosclerotic lesions have a heterogeneous phenotype induced by different stimuli (7). Although macrophages in atheromas are considered a source of calcification, apart from classical foamy macrophages, alternative macrophages could play a different role in plaque development including calcification (31). In this study, we have explored an important aspect regarding a paradox observed in human lesions in which often more stable lesions are heavily calcified while thin-cap fibroatheromas and ruptured plaques demonstrate significantly less calcification. IPH induces plaque progression into high-risk vulnerable plaque phenotypes with large NC and is accompanied by infiltration of CD163^+^ alternative macrophages. We previously showed CD163^+^ macrophages promote plaque progression through mechanisms such as inflammatory cell recruitment and leaky angiogenesis (10), but their role on plaque calcification has not been explored. Here we report for the first time that CD163^+^ alternative macrophages at the sites of IPH prevent atherosclerotic VC. Athero-prone mice with genetic deletion of CD163 demonstrated greater lesion calcification compared with WT mice. Moreover, this anticalcific effect is mediated by NF-κB stimulation within VSMCs and NF-κB–mediated production of HA. In mice, systemic inhibition of NF-κB decreased atheroma progression but promoted VC. Together our results suggest that plaque progression itself is driven by CD163^+^ macrophage–induced inflammatory activation within VSMCs, which at the same time inhibits lesion calcification.

### Macrophage subtype and VC.

Macrophages in the plaque have been reported to have both pro- and anticalcific roles during VC formation. Release of pro-inflammatory cytokines, reactive oxygen species, apoptosis, and matrix vesicles have been explained as key factors of macrophages for promoting VC, while mineral dissolution as osteoclast-like activity was reported for an anticalcific modulation by macrophages (32, 33). Recent investigations have suggested the macrophage phenotypes in the plaque are remarkably diverse, induced by multiple stimuli for their differentiation and maturation; therefore, several subpopulations might have different roles in VC formation. Chinetti-Gbaguidi et al. have shown impaired osteoclastic activity in alternative macrophages expressing both CD68 and mannose receptor and their contribution for VC acceleration (31). The subpopulation of macrophage we investigated in the current study is also an alternative macrophage but another subtype, which is called M(Hb), found at sites of IPH. In fact, our observation in human carotid arteries clearly showed less microcalcification found in lesions rich in CD163^+^CD68^+^ macrophages compared with areas occupied by CD163^–^CD68^+^ cells, suggesting the different effect of macrophage subpopulation on the process of VC (Figure 1).

### IPH, macrophages, and VC.

IPH is considered one of the factors contributing to plaque destabilization and sudden increase in plaque volume (34, 35). However, the relationship between IPH and VC is not well understood. Recently, Tziakas et al. proposed the potential procalcific mechanism of erythrocyte membranes on VSMC osteogenic differentiation at the sites of IPH (13, 14). In their study, the authors added lysed erythrocyte membrane fraction without hemoglobin to the osteogenic media and found greater calcium deposition in cultured VSMCs compared with no erythrocyte membrane condition. Nitric oxide generated by erythrocyte membrane was considered the key procalcific mechanism, and the authors pointed out a potential role for hemoglobin as a scavenger of nitric oxide and its possible contribution for the counterbalance on the erythrocyte membrane-derived procalcific activity (36). Although our in vitro experimental setting did not include erythrocyte membrane fraction, the HH complex itself did not affect the result of in vitro VSMC calcification in our study, and only M(Hb) supernatant showed remarkable anticalcific effect (Figure 4). Moreover, we studied the association of M(Hb) cells with VC in human atherosclerotic lesions as well as a *CD163^–/–^* mouse model and found CD163 to be associated with lower VC. Since IPH is not a simple biologic event but instead involves multiple types of cells (not just erythrocytes) in temporal sequence, complex mechanisms probably govern the effect of IPH on VC. Therefore, it is not surprising that erythrocyte membranes and hemoglobin via M(Hb) may have different effects on VC formation. Of note, in the pathology of human atherosclerosis, the NC filled with apoptotic lipid-laden foamy macrophages is known as the most susceptible area for calcification in the plaque (37), probably due to the excessive accumulation of cellular debris containing calcium and phosphate. Since IPH is also frequently found in the NC areas, analyzing the effect of M(Hb) on VC may be complicated by its proximity to the NC. Therefore, we applied the thrombus occluded lumen at human coronary CTO sections for the further assessment of CD163 and VC. Although there is an intrinsic weakness of pathologic observational studies (i.e., causation cannot be explored in itself), as shown in Figure 2, most of the calcification was found in the original plaque area, and almost no calcification was observed at the site of occluded lumen where CD163^+^ macrophages are abundantly found in the initial thrombus organizing area. In line with this observation, advanced fibrosis is a common finding of the site at deep venous thrombosis, but calcification is quite a rare observation (38). These observations further support our data concerning the anticalcific mechanisms of CD163^+^ macrophages.

### Contribution of HA on anticalcification in the plaque.

The M(Hb) macrophage is inducible in vitro by HH complexes. To elucidate the anticalcific effect of M(Hb), supernatant supplemented with OS was applied to VSMCs in the current study. Accordingly, in vitro calcification of VSMCs assessed by multiple assays supported the anticalcific effect of M(Hb) supernatant. Several calcification-related genes as well as protein levels of RUNX2, a master regulator of bone development and a critical TF for VSMC osteogenic differentiation in the plaque (39), were markedly suppressed by M(Hb) compared with M(con) supernatant. Simultaneously, we explored the specific changes in VSMCs exposed to M(Hb) supernatant by analyzing microarray data obtained from VSMCs exposed to M(con) or M(Hb) with OS and found the expression levels of HASs were markedly augmented in the M(Hb) group. It is well known that ECM molecules, which are mainly synthesized by VSMCs, play crucial roles in plaque volume progression, lipid retention, and inflammatory cell trafficking (40, 41). Further, a number of studies have also demonstrated ECM molecules such as matrix Gla protein, osteoprotegerin, decorin, and HA have critical roles for regulating VC (20, 42–44). Kong et al. suggested HA attenuates VSMC osteogenic differentiation and VC (20). Moreover, in a previous human histopathology study, great HA accumulation in the lipid pool in pathologic intimal thickening (PIT), an early type of atherosclerotic lesion, was reported. However, the volume of HA is dramatically attenuated during NC development in the late lesions of human plaques (i.e., as lesions progress from PIT to early then late FA) (40). In contrast, calcification in the large NC of FA is much larger than in the lipid pool in PIT. This inverse correlation between HA and calcification in the development of advanced atheroma lends further support to the anticalcific effect of HA in human atheroma (40). In the current study, M(Hb) macrophage supernatant–exposed VSMCs released HA into the surrounding media. The biologic effect of HA in VSMCs was in part responsible for the anticalcific properties of M(Hb) and was supported by mechanistic pharmacological and gene knockdown experiments. Moreover, in vivo evidence of less HA expression in the plaque accompanied by greater calcification was observed in CD163-knockout athero-prone mice and in human plaque data examining colocalization of CD163, HA, and HAS.

### NF-κB signaling in VSMCs and VC.

We also found that NF-κB activation was responsible for HA production in VSMCs after stimulation with M(Hb) macrophage supernatants. Prior studies have reported NF-κB directly regulates transcriptional level of HASs in endothelial cells and other cell types (21, 45). Our microarray data of M(con) and M(Hb) supernatant–exposed VSMCs as well as immunoblotting data of plaque from *ApoE^–/–^* mice with or without CD163 codeletion revealed greater NF-κB signaling stimulation in the presence of CD163. According to our in vivo data, both genetic deletion of CD163 and pharmacological inhibition of NF-κB resulted in attenuation of the HA and aggravation of calcification in the plaque. NF-κB is known as a pivotal mediator of inflammatory responses (46), and its contribution to atherosclerosis is well documented (47). However, the role of NF-κB signaling on VC seems to be rather complex (19, 48, 49). Al-Huseini et al. investigated the contribution of NF-κB signaling in the VSMC for VC development (19). They applied genetically modified mice with SMC-specific deletion of IKKβ, an essential kinase for NF-κB activation. Both in vivo experiment with CaCl_2_-mediated abdominal aorta injury and in vitro calcification study of VSMCs showed exacerbation of VC accompanied by greater RUNX2 upregulation by IKKβ deletion compared with WT control. Indeed, NBDpep, which we used in our in vitro and in vivo experiments, inhibits the exact same molecular target (i.e., the IκB-kinase complex) and revealed the similar result, i.e., acceleration of VC. Of note, in our in vivo interventional study using this NBDpep, atheroma volume itself was suppressed but calcium deposition was accelerated, indicating the complex behavior of NF-κB signaling in atherosclerosis development. This paradoxical contribution of NF-κB on atheroma evokes the clinical evidence of statins, which are known NF-κB inhibitors, on morphologic changes of plaque including regression of plaque volume and lipid composition accompanied by promotion of calcification (50, 51). Although the studies have explored the molecular mechanisms of statins’ procalcific effect (52), a precise understanding is still incomplete (53, 54). Further investigation is needed to elucidate a more detailed understanding of the contribution of NF-κB to VC.

There are several limitations in our study that deserve attention. First, CEA specimens involve a certain selection bias as well as surgical manipulation, and it should be acknowledged that our findings might not be representative of humans with IPH who do not undergo CEA. Second, our in vitro experiments did not consider the effect of erythrocyte membranes, which have been previously shown as a stimulator of VC (13). However, our in vivo data from both humans and mice suggest IPH and inflammatory reaction do not promote VC. Third, the continuous injection of NBDpep in our mouse model likely suppressed the activity of NF-κB signaling in many cell types and systemically, rather than in the VSMCs in the plaque, and thus our data on the anticalcific effect of this TF must be interpreted within that context. Fourth, we cannot rule out other mechanisms regarding CD163 and its anticalcific effect, such as tumor necrosis factor-like weak inducer of apoptosis neutralization (25), that might also contribute to the effect seen on VC. Last, we identified an inverse relationship between a human CD163 polymorphism (i.e., rs7136716) and dense coronary calcification development in African Americans who suddenly died with coronary plaque rupture. However, the relationship between this SNP and calcification has not been shown in larger GWAS, perhaps because the frequency of this SNP is quite low in Caucasians, who are the predominant participant of most GWAS.

In conclusion, our study suggests CD163^+^ M(Hb) macrophages are a unique alternative macrophage subtype found at sites of IPH that inhibit, rather than promote, calcification through activation of the NF-κB pathway in VSMCs. These data provide one reasonable understanding of why rupture-prone vulnerable plaques, which occur as a result of IPH, are not heavily calcified but often demonstrate inflammatory activation.

## Methods

Details of experimental methods are described in Supplemental Methods.

### Data availability.

The raw microarray data files were posted at NCBI’s Gene Expression Omnibus public database (GEO GSE222453).

### Statistics.

Continuous variables with normal distribution are expressed as mean ± SD, or standard error, as appropriate. Variables with skewed distribution were expressed as median and 25th to 75th percentiles. Normality of distribution was tested by the Shapiro-Wilk test. Normally and skewed distributed data were analyzed for significance by Student’s 2-tailed *t* test or Mann-Whitney test, respectively. For multiple-group comparisons, 1-way ANOVA followed by post hoc Tukey’s test or Kruskal-Wallis test followed by post hoc Dunn’s test was applied.

GraphPad Prism (GraphPad Software version 8 or 9) software was used for analysis. *P* < 0.05 was considered statistically significant.

### Study approval.

Studies using deidentified human pathological and autopsy specimens were approved for exempt review by the IRB of CVPath Institute. All human participants provided written informed consent to participate in the study. The IACUC of Medstar Health Research Institute (Washington, DC, USA) approved all animal protocols. All animal experiments were conducted according to the NIH’s *Guide for the Care and Use of Laboratory Animals* (National Academies Press, 2011).

## Author contributions

AS, RK, MM, LG, AC, HA, RK, FDK, RV, and AVF contributed to the analysis of human atherosclerotic specimens. AS, RK, KHP, MM, DTF, WX, RF, AEV, ALK, KH, CMM, RD, and JU performed in vitro experiments involving human macrophages and aortic SMCs. AS, RK, KHP, MM, LG, KK, TK, PJL, and ME performed mouse experiments and analysis. AS, AC, SKBG, and AD performed bioinformatics analysis except for scRNA-Seq of human coronary artery samples. JVM, AWT, and CLM performed scRNA-Seq data analysis for human coronary artery samples. AS, RK, LG, AC, SKBG, HJ, ST, YS, KK, CCW, SK, LMW, NVG, MER, FDK, RV, and AVF participated in scientific discussions. AS, RK, LG, SKBG, RV, and AVF drafted and/or edited the manuscript, which was critically reviewed by all the authors.

## Supplementary Material

Supplemental data

## Figures and Tables

**Table 1:**
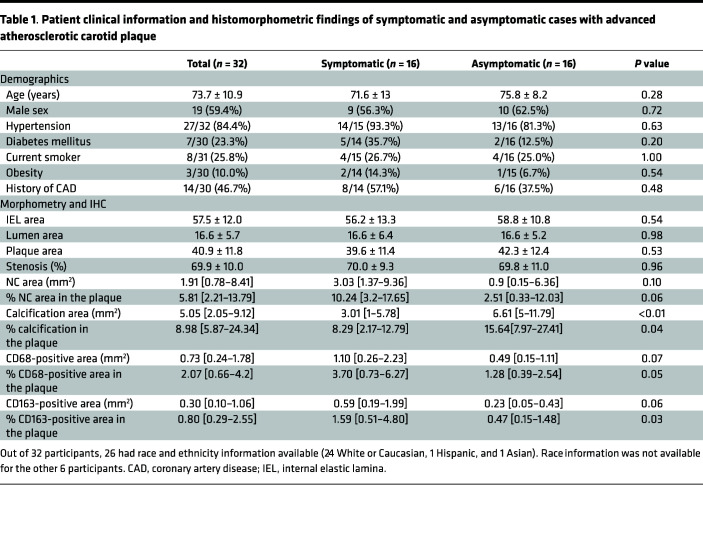
Patient clinical information and histomorphometric findings of symptomatic and asymptomatic cases with advanced atherosclerotic carotid plaque

## Version 1 01/31/2023

In-Press Preview

## Version 2 03/08/2023

Electronic publication
