# Supplementary material for: CD163+ macrophages restrain vascular calcification, promoting the development of high-risk plaque
Source: JCI Insight. 2023 Mar 8;8(5):e154922. doi: 10.1172/jci.insight.154922 (PMC10077470; doi:10.1172/jci.insight.154922)
Supplement: Supplemental data [file jciinsight-8-154922-s163.pdf]

Supplemental Materials for:

CD163[+] Macrophages Restrain Vascular Calcification, Promoting the Development of High-Risk Plaque

Authors: Atsushi Sakamoto[1,a*]; Rika Kawakami[1*]; Masayuki Mori[1,b]; Liang Guo[1,c]; Ka Hyun Paek[1]; Jose Verdezoto Mosquera[2,3]; Anne Cornelissen[1,d]; Saikat Kumar B. Ghosh[1]; Kenji Kawai[1]; Takao Konishi[1]; Raquel Fernandez[1,e]; Daniela Fuller[1,f]; Weili Xu[1]; Aimee Vozenilek[1]; Yu Sato[1,g]; Hiroyuki Jinnouchi[1,h]; Sho Torii[1,g]; Adam W. Turner[2]; Hirokuni Akahori[4]; Salome Kuntz[1,i]; Craig C. Weinkauf[5]; Parker J. Lee[1]; Bob Kutys[1]; Kathryn Harris[6]; Alfred Lawrence Killey[1]; Christina M. Mayhew[1]; Matthew Ellis[1]; Leah M. Weinstein[1]; Neel V. Gadhoke[1]; Roma Dhingra[1]; Jeremy Ullman[1]; Maria E. Romero[1]; Frank D. Kolodgie[1]; Clint Miller[2]; Renu Virmani[1]; Aloke V. Finn[1,6]

Affiliations:
[1]CVPath institute, Inc., Gaithersburg, MD
[2]Department of Public Health Sciences, Center for Public Health Genomics, University of Virginia School of Medicine, Charlottesville, VA
[3]Department of Biochemistry and Molecular Genetics, University of Virginia, Charlottesville, VA
[4] Department of Cardiovascular and Renal Medicine, Hyogo Medical University, Nishinomiya, Hyogo, Japan
[5]Division of Vascular and Endovascular Surgery, University of Arizona, Tucson, AZ
[6]University of Maryland, School of Medicine, Baltimore, MD

Correspondence:
Aloke V. Finn, MD

CVPath Institute, Inc

19 Firstfield Rd. Gaithersburg, MD 20878

Tel: +1-301-208-3570

Email: afinn@cvpath.org

a. Current address: Division of Cardiology, Internal Medicine III, Hamamatsu University School of Medicine, Hamamatsu, Shizuoka, Japan
b. Current address: Department of Cardiovascular Medicine, Kanazawa University Graduate School of Medical Sciences, Kanazawa, Ishikawa, Japan

c. Current address: Bioscience Cardiovascular Research and Early Development, Cardiovascular, Renal, and Metabolism, BioPharmaceuticals Research and Development, AstraZeneca, Gaithersburg, Maryland, USA
d. Current address: Department of Cardiology, Angiology and Internal Intensive Medicine, University Hospital Aachen, RWTH Aachen University, Aachen, Germany
e. Current address: American College of Medical Genetics and Genomics, ACMG Foundation for Genetic and Genomic Medicine, Bethesda, Maryland, USA
f. Current address: Molecular Medicine, University of Maryland, School of Medicine, Baltimore, Maryland, USA
g. Current address: Department of Cardiology, Tokai University School of Medicine, Isehara, Kanagawa, Japan
h. Current address: Division of Cardiovascular Medicine, Saitama Medical Center, Jichi Medical University, Saitama City, Saitama, Japan
i. Current address: Department of Vascular Surgery and Kidney Transplantation, University Hospital of Strasbourg, France

*These authors contributed equally to this work.

Supplemental Methods

Histology of human carotid artery endarterectomy samples

Human carotid plaques removed from patients eligible for CEA were selected from the CVPath Registry (1). Thirty-two human carotid plaques were removed from 32 patients eligible for surgical CEA. The carotid plaque (common and internal carotid artery plaque) was removed in an undisturbed manner and selected for the current study. Indications for surgery were ipsilateral symptoms involving transient ischemic attack or stroke in the previous 6 months (Sym) or significant carotid stenosis (>70%) without ischemia related symptom (Asy) measured by duplex ultrasound. In total, there were 16 Sym cases and 16 Asy cases. At surgery, the carotid tissue was chilled immediately after excision and then promptly frozen at –80°C and shipped to CVPath Institute. The CEA specimen was partially thawed and frozen in Optimal Cutting Temperature (OCT) compound (Miles Inc.). Cryosections were prepared using a Hacker Bright OTF/HS microtome cryostat equipped with a tungsten carbide knife with an installed CryoJane (Leica Microsystems). The CryoJane Tape-Transfer System improves cryosectioning outcomes on glass slides, particularly for the heavily calcified tissues common to carotid plaques (2). The frozen tissue was step sectioned at 10μm thickness, 15 to 20 serial sections for staining were collected on glass slides, and adjacent frozen sections of 1.0 to 1.2mm were collected in plastic tubes and frozen for protein extraction. These steps were repeated until the frozen block was exhausted. This method allowed matching of histological findings with protein analysis of western blotting.

Lesion classification and selection

Lesion characterization of all plaques were determined according to a modified American Heart Association classification as previously reported (3). Three sections from each carotid plaque were selected for morphometric analysis. Lesions with the plaque rupture site (or ulceration) or the most severely narrowed segment were used as the culprit site, and lesions (flanking plaques) located 1.2mm above or below the presenting lesion were also assessed by IHC. Thirty-two specimens containing culprit segments, along with proximal and distal flanking segments, were available for analysis and assessed for plaque morphology. A total of 96 sections (32 culprit, 32 proximal flanking, and 32 distal flanking plaques) were classified, of which 38 were fibroatheromas (including 13 fibroatheroma and 25 fibroatheroma with calcification), 12 were healed plaque ruptures, 12 were fibrocalcific lesions, 10 were plaque ruptures, 4 were TCFAs, 7 were calcified nodules, 6 were PITs, 3 were intimal xanthomas, 2 were chronic total occlusions, and 2 were fissures. From these sections, 70 sections with advanced plaque phenotype were selected for additional analysis, as shown in Figure 1.

Histology of human coronary artery chronic total occlusion samples

Cases with chronic total occlusion (CTO) were obtained from CVPath Institute's sudden coronary death registry. Coronary arteries had been removed from the heart following perfusion fixation in 10% neutral buffered formalin. The major epicardial coronary arteries were radiographed and decalcified in EDTA. The arteries were sequentially cut at 3 mm intervals, labelled, dehydrated, and embedded in paraffin. The right coronary artery (RCA), left anterior descending artery (LAD), and left circumflex artery (LCX) were divided into proximal and mid to distal regions as previously described. Histological definition of CTO was defined as the

occluded lumen occupied by proteoglycan and/or collagen with or without neovascularization and chronic inflammation. A total of 145 sections from 21 CTO lesions (19 patients) were included for the analysis. The CTO sections were divided into three phases by the luminal thrombus organizing time course i.e., 1) organizing thrombus with persistent fibrin, 2) organized with proteoglycan-rich thrombus without fibrin, and 3) organized with type I collagen-rich without fibrin. The borderline of original plaque and thrombotic occluded lumen was determined by 2 expertized cardiovascular pathologists (AS and RK) with H&E and Movat staining sections.

Immunohistochemistry and immunofluorescence staining

Cryosections of human carotid endarterectomy (CEA) samples were stained with H&E, Movat Pentachrome (Movat), Alizarin Red (AR), and von Kossa (VK) stains. Immunohistochemical staining with CD68 (a general macrophage marker) and CD163 (a M[Hb] macrophage marker) were performed, as previously reported (4). Paraffin-embedded human coronary CTO samples were stained by H&E, Movat, and immunohistochemical staining with CD163 and alpha smooth muscle cell actin (αSMA). Six CEA samples each from the plaques with low and high CD163 expression (assessed by the amount of CD163 staining positive area in the adjacent sections) were selected for further western blotting analysis (see below).

Immunofluorescence staining for CD163 and hyaluronan synthase 1 (HAS1)/hyaluronan was carried out on formalin-fixed paraffin-embedded human coronary sections. The frozen sections were fixed in cold acetone for 10 minutes, with further exposure to 0.15% H_2O_2 for 20 minutes. The sections were treated with Dako protein block (catalog X0909) for 10 minutes before incubation with primary antibodies against CD163 (Santa Cruz Biotechnology, catalog sc-20066, clone GHI/61, dilution 1:200, overnight at 4°C); CD68 (Agilent (Dako), clone KP1,

dilution 1:800, 1 hour). Antibody detection included biotinylated goat anti-rabbit, horse anti-mouse, and rabbit anti-goat (Vector Laboratories, BA-1000, BA-2000, BA-5000, respectively, at 1:200 dilution) and Alexa Flour 488 and 555 streptavidin (Invitrogen, Thermo Fisher Scientific, S32354 and S32355, respectively, dilution 1:100). The positive and negative controls (sections stained with a secondary antibody only or without any antibody to detect autofluorescence) were included in every staining procedure. The sections were counterstained with DAPI (Invitrogen, Thermo Fisher Scientific, catalog D3571). Images were captured by laser-scanning confocal microscopy (Carl Zeiss, LSM 700, 800, or 880) using ×20 or ×40 objectives with optical slicing in the z axis. All quantification of immunostaining area was conducted using Zen software (Carl Zeiss) or the HALO platform (Indica Labs).

Mice

The Institutional Animal Care and Use Committee at the MedStar Health Research Institute approved all animal protocols. All animal experiments were conducted according to the National Institutes of Health Guide for the Care and Use of Laboratory Animals. Both male and female littermates were used in this study.

CD163 knockout mice ($CD163^{tm1(KOMP)Vlcg}$) were generated using targeting constructs available from the University of California at Davis International Mouse Consortium (KOMP) as previously described (5). Mice with genetic deficiency of Apolipoprotein E (on a C57BL/6J) were purchased from the Jackson Laboratory. $ApoE^{-/-}$ mated to $CD163^{-/-}$ mice until homozygosity was achieved for both genes (i.e. $ApoE^{-/-}CD163^{-/-}$). These mice were compared to littermate control $ApoE^{-/-}CD163^{+/+}$ mice. All animals in the study of $ApoE^{-/-}$ (i.e., $ApoE^{-/-}$

CD163$^{+/+}$) versus *ApoE*$^{-/-}$*CD163*$^{-/-}$ mice were maintained on regular chow diet for 1.5-years to the end of experiment.

For the interventional experiment for NFκB signaling, *ApoE*$^{-/-}$ mice were randomly assigned to two groups: mice subjected to administration of IKK-NBD control peptide (CTLpep) or NFκB inhibitor NBD peptide (NBDpep) (n=12 per group, both 100μg $kg^{-1}day^{-1}$, Enzo Life Sciences). High fat diet (HFD) feeding (21.0% fat and 0.2% cholesterol) was started at the age of 8-weeks and continued to the end of experiment, and then received either CTLpep or NBDpep via subcutaneously implanted ALZET Osmotic Pumps (Model 2006, DURECT Corporation, Cupertino, CA, USA) for 18 weeks (by the age of 32-weeks) (Figure 8A). Osmotic pumps were surgically replaced every 6 weeks (a total of 3 surgeries per mouse were required) due to the lifetime of the pump. At the end of the experiment, mice were sacrificed and perfused by saline via apical heart injection. BCA was harvested and embedded in OCT compound and frozen. After BCA collection, whole thoracic aorta (half of the mice in each group) or whole plaque samples (other half of mice in each group) peeled out from whole thoracic aorta (Figure 7H) were obtained from CTLpep- or NBDpep-loaded mice for further experiments.

Mouse histology and immunofluorescent staining

OCT compound embedded frozen tissue samples were sectioned and embedded on slides at 8 μm thickness. Slides were stained by H&E, Movat pentachrome and Alizarin Red for histological analysis. For immunofluorescence, sections were incubated in primary antibody for p65 (R&D AF5078) at a dilution of 1:50 for 16h at 4 °C. Regarding runt-related transcription factor 2 (RUNX2) staining, following methanol permeabilization, sections were stained with primary antibody for RUNX2 (1:8000, CST, #12556) for 16h at 4 °C. Alexa Fluor labeled

secondary antibody (Life Technologies) were used for further incubation. Spleen sections from wild type mice were used as positive controls (in both anti-p65 and -RUNX2 stains). Tissue slides without incubation by primary antibody were used as negative controls. DAPI was used as nuclear counterstaining. Tissue samples were mounted on slides with Vectashield antifade mounting medium (Vector Labs, Burlingame, CA). Tissue was visualized using objective on a LSM 700, 800, or 880 laser scanning confocal microscopy (Zeiss). Projection images were generated by collecting the maximum pixel intensity from each image of the Z stack and by projecting pixel intensity onto the single (projection) image.

Cell culture and reagents

Human Peripheral blood mononuclear cells (PBMC) collected from healthy volunteers (Astarte Biologics, Redmond, WA, or BioIVT, Westbury, NY) were seeded into 6-well culture plates at a density of 3-4 x 10^6 cell/well. After 6 hours incubation, non-attached cells were removed, and culture media was replaced. Remaining monocyte/macrophage fraction were differentiated over 6 days into macrophages with RPMI 1640 medium (Gibco, Invitrogen) supplemented with 10% human serum (Sigma Aldrich) and penicillin-streptomycin (the final concentrations were 100 units/mL of penicillin and 100 μg/mL of streptomycin, Gibco) in humidified air (5% of CO_2) at 37 °C. Prior to use, hemoglobin (lyophilized stabilized purified A0 ferrous hemoglobin [Sigma Aldrich]) and haptoglobin phenotype 1-1 (lyophilized purified [Sigma Aldrich]) were reconstituted in sterile phosphate buffered solution (PBS) at a concentration of 0.1 mg/ml. Equimolar amounts of hemoglobin and haptoglobin in growth medium were used to generate hemoglobin:haptoglobin complexes (HH), which were added to cultured monocytes at 6 hours after plating. We differentiated human monocytes over 6-days

with or without HH which reproduced the phenotype seen in human atherosclerotic plaques at areas of hemorrhage, as previously described (4). Supernatant from control macrophages [M(con)] and HH-differentiated [M(Hb)] macrophages were used in further experiments.

Primary human aortic smooth muscle cells (HASMCs, Cell applications or ATCC) were cultured, up to passage 10, using the smooth muscle cell growth medium (Cell applications) or vascular cell basal medium (ATCC) supplemented with vascular smooth muscle cell growth kit (ATCC) and penicillin-streptomycin-Amphotericin B Solution (ATCC) in humidified air (5% of CO_2) at 37 °C. For the induction of calcification in vitro, HASMCs were cultivated in M(con), M(Hb) macrophage supernatant, or RPMI 1640 medium-based macrophage control media with or without HH (see above) supplemented by $CaCl_2$ (4 mM; Sigma), β-glycerophosphate (5 mM; Sigma), l-ascorbic acid (50 µg/mL; Sigma), insulin (1 µM; Sigma), and dexamethasone (0.1 µM; Sigma) (osteogenic media components; OS) (6). Hyaluronan and 4-methylumbelliferone were purchased from R&D systems and Sigma-Aldrich, respectively.

For mice peritoneal macrophage experiments, peritoneal macrophages were extracted and cultured as previously described (7). Age and gender-matched wild type (WT) and *CD163*$^{-/-}$ mice were applied. Macrophages were cultured with RPMI 1640 media containing 10% human serum with or without 0.1mg/ml mice hemoglobin (324-30, Lee BIOSOLUTIONS, Maryland heights, MO., LS-G11199-5, LifeSpan Bioscience, Seattle, WA). Supernatant was collected after 24 hours exposure. HASMCs were cultured with mouse peritoneal macrophage with or without OS (identical components described above) for 24 hours.

HASMC transfection

The small interfering RNA (siRNA) target sequences for the human HAS1 and p65 genes were purchased from the Santa-Cruz (sense: 5’-GGGAGGGUAUUUAUUGGUCtt-3’, antisense: 5’-GACCAAUAAAUACCCUCCCtt-3’) and Ambion (Assay ID: s11914), respectively. The scrambled control siRNA purchased from Thermo Fisher Scientific and Ambion were also used for the experiments. The siRNAs were transfected into the HASMC, employing Lipofectamine RNAiMAX reagent (Life Technologies Corporation), and the siRNA-expressing HASMC were used for the experiment 48-72 hours after transfection. Knockdown of targets were validated by western blot.

Alizarin Red staining of cultured HASMC

AR staining was used to determine the calcification. Cells were washed once with calcium and magnesium free PBS (Thermo Fisher) and fixed with 4% formaldehyde for 10 minutes at room temperature. After washing with PBS twice, cells were exposed with 2% Alizarin Red S solution (pH 4.2) for 15 minutes and then washed 5 times with dH_2O and observed under optical microscope.

Quantification of calcium deposition

Calcium deposits in HASMCs were quantified, as described previously (8). Cells were washed with PBS and treated with HCl (0.6M) for 24 hours at 4 °C and the supernatant was analyzed with a colorimetric quantification calcium assay kit (QuantiChrom^{TM}, BioAssay Systems, Hayward, CA). Protein was extracted from remaining cell components with 0.1M NaOH + 0.1% SDS, and measured calcium levels were normalized by protein concentration. For

separated mice aorta in in vivo experiments, samples were minced and treated with 0.6M HCl (24 hours at 4 °C). The supernatant was also analyzed by the QuantiChrom™ calcium assay.

RNA isolation, cDNA synthesis, and quantitative real-time polymerase chain reaction

Total RNA was extracted from monocyte/macrophage or HASMC using RNeasy Mini or RNeasy Plus Mini Kit (Qiagen, Valencia, CA) in accordance with manufacturer's instruction. Quantitation and quality assessment of the RNA were assessed by using Thermo NanoDrop 2000 (Thermo Fisher). RNA was converted to cDNA using High-Capacity cDNA Reverse Transcription Kit (Applied Biosystems). Quantitative real-time polymerase chain reaction (RT-PCR) analysis was performed by QuantStudio 3 (Thermo Fisher). Primer sequences for SYBR green RT-PCR are shown in Supplemental Table 4. Values of the cycle threshold (Ct), obtained for quantification, were used for calculations of fold-change in mRNA abundance following the $2^{-\Delta\Delta Ct}$ method. GAPDH was chosen as the housekeeping gene.

Microarray

RNA samples extracted from HASMC were used for global gene expression profiling on human Clariom S™ Assay microarrays (Thermo Fisher). Gene expression was determined in 4 RNA samples from each group. Sample preparation, microarray hybridization, scanning, and quality control were carried out at the Genomic Core Facility, Translational Genomic Laboratory, The University of Maryland School of Medicine.

Raw signal intensities were normalized using Transcriptome Analysis Console (TAC) 4.0 software (Applied Biosystems, Thermo Fisher Scientific). The RStudio (v1.2.5019) and GraphPad Prism (GraphPad Software version 8 or 9, La Jolla, CA, USA) software were used to

create the heat map, and TAC 4.0 software was used to create the volcano plot for the gene expression data. For functional annotation of Gene Ontology biological process (GO-BP) enrichment, the online software Database for Annotation, Visualization, and Integrated Discovery (DAVID) version 6.8 was applied. The raw data of Affymetrix CEL files were posted at NCBI's Gene Expression Omnibus (GEO) public database (GSE222453).

ELISA

In select experiments, supernatants from cultured macrophages or HASMC, as well as mice aortic plaque samples, were analyzed for the hyaluronan concentration using ELISA (R&D Systems) in accordance with the manufacturer's instruction. Absorbance was measured by a FLUOstar OPTIMA Microplate Reader (BMG labteck, Ortenberg, Germany) or by a Varioskan™ LUX multimode microplate reader (Thermo Fisher).

Western blotting

Whole-cell lysates of HASMC, mice plaque samples, and frozen human CEA samples were prepared by resuspending the samples in ice-cold RIPA lysis buffer (CST) supplemented with protease inhibitors (Thermo Fisher) and phosphatase inhibitors (Thermo Fisher). Protein from the nuclear fraction was extracted utilizing NE-PER™ nuclear and cytoplasmic extraction reagents (Thermo Fisher). After sonication, lysates were then clarified by centrifugation at 14000rpm, 4 °C, for 15 min. Protein concentrations were estimated using the BCA Protein Assay Kit (Thermo Fisher). Equal amounts of protein extract (5-20 ug) were subjected to SDS-polyacrylamide gel (BioRad) electrophoresis and electrotransferred onto Nitrocellulose membranes (Invitrogen). Blocking was performed using BLOK™ Casein in 1% TBS (G-BIOSCIENCES) or Blocker™

Casein in TBS (Thermo Fisher) for 1 hour at room temperature. Membranes were then probed with primary antibodies diluted in the block solution, followed by detection using horseradish peroxidase conjugated secondary antibodies (BioRad) and enhanced chemiluminescence substrates (BioRad). Antibodies used were rabbit anti-RUNX2 (1:1000, CST, #12556), rabbit anti-NFκB p65 (1:2000, CST, #4764), rabbit anti-phospho-NFκB p65 (Ser536) (1:5000, CST, #3033), mouse anti-HAS1 (1:1000, Thermo, MA5-15671), rabbit anti-HAS1 (1:1000, Thermo, PA5-50674), mouse anti-HAS2 (1:1000, Abcam, ab140671), rabbit anti-HAS2 (1:1000, LSBio, LSC411405), mouse anti-HAS3 (1:1000, Thermo, MA5-17088), rabbit anti-HAS3 (1:1000, Thermo, PA5-89266), rabbit anti-CD44 (1:1000, CST, #37259), mouse anti-CD163 (1:1000, Thermo, 15227267), and mouse anti-β-actin (1:2000, Abcam, ab8226) for normalization.

PrestoBlue HS cell viability assays and Terminal deoxynucleotidyl transferase dUTP nick end labeling (TUNEL) assay

HASMCs viability after exposure to M(con) or M(Hb) macrophage supernatants was assessed by PrestoBlue HS cell viability dye (Thermo Fischer, P50200) in accordance with manufacture's instruction. Briefly, cells were cultured on 96-well plates until grown to be confluent. Media was replaced to M(con) or M(Hb) macrophage supernatant and incubated for 24 hours. PrestoBlue HS cell viability dye was added at a 1:10 volume ratio. PrestoBlue HS dye was also incubated with cell-free, media only controls, negative control with normal growth media, and positive control (10 min exposure to growth medium containing H_2O_2, 200mM). PrestoBlue HS fluorescence signal was quantified using a Varioskan™ LUX multimode microplate reader (Thermo Fisher). Resulting signal with background correction was reported as a ratio of 560/590 nm fluorescence. For TUNEL assay, HASMCs were cultured on 35mm glass bottom dishes

(MatTek Corporation, MA). After exposing to M(con) or M(Hb) macrophage supernatant, cells were fixed by 4% formaldehyde, and TUNEL staining by CFTM Dye TUNEL Assay Apoptosis Detection Kit (#30063, Biotium, Dremont, CA) were performed under manufacture's instruction. Hoechst 33342 (62249, Thermo Scientific) was used for nuclei counter staining. Images were obtained by laser-scanning confocal microscopy (LSM800, Carl Zeiss) using ×20 or ×40 or x63 objectives with optical slicing in the z axis.

Human coronary plaque and CD163 SNPs

SNPs from the CD163 gene were examined in 346 subjects from the CVPath Registry by Illumina Exome BeadChip array, as previously described (9). For further analysis of rs7136716, there were a total of 111 subjects with homozygous major AA alleles, 175 with heterozygous AG alleles, and 60 with homozygous minor GG alleles. Thirty age-matched victims who died due to severe CAD (15 cases each of AA and GG genotype carriers) who have available post-mortem *ex vivo* heart X-ray were selected for further assessment. According to the X-ray, coronary arteries were divided into 10 segments (i.e., left main trunk, and proximal, middle, and distal of each RCA, LAD, and LCX). The semi-quantitative calcification score of each segment was evaluated in 0 to 10 scales by 2 independent cardiovascular pathologists (LG and AS) with blinded manner for rs7136716 genotype. Total amount of calcium score in 10 segments were considered as the score of each case. For histological assessment, 3 sections with most stenotic lesion were selected from RCA, LAD, and LCX proximal site (i.e. 9 sections/case). Each section was apart at least 3mm of longitudinal distance. Morphometric data was averaged in 3 sections in each branch. Percent area stenosis (mean) and (max) represent average of 3 vessels and the value of the most stenotic branch, respectively. Calcification area (mean) and (total) represents average and total of calcification in

3 vessels. Percent calcification/plaque (mean) and (max) are average of 3 vessels and the maximum value in the 3 vessels. Other definitions of values related to necrotic core were the same as those for calcification.

Single cell RNA sequence (scRNA-seq) data processing for human coronary artery samples

We used scRNA-seq data from prior study by Wirka et al. (10) (GEO accession GSE131780). In addition to quality control (QC) metrics used by Wirka et al, we included coverage as an additional metric when processing data with the Seurat package (v.4.1.0) (11). Genes expressed in less than 5 cells were filtered out. Cells expressing < 500 and > 2500 genes, and with < 2000 UMIs were also trimmed from the dataset to prune defective cells, multiplets, or cells with low coverage. Cells with 5% of reads mapping to mitochondrial genes were also discarded. After removing lower-quality cells, 7209 high-quality cells remained for subsequent analyses. Read counts were normalized using Seurat's global-scaling method. After finding the 2,000 most variable genes in the data, dimensionality reduction was performed using principal component analysis (PCA). The top ten principal components (PCs) were further used for uniform manifold approximation and projection (UMAP) visualization and Louvain clustering. Clusters were annotated using gene lists provided by Wirka et al (10).

Single-nucleus assay for transposase-accessible chromatin with sequencing (snATAC-seq) data processing

FASTQ files for the snATAC dataset were generated as detailed in Turner et al (12) (GEO accession GSE175621). Briefly, FASTQ files were preprocessed using the 10x Genomics Cellranger pipeline (CellRanger ATAC v1.2.0) using the hg38 reference genome. Samples were

preprocessed separately and cellranger outputs were used to filter low-quality cells with the ArchR pipeline (v.1.0.2) (13) as follows: TSS enrichment > 7, unique number of fragments > 10000 and a doublet ratio < 1.5. Fragment files for the 41 patients were used to generate ArchR arrow files. We used the first 30 components output by LSI dimensionality reduction for UMAP analyses. Cell clustering was then performed using the Louvain community detection algorithm as implemented in Seurat (v.4.1.0). Gene scores were then estimated based on the accessibility within gene bodies as well as distal/proximal regions to the TSS.

snATAC-scRNA integration

The cell-type annotated scRNA-seq expression matrix was integrated with the snATAC-seq gene score matrix using the 'addGeneIntegrationMatrix' function from ArchR, which identifies corresponding cells across datasets or 'anchors' using Seurat's mutual nearest neighbors algorithm. Cell-type labels within the Seurat scRNA-seq metadata were transferred to the corresponding mutual nearest neighbors in the snATAC-seq data along with their gene expression signatures. This resulted in snATAC-seq cells having both a chromatin accessibility and a gene expression profile.

Stratification of patients and Differential Expression (DE) analyses

Using the scRNA-seq-snATAC data, we extracted macrophages and calculated the mean CD163 expression for this cell type across each of the 41 patients in the integrated scRNA-seq-snATAC integrated dataset as described above. We then calculated the median and interquartile ranges (IQR) using the stats R package (R core team, 2020). Individuals below the CD163 mean macrophage expression distribution 25% quartile were denoted as low CD163 (n=11, norm

expression<3.87), whereas those above the distribution 75% quartile were denoted as high CD163 (n=11, norm expression>5.33). We then extracted and aggregated SMC count matrices from individuals in each of those two groups, annotated SMCs as high CD163 or low CD163, and merged matrices for downstream analyses. We used this merged matrix in order to perform an unpaired T-test-based differential expression (DE) analysis between SMCs from the two groups with the following parameters (var.equal=TRUE). We kept genes that had an FDR < 0.05. Genes were then ranked according to the difference in the mean expression value between the two groups.

Gene set enrichment and Transcription Factor (TF) over-representation analyses

Genes from the above DE analysis were ranked according to the difference in mean expression across the two groups. We then created gene sets with the top 100 genes upregulated in high and low CD163 SMCs. We used these gene sets as input for gene set enrichment analyses (GSEA) with the Enrichr web tool (14). GSEA was carried out across multiple ontologies (Jensen tissues, Mouse gene atlas, and Panglaodb). Finally, these gene sets were used as input for TF-target over-representation analyses with the ChEA3 web tool (v.3.0) (15). To identify TFs associated with the gene sets of interest, we performed this analysis using the ChEA3 TF-target gene set libraries assembled from ENCODE ChiP-seq data. Briefly, the Fisher's Exact Test, with a background size of 20000, was used to compare input gene sets of interest to the ENCODE-based TF-target gene set library available in ChEA3. This allowed us to determine which TFs may be most closely associated with gene sets that were upregulated in high/low CD163 groups.

Supplemental Figure legends

Supplemental Figure 1. Outline of analysis for the amount of calcification in the spatial area of CD163$^+$/CD68$^+$ and CD163$^-$/CD68$^+$ in human atheroma of carotid artery (frozen CEA specimens)

Surgically removed carotid artery pathologic sections from 62-yo male patient. **A**: Low-power H&E image of fibroatheroma. **B**-**F**: High-power image of H&E (**B**), VK (**C**), CD163 (**D**) and CD68 (**E**) immunostaining, and AR (**F**) images of the corresponding black rectangular field in Image A. To determine the spatial distribution of CD163, CD68, and calcification, CD163 positive areas were digitally traced (bordered by blue line [**D**]) in CD163 IHC images (1st step). The CD163 positive area was digitally overlaid on an adjacent CD68 IHC image (2nd step). Subsequently, CD68 positive but CD163 negative area was digitally traced (bordered by red line [**E**]) in CD68 IHC image (3rd step). Both blue border (CD163$^+$/CD68$^+$) and red (CD163$^-$/CD68$^+$) border areas were digitally overlaid on an adjacent AR image (4th step). Finally, AR positive areas in blue and red zones were determined by HALO digital software (5th step). Total 70 advanced atheroma sections obtained from 32 patients were applied for this analysis. AR=Alizarin Red, CEA=carotid endarterectomy, IHC=immunohistochemistry, NC=necrotic core, VK=Von Kossa.

Supplemental Figure 2. Time dependent effect of OS media on HASMC calcification in vitro

A-**B**: HASMC were seeded in 24-well culture plates, grown to confluence, and cultivated for 6, 24, and 48 hours. Representative image of AR staining of HASMC (**A**) and summary of % AR

positive areas for each time point after exposure to culture media with OS is shown (**B**) (n=3 in each).

C: Summary of calcium amount in samples of each time point examined by colorimetric assay adjusted for protein content (n=4 in each).

D-**E**: Representative western blot images of RUNX2 and β-actin from protein samples extracted at each time point (**D**). Summary of densitometry analysis is shown in **E** (n=4 in each). ** $p<0.01$ vs 0h.

Results are presented as the mean±standard error and ANOVA followed by post-hoc Tukey's test was conducted for statistical analysis (**B**-**C**, **E**). Data normality was tested by Shapiro-Wilk test. All experiments were performed at least three times to confirm the reproducibility.

AR=Alizarin Red, HASMC=human aortic smooth muscle cell, OS=osteogenic components supplementation, RUNX2=runt-related transcription factor 2.

Supplemental Figure 3. Validation results of RT-PCR in RNA samples extracted from M(con) and M(Hb) plus OS exposed HASMC

HASMCs were treated with M(con) or M(Hb) + OS supernatant for 24 h. Real-time RT–PCR analysis was performed in order to validate the result of microarray analysis (Figure 4) including the expression levels of *NFKIA*, *NFKB1*, *RELA*, *RUNX2*, *BMP4*, *MGP*, *HAS1*, *HAS2*, *HAS3*. Data were normalized with GAPDH expression. Results are presented as the mean±standard error and t-test was conducted for statistical analysis (n=3 per group). Data normality was tested by Shapiro-Wilk test. All experiments were performed at least three times to confirm the reproducibility.

M(con)sup=control macrophage supernatant, M(Hb)sup=HH-differentiated macrophage supernatant, OS=osteogenic components supplementation, RQ=relative quantification.

Supplemental Figure 4. The effect of M(con) and M(Hb) supernatant on the cell apoptosis and viability of HASMC

A-**G**: Representative fluorescent microscopic images of terminal deoxynucleotidyl transferase-mediated dUTP nick end-labeling (TUNEL) staining of HASMC cultured with normal growth media (negative control; NC) with or without OS (**A**; OS(-), **B**; OS(+)), M(con) with or without OS (**C**; OS(-), **D**; OS(+)), or M(Hb) supernatant with or without OS (**E**; OS(-), **F**; OS(+)) for 48 hours. For positive control (PC), HASMCs were exposed to culture media containing 2mM of H_2O_2 for 8 hours (**G**). DNA fragmentation in apoptotic cells were detected by fluorescein-12-dUTP (green). Nucleus were counterstained by DAPI (blue). Small inset on upper-right in **C** and **E** represent higher magnification of TUNEL positive cells in the white rectangular field of each group. Scale bars indicate 50μm in low power or 10μm in high power fields. **H**: Summarized data of TUNEL positive cells. Bars indicate % of TUNEL positive cells in each condition. The values represent the mean±standard error from 3 independent experiments, which include 3 different fields involving >100 cells in each (overall n=9 per group). **I**: Summarized data from PrestoBlue HS cell viability assay (n=5 per group) in the same experimental condition as TUNEL assay. The experiment was performed three times to confirm the reproducibility. The values represent the mean±standard error. ANOVA followed by post-hoc Tukey's test was applied. Data normality was tested by Shapiro-Wilk test. NC=negative control, PC=positive control, TUNEL=terminal

deoxynucleotidyl transferase-mediated dUTP nick end-labeling, Others are as Supplemental Figure 3.

Supplemental Figure 5. Augmented hyaluronan synthesis in HASMC by M(Hb) supernatant exposure is attenuated by VEGF receptor antagonists

A-**B**: Summary of HA concentration in M(con) and M(Hb) supernatant post-exposing (24 hours) to HASMC (ELISA, n=3 per group) with VEGF receptor antagonist, Axitinib (**A**) or Cabozantinib (**B**), in different dose setting (0, 10, and 100nM). **P<0.01. Results are presented as the mean±standard error. ANOVA followed by post-hoc Tukey's test was applied. Data normality was tested by Shapiro-Wilk test. All experiments were performed at least three times to confirm the reproducibility. HA=hyaluronan, M(con)=control macrophage supernatant, M(Hb)=hemoglobin-haptoglobin complex-differentiated macrophage supernatant.

Supplemental Figure 6. Effect of peritoneal macrophage supernatant obtained from WT or *CD163*$^{-/-}$ mice on HASMC NFκB signaling and HA synthesis

Peritoneal macrophages are collected from WT or *CD163*$^{-/-}$ mice and cultured with or without mouse hemoglobin for 24 hours. The supernatant was exposed to HASMC for 24 hours. **A**-**D**: Immunoblotting of HASMC with quantitation of densitometry for phospho-p65 (Ser 536)/total-p65 (**A**), HAS1/β-actin (**B**), HAS2/β-actin (**C**), and HAS3/β-actin (**D**) in HASMC exposed to mouse peritoneal macrophage supernatant for 24 hours (1: WT M(con), 2: WT M(Hb), 3: *CD163*$^{-}$

$^{-/-}$ M(con), and 4: *CD163*$^{-/-}$ M(Hb)) (n=4 per group). **E**: Summary of HA concentration in WT M(con), WT M(Hb), CD163$^{-/-}$ M(con), and *CD163*$^{-/-}$ M(Hb) supernatant exposed (24 hours) to HASMC (ELISA, n=4 per group). **F**-**H**: Result of real-time PCR analysis of HASMC which exposed to peritoneal macrophage supernatant for 24 hours, including expression levels of *HAS1* (**F**), *HAS2* (**G**), and *HAS3* (**H**). Data were normalized with *GAPDH* expression and presented as mean±standard error (n=3 per group). Results are presented as the mean±standard error and ANOVA followed by post-hoc Tukey's test was applied for statistical analysis. Data normality was tested by Shapiro-Wilk test. All experiments were performed at least three times to confirm the reproducibility.

CD163$^{-/-}$ M(con)=*CD163*$^{-/-}$ macrophage control supernatant, *CD163*$^{-/-}$ M(Hb)=Hb-stimulated CD163$^{-/-}$ macrophage supernatant, FC=fold changes, RQ=relative quantification, WT=wild type, WT M(con)=WT macrophage control supernatant, WT M(Hb)=Hb-stimulated WT macrophage supernatant

Supplemental Figure 7. Representative radiographs of autopsy hearts of sudden cardiac death cases of African American victims with major AA or minor GG genotype of rs7136716 SNP

Radiographs of **A**-**F** are from carriers of major AA genotype and **G**-**L** are from carriers of minor GG genotype of rs7136716 SNPs.

A: A heart with severe CAD from 41-year-old male without any cardiac risk factors. **B**: A heart with severe CAD from 61-year-old female with history of hypertension, diabetes, and CKD. **C**: A heart with severe CAD from 50-year-old female with history of hypertension. **D**: A heart with

severe CAD from 78-year-old male with history of abdominal aortic aneurysm. **E**: A heart with severe CAD from 41-year-old male with history of OMI, hypertension, and diabetes. **F**: A heart with severe CAD from 47-year-old male with history of OMI. **G**: A heart with severe CAD from 44-year-old male with history of drug use without other cardiac risk factors. **H**: A heart with severe CAD from 62-year-old male with history of hypertension and valvular heart disease. **I**: A heart with severe CAD from 48-year-old male without known cardiac risk factors. **J**: A heart with severe CAD from 74-year-old male with history of diabetes. **K**: A heart with recent MI from 47-year-old female with history of hypertension and smoking. **L**: A heart with severe CAD from 46-year-old male with history of OMI.

White arrowheads indicate visible coronary calcification on X-ray. CAD=coronary artery disease, CKD=chronic kidney disease, OMI=old myocardial infarction.

Supplemental Tables

Supplemental Table 1. Clinical information and pathologic findings from coronary CTO cases

**Patient, n**	**n=19**
Age, years	57.5±15.5
Male sex, n (%)	16 (84.2%)
Hypertension, n (%)	10/15 (66.7%)
Diabetes, n (%)	7/15 (46.7%)
Dyslipidemia, n (%)	2/15 (13.3%)
Previous MI, n (%)	3/15 (20.0%)
Cause of death	
Coronary/non-cardiac death	17 (89.5%)
Pathologic healed myocardial infarction	16 (84.2%)
**Vessels, n**	**n=21**
LAD/LCX/RCA	2 (9.5%)/3 (14.3%)/16 (76.2%)
**Section, n**	**n=145**
Thrombus organizing stage	
organizing thrombus (fibrin rich)	23 (15.9%)
Organized thrombus (PGs-rich)	31 (21.4%)
Organized thrombus (type I collagen-rich)	91 (62.8%)

CTO=chronic total occlusion, LAD=left anterior descending artery, LCX=left circumflex, MI=myocardial infarction, PGs=proteoglycans, RCA=right coronary artery

Supplemental Table 2. Differentially Expressed Genes in HASMC exposed to M(Hb) or M(con) supernatant with OS

Gene Symbol	M(Hb)sup +OS mean signal (log2)	M(con)sup +OS mean signal (log2)	Fold Change	P value		Gene Symbol	M(Hb)sup +OS mean signal (log2)	M(con)sup +OS mean signal (log2)	Fold Change	P value
CXCL10	17.83	4.27	12075.77	5.84E-09		*IL1A*	12.03	7.08	31.00	7.97E-07
CXCL9	16.52	4.38	4533.12	3.12E-08		*SAMD9L*	15.05	10.12	30.52	6.33E-06
CXCL11	15.28	5.00	1237.54	1.61E-07		*ICAM1*	16.80	11.90	29.87	1.31E-05
IDO1	15.09	5.15	984.82	2.53E-08		*APOL3*	12.95	8.08	29.33	1.55E-06
GBP4	15.39	5.89	722.01	3.60E-09		*IFIH1*	10.18	5.33	28.82	2.35E-05
IRF1	15.14	7.28	233.24	1.68E-07		*BDKRB1*	14.97	10.19	27.59	1.02E-03
GBP2	14.28	6.43	230.28	5.01E-08		*IL7R*	12.46	7.69	27.38	3.68E-05
XIRP1	13.04	5.23	223.93	1.34E-09		*RNF19B*	13.69	8.94	26.85	8.79E-09
IFIT3	16.07	8.42	201.07	2.39E-08		*OAS1*	10.96	6.31	25.09	2.63E-03
IL18BP	14.25	7.15	137.41	2.78E-09		*BIRC3*	10.03	5.58	21.87	3.95E-04
CX3CL1	12.69	5.78	120.03	1.46E-03		*APOL2*	15.40	10.95	21.80	3.32E-07
GBP1	15.47	8.65	112.69	3.25E-08		*IFIT2*	10.00	5.56	21.73	9.99E-06
CSF3	16.14	9.34	111.30	3.46E-03		*SLC7A2*	14.34	9.92	21.41	1.43E-04
GBP5	11.55	5.02	92.01	1.10E-06		*ADAMTS4*	13.22	8.99	18.86	3.63E-05
CIITA	11.81	5.34	88.87	2.14E-07		*RSPO3*	9.15	4.95	18.32	2.72E-05
CD274	12.49	6.04	87.14	5.19E-08		*VCAM1*	12.44	8.25	18.28	1.28E-02
SLAMF8	10.80	4.40	84.17	7.64E-07		*COL27A1*	10.27	6.09	18.14	2.14E-05
CD38	10.36	4.08	78.05	2.28E-05		*RARRES3*	10.63	6.48	17.76	4.04E-06
CCL8	11.44	5.27	72.38	5.79E-04		*IFI44L*	9.57	5.45	17.38	2.85E-04
HAPLN3	11.98	5.86	69.55	2.29E-07		*ATF3*	10.84	6.76	16.84	9.66E-06
IL32	14.14	8.05	68.32	3.88E-07		*ZC3H12A; MIR6732*	14.99	10.99	15.99	1.37E-04
TNFAIP2	15.33	9.34	63.48	3.46E-05		*PRDM1*	13.50	9.56	15.40	8.88E-04
TAP1	13.74	7.76	63.40	1.17E-06		*TNFAIP6*	15.24	11.33	15.10	2.62E-03
OAS2	10.89	4.98	60.07	8.22E-06		*EPSTI1*	9.30	5.40	14.96	9.35E-06
MX1	12.88	7.11	54.78	9.70E-05		*TNFAIP8*	10.78	6.89	14.87	2.85E-03
GCH1	12.34	6.58	53.92	1.65E-05		*OAS3*	12.58	8.69	14.81	8.67E-04
CTSS	12.47	6.77	52.02	4.27E-07		*PSTPIP2*	9.83	5.94	14.80	1.93E-04
SECTM1	11.16	5.51	50.19	2.21E-05		*LRRTM2*	8.38	4.49	14.77	9.85E-06
LIF	12.22	6.73	44.74	1.06E-03		*RIPK2*	15.53	11.65	14.76	8.12E-05
PARP14	15.14	9.75	42.11	1.54E-06		*TLR2*	9.93	6.17	13.51	1.92E-03
UBD	9.61	4.28	40.39	6.70E-05		*LAP3*	15.26	11.54	13.16	4.18E-07
CCL20	16.15	10.99	35.64	1.23E-02		*TAP2*	12.53	8.86	12.72	4.40E-06
XAF1	11.09	6.10	31.93	1.49E-05		*DDX58*	11.48	7.84	12.48	4.27E-05

Gene Symbol	M(Hb)sup +OS mean signal (log2)	M(con)sup +OS mean signal (log2)	Fold Change	P value		Gene Symbol	M(Hb)sup +OS mean signal (log2)	M(con)sup +OS mean signal (log2)	Fold Change	P value
ETV7	9.20	5.56	12.45	2.00E-05		*ADAMTS9*	13.62	10.76	7.28	2.71E-03
TIFA	11.04	7.41	12.36	2.03E-04		*RARRES1*	11.13	8.27	7.26	2.56E-03
APOL6	11.24	7.62	12.28	3.28E-07		*SAA2; SAA4*	8.48	5.63	7.21	4.31E-02
APOL1	11.97	8.39	11.92	3.44E-06		*MMP25-AS1*	8.55	5.71	7.14	2.72E-08
DTX3L	12.98	9.45	11.55	1.02E-06		*SUSD6*	12.39	9.56	7.14	3.31E-04
PARP9	12.22	8.69	11.48	5.06E-06		*KLF5*	10.87	8.05	7.07	2.76E-04
STEAP4	8.61	5.10	11.36	2.56E-05		*PRICKLE2*	11.01	8.21	6.97	4.57E-05
BMP2	11.61	8.12	11.25	4.06E-05		*GK*	9.71	6.91	6.93	2.61E-04
HECW2	11.24	7.77	11.09	5.78E-05		*IFI44*	8.28	5.50	6.88	8.15E-04
PSMB9	12.35	8.90	10.93	2.51E-07		*RNF144B*	9.05	6.28	6.82	2.47E-04
CXCL3	13.21	9.77	10.85	4.02E-03		*LITAF*	13.88	11.12	6.78	2.93E-05
HAS1	10.11	6.68	10.82	6.16E-03		*TNFSF13B*	9.18	6.43	6.74	1.75E-05
TMEM140	10.24	6.85	10.50	3.26E-05		*NKX3-1*	10.34	7.59	6.73	4.43E-06
SERPINA3	12.30	8.93	10.41	1.68E-02		*SOCS3*	9.72	6.98	6.71	6.21E-04
GBP3	13.55	10.22	10.02	7.86E-05		*CXCL1*	16.00	13.26	6.69	1.70E-02
CMPK2	7.71	4.40	9.90	7.46E-04		*CASP7*	10.63	7.89	6.69	7.41E-07
TRAFD1	11.71	8.43	9.76	1.27E-05		*LIMK2*	11.05	8.33	6.61	5.21E-05
IRG1	7.55	4.30	9.51	1.61E-04		*EDNRA*	10.97	8.27	6.48	2.47E-04
IRAK2	10.07	6.85	9.32	2.66E-04		*TNFSF10*	6.97	4.29	6.42	1.48E-02
BDKRB2	9.76	6.57	9.13	9.36E-04		*C8orf4*	7.97	5.30	6.37	6.57E-05
NFKB1	14.29	11.11	9.03	1.29E-04		*IL1B*	17.34	14.69	6.29	2.69E-03
TESK2	8.61	5.46	8.90	4.65E-05		*RBM47*	9.41	6.76	6.29	2.22E-04
SLC39A8	13.84	10.72	8.74	1.05E-02		*GBP6*	6.75	4.10	6.29	5.53E-07
SELE	7.49	4.37	8.70	5.71E-05		*RNF213*	12.43	9.78	6.26	5.23E-06
SSTR2	6.73	3.61	8.69	1.80E-03		*TRIM25; MIR3614*	14.73	12.12	6.11	1.01E-05
TRIM21	9.70	6.60	8.59	2.79E-06		*TRIB1*	12.42	9.82	6.10	2.16E-06
SLC25A28	12.34	9.25	8.56	4.38E-05		*NINJ1*	11.21	8.61	6.08	1.15E-06
PLSCR1	12.56	9.49	8.40	9.38E-05		*CCL2*	16.89	14.32	5.91	1.36E-03
CA13	10.31	7.24	8.36	3.80E-04		*CCL7*	8.10	5.54	5.90	1.47E-04
PHACTR1	7.99	4.94	8.28	2.56E-05		*PDE4B*	6.91	4.37	5.85	1.87E-02
PMAIP1	10.32	7.31	8.04	6.56E-08		*MICB*	11.77	9.25	5.74	4.17E-09
LIPG	9.88	6.90	7.93	3.04E-05		*CXCL2*	13.79	11.29	5.69	3.21E-03
JAK2	10.52	7.56	7.79	8.71E-04		*TRIM69*	8.45	5.94	5.69	2.37E-05
MLKL	11.76	8.81	7.73	5.36E-06		*MAP2*	12.04	9.53	5.69	2.38E-06
RNF19B	8.04	5.11	7.64	7.12E-04		*LYN*	9.88	7.38	5.65	2.79E-05
PARP8	11.08	8.14	7.63	1.30E-05		*MSC*	14.91	12.42	5.61	3.26E-05
PID1	10.51	7.60	7.51	1.53E-02		*INHBA*	13.17	10.68	5.61	2.12E-03

Gene Symbol	M(Hb)sup +OS mean signal (log2)	M(con)sup +OS mean signal (log2)	Fold Change	P value		Gene Symbol	M(Hb)sup +OS mean signal (log2)	M(con)sup +OS mean signal (log2)	Fold Change	P value
DRAM1	14.22	11.74	5.59	1.35E-04		*HELZ2*	7.12	4.98	4.39	1.37E-03
DUSP16	9.22	6.75	5.55	2.63E-03		*IFIT5*	10.68	8.55	4.37	2.44E-06
AMPD3	8.16	5.69	5.54	7.40E-04		*BCOR*	11.08	8.96	4.33	3.26E-04
BTG3	11.79	9.33	5.52	9.41E-04		*SQRDL*	11.89	9.78	4.33	1.32E-04
B3GNT2	11.87	9.42	5.50	4.31E-05		*GRAMD1A*	12.68	10.56	4.33	5.08E-03
NFKB2	13.14	10.70	5.43	1.93E-03		*TNIP1*	13.45	11.34	4.31	5.07E-04
GRAMD3	12.56	10.15	5.33	7.70E-05		*NUAK2*	8.42	6.32	4.28	1.17E-06
TNC	12.13	9.71	5.32	1.21E-02		*ST6GAL1*	9.23	7.16	4.19	1.65E-05
SLC12A7	9.47	7.06	5.32	7.41E-04		*XRN1*	11.66	9.60	4.15	1.24E-05
NRP2	13.63	11.25	5.22	1.49E-04		*IRAK3; MIR6502*	13.96	11.90	4.15	6.77E-03
PSMB8	11.83	9.46	5.17	5.90E-05		*CCDC71L*	9.57	7.52	4.13	1.14E-03
TNS3	12.33	9.98	5.11	2.18E-04		*CACHD1*	9.46	7.42	4.11	1.06E-04
IL6	17.69	15.37	4.99	5.79E-03		*SP110*	14.70	12.67	4.10	4.39E-04
RNF114	13.16	10.85	4.99	2.93E-06		*PTPN2*	11.64	9.61	4.08	9.68E-05
ZNF267	11.37	9.05	4.98	2.21E-06		*ANO4*	7.07	5.04	4.07	7.72E-05
DUSP16	8.72	6.42	4.93	9.85E-04		*BAK1*	10.36	8.35	4.04	1.63E-07
DHX58	8.37	6.07	4.92	2.69E-04		*SDC4*	14.03	12.02	4.03	1.15E-04
NOCT	12.27	9.97	4.92	1.59E-04		*NRG1*	12.48	10.48	4.02	1.88E-04
NUB1	13.07	10.78	4.90	1.09E-04		*RTP4*	5.89	3.89	4.01	8.96E-04
RELB	10.53	8.24	4.87	1.67E-03		*PRRG4*	7.92	5.91	4.01	3.13E-04
RNF122	8.94	6.68	4.81	3.56E-04		*PDPN*	8.15	6.16	3.98	2.87E-04
STARD8	7.60	5.35	4.76	7.42E-04		*TRIM22*	13.26	11.28	3.96	4.86E-05
TNIP3	6.75	4.51	4.74	9.53E-03		*IRF8*	5.59	3.63	3.90	3.73E-05
SERPING1	13.57	11.32	4.73	7.00E-03		*BCL2A1*	7.60	5.63	3.90	3.39E-04
IL15	8.32	6.09	4.67	7.09E-05		*PARP12*	11.24	9.28	3.89	5.78E-05
SP140L	10.10	7.88	4.66	4.58E-06		*IFI6*	12.62	10.67	3.88	4.38E-04
IFI35	9.62	7.42	4.59	4.65E-05		*SLC39A14*	15.98	14.02	3.87	4.91E-03
SLC2A6	11.25	9.05	4.59	1.84E-04		*ZNFX1*	12.87	10.92	3.86	3.92E-05
SLFN11	11.68	9.49	4.57	3.68E-04		*SAA1*	7.75	5.81	3.86	4.73E-02
HLA-E	13.03	10.85	4.54	7.02E-06		*FOSL2*	13.16	11.22	3.84	5.12E-04
MX2	7.29	5.10	4.54	3.10E-03		*DUSP16*	10.23	8.29	3.83	2.36E-03
SERPINB2	15.52	13.34	4.51	7.17E-03		*FAM107A*	8.80	6.87	3.82	1.91E-04
SLC2A6	8.71	6.56	4.45	9.38E-05		*NMI*	9.49	7.56	3.81	2.35E-05
IRF2	11.51	9.36	4.44	1.12E-04		*RALA*	13.20	11.27	3.79	1.96E-04
FST	13.10	10.95	4.43	6.90E-04		*DUSP16*	7.89	5.96	3.79	8.89E-04
ICAM4	8.61	6.47	4.42	4.17E-05		*HAS2*	10.42	8.51	3.76	1.48E-02
SLC2A6	10.34	8.20	4.41	1.57E-05		*IL15RA*	11.27	9.36	3.74	3.55E-05

Gene Symbol	M(Hb)sup +OS mean signal (log2)	M(con)sup +OS mean signal (log2)	Fold Change	P value		Gene Symbol	M(Hb)sup +OS mean signal (log2)	M(con)sup +OS mean signal (log2)	Fold Change	P value
MSX2	8.97	7.06	3.74	5.54E-04		*ARL5B*	12.31	10.58	3.33	3.90E-07
ATP10A	9.12	7.22	3.73	5.23E-03		*AK4*	11.58	9.86	3.29	4.60E-05
CPNE8	12.65	10.75	3.72	2.69E-05		*TNFAIP3*	17.10	15.38	3.28	6.62E-03
P2RY6	9.75	7.86	3.69	2.05E-04		*SH3BP2*	11.70	9.99	3.27	4.15E-05
BTN3A2	11.95	10.07	3.69	5.44E-03		*SAMHD1*	13.34	11.64	3.25	6.69E-03
SPECC1L-ADORA2A	9.61	7.73	3.69	1.55E-03		*IL31RA*	6.84	5.14	3.24	4.19E-02
CD83	7.17	5.29	3.67	2.46E-04		*CXCL5*	14.79	13.11	3.21	4.46E-02
MB21D1	8.04	6.16	3.67	7.87E-03		*HIVEP2*	11.53	9.85	3.20	6.69E-04
IRF9	11.51	9.63	3.67	1.11E-05		*SOD2*	17.37	15.69	3.20	8.22E-03
LACTB	11.57	9.70	3.66	5.94E-04		*WARS*	17.00	15.32	3.20	2.25E-06
MYB	7.87	6.00	3.66	1.30E-02		*HAS3*	5.53	3.86	3.20	1.38E-04
PML	14.42	12.56	3.65	3.39E-07		*PIM1*	10.27	8.59	3.19	5.16E-04
SLC43A2	10.75	8.88	3.64	5.29E-04		*CCL13*	6.46	4.79	3.16	8.45E-03
RELA	13.68	11.82	3.62	2.45E-05		*CREB5*	6.84	5.18	3.16	1.12E-03
STAT1	16.36	14.51	3.60	2.79E-06		*STX11*	7.65	5.99	3.15	4.27E-04
SLC25A37	9.96	8.13	3.57	4.80E-04		*TMEM63B*	13.01	11.35	3.14	3.95E-05
SLC2A6	10.92	9.09	3.55	1.65E-06		*CCDC109B*	12.32	10.67	3.13	8.42E-03
C19orf66	10.20	8.38	3.53	2.46E-06		*TRAF1*	7.42	5.77	3.13	6.00E-03
C3orf38	10.87	9.06	3.52	2.74E-04		*OPTN*	13.57	11.93	3.12	2.13E-05
WTAP	14.40	12.58	3.52	6.49E-03		*IFITM1*	16.16	14.53	3.11	2.13E-03
BATF2	7.96	6.15	3.51	5.00E-07		*MITD1*	9.01	7.38	3.10	5.20E-03
SOCS1	6.86	5.05	3.51	1.22E-03		*LAP3*	6.52	4.88	3.10	1.81E-03
PRPF3	13.33	11.52	3.51	7.23E-06		*RAB38*	9.32	7.69	3.10	4.69E-05
SERPINB9	8.40	6.59	3.49	3.14E-03		*KRAS*	11.73	10.10	3.09	1.83E-02
ARHGAP31	12.53	10.73	3.49	1.37E-05		*PTGES*	11.18	9.56	3.09	2.28E-02
POU2F2	7.64	5.85	3.45	2.50E-03		*IL7*	8.57	6.95	3.06	5.31E-04
CASP10	6.68	4.89	3.45	5.32E-03		*LRCH2*	9.13	7.52	3.06	1.18E-03
MYO1B	12.95	11.17	3.44	9.93E-04		*PSME2; MIR7703*	11.03	9.42	3.06	3.81E-05
APOBEC3G	7.27	5.49	3.43	1.52E-05		*IFNAR2*	13.33	11.72	3.05	1.41E-03
TNFRSF1B; MIR4632; MIR7846	11.20	9.42	3.43	3.74E-05		*IKZF4*	7.71	6.10	3.05	1.59E-04
DENND5A	12.88	11.11	3.41	1.54E-04		*SLC11A2*	10.57	8.96	3.05	1.40E-03
JAK2	8.51	6.75	3.38	2.24E-03		*C10orf10*	14.85	13.24	3.05	1.25E-02
GBP7	6.36	4.61	3.37	5.92E-04		*SBNO2*	14.78	13.17	3.04	4.29E-06
RIN2	11.87	10.11	3.36	4.07E-05		*CHN1*	10.22	8.63	3.02	1.22E-04
STAT2	12.46	10.72	3.36	1.81E-04		*SPTLC3*	9.88	8.30	2.99	2.53E-02
ASPHD2	6.46	4.72	3.34	7.50E-06		*GTPBP1*	9.68	8.11	2.98	1.49E-04
TMEM2	13.71	11.98	3.33	2.38E-04		*LONRF1*	8.73	7.17	2.96	6.81E-03

Gene Symbol	M(Hb)sup +OS mean signal (log2)	M(con)sup +OS mean signal (log2)	Fold Change	P value		Gene Symbol	M(Hb)sup +OS mean signal (log2)	M(con)sup +OS mean signal (log2)	Fold Change	P value
PLAGL2	10.86	9.29	2.95	1.95E-05		*ZNF22*	9.82	8.35	2.77	2.33E-02
CDC42EP2	12.91	11.35	2.95	3.09E-05		*LHFPL2*	12.72	11.25	2.77	3.27E-04
CSRNP2	10.42	8.86	2.95	4.06E-05		*KIAA1644*	9.40	7.94	2.76	4.06E-03
DUSP5	12.67	11.11	2.94	6.87E-06		*SLC41A2*	9.92	8.47	2.74	2.12E-04
ATP6V1B2	13.28	11.73	2.94	2.68E-05		*OTUD4*	14.33	12.87	2.74	1.46E-04
SP100	14.08	12.54	2.92	5.89E-05		*SLC15A3*	6.73	5.28	2.73	5.72E-04
C1QTNF1	14.75	13.20	2.91	1.56E-02		*CYTH1*	9.64	8.19	2.72	1.57E-05
CHST3	11.32	9.80	2.88	8.94E-05		*SLC25A12*	11.13	9.69	2.71	5.68E-04
CD40	7.95	6.42	2.87	5.79E-06		*TNFAIP1*	12.88	11.45	2.70	1.77E-04
CLIC2	7.31	5.79	2.87	8.85E-04		*CPEB4*	10.88	9.45	2.70	1.93E-04
CYLD	10.52	9.00	2.87	2.03E-03		*NNMT*	12.00	10.57	2.68	7.84E-03
CISH	6.23	4.71	2.86	2.86E-02		*ISOC1*	10.47	9.05	2.68	6.09E-03
ZC3HAV1	12.42	10.91	2.86	4.29E-05		*TEAD4*	8.66	7.23	2.68	1.78E-04
ELOVL7	6.44	4.93	2.86	2.92E-04		*PSMB10*	8.64	7.21	2.68	1.31E-05
OLFML2B	10.70	9.19	2.86	7.72E-03		*LMTK2*	10.44	9.02	2.68	2.04E-04
BTN3A3	11.47	9.95	2.86	2.74E-03		*PPA1*	12.47	11.05	2.68	5.34E-04
CSRNP1	8.93	7.41	2.86	3.76E-03		*ARHGAP42*	8.32	6.90	2.67	2.22E-04
TMEM217	8.56	7.05	2.86	5.68E-03		*VEGFC*	14.07	12.65	2.67	2.81E-04
ETS1	13.55	12.04	2.85	4.50E-04		*BCL3; MIR8085*	10.49	9.08	2.66	3.28E-05
UBE2L6	14.90	13.39	2.85	4.31E-05		*MORC3*	11.67	10.26	2.66	6.47E-04
TMEM106A	9.93	8.42	2.85	1.85E-04		*BOC*	8.33	6.92	2.66	1.36E-03
ITPKC	8.94	7.43	2.84	1.18E-04		*UGP2*	12.21	10.80	2.65	2.67E-02
TMEM51	7.79	6.29	2.84	4.39E-06		*YPEL2*	8.62	7.22	2.65	2.80E-03
JUN	11.41	9.92	2.81	1.52E-04		*CREM*	10.97	9.58	2.63	8.79E-03
BTN2A1	11.34	9.84	2.81	1.15E-03		*CASP8*	9.59	8.19	2.63	3.42E-03
TBC1D9	10.91	9.42	2.81	2.35E-04		*ZNF792*	8.65	7.25	2.63	9.32E-03
CYP1B1	14.24	12.75	2.81	4.66E-03		*SCYL3*	9.19	7.80	2.63	3.09E-04
OGFR	13.40	11.92	2.80	1.29E-04		*SAMD4A*	12.27	10.88	2.62	6.91E-04
MMAA	10.21	8.73	2.80	3.29E-04		*CD47*	12.31	10.92	2.62	1.04E-03
DNAJA1	14.89	13.40	2.80	3.58E-05		*TDRD7*	8.95	7.57	2.61	5.40E-05
PANX1	14.06	12.58	2.80	4.65E-03		*IFIT1*	6.97	5.59	2.60	9.20E-03
AIM2	6.18	4.71	2.78	3.26E-02		*EMP2*	14.60	13.22	2.60	1.33E-03
BAZ1A	12.04	10.56	2.78	1.71E-05		*IER3*	16.51	15.13	2.60	5.55E-03
SLC30A7	14.33	12.85	2.78	2.35E-05		*KSR1*	9.57	8.19	2.60	5.10E-03
PIGV	9.29	7.82	2.77	1.56E-03		*LRCH1*	9.51	8.13	2.60	2.12E-03
B4GALT5	12.79	11.31	2.77	9.14E-05		*PSMB8-AS1*	5.50	4.13	2.60	4.73E-02
TAB3	13.28	11.81	2.77	1.68E-03		*DOCK10*	10.81	9.44	2.59	8.60E-04

Gene Symbol	M(Hb)sup +OS mean signal (log2)	M(con)sup +OS mean signal (log2)	Fold Change	P value		Gene Symbol	M(Hb)sup +OS mean signal (log2)	M(con)sup +OS mean signal (log2)	Fold Change	P value
SLC9B2	8.44	7.06	2.59	2.57E-04		*APOBEC3D*	9.44	8.15	2.45	5.73E-05
MUC1	8.56	7.19	2.59	5.83E-03		*TRIM26*	11.23	9.94	2.45	7.60E-05
PIM2	9.32	7.95	2.58	1.37E-02		*C21orf91*	10.37	9.08	2.44	2.21E-04
TRANK1	8.50	7.13	2.58	2.43E-03		*MOB3C*	10.23	8.95	2.43	1.22E-05
PCYT1A	12.41	11.05	2.58	3.76E-04		*OLR1*	5.87	4.59	2.43	7.42E-04
ADAR	11.71	10.34	2.58	3.30E-05		*INSIG2*	11.00	9.72	2.43	6.04E-03
TOP1	13.27	11.91	2.57	2.62E-04		*HLA-L*	13.71	12.43	2.43	1.92E-04
NAMPT	12.75	11.39	2.57	4.37E-02		*FAM231D; LINC00869*	11.27	10.00	2.42	2.12E-04
TBX3	12.98	11.62	2.57	1.85E-03		*GTF2B*	10.81	9.54	2.41	5.73E-06
SMAD7	10.40	9.04	2.57	9.66E-05		*HLA-B*	11.89	10.62	2.41	9.68E-04
IL33	14.17	12.81	2.56	2.42E-03		*TRAF3IP2*	10.65	9.38	2.41	9.31E-06
SPHK1	13.88	12.52	2.56	1.55E-03		*TXNL4B*	11.21	9.95	2.40	3.71E-05
NDST2	9.75	8.39	2.56	5.79E-04		*VDR*	10.93	9.66	2.40	8.67E-03
BATF3	7.98	6.63	2.54	6.13E-04		*CNDP2*	14.41	13.15	2.39	2.28E-04
C2CD4A	5.68	4.34	2.53	3.04E-02		*TMEM133*	7.19	5.93	2.39	1.86E-02
SDE2	10.15	8.81	2.52	1.10E-03		*DDX23*	12.78	11.52	2.38	1.17E-03
TRIM36	4.50	3.17	2.52	5.56E-03		*PTPN1*	15.05	13.80	2.38	6.56E-04
CXCL8	16.88	15.55	2.52	3.51E-02		*PCYT1A*	12.91	11.66	2.37	3.51E-04
SERPINB8	13.03	11.70	2.51	7.57E-04		*ACKR4*	6.42	5.18	2.37	3.83E-02
HSPB8	10.86	9.53	2.51	2.87E-02		*HIVEP1*	10.82	9.57	2.37	1.34E-02
ACSL4	12.91	11.59	2.51	1.17E-02		*BID*	11.71	10.46	2.37	1.84E-02
FMNL3	8.93	7.60	2.50	1.17E-03		*CFLAR*	15.58	14.34	2.36	1.42E-04
NAB1	10.66	9.34	2.50	1.36E-02		*CLEC2D*	7.06	5.83	2.35	2.57E-02
STARD13	9.87	8.55	2.50	3.54E-02		*FEM1C*	12.66	11.43	2.34	1.85E-02
PPP4R4	9.11	7.79	2.50	1.00E-02		*RDH10*	12.74	11.51	2.34	6.69E-05
DUSP16	7.87	6.55	2.50	2.93E-03		*CTSC*	16.35	15.13	2.33	1.97E-05
PTPN12	12.58	11.26	2.49	2.74E-04		*GFPT2*	15.41	14.20	2.33	6.01E-03
ODF2L	10.49	9.18	2.49	5.21E-05		*BAZ2A*	12.28	11.08	2.31	6.02E-05
DENND4A	11.61	10.29	2.49	7.87E-05		*STX12*	11.92	10.71	2.31	4.66E-03
RASSF4	8.83	7.52	2.48	3.18E-02		*HNRNPF*	4.89	3.69	2.31	4.55E-02
MYD88	11.72	10.41	2.48	8.15E-05		*TTC7B*	10.61	9.41	2.31	1.72E-04
WSB1	11.59	10.28	2.48	1.70E-03		*SPSB1*	12.81	11.61	2.30	4.20E-06
C6orf132	9.41	8.10	2.48	1.39E-04		*C5AR2*	6.67	5.47	2.29	8.70E-04
UBR1	13.03	11.72	2.48	1.09E-05		*NR4A3*	7.58	6.38	2.29	3.77E-02
VPS54	8.51	7.21	2.46	1.23E-02		*FNIP2*	8.44	7.25	2.29	1.78E-02
GPR68	5.39	4.09	2.46	2.48E-04		*DNER*	6.70	5.51	2.28	4.10E-02
RGL1	12.44	11.15	2.46	3.36E-02		*FLT1*	7.44	6.25	2.28	2.25E-04

Gene Symbol	M(Hb)sup +OS mean signal (log2)	M(con)sup +OS mean signal (log2)	Fold Change	P value		Gene Symbol	M(Hb)sup +OS mean signal (log2)	M(con)sup +OS mean signal (log2)	Fold Change	P value
BTN3A1	9.91	8.73	2.28	5.91E-03		*LMO4*	12.05	10.93	2.17	3.53E-02
BIRC2	11.73	10.54	2.28	1.08E-02		*MRPL32*	10.98	9.86	2.17	5.67E-03
HDX	9.13	7.94	2.27	8.11E-05		*ZBTB17*	8.39	7.27	2.16	1.28E-03
SYNC	10.78	9.59	2.27	1.97E-02		*ARNTL2*	10.72	9.60	2.16	1.54E-02
EYA3	8.64	7.46	2.27	1.77E-04		*CYB561D2*	8.23	7.11	2.16	3.26E-04
SLFN5	12.66	11.48	2.26	6.70E-04		*PCED1A*	10.68	9.57	2.16	4.68E-03
RNF24	13.85	12.68	2.26	2.00E-04		*DDX60*	8.50	7.39	2.16	2.50E-02
SPATA13; C1QTNF9	7.16	5.98	2.26	8.64E-03		*PLSCR2*	7.64	6.54	2.15	5.02E-04
USP42	11.59	10.42	2.25	1.61E-04		*GATA2*	7.65	6.55	2.15	9.52E-04
PSEN1	12.35	11.18	2.25	1.26E-03		*EFHD2*	11.54	10.44	2.15	2.29E-04
DCP1A	9.00	7.83	2.25	1.24E-04		*CBLB*	12.74	11.64	2.14	5.16E-05
FBXO6	6.91	5.74	2.25	7.14E-04		*AZIN2*	7.86	6.76	2.14	4.63E-04
EHD1	12.39	11.23	2.24	3.37E-02		*RIPK1*	10.84	9.74	2.13	3.84E-05
ETV6	11.64	10.47	2.24	8.89E-05		*PSME1*	11.67	10.57	2.13	5.61E-04
C10orf25	6.32	5.16	2.24	3.30E-03		*NFKBIA*	14.93	13.84	2.13	8.02E-03
FZD5	7.00	5.84	2.24	1.33E-04		*APOL4*	6.79	5.70	2.13	2.25E-03
ETS2	12.55	11.38	2.24	3.32E-04		*TRIM22*	8.73	7.64	2.13	5.42E-03
HLA-F	10.16	9.00	2.24	1.46E-04		*RFX5*	10.42	9.33	2.13	6.42E-03
SHISA2	5.37	4.21	2.23	5.21E-03		*NUDCD1*	12.32	11.23	2.13	5.32E-03
TMEM170A	10.08	8.93	2.22	5.02E-05		*VCPIP1*	11.05	9.97	2.12	9.84E-05
HSBP1L1	9.72	8.57	2.22	5.92E-03		*RABGGTA*	10.68	9.60	2.12	1.44E-02
SNTB2	9.64	8.49	2.22	2.11E-03		*ZC3H12C*	11.92	10.84	2.12	4.38E-02
ANXA2R	6.30	5.15	2.21	6.15E-03		*PHACTR4*	10.84	9.76	2.12	6.29E-04
CASP1	7.29	6.15	2.21	7.63E-04		*PZP*	5.88	4.80	2.11	4.34E-02
CDC42SE1	12.61	11.47	2.21	4.85E-05		*AKAP12*	14.13	13.05	2.11	2.45E-03
RELL1	12.11	10.97	2.20	4.20E-02		*VSNL1*	7.56	6.48	2.11	8.85E-04
NLRC5	6.81	5.67	2.20	7.96E-04		*ACSL5*	7.69	6.61	2.11	4.72E-04
4-Sep	6.09	4.96	2.20	3.26E-02		*ARID5A*	9.46	8.38	2.11	1.82E-04
KCNJ15	6.35	5.21	2.20	6.37E-05		*PPP2R1B*	10.92	9.84	2.10	1.05E-02
STAT5A	10.90	9.76	2.19	4.87E-04		*TP53BP2*	12.23	11.16	2.10	6.57E-05
EPT1	9.30	8.17	2.19	5.06E-04		*KREMEN1*	9.14	8.07	2.10	1.98E-03
BCL6	15.16	14.03	2.19	1.29E-03		*SAT1*	13.67	12.60	2.10	1.89E-02
TANC1	9.03	7.91	2.18	8.95E-04		*NXT2*	10.07	9.00	2.10	1.23E-02
MR1	9.25	8.13	2.18	2.52E-03		*ATP2B1*	12.64	11.57	2.10	3.82E-02
KLF10	12.14	11.02	2.17	3.28E-04		*STXBP1*	13.88	12.81	2.09	1.09E-03
PPARD	12.78	11.66	2.17	1.53E-03		*BST2*	5.03	3.96	2.09	4.95E-03
IFI27	8.44	7.32	2.17	1.70E-02		*ADAP1*	6.85	5.78	2.09	1.36E-04

Gene Symbol	M(Hb)sup +OS mean signal (log2)	M(con)sup +OS mean signal (log2)	Fold Change	P value		Gene Symbol	M(Hb)sup +OS mean signal (log2)	M(con)sup +OS mean signal (log2)	Fold Change	P value
PPP1R12A	10.61	9.55	2.09	2.09E-03		*HERC6*	7.73	6.72	2.00	1.12E-02
ABHD16A	9.61	8.55	2.08	9.23E-04		*NFIA*	10.63	11.63	-2.00	4.55E-04
TAF4B	7.49	6.44	2.08	3.86E-02		*TRIM2*	8.69	9.69	-2.00	4.54E-03
MFSD2A	12.67	11.62	2.08	6.89E-03		*CMTR2*	7.84	8.85	-2.01	1.05E-02
CDKN1A	14.34	13.29	2.08	3.58E-02		*LRRC8D*	11.29	12.30	-2.02	5.36E-03
ZNF486	6.55	5.50	2.07	1.71E-03		*FBXO25*	8.18	9.19	-2.02	8.48E-03
BLZF1	12.48	11.44	2.06	1.61E-04		*RASD1*	7.15	8.16	-2.02	4.77E-02
ADPRHL2	7.47	6.43	2.06	8.32E-04		*TMEM47*	14.10	15.11	-2.02	1.17E-03
FRRS1	6.15	5.11	2.06	7.44E-03		*SH2D4A*	5.03	6.04	-2.02	7.33E-04
PNRC2	12.89	11.85	2.06	1.50E-02		*TRAM2*	14.23	15.24	-2.02	8.33E-03
SRFBP1	10.61	9.57	2.06	1.06E-02		*NUMA1*	10.16	11.18	-2.02	1.07E-03
PPAP2B	7.40	6.36	2.06	2.89E-02		*SF3B5*	9.57	10.59	-2.03	1.24E-02
ENC1	9.00	7.96	2.06	2.64E-02		*SETDB2*	5.91	6.93	-2.03	2.03E-02
FBXL14	10.85	9.81	2.05	7.95E-03		*HDAC5*	8.12	9.14	-2.03	4.51E-04
HS3ST3B1	13.57	12.53	2.05	6.26E-03		*KCTD1*	8.09	9.11	-2.03	2.04E-04
C12orf4	10.51	9.47	2.05	2.46E-03		*MBNL1*	14.03	15.05	-2.03	1.07E-03
DDX21	14.34	13.30	2.05	1.39E-03		*TCF7L2*	5.90	6.93	-2.04	3.42E-04
RBMS1; MIR4785	12.47	11.44	2.05	4.98E-03		*RPS6KA6*	6.18	7.21	-2.04	2.16E-03
KIRREL	13.38	12.35	2.05	4.76E-04		*SHPRH*	7.05	8.08	-2.04	5.13E-03
FGF2	14.53	13.50	2.04	1.26E-02		*MRGPRF*	7.27	8.30	-2.04	2.04E-03
CD2AP	10.26	9.23	2.04	7.83E-04		*ZNF614*	5.52	6.55	-2.04	7.40E-03
RTTN	8.64	7.61	2.03	5.72E-03		*SLC7A11*	12.12	13.15	-2.04	1.48E-03
LONRF3	4.42	3.39	2.03	4.66E-03		*C10orf2*	7.68	8.72	-2.04	3.95E-03
LRIG1	10.53	9.51	2.03	1.38E-03		*NREP*	12.71	13.75	-2.05	1.66E-02
ITPRIP	11.39	10.37	2.03	1.50E-03		*ZMAT3*	10.19	11.23	-2.05	1.00E-02
IFNGR2	15.17	14.15	2.03	3.59E-03		*RHOBTB1*	5.62	6.66	-2.06	3.27E-04
SLCO5A1	6.29	5.27	2.03	6.62E-03		*TMEM168*	6.83	7.87	-2.06	1.36E-02
PDCD1LG2	11.31	10.29	2.02	2.78E-02		*ZNF639*	9.69	10.74	-2.06	5.00E-03
3-Mar	12.62	11.60	2.02	2.83E-02		*NFIB*	12.97	14.02	-2.06	3.03E-04
GLA	10.75	9.74	2.02	3.10E-03		*ACVRL1*	7.58	8.63	-2.07	3.61E-02
PCDH17	6.76	5.74	2.02	2.10E-02		*FAM200A*	5.39	6.44	-2.07	1.12E-03
GPD2	9.84	8.83	2.02	4.42E-03		*C2CD2*	8.78	9.84	-2.08	5.75E-04
TYMP; SCO2	6.95	5.94	2.01	5.41E-03		*ARL4A*	6.44	7.50	-2.08	4.13E-03
BACH1	11.85	10.85	2.01	1.41E-03		*TCEAL1*	9.08	10.14	-2.09	3.82E-03
APOBEC3F	8.00	6.99	2.01	1.09E-03		*CAMK2G*	8.30	9.36	-2.09	3.97E-04
HMOX2	11.97	10.96	2.01	1.85E-03		*DIXDC1*	9.03	10.09	-2.09	1.42E-03
ZNF189	10.99	9.98	2.01	3.63E-02		*GTF2A1L; STON1*	6.19	7.25	-2.09	1.84E-03

Gene Symbol	M(Hb)sup +OS mean signal (log2)	M(con)sup +OS mean signal (log2)	Fold Change	P value		Gene Symbol	M(Hb)sup +OS mean signal (log2)	M(con)sup +OS mean signal (log2)	Fold Change	P value
RNF141	10.63	11.70	-2.09	6.50E-03		*WEE1*	10.17	11.29	-2.17	9.25E-03
KANK2	7.20	8.27	-2.09	4.74E-03		*CDC37L1*	7.80	8.92	-2.18	1.09E-03
F3	10.78	11.85	-2.09	2.79E-02		*ZHX3*	11.03	12.16	-2.18	4.67E-03
CEP85	4.07	5.14	-2.09	8.09E-04		*EFNA5*	8.47	9.60	-2.18	1.33E-02
EVA1C	8.32	9.39	-2.10	4.80E-03		*AKR1B10*	5.94	7.07	-2.19	1.78E-02
ARHGEF26	5.79	6.86	-2.10	2.62E-02		*ROR1*	11.64	12.77	-2.20	2.86E-02
SH3BP5	9.35	10.42	-2.10	3.85E-03		*ZNF260*	8.23	9.37	-2.20	3.20E-03
ANKRD34A	3.96	5.03	-2.10	4.45E-04		*RCOR3*	7.14	8.28	-2.21	1.41E-03
ADORA2B	7.32	8.39	-2.10	1.23E-02		*KAT6B*	5.53	6.67	-2.21	9.38E-05
APOOL	8.58	9.65	-2.10	6.37E-04		*BAMBI*	9.53	10.68	-2.21	2.74E-05
ERCC6; PGBD3	11.51	12.59	-2.11	1.75E-02		*ZNF780A*	6.07	7.22	-2.21	4.62E-02
GTPBP8	9.80	10.88	-2.12	3.57E-04		*TMOD2*	6.18	7.33	-2.22	2.20E-02
ABHD17C	7.80	8.88	-2.12	6.11E-03		*CUL4B*	13.03	14.18	-2.23	8.40E-04
MKL2; TVP23CP2	8.15	9.23	-2.12	2.64E-04		*EBF1*	7.60	8.76	-2.23	2.87E-04
PXYLP1	5.81	6.90	-2.12	6.80E-03		*GTF2E1*	7.11	8.27	-2.23	4.27E-02
TP53TG3B; TP53TG3; TP53TG3C	6.12	7.20	-2.12	5.23E-03		*NPR3*	9.26	10.42	-2.24	1.32E-02
ZNF385D	5.62	6.70	-2.12	2.25E-02		*FNBP1L*	5.92	7.08	-2.24	1.91E-03
NAT6	7.34	8.43	-2.13	2.19E-03		*ERRFI1*	15.74	16.91	-2.24	2.29E-03
ENOX1	7.23	8.32	-2.13	1.13E-02		*RASSF9*	3.17	4.34	-2.25	5.12E-04
CXXC5	8.06	9.15	-2.13	1.41E-02		*SOX12*	5.06	6.23	-2.25	8.66E-04
GNAI1	10.35	11.45	-2.13	2.88E-02		*GCNT1*	5.31	6.49	-2.26	7.36E-04
TSEN2	6.31	7.40	-2.14	3.83E-03		*CERK*	9.01	10.19	-2.26	2.44E-04
PANK1	6.83	7.93	-2.14	2.95E-03		*PTK2B*	6.73	7.91	-2.27	2.15E-02
ZNF623	7.48	8.58	-2.14	1.57E-02		*CEP68*	7.57	8.76	-2.27	1.82E-03
DCLK1	5.91	7.01	-2.14	7.56E-04		*KLF12*	8.67	9.86	-2.28	7.00E-05
SLC7A8	5.62	6.72	-2.15	1.07E-02		*AGFG2*	6.79	7.99	-2.29	1.69E-03
DNAJC6	5.36	6.46	-2.15	1.19E-02		*HIST1H3D; HIST1H2AD*	5.63	6.82	-2.29	3.04E-02
DCLK2	7.68	8.78	-2.15	1.80E-03		*STPG1*	6.72	7.92	-2.29	6.28E-03
ACVR2A	8.73	9.83	-2.15	1.43E-03		*FLRT2*	8.06	9.26	-2.30	3.71E-02
CUTC	8.28	9.39	-2.16	3.12E-03		*NAPEPLD*	7.81	9.01	-2.30	2.29E-05
TCEAL8	8.43	9.54	-2.16	6.20E-04		*GYG2*	6.15	7.36	-2.30	1.12E-02
NT5DC2	13.12	14.23	-2.16	6.80E-04		*ADAMTS15*	4.72	5.92	-2.31	1.50E-02
ABHD4	7.89	9.00	-2.16	2.05E-02		*ZNF81*	5.18	6.39	-2.31	1.24E-03
ZRANB1	11.66	12.78	-2.17	2.07E-04		*FADS2*	13.32	14.53	-2.32	2.39E-02
FAM172A; POU5F2; MIR2277	8.56	9.68	-2.17	3.07E-04		*TES*	11.86	13.09	-2.34	2.80E-02
ARHGAP20	7.64	8.76	-2.17	1.71E-02		*TIMM21*	9.49	10.72	-2.35	2.61E-04

Gene Symbol	M(Hb)sup +OS mean signal (log2)	M(con)sup +OS mean signal (log2)	Fold Change	P value		Gene Symbol	M(Hb)sup +OS mean signal (log2)	M(con)sup +OS mean signal (log2)	Fold Change	P value
PTPN3	6.90	8.14	-2.36	3.06E-03		*AGPAT5*	7.90	9.24	-2.54	6.30E-03
C5orf30	8.14	9.38	-2.36	1.23E-02		*EDA2R*	9.49	10.84	-2.55	3.54E-04
SESN1	7.41	8.65	-2.36	5.72E-03		*PRKAG2*	9.86	11.21	-2.55	1.56E-03
TIMP4	6.59	7.84	-2.37	1.12E-03		*ICK*	8.80	10.15	-2.56	2.69E-03
SH3D19	10.53	11.78	-2.38	2.74E-03		*RUNX2*	10.00	11.36	-2.57	1.52E-03
H1F0	8.23	9.48	-2.38	2.39E-03		*DTNA*	5.60	6.96	-2.58	2.65E-04
SPATC1L	7.81	9.07	-2.39	1.41E-02		*FOXN3*	9.25	10.62	-2.58	1.05E-02
PMP22	11.58	12.83	-2.39	3.46E-02		*PET117; CSRP2BP*	6.82	8.19	-2.59	2.30E-03
MTURN	5.96	7.21	-2.39	2.96E-03		*SULT1E1*	11.29	12.67	-2.59	1.20E-03
GABRE; MIR224; MIR452	9.97	11.23	-2.40	4.30E-03		*MAP3K12*	7.96	9.33	-2.59	9.09E-04
FKBP5	12.94	14.20	-2.40	2.93E-02		*OPN3*	7.08	8.46	-2.61	1.59E-03
CYP2R1	5.04	6.31	-2.40	2.63E-03		*PELI2*	4.60	5.98	-2.61	7.61E-05
AP4B1	5.75	7.01	-2.40	1.28E-03		*FIGN*	5.48	6.88	-2.63	2.06E-02
CRNDE	11.02	12.28	-2.40	1.31E-02		*MYC*	11.62	13.02	-2.64	6.78E-05
MEGF9	7.20	8.47	-2.41	9.94E-05		*RHOU*	6.85	8.25	-2.65	1.08E-02
BTBD3	8.60	9.87	-2.41	2.21E-04		*PRR16*	7.86	9.27	-2.66	2.74E-02
STXBP5	11.27	12.54	-2.42	1.02E-04		*TP53TG3D*	5.64	7.06	-2.67	1.28E-03
NEDD4	10.43	11.70	-2.42	1.66E-03		*MECOM*	8.36	9.78	-2.69	3.09E-04
NXPE3	7.98	9.26	-2.42	2.33E-05		*TSHZ1*	6.68	8.11	-2.69	7.08E-04
POT1	9.32	10.60	-2.43	1.41E-02		*RFTN2*	6.37	7.80	-2.70	3.39E-02
JDP2	10.22	11.50	-2.44	8.11E-03		*APPL2*	7.77	9.21	-2.71	4.26E-03
BDNF	6.79	8.07	-2.44	6.12E-03		*GPAT3*	4.68	6.12	-2.72	1.98E-04
KITLG	9.39	10.68	-2.45	1.92E-04		*PRKD1*	7.66	9.10	-2.72	2.04E-04
YPEL3	10.35	11.65	-2.46	1.48E-04		*SSX2IP*	8.51	9.96	-2.73	2.48E-04
LDB1	12.02	13.32	-2.46	5.71E-04		*CAMKK1*	5.58	7.03	-2.74	1.52E-03
OSGEPL1	5.18	6.48	-2.47	3.93E-04		*GATA6*	9.51	10.96	-2.75	1.45E-04
EFNB1	8.56	9.87	-2.49	1.49E-03		*GPSM2*	8.38	9.85	-2.76	7.53E-03
MED20	5.93	7.25	-2.50	3.84E-05		*ZMYM1*	6.98	8.45	-2.79	3.09E-05
GPR1	10.93	12.27	-2.52	2.66E-05		*MGP*	8.14	9.64	-2.82	1.83E-03
BCL2L11	5.96	7.30	-2.52	2.05E-02		*CNTN3*	8.14	9.65	-2.84	8.74E-03
PTCH1	8.09	9.42	-2.52	2.93E-05		*CITED2*	13.10	14.61	-2.84	4.22E-04
KAT6B	8.28	9.61	-2.52	6.44E-05		*TET1*	4.94	6.45	-2.86	1.41E-02
FAM110B	5.79	7.12	-2.52	4.07E-03		*FAT4*	10.78	12.30	-2.86	6.98E-03
ZNF850	6.63	7.98	-2.53	1.25E-04		*ADAMTS1*	14.59	16.14	-2.93	3.56E-05
MLPH	8.14	9.48	-2.53	1.20E-02		*SOBP*	6.10	7.65	-2.94	3.35E-03
TNFAIP8L3	9.18	10.52	-2.54	3.86E-02		*PLIN2*	13.99	15.56	-2.96	2.35E-03
GPER1	5.53	6.87	-2.54	3.44E-03		*MMD*	8.46	10.03	-2.97	1.46E-02

Gene Symbol	M(Hb)sup +OS mean signal (log2)	M(con)sup +OS mean signal (log2)	Fold Change	P value		Gene Symbol	M(Hb)sup +OS mean signal (log2)	M(con)sup +OS mean signal (log2)	Fold Change	P value
BNC2	10.38	11.95	-2.98	4.10E-05		*PIK3R1*	9.71	11.60	-3.72	5.89E-05
PHLPP1	9.01	10.58	-2.98	7.35E-04		*AREG*	7.65	9.59	-3.83	2.85E-03
AKR1C3	12.00	13.59	-3.02	3.02E-04		*TMEM100*	6.70	8.65	-3.87	1.52E-03
CYTH3	9.80	11.40	-3.03	5.53E-03		*ANKRD28*	10.15	12.13	-3.95	3.75E-05
SSH2	9.88	11.49	-3.04	4.77E-04		*NFYB*	8.26	10.26	-4.00	5.08E-05
GADD45A	9.23	10.84	-3.04	5.20E-03		*RGS4*	10.90	12.91	-4.02	4.47E-02
TMEM246	5.53	7.15	-3.08	9.37E-04		*MTSS1*	11.56	13.58	-4.06	8.36E-05
AR	5.48	7.10	-3.08	2.56E-06		*AKR1B15*	6.83	8.87	-4.10	4.23E-04
USP53	12.09	13.73	-3.11	3.47E-02		*CRYAB*	11.11	13.15	-4.11	1.41E-02
ID2	11.43	13.07	-3.11	4.18E-02		*SORBS2*	9.41	11.46	-4.12	3.65E-05
AOX1	8.95	10.59	-3.13	4.64E-05		*FOXO1*	7.22	9.27	-4.13	2.76E-03
KCNJ8	7.38	9.04	-3.15	6.52E-04		*LURAP1L*	8.15	10.30	-4.43	2.54E-07
GPAM	6.97	8.63	-3.16	1.83E-03		*HS6ST1*	7.80	9.95	-4.44	3.41E-04
CALHM2	9.53	11.19	-3.17	1.37E-04		*NEXN*	11.29	13.46	-4.50	4.53E-04
KLHL4	6.46	8.13	-3.18	5.48E-04		*PCDH18*	9.18	11.41	-4.68	1.72E-03
NR2F2	12.48	14.17	-3.21	1.16E-04		*BMP4*	4.87	7.13	-4.79	2.42E-06
MAOA	6.28	7.99	-3.27	1.14E-04		*EBF2*	9.78	12.09	-4.95	3.18E-07
PPARG	6.98	8.69	-3.28	1.94E-05		*DEPTOR*	7.11	9.43	-4.98	4.15E-03
PLCXD3	3.98	5.69	-3.29	9.79E-03		*METTL7A*	9.25	11.76	-5.68	1.21E-03
TNFRSF10D	11.13	12.86	-3.30	1.84E-02		*SDPR*	5.95	8.47	-5.72	4.26E-05
ZADH2	6.90	8.63	-3.30	2.47E-03		*RARB*	6.89	9.41	-5.75	2.00E-04
PDK4	10.15	11.88	-3.33	3.10E-05		*NR2F1*	8.56	11.12	-5.88	1.59E-05
BAG2	10.56	12.29	-3.33	7.21E-05		*KCND2*	7.62	10.21	-6.01	4.35E-04
BHLHE41	7.97	9.70	-3.34	8.00E-04		*LMO3*	5.41	8.04	-6.21	1.88E-03
TGFBR3	10.79	12.56	-3.40	1.06E-04		*ADAMTS5*	6.86	9.52	-6.32	2.76E-05
CYP26B1	5.35	7.12	-3.41	1.19E-04		*PLK2*	9.52	12.20	-6.41	5.08E-06
HAND2	6.26	8.05	-3.46	1.97E-03		*OSR1*	9.59	12.35	-6.80	8.54E-06
PDE7B	8.89	10.69	-3.50	1.79E-05		*SPRY1*	8.14	11.11	-7.85	1.13E-05
SOCS2	6.31	8.13	-3.53	7.52E-03		*STXBP6*	7.13	10.17	-8.22	7.06E-05
IRX3	6.11	7.96	-3.60	1.20E-03		*ADH1B*	4.30	7.72	-10.71	1.53E-02
EPHA4	8.74	10.59	-3.60	8.72E-07		*JADE1*	6.04	9.49	-10.97	1.82E-05
AKR1C2	12.89	14.75	-3.62	7.13E-03		*EGR1*	8.98	13.19	-18.41	9.68E-03
FAXDC2	6.25	8.12	-3.65	2.14E-03		*DKK1*	8.69	13.76	-33.78	2.73E-05
SORT1	7.39	9.28	-3.72	1.31E-02						

Supplemental Table 3. Clinical background and pathologic characteristics of cases with CD163 rs7136716 SNP (AA vs GG genotype carriers)

rs7136716 SNP genotype	AA (n=15)	GG (n=15)	p value
**Demographic**			
Age (years)	46.8±10.8	44.6±6.9	0.51
Male gender	13 (86.7)	11 (73.3%)	0.36
BMI	29.6±6.1	33.6±9.4	0.18
**X-ray calcification score**	25[7-55]	10[5-18]	0.03
**Histomorphometry**			
IEL area	8.6±2.5	7.9±2.3	0.43
Lumen area	1.9±0.9	1.8±0.7	0.95
Plaque area (mean)	6.8±2.4	6.1±2.1	0.40
Plaque area (total)	20.4±7.2	18.3±6.2	0.40
% area stenosis (mean) (%)	77.4±13.4	75.6±10.4	0.68
% area stenosis (max) (%)	87.6±9.6	82.9±10	0.20
Calcification area (mean) (mm^2)	0.27[0.1-1.5]	0.05[0.01-0.26]	0.06
Calcification area (total) (mm^2)	0.82[0.3-4.5]	0.16[0.02-0.77]	0.06
% Calcification/plaque (mean) (%)	4.47[1.61-14.08]	0.64[0.09-2.78]	0.05
% Calcification/plaque (max) (%)	11.92[4.83-28.56]	1.75[0.21-7.46]	0.03
Necrotic core area (mean) (mm^2)	0.11[0.02-0.23]	0.3[0.13-0.51]	0.12
Necrotic core area (total) (mm^2)	0.34[0.07-0.7]	0.9[0.38-1.53]	0.12
% Necrotic core/plaque (mean) (%)	1.59[0.26-2.61]	4.26[1.82-5.66]	0.06
% Necrotic core/plaque (max) (%)	3.7[0.58-5.07]	7.99[3.94-11.34]	0.10

BMI=body mass index, IEL=internal elastic lamina

Supplemental Table 4. Primer sequences for RT-PCR

Target Gene	Forward	Reverse
BMP4	CTGGTCTTGAGTATCCTGAGCG	TCACCTCGTTCTCAGGGATGCT
HAS1	CAAGATTCTTCAGTCTGGAC	TAAGAACGAGGAGAAAGCAG
HAS2	CAGAATCCAAACAGACAGTTC	TAAGGTGTTGTGTGTGACTG
HAS3	CTTAAGGGTTGCTTGCTTGC	GTTCGTGGGAGATGAAGGAA
MGP	GACCCTGAGACTGACCTGCAGGACGA	TCAGTAATGCTGCTACAGGGGGATAC
NFKIA	TCCACTCCATCCTGAAGGCTAC	CAAGGACACCAAAAGCTCCACG
NFKB1	GCAGCACTACTTCTTGACCACC	TCTGCTCCTGAGCATTGACGTC
RELA	TGAACCGAAACTCTGGCAGCTG	CATCAGCTTGCGAAAAGGAGCC
RUNX2	CAGACCAGCAGCACTCCATA	CAGCGTCAACACCATCATTC

Supplemental Table 5. Meta data and rs7136716 genotype for patients in high vs low CD163 analysis in human single cell RNA sequence

No	Sample ID	Age	Gender	Coronary Segment	Disease Category*	Patient group	CD163 expression	rs7136716 genotype
1	scA	57	M	RCA	2	Low CD163	lower quartile	GA
2	scAA	60	F	LCX	3	Low CD163	lower quartile	GA
3	scAC	59	M	LAD	3	Low CD163	lower quartile	GA
4	scAK	32	M	LAD	1	Low CD163	lower quartile	GA
5	scAP	61	F	LAD	1	Low CD163	lower quartile	AA
6	scAQ	61	M	LAD	1	Low CD163	lower quartile	AA
7	scB	61	M	RCA	3	Low CD163	lower quartile	AA
8	scD	61	M	LAD	2	Low CD163	lower quartile	AA
9	scJ	57	F	LAD	3	Low CD163	lower quartile	AA
10	scL	57	F	LAD	3	Low CD163	lower quartile	AA
11	scP	62	M	LAD	3	Low CD163	lower quartile	AA

12	scAB	62	F	LAD	1	High CD163	upper quartile	GA
13	scAM	55	F	LCX	2	High CD163	upper quartile	GA
14	scE	60	M	RCA	1	High CD163	upper quartile	AA
15	scF	62	M	LCX	1	High CD163	upper quartile	AA
16	scG	67	M	LCX	3	High CD163	upper quartile	AA
17	scH	60	M	RCA	2	High CD163	upper quartile	AA
18	scK	71	M	LAD	3	High CD163	upper quartile	AA
19	scS	43	M	RCA	2	High CD163	upper quartile	AA
20	scU	30	M	RCA	1	High CD163	upper quartile	GA
21	scV	63	M	RCA	2	High CD163	upper quartile	AA
22	scZ	54	F	LAD	2	High CD163	upper quartile	AA

* Category 1 reflects normal to Stary atherosclerosis stage I/II lesions with adaptive intimal thickening and early lipid and collagen accumulation in the subintimal layer. Category 2 reflects Stary stage III/IV early/intermediate atheroma lesions with increased lipid and collagen accumulation and proliferation. Category 3 reflects Stary stage V/VI advanced fibroatheroma or complex lesions with more severe lipid and collagen deposition as well as lipid core and thin media layer.

Supplemental Table 6. Differentially expressed genes between high and low CD163 SMCs (only kept those with FDR < 0.05)

Gene Symbol	SMC in High CD163	SMC in Low CD163	Means diff	Statistic	Conf.low	Conf.high	Adj_p-value
IGFBP7	139.44116	141.69585	-2.25469	-2.84997	-3.80589	-0.70349	0.00440
MGP	30.00128	31.84374	-1.84247	-2.52092	-3.27552	-0.40941	0.01176
ADIRF	79.63072	81.16092	-1.53020	-2.30623	-2.83117	-0.22923	0.02117
S100A6	61.28443	62.58050	-1.29607	-3.35403	-2.05374	-0.53839	0.00081
S100A4	14.56757	15.21733	-0.64976	-2.81334	-1.10262	-0.19691	0.00493
LGALS1	33.44968	34.01586	-0.56618	-2.78830	-0.96432	-0.16804	0.00533
MFGE8	20.48003	20.96875	-0.48872	-2.65456	-0.84970	-0.12773	0.00798
CALM2	26.64128	27.09409	-0.45282	-3.27053	-0.72429	-0.18134	0.00109
SOD3	21.62874	22.07558	-0.44684	-2.36010	-0.81807	-0.07561	0.01833
FN1	2.49978	2.73965	-0.23987	-3.57225	-0.37153	-0.10821	0.00036

IGFBP6	4.92908	5.15730	-0.22821	-2.45796	-0.41026	-0.04616	0.01403
ITGB1	9.97847	10.20245	-0.22398	-2.98116	-0.37130	-0.07667	0.00289
ID3	7.43063	7.65143	-0.22080	-1.97089	-0.44046	-0.00114	0.04883
IGFBP2	3.07411	3.29049	-0.21638	-3.26189	-0.34645	-0.08631	0.00112
CD151	12.24972	12.44983	-0.20011	-2.03466	-0.39295	-0.00727	0.04197
LDHA	7.40312	7.59183	-0.18871	-3.24225	-0.30283	-0.07459	0.00120
GAS6	4.95002	5.12837	-0.17835	-2.50401	-0.31801	-0.03869	0.01233
AEBP1	5.94686	6.12434	-0.17748	-2.28672	-0.32966	-0.02530	0.02228
S100A11	7.94962	8.12666	-0.17705	-2.44313	-0.31913	-0.03496	0.01462
CRIP2	8.66308	8.83605	-0.17296	-2.05844	-0.33772	-0.00821	0.03963
VCAN	0.78565	0.92569	-0.14005	-3.72796	-0.21370	-0.06639	0.00020
CLIC1	4.76070	4.90066	-0.13996	-3.22885	-0.22495	-0.05497	0.00126
PLS3	6.86418	7.00182	-0.13763	-2.44033	-0.24822	-0.02705	0.01473
ELN	2.47925	2.61166	-0.13241	-3.01569	-0.21850	-0.04632	0.00259
NUPR1	5.76135	5.88827	-0.12692	-2.52904	-0.22533	-0.02852	0.01149
COL1A2	2.24983	2.37569	-0.12586	-2.36169	-0.23035	-0.02137	0.01825
COL6A2	4.98362	5.10444	-0.12082	-2.22573	-0.22725	-0.01438	0.02611
DKK3	3.01750	3.13083	-0.11332	-2.30852	-0.20957	-0.01707	0.02104
PRELP	3.18169	3.29415	-0.11246	-2.09192	-0.21786	-0.00705	0.03653
LMNA	7.51821	7.63001	-0.11180	-2.03712	-0.21940	-0.00419	0.04173
TUBB	5.34794	5.45919	-0.11125	-2.21607	-0.20968	-0.01282	0.02676
RHOA	5.70573	5.81552	-0.10978	-2.77499	-0.18736	-0.03221	0.00555
PPIB	2.68783	2.78958	-0.10175	-2.74346	-0.17448	-0.02903	0.00612
AQP1	1.95585	2.05509	-0.09923	-3.22017	-0.15966	-0.03881	0.00129
TMA7	5.81435	5.91009	-0.09574	-2.35101	-0.17559	-0.01589	0.01879
SLC25A3	5.91563	6.01115	-0.09552	-2.54875	-0.16900	-0.02204	0.01086
HSP90B1	3.79414	3.88931	-0.09517	-2.09937	-0.18406	-0.00628	0.03587
CRIP1	4.18551	4.27908	-0.09356	-1.99795	-0.18538	-0.00174	0.04581
PRSS23	1.13995	1.23236	-0.09240	-3.16499	-0.14965	-0.03516	0.00157
COL6A1	3.11444	3.20039	-0.08595	-2.41251	-0.15581	-0.01610	0.01590
NDFIP1	2.21240	2.29767	-0.08527	-3.52895	-0.13264	-0.03789	0.00042
NPC2	1.64182	1.72606	-0.08423	-2.43029	-0.15219	-0.01627	0.01514

EFEMP1	1.69611	1.77908	-0.08297	-2.36478	-0.15176	-0.01418	0.01810
ATP6AP2	2.68323	2.76473	-0.08149	-3.31839	-0.12965	-0.03334	0.00092
ESD	3.66597	3.74081	-0.07484	-2.39093	-0.13621	-0.01346	0.01687
MAGED2	3.27994	3.35467	-0.07473	-2.33187	-0.13756	-0.01189	0.01977
CAV2	3.53513	3.60787	-0.07274	-2.00207	-0.14398	-0.00150	0.04537
SEC62	4.14794	4.21685	-0.06891	-2.10054	-0.13324	-0.00459	0.03576
ATRAID	2.00197	2.06802	-0.06605	-2.74307	-0.11327	-0.01884	0.00612
THBS2	0.92963	0.99552	-0.06588	-2.68381	-0.11402	-0.01775	0.00732
TMEM30A	0.75481	0.82042	-0.06561	-5.00861	-0.09129	-0.03992	0.00000
PSMB6	3.07012	3.13546	-0.06534	-2.34611	-0.11995	-0.01073	0.01903
SYNGR2	1.37381	1.43800	-0.06420	-3.25640	-0.10285	-0.02554	0.00114
TMED10	1.51981	1.58325	-0.06344	-3.13200	-0.10316	-0.02372	0.00175
RWDD1	2.44982	2.51306	-0.06324	-2.89824	-0.10602	-0.02046	0.00378
OAT	1.62786	1.68992	-0.06207	-3.19864	-0.10011	-0.02402	0.00140
COL1A1	1.15972	1.22136	-0.06164	-2.25371	-0.11527	-0.00801	0.02429
LTBP4	1.80797	1.86899	-0.06102	-1.96393	-0.12194	-0.00010	0.04963
CTHRC1	0.57084	0.63175	-0.06091	-3.37909	-0.09626	-0.02557	0.00074
PLD3	1.45923	1.52005	-0.06081	-3.16486	-0.09849	-0.02314	0.00157
YBX3	2.65665	2.71490	-0.05825	-2.00548	-0.11520	-0.00130	0.04500
PCBP1	2.08135	2.13946	-0.05812	-2.76100	-0.09939	-0.01684	0.00580
PKIG	2.93162	2.98932	-0.05770	-2.03623	-0.11326	-0.00214	0.04181
BUB3	0.61363	0.67059	-0.05696	-5.13336	-0.07872	-0.03520	0.00000
ATP6V1G1	3.43668	3.49352	-0.05684	-2.04457	-0.11135	-0.00233	0.04098
NDN	1.82399	1.87987	-0.05588	-2.46412	-0.10034	-0.01141	0.01379
SF3B6	2.03069	2.08595	-0.05526	-2.25666	-0.10327	-0.00725	0.02410
C19orf53	2.41321	2.46634	-0.05313	-2.24151	-0.09961	-0.00665	0.02507
COL4A2	0.92285	0.97502	-0.05217	-3.20563	-0.08408	-0.02026	0.00136
UBE2N	1.57245	1.62279	-0.05034	-2.77120	-0.08596	-0.01472	0.00562
TGM2	1.17503	1.22458	-0.04955	-2.19064	-0.09390	-0.00520	0.02855
LRRC32	0.54233	0.58998	-0.04766	-3.86993	-0.07181	-0.02351	0.00011
CUTA	2.10000	2.14716	-0.04716	-2.30617	-0.08725	-0.00706	0.02117
RNF187	0.71273	0.75977	-0.04704	-3.50565	-0.07335	-0.02073	0.00046

RAB1A	1.84694	1.89342	-0.04647	-2.28718	-0.08631	-0.00663	0.02225
SCP2	1.56177	1.60762	-0.04586	-2.56624	-0.08089	-0.01082	0.01033
TSPYL1	0.99007	1.03432	-0.04425	-2.81192	-0.07511	-0.01340	0.00496
EAPP	0.87895	0.92316	-0.04421	-3.43895	-0.06942	-0.01900	0.00059
EDIL3	0.57200	0.61556	-0.04357	-3.76361	-0.06626	-0.02087	0.00017
TERF2IP	1.62373	1.66717	-0.04343	-2.34263	-0.07978	-0.00708	0.01921
COL8A1	0.96084	1.00421	-0.04337	-2.18026	-0.08237	-0.00437	0.02931
SUN2	1.57956	1.62286	-0.04330	-2.25278	-0.08099	-0.00561	0.02434
REEP5	2.01645	2.05934	-0.04289	-2.16328	-0.08176	-0.00402	0.03060
GPAA1	0.91075	0.95323	-0.04248	-3.27332	-0.06792	-0.01703	0.00107
EMD	0.90922	0.95119	-0.04196	-2.66000	-0.07289	-0.01103	0.00786
RNASEH2C	1.47390	1.51482	-0.04092	-2.24340	-0.07668	-0.00516	0.02494
INMT	1.00201	1.04257	-0.04056	-2.24172	-0.07604	-0.00508	0.02505
EFEMP2	1.22080	1.26074	-0.03994	-2.42800	-0.07220	-0.00769	0.01524
KRT8	0.93922	0.97852	-0.03929	-2.10762	-0.07585	-0.00274	0.03515
C1orf123	1.00526	1.04431	-0.03905	-2.93419	-0.06515	-0.01296	0.00337
TNFRSF11B	0.46605	0.50481	-0.03875	-2.19536	-0.07336	-0.00414	0.02821
TFG	1.00424	1.04290	-0.03866	-2.39194	-0.07035	-0.00697	0.01682
CMPK1	1.56650	1.60442	-0.03792	-2.05661	-0.07407	-0.00177	0.03981
LRP1	0.51338	0.55107	-0.03769	-2.64940	-0.06558	-0.00980	0.00811
PDGFRB	0.80636	0.84387	-0.03751	-2.35065	-0.06879	-0.00622	0.01880
NDUFS7	1.27704	1.31451	-0.03748	-2.61011	-0.06563	-0.00932	0.00910
ITGA1	0.55663	0.59347	-0.03683	-3.26044	-0.05898	-0.01468	0.00112
PCOLCE	0.73963	0.77616	-0.03653	-1.97319	-0.07283	-0.00023	0.04856
CTSA	0.88875	0.92444	-0.03569	-2.61640	-0.06244	-0.00894	0.00893
CYBA	0.82904	0.86466	-0.03562	-2.27930	-0.06627	-0.00498	0.02272
MANF	0.90350	0.93873	-0.03523	-2.68522	-0.06096	-0.00951	0.00729
CNIH1	0.94352	0.97871	-0.03519	-2.39514	-0.06400	-0.00638	0.01667
REXO2	1.27658	1.31136	-0.03479	-2.15883	-0.06638	-0.00319	0.03094
LTBP2	0.34802	0.38273	-0.03471	-2.36008	-0.06355	-0.00587	0.01833
ERP29	1.24604	1.28029	-0.03425	-2.15430	-0.06543	-0.00308	0.03130
PMM1	0.48525	0.51875	-0.03350	-4.05122	-0.04971	-0.01728	0.00005

ERLEC1	0.85665	0.89001	-0.03336	-2.52682	-0.05926	-0.00747	0.01156
SOD2	0.62233	0.65569	-0.03336	-2.82181	-0.05654	-0.01018	0.00481
MMP2	0.28814	0.32104	-0.03290	-2.63328	-0.05741	-0.00840	0.00850
MICAL2	0.43964	0.47245	-0.03281	-2.87344	-0.05519	-0.01042	0.00409
KRT17	0.09938	0.13162	-0.03225	-3.12395	-0.05249	-0.01201	0.00180
EPB41L4A-AS1	0.89203	0.92394	-0.03191	-2.05909	-0.06230	-0.00152	0.03957
POLR2K	1.67843	1.71033	-0.03191	-1.96622	-0.06373	-0.00009	0.04937
PTTG1IP	1.00619	1.03768	-0.03149	-2.19912	-0.05956	-0.00341	0.02794
DHRS7	1.34792	1.37922	-0.03131	-2.15250	-0.05982	-0.00279	0.03144
HMG20B	0.71126	0.74249	-0.03123	-2.93725	-0.05207	-0.01038	0.00334
DCTN6	0.99083	1.02174	-0.03090	-2.33188	-0.05689	-0.00492	0.01977
SCARB2	0.61285	0.64337	-0.03051	-2.81720	-0.05175	-0.00928	0.00488
OPTN	1.14918	1.17956	-0.03039	-1.99995	-0.06018	-0.00060	0.04559
MEAF6	1.29346	1.32359	-0.03013	-2.05264	-0.05892	-0.00135	0.04019
OMD	0.22547	0.25541	-0.02993	-2.62461	-0.05229	-0.00757	0.00872
TUBA1C	0.78320	0.81308	-0.02988	-2.53961	-0.05295	-0.00681	0.01115
CERS2	0.64728	0.67646	-0.02918	-2.69066	-0.05045	-0.00792	0.00717
MSC	0.66217	0.69116	-0.02898	-1.99322	-0.05750	-0.00047	0.04633
KLC1	0.86175	0.89061	-0.02886	-2.53292	-0.05120	-0.00652	0.01136
ATPAF1	0.51237	0.54078	-0.02841	-3.36545	-0.04496	-0.01186	0.00077
C1QBP	1.18066	1.20897	-0.02831	-2.04743	-0.05542	-0.00120	0.04070
MXRA8	0.70028	0.72846	-0.02818	-1.96105	-0.05635	0.00000	0.04997
TSEN34	0.78177	0.80992	-0.02815	-2.55225	-0.04977	-0.00652	0.01075
TMEM204	0.48798	0.51576	-0.02779	-2.56929	-0.04899	-0.00658	0.01024
HNMT	0.74326	0.77104	-0.02778	-2.15939	-0.05300	-0.00256	0.03090
CPNE3	0.76427	0.79199	-0.02771	-2.23711	-0.05201	-0.00342	0.02535
NBR1	0.49530	0.52256	-0.02726	-3.12775	-0.04434	-0.01017	0.00178
DHX15	0.39023	0.41745	-0.02722	-3.46679	-0.04261	-0.01182	0.00053
EPM2AIP1	0.33737	0.36447	-0.02710	-3.41989	-0.04264	-0.01156	0.00063
RUVBL2	0.70293	0.72996	-0.02703	-2.45195	-0.04865	-0.00542	0.01426
CD82	0.39342	0.42032	-0.02690	-2.90575	-0.04505	-0.00875	0.00369
ARL6IP1	1.06625	1.09310	-0.02685	-1.96310	-0.05367	-0.00003	0.04973

MYO1D	0.32106	0.34781	-0.02675	-3.49598	-0.04175	-0.01175	0.00048
RIC8A	0.53599	0.56256	-0.02657	-3.30952	-0.04230	-0.01083	0.00095
PHACTR2	0.58466	0.61107	-0.02642	-2.46287	-0.04744	-0.00539	0.01384
FGFR1OP2	0.71380	0.74011	-0.02631	-1.99337	-0.05220	-0.00043	0.04631
FBLN2	0.15059	0.17650	-0.02591	-2.40699	-0.04702	-0.00480	0.01614
GLUD1	0.71325	0.73910	-0.02585	-2.49766	-0.04615	-0.00556	0.01255
FGF1	0.28066	0.30650	-0.02585	-3.51090	-0.04028	-0.01141	0.00045
SMIM14	0.52988	0.55557	-0.02570	-2.76977	-0.04389	-0.00751	0.00564
SMIM4	0.61577	0.64142	-0.02566	-2.77723	-0.04377	-0.00754	0.00552
VCAM1	0.31120	0.33683	-0.02563	-2.43218	-0.04629	-0.00497	0.01507
ADIPOR1	0.74530	0.77080	-0.02550	-2.01758	-0.05028	-0.00072	0.04372
NARS	0.85888	0.88437	-0.02548	-2.13301	-0.04890	-0.00206	0.03300
YWHAG	0.31575	0.34107	-0.02532	-3.17782	-0.04095	-0.00970	0.00150
PEX19	0.36526	0.39057	-0.02532	-3.27279	-0.04049	-0.01015	0.00108
SEC11C	0.63818	0.66347	-0.02529	-2.41716	-0.04581	-0.00478	0.01570
F2R	0.08955	0.11477	-0.02521	-3.04465	-0.04145	-0.00898	0.00235
EDEM2	0.13442	0.15960	-0.02518	-4.19221	-0.03695	-0.01340	0.00003
SF3B1	0.98656	1.01164	-0.02508	-2.02617	-0.04935	-0.00081	0.04284
TSG101	0.81601	0.84106	-0.02504	-2.34524	-0.04598	-0.00411	0.01908
6-Mar	0.73503	0.75996	-0.02493	-2.04206	-0.04886	-0.00099	0.04123
STOML1	0.19450	0.21933	-0.02483	-4.24715	-0.03629	-0.01337	0.00002
TMX2	0.50361	0.52817	-0.02456	-2.65149	-0.04272	-0.00640	0.00806
GLT8D1	0.28741	0.31168	-0.02427	-3.75741	-0.03694	-0.01161	0.00017
PLXDC2	0.25309	0.27724	-0.02416	-2.48511	-0.04322	-0.00510	0.01300
TSNAX	0.43954	0.46345	-0.02391	-2.80866	-0.04061	-0.00722	0.00501
CSPG4	0.33314	0.35679	-0.02364	-2.80497	-0.04017	-0.00712	0.00506
GAMT	0.57364	0.59714	-0.02350	-2.62610	-0.04104	-0.00595	0.00868
DST	0.84930	0.87276	-0.02346	-1.97071	-0.04680	-0.00012	0.04885
ISLR	0.29232	0.31537	-0.02305	-1.98409	-0.04584	-0.00027	0.04734
ACSL3	0.43792	0.46086	-0.02294	-2.88835	-0.03851	-0.00737	0.00390
SRSF8	0.79922	0.82204	-0.02282	-2.15645	-0.04357	-0.00207	0.03113
NRDC	0.40617	0.42880	-0.02263	-2.47940	-0.04053	-0.00473	0.01321

PTEN	0.46310	0.48573	-0.02263	-2.51532	-0.04026	-0.00499	0.01194
RAB5B	0.36753	0.39012	-0.02259	-2.91213	-0.03780	-0.00738	0.00362
ACADM	0.57889	0.60131	-0.02242	-2.21467	-0.04227	-0.00257	0.02686
NONO	0.68568	0.70804	-0.02236	-2.23608	-0.04196	-0.00275	0.02542
PRKDC	0.21803	0.24009	-0.02206	-3.41226	-0.03474	-0.00938	0.00065
PPP1R11	0.46871	0.49073	-0.02201	-2.74165	-0.03776	-0.00627	0.00615
ALG14	0.17475	0.19658	-0.02183	-4.12131	-0.03222	-0.01145	0.00004
SLC22A3	0.58334	0.60514	-0.02180	-2.05477	-0.04261	-0.00100	0.03999
BCL3	0.16740	0.18900	-0.02161	-3.58296	-0.03343	-0.00978	0.00035
TBXA2R	0.36182	0.38332	-0.02150	-2.40446	-0.03902	-0.00397	0.01626
CRBN	0.47974	0.50122	-0.02149	-2.37485	-0.03922	-0.00375	0.01762
G6PD	0.46632	0.48754	-0.02122	-2.32191	-0.03915	-0.00330	0.02030
YIPF5	0.40968	0.43075	-0.02107	-2.83437	-0.03565	-0.00650	0.00462
TXNDC16	0.13809	0.15913	-0.02104	-4.13532	-0.03102	-0.01106	0.00004
COX11	0.59643	0.61736	-0.02092	-2.29274	-0.03882	-0.00303	0.02193
GPC1	0.45128	0.47219	-0.02090	-2.29138	-0.03879	-0.00302	0.02201
ISG15	0.34598	0.36668	-0.02070	-2.27497	-0.03854	-0.00286	0.02298
COPS5	0.51310	0.53377	-0.02067	-2.10682	-0.03992	-0.00143	0.03521
RBM6	0.42124	0.44182	-0.02058	-2.44487	-0.03709	-0.00408	0.01455
ANKRD29	0.21837	0.23893	-0.02056	-3.49222	-0.03211	-0.00902	0.00049
ALCAM	0.22995	0.25042	-0.02047	-3.03086	-0.03372	-0.00723	0.00246
LYPLA2	0.24244	0.26260	-0.02016	-3.26542	-0.03226	-0.00805	0.00111
BFAR	0.34059	0.36068	-0.02009	-2.93259	-0.03353	-0.00666	0.00339
SP100	0.54785	0.56791	-0.02006	-2.17921	-0.03810	-0.00201	0.02939
LMF2	0.37564	0.39562	-0.01999	-2.47629	-0.03581	-0.00416	0.01333
SERGEF	0.47029	0.49024	-0.01995	-2.48179	-0.03572	-0.00419	0.01313
NUMA1	0.21547	0.23541	-0.01994	-3.07396	-0.03265	-0.00722	0.00213
SLC39A13	0.35980	0.37965	-0.01985	-2.75722	-0.03396	-0.00573	0.00586
UQCC2	0.55200	0.57183	-0.01983	-2.06482	-0.03866	-0.00100	0.03903
SFXN3	0.19895	0.21858	-0.01963	-4.28008	-0.02862	-0.01064	0.00002
AZIN1	0.32092	0.34051	-0.01958	-2.94393	-0.03263	-0.00654	0.00327
ACAD9	0.27487	0.29430	-0.01943	-3.48502	-0.03036	-0.00850	0.00050

QKI	0.27792	0.29734	-0.01943	-3.27774	-0.03105	-0.00781	0.00106
ARHGAP1	0.42538	0.44477	-0.01939	-2.12784	-0.03726	-0.00152	0.03343
STK3	0.32278	0.34211	-0.01933	-2.53294	-0.03429	-0.00437	0.01136
SULF1	0.19913	0.21846	-0.01933	-2.73046	-0.03320	-0.00545	0.00636
ZNF622	0.30502	0.32432	-0.01930	-3.02681	-0.03180	-0.00680	0.00249
SEC22C	0.38764	0.40690	-0.01926	-2.62792	-0.03363	-0.00489	0.00863
SERTAD3	0.47194	0.49116	-0.01923	-1.99350	-0.03814	-0.00032	0.04630
NFYC	0.34758	0.36681	-0.01922	-2.33695	-0.03535	-0.00309	0.01951
R3HCC1	0.59148	0.61063	-0.01915	-2.13729	-0.03672	-0.00158	0.03265
RBCK1	0.46860	0.48769	-0.01908	-2.12651	-0.03668	-0.00149	0.03354
KRT7	0.10124	0.12030	-0.01906	-2.84074	-0.03221	-0.00590	0.00453
AGA	0.15857	0.17756	-0.01899	-3.27902	-0.03034	-0.00763	0.00105
MMP23B	0.14085	0.15982	-0.01898	-2.82816	-0.03213	-0.00582	0.00471
KIAA1671	0.20870	0.22768	-0.01897	-3.83507	-0.02867	-0.00927	0.00013
ATF6B	0.45073	0.46962	-0.01889	-2.36309	-0.03456	-0.00322	0.01819
ZFYVE16	0.14599	0.16471	-0.01872	-4.10017	-0.02767	-0.00977	0.00004
TMCO3	0.51064	0.52922	-0.01858	-2.05781	-0.03629	-0.00088	0.03969
ATL3	0.32998	0.34844	-0.01845	-2.55925	-0.03259	-0.00431	0.01054
CARD19	0.56580	0.58417	-0.01838	-2.05081	-0.03594	-0.00081	0.04037
SCO2	0.13030	0.14867	-0.01837	-3.33524	-0.02917	-0.00757	0.00086
CIZ1	0.22016	0.23853	-0.01837	-3.47630	-0.02872	-0.00801	0.00052
SPPL2A	0.36427	0.38263	-0.01836	-2.57247	-0.03235	-0.00437	0.01014
MITD1	0.20578	0.22408	-0.01830	-3.39700	-0.02886	-0.00774	0.00069
GALM	0.21937	0.23763	-0.01826	-3.54365	-0.02837	-0.00816	0.00040
SNRPA	0.41122	0.42946	-0.01824	-2.22549	-0.03431	-0.00217	0.02612
ABHD14A	0.20092	0.21909	-0.01817	-3.67210	-0.02787	-0.00847	0.00024
RND3	0.24924	0.26732	-0.01808	-2.76910	-0.03088	-0.00528	0.00566
VPS13A	0.22549	0.24356	-0.01807	-2.75181	-0.03094	-0.00519	0.00596
CNOT4	0.50521	0.52322	-0.01801	-2.01904	-0.03550	-0.00052	0.04357
CNPY3	0.30375	0.32176	-0.01801	-2.80356	-0.03060	-0.00541	0.00509
ITGAV	0.21388	0.23188	-0.01800	-3.02050	-0.02968	-0.00631	0.00254
RBM4	0.20177	0.21975	-0.01798	-3.29524	-0.02868	-0.00728	0.00099

KHSRP	0.18579	0.20366	-0.01787	-2.82886	-0.03025	-0.00548	0.00470
CNPPD1	0.33218	0.34992	-0.01773	-2.40338	-0.03220	-0.00327	0.01630
OARD1	0.31150	0.32921	-0.01771	-2.76786	-0.03026	-0.00516	0.00568
TEAD2	0.37296	0.39062	-0.01766	-2.36234	-0.03232	-0.00300	0.01822
RANBP3	0.33990	0.35750	-0.01760	-2.45274	-0.03167	-0.00353	0.01423
MRPL14	0.48663	0.50421	-0.01758	-2.01578	-0.03469	-0.00048	0.04391
TNFAIP8	0.25106	0.26853	-0.01747	-2.74088	-0.02997	-0.00497	0.00616
USP33	0.23274	0.24998	-0.01724	-3.24450	-0.02766	-0.00682	0.00119
ANKH	0.24769	0.26484	-0.01715	-2.49175	-0.03065	-0.00366	0.01276
MEGF6	0.05551	0.07265	-0.01714	-4.25471	-0.02503	-0.00924	0.00002
NT5DC1	0.55676	0.57385	-0.01709	-1.98342	-0.03399	-0.00020	0.04741
SPATS2L	0.52719	0.54418	-0.01699	-2.14486	-0.03253	-0.00146	0.03204
GNB1	0.36321	0.38019	-0.01697	-1.98160	-0.03377	-0.00018	0.04761
GMPPB	0.21853	0.23545	-0.01692	-2.77355	-0.02888	-0.00496	0.00558
ITGBL1	0.12640	0.14313	-0.01673	-2.35477	-0.03066	-0.00280	0.01860
PLEKHA1	0.31914	0.33585	-0.01671	-2.10518	-0.03228	-0.00115	0.03536
CD68	0.14423	0.16089	-0.01666	-2.96476	-0.02768	-0.00564	0.00305
CALCOCO1	0.42935	0.44595	-0.01660	-2.15097	-0.03173	-0.00147	0.03156
GLB1	0.24825	0.26481	-0.01656	-3.16580	-0.02682	-0.00630	0.00156
SLC17A5	0.22639	0.24295	-0.01655	-2.65271	-0.02879	-0.00432	0.00803
STT3A	0.27710	0.29357	-0.01647	-2.66233	-0.02860	-0.00434	0.00780
PDGFD	0.11879	0.13490	-0.01611	-2.34037	-0.02961	-0.00261	0.01933
NCOR2	0.15264	0.16875	-0.01611	-3.31992	-0.02562	-0.00659	0.00091
CCDC88A	0.21952	0.23561	-0.01609	-2.46255	-0.02891	-0.00328	0.01385
WDR20	0.19205	0.20810	-0.01605	-3.13994	-0.02607	-0.00603	0.00171
ELL2	0.25105	0.26707	-0.01602	-2.36980	-0.02928	-0.00277	0.01786
MMAB	0.33139	0.34740	-0.01601	-2.39939	-0.02909	-0.00293	0.01648
SLC33A1	0.17312	0.18910	-0.01598	-3.50418	-0.02492	-0.00704	0.00046
MICU2	0.30685	0.32280	-0.01596	-2.18628	-0.03026	-0.00165	0.02887
LGR6	0.40805	0.42396	-0.01590	-2.12593	-0.03057	-0.00124	0.03359
MSTO1	0.10769	0.12355	-0.01586	-3.82877	-0.02398	-0.00774	0.00013
DCAF11	0.38634	0.40203	-0.01569	-2.01792	-0.03093	-0.00044	0.04369

GATC	0.09753	0.11313	-0.01560	-3.88616	-0.02348	-0.00773	0.00010
GTF2B	0.43960	0.45508	-0.01548	-1.97453	-0.03084	-0.00011	0.04841
CPSF6	0.27435	0.28976	-0.01541	-2.38965	-0.02806	-0.00277	0.01693
POLR3E	0.14947	0.16482	-0.01535	-3.16195	-0.02487	-0.00583	0.00158
CES1	0.23337	0.24870	-0.01533	-2.10713	-0.02960	-0.00107	0.03519
GPATCH8	0.37687	0.39215	-0.01528	-1.96177	-0.03056	-0.00001	0.04988
NAA60	0.21285	0.22807	-0.01522	-2.95625	-0.02531	-0.00512	0.00314
CNRIP1	0.34296	0.35815	-0.01519	-1.97362	-0.03029	-0.00010	0.04852
PDLIM4	0.32530	0.34049	-0.01519	-2.23220	-0.02853	-0.00185	0.02567
PRKD3	0.13860	0.15379	-0.01519	-3.10688	-0.02477	-0.00560	0.00191
EXT2	0.30622	0.32140	-0.01518	-2.52273	-0.02698	-0.00338	0.01170
IMMP1L	0.21325	0.22840	-0.01515	-2.77494	-0.02586	-0.00445	0.00556
DAG1	0.17720	0.19233	-0.01513	-2.59950	-0.02654	-0.00372	0.00938
AGBL5	0.14518	0.16030	-0.01512	-3.01521	-0.02495	-0.00529	0.00259
KLC2	0.30353	0.31863	-0.01510	-2.22486	-0.02841	-0.00179	0.02616
MGAT2	0.21139	0.22641	-0.01501	-2.69733	-0.02593	-0.00410	0.00703
SGIP1	0.12560	0.14061	-0.01501	-3.39914	-0.02367	-0.00635	0.00068
DCK	0.19929	0.21422	-0.01493	-2.75000	-0.02557	-0.00428	0.00600
IFT74	0.10106	0.11588	-0.01482	-3.58956	-0.02291	-0.00672	0.00034
CPEB4	0.23056	0.24537	-0.01481	-2.12488	-0.02847	-0.00114	0.03368
ZNF362	0.13629	0.15108	-0.01479	-3.03570	-0.02434	-0.00524	0.00242
KCTD9	0.32827	0.34291	-0.01464	-2.38932	-0.02665	-0.00263	0.01694
VTA1	0.13474	0.14931	-0.01456	-3.12111	-0.02371	-0.00541	0.00182
PHF23	0.28323	0.29767	-0.01445	-2.23927	-0.02709	-0.00180	0.02521
SIGMAR1	0.28224	0.29660	-0.01435	-2.32906	-0.02644	-0.00227	0.01992
CST6	0.07015	0.08448	-0.01433	-2.94153	-0.02389	-0.00478	0.00329
MRPS9	0.34713	0.36142	-0.01429	-2.14392	-0.02736	-0.00122	0.03212
NAF1	0.15007	0.16434	-0.01427	-3.00512	-0.02357	-0.00496	0.00268
MTERF4	0.24181	0.25599	-0.01419	-2.44038	-0.02559	-0.00279	0.01473
SLC25A12	0.17613	0.19032	-0.01418	-3.04346	-0.02332	-0.00505	0.00236
PABPN1	0.25488	0.26906	-0.01418	-2.17580	-0.02696	-0.00140	0.02965
MNAT1	0.27769	0.29184	-0.01415	-2.34460	-0.02599	-0.00232	0.01911

KCTD17	0.17277	0.18689	-0.01413	-2.46491	-0.02537	-0.00289	0.01376
FAM160A2	0.20151	0.21561	-0.01411	-2.55326	-0.02494	-0.00327	0.01072
RBM26	0.17508	0.18906	-0.01398	-2.60599	-0.02450	-0.00346	0.00921
ACOX1	0.17034	0.18417	-0.01383	-2.75403	-0.02368	-0.00398	0.00592
IPO7	0.29507	0.30887	-0.01380	-2.25548	-0.02579	-0.00180	0.02417
ST6GALNAC4	0.32639	0.34017	-0.01378	-2.08060	-0.02677	-0.00079	0.03755
FAM173A	0.37602	0.38978	-0.01376	-2.01075	-0.02718	-0.00034	0.04444
MSC-AS1	0.29584	0.30958	-0.01374	-2.11160	-0.02650	-0.00098	0.03480
CLEC18A	0.05312	0.06682	-0.01370	-3.51734	-0.02134	-0.00606	0.00044
BLOC1S2	0.20972	0.22337	-0.01365	-2.21398	-0.02575	-0.00156	0.02690
MRPL19	0.31118	0.32482	-0.01363	-2.14710	-0.02608	-0.00118	0.03186
C5orf46	0.11224	0.12580	-0.01355	-2.14002	-0.02597	-0.00114	0.03243
KLC4	0.10802	0.12156	-0.01355	-3.50015	-0.02113	-0.00596	0.00047
NDRG3	0.32283	0.33638	-0.01354	-2.08204	-0.02630	-0.00079	0.03742
BEND5	0.10177	0.11528	-0.01352	-3.50411	-0.02108	-0.00595	0.00046
FAP	0.06320	0.07670	-0.01350	-2.80814	-0.02293	-0.00408	0.00501
FUBP3	0.27978	0.29327	-0.01349	-2.26131	-0.02519	-0.00179	0.02381
ATP2B4	0.27404	0.28751	-0.01347	-2.32620	-0.02481	-0.00212	0.02007
VPS45	0.25991	0.27337	-0.01346	-2.13653	-0.02582	-0.00111	0.03272
IL17RE	0.25751	0.27092	-0.01341	-2.11834	-0.02582	-0.00100	0.03423
ME2	0.22080	0.23414	-0.01333	-2.22127	-0.02510	-0.00156	0.02641
GALNT1	0.10508	0.11835	-0.01326	-2.97116	-0.02202	-0.00451	0.00299
THUMPD2	0.22276	0.23596	-0.01320	-2.00555	-0.02610	-0.00029	0.04499
UBE2H	0.28438	0.29756	-0.01318	-2.06952	-0.02566	-0.00069	0.03858
ZNF524	0.24941	0.26255	-0.01314	-2.38661	-0.02394	-0.00234	0.01707
KRT18	0.17717	0.19028	-0.01311	-2.08445	-0.02545	-0.00078	0.03720
MFSD11	0.24595	0.25905	-0.01310	-2.10845	-0.02529	-0.00092	0.03507
RRAGC	0.35102	0.36405	-0.01304	-2.05405	-0.02548	-0.00059	0.04006
HAPLN1	0.15135	0.16438	-0.01303	-2.14076	-0.02497	-0.00110	0.03237
PODNL1	0.05443	0.06740	-0.01297	-2.77769	-0.02213	-0.00381	0.00551
CYP2U1	0.09598	0.10890	-0.01292	-3.09473	-0.02110	-0.00473	0.00199
C16orf58	0.13877	0.15162	-0.01285	-2.35727	-0.02353	-0.00216	0.01847

SLC25A20	0.15524	0.16804	-0.01280	-2.75601	-0.02191	-0.00369	0.00589
OGFOD3	0.30395	0.31674	-0.01279	-1.97539	-0.02548	-0.00009	0.04831
STX17	0.16731	0.18006	-0.01275	-2.72912	-0.02191	-0.00359	0.00639
PCNA	0.25435	0.26707	-0.01271	-2.07696	-0.02472	-0.00071	0.03789
MFSD1	0.20306	0.21570	-0.01265	-2.10560	-0.02442	-0.00087	0.03532
RIT1	0.15827	0.17090	-0.01263	-3.20341	-0.02036	-0.00490	0.00137
GPR176	0.16463	0.17725	-0.01262	-2.29074	-0.02342	-0.00182	0.02205
TMEM56	0.21702	0.22952	-0.01250	-2.12742	-0.02403	-0.00098	0.03347
THUMPD3-AS1	0.30269	0.31515	-0.01246	-1.99917	-0.02468	-0.00024	0.04568
ARHGAP18	0.05829	0.07067	-0.01237	-3.48217	-0.01934	-0.00541	0.00050
IBTK	0.14114	0.15350	-0.01236	-2.76477	-0.02112	-0.00359	0.00573
SGSM3	0.24207	0.25438	-0.01231	-2.25314	-0.02303	-0.00160	0.02432
TRPT1	0.23715	0.24939	-0.01224	-2.36928	-0.02236	-0.00211	0.01789
ZNF664	0.09415	0.10631	-0.01216	-3.14693	-0.01973	-0.00458	0.00167
CCNT2	0.12753	0.13966	-0.01212	-2.59855	-0.02127	-0.00298	0.00941
LPCAT3	0.18966	0.20172	-0.01206	-2.23695	-0.02264	-0.00149	0.02536
PIGX	0.27816	0.29020	-0.01204	-2.13703	-0.02308	-0.00099	0.03268
BCL10	0.10594	0.11789	-0.01196	-2.34860	-0.02194	-0.00197	0.01891
MXRA7	0.26473	0.27669	-0.01195	-1.97680	-0.02381	-0.00010	0.04815
ANGPTL2	0.15754	0.16942	-0.01188	-2.33571	-0.02185	-0.00191	0.01957
PTBP2	0.16350	0.17535	-0.01185	-2.35265	-0.02173	-0.00197	0.01870
DHX30	0.25476	0.26661	-0.01185	-2.16334	-0.02258	-0.00111	0.03059
MPP1	0.26270	0.27454	-0.01184	-2.00751	-0.02340	-0.00028	0.04478
SEMA4C	0.18920	0.20104	-0.01183	-2.40941	-0.02146	-0.00220	0.01604
SIAH2	0.23940	0.25123	-0.01182	-2.18696	-0.02243	-0.00122	0.02882
ZFAND2A	0.18819	0.20000	-0.01181	-2.72322	-0.02031	-0.00331	0.00650
GTF3C3	0.12400	0.13581	-0.01181	-3.09711	-0.01928	-0.00433	0.00197
TMEM245	0.21379	0.22553	-0.01175	-2.03288	-0.02307	-0.00042	0.04215
C18orf21	0.12686	0.13854	-0.01168	-2.83030	-0.01977	-0.00359	0.00468
CSF1	0.12484	0.13652	-0.01168	-2.80257	-0.01984	-0.00351	0.00510
TAF13	0.16171	0.17336	-0.01165	-2.39357	-0.02120	-0.00211	0.01675
RRS1	0.24787	0.25949	-0.01162	-2.20291	-0.02197	-0.00128	0.02768

SEC23B	0.19583	0.20734	-0.01151	-2.36528	-0.02105	-0.00197	0.01808
HSD17B14	0.16962	0.18109	-0.01147	-2.14000	-0.02198	-0.00096	0.03243
ARRDC4	0.13667	0.14814	-0.01147	-2.26612	-0.02139	-0.00155	0.02351
MTG2	0.10141	0.11282	-0.01141	-2.47898	-0.02043	-0.00238	0.01323
SKI	0.13648	0.14789	-0.01140	-2.49230	-0.02038	-0.00243	0.01274
STEAP2	0.09800	0.10934	-0.01134	-2.44942	-0.02041	-0.00226	0.01436
ERCC5	0.09907	0.11040	-0.01134	-2.53117	-0.02012	-0.00255	0.01142
POLG	0.14829	0.15958	-0.01130	-2.71622	-0.01945	-0.00314	0.00664
SERINC5	0.09902	0.11029	-0.01128	-2.50224	-0.02011	-0.00244	0.01239
UBR7	0.12553	0.13672	-0.01119	-2.41798	-0.02026	-0.00212	0.01567
S1PR2	0.05153	0.06271	-0.01118	-2.91726	-0.01869	-0.00366	0.00356
INO80E	0.25618	0.26732	-0.01115	-1.98473	-0.02216	-0.00013	0.04726
GFOD2	0.12740	0.13850	-0.01110	-2.64977	-0.01931	-0.00289	0.00810
TFB1M	0.11641	0.12745	-0.01105	-2.88762	-0.01855	-0.00355	0.00391
PPP1R35	0.14972	0.16071	-0.01099	-2.47847	-0.01969	-0.00230	0.01325
DMTF1	0.12383	0.13479	-0.01095	-2.34810	-0.02010	-0.00181	0.01893
TBC1D15	0.22654	0.23745	-0.01091	-1.99056	-0.02166	-0.00016	0.04662
COL12A1	0.10198	0.11288	-0.01091	-2.73673	-0.01872	-0.00309	0.00624
SF3B3	0.17064	0.18155	-0.01091	-2.29429	-0.02022	-0.00159	0.02184
FOXC2	0.24655	0.25742	-0.01087	-1.99112	-0.02158	-0.00017	0.04656
ERGIC1	0.17558	0.18643	-0.01085	-2.06152	-0.02117	-0.00053	0.03934
CENPC	0.19260	0.20344	-0.01084	-1.99379	-0.02150	-0.00018	0.04626
OSTF1	0.17248	0.18327	-0.01080	-2.27450	-0.02010	-0.00149	0.02301
ZNF48	0.07669	0.08747	-0.01078	-3.63828	-0.01659	-0.00497	0.00028
PCDHB16	0.09906	0.10983	-0.01077	-3.20601	-0.01736	-0.00418	0.00136
CAMKK2	0.13760	0.14837	-0.01077	-2.49018	-0.01924	-0.00229	0.01282
ICE1	0.16983	0.18060	-0.01076	-2.32353	-0.01985	-0.00168	0.02022
TSC22D2	0.11512	0.12586	-0.01073	-2.74412	-0.01840	-0.00306	0.00610
SUPV3L1	0.14133	0.15194	-0.01061	-2.58085	-0.01868	-0.00255	0.00990
SH3GL1	0.13791	0.14852	-0.01061	-2.64799	-0.01847	-0.00275	0.00814
DHDDS	0.19528	0.20584	-0.01056	-2.26636	-0.01970	-0.00142	0.02350
RNF26	0.20888	0.21938	-0.01050	-2.21946	-0.01978	-0.00122	0.02653

DEPDC5	0.04456	0.05504	-0.01048	-4.03709	-0.01557	-0.00539	0.00006
NPRL2	0.20261	0.21305	-0.01045	-2.09761	-0.02021	-0.00068	0.03602
PTPRM	0.08457	0.09500	-0.01043	-2.90805	-0.01746	-0.00340	0.00366
PIGBOS1	0.17288	0.18326	-0.01038	-2.00166	-0.02055	-0.00021	0.04541
ANKRD36	0.08996	0.10031	-0.01035	-2.80099	-0.01759	-0.00310	0.00513
XIAP	0.10288	0.11322	-0.01034	-2.70379	-0.01784	-0.00284	0.00689
P4HA3	0.08828	0.09859	-0.01031	-2.32647	-0.01901	-0.00162	0.02006
G0S2	0.04816	0.05845	-0.01029	-3.39705	-0.01623	-0.00435	0.00069
TRMT6	0.19336	0.20365	-0.01028	-2.14990	-0.01966	-0.00090	0.03164
NSUN5	0.17436	0.18461	-0.01026	-2.18914	-0.01944	-0.00107	0.02866
ADAMTS8	0.14288	0.15313	-0.01025	-2.39368	-0.01865	-0.00185	0.01674
BBS10	0.14201	0.15218	-0.01017	-2.42425	-0.01840	-0.00194	0.01540
MAP3K6	0.11505	0.12520	-0.01015	-2.51784	-0.01805	-0.00225	0.01186
MED22	0.10940	0.11955	-0.01014	-2.49921	-0.01810	-0.00219	0.01250
AP5Z1	0.08635	0.09647	-0.01012	-2.69475	-0.01748	-0.00276	0.00708
CDKN2A	0.04277	0.05287	-0.01010	-2.08239	-0.01961	-0.00059	0.03739
THBS3	0.15144	0.16153	-0.01009	-1.97283	-0.02012	-0.00006	0.04861
KBTBD3	0.10853	0.11861	-0.01008	-2.76435	-0.01723	-0.00293	0.00574
CLP1	0.11104	0.12110	-0.01005	-2.48167	-0.01800	-0.00211	0.01313
PTGES3L	0.09321	0.10326	-0.01005	-2.72576	-0.01728	-0.00282	0.00645
C2orf68	0.11155	0.12158	-0.01003	-2.26693	-0.01871	-0.00136	0.02346
GIGYF2	0.21073	0.22069	-0.00997	-2.02952	-0.01959	-0.00034	0.04249
CEP126	0.10297	0.11294	-0.00996	-2.31703	-0.01840	-0.00153	0.02057
DACT1	0.07800	0.08793	-0.00993	-2.53439	-0.01762	-0.00225	0.01131
SOWAHC	0.08652	0.09641	-0.00989	-2.85137	-0.01670	-0.00309	0.00438
FOXN2	0.13788	0.14773	-0.00985	-2.20283	-0.01862	-0.00108	0.02768
HMCN1	0.04581	0.05566	-0.00985	-3.08744	-0.01610	-0.00359	0.00204
GLA	0.11822	0.12806	-0.00983	-2.37231	-0.01796	-0.00171	0.01774
PUM2	0.12476	0.13458	-0.00982	-2.46328	-0.01764	-0.00200	0.01382
TEK	0.04588	0.05570	-0.00981	-3.40741	-0.01546	-0.00417	0.00066
SYT12	0.03730	0.04708	-0.00977	-2.73250	-0.01679	-0.00276	0.00632
SPSB2	0.11061	0.12030	-0.00969	-2.35538	-0.01776	-0.00162	0.01857

BICD2	0.11659	0.12626	-0.00967	-2.41061	-0.01754	-0.00180	0.01599
FOXO4	0.07484	0.08449	-0.00966	-2.89784	-0.01620	-0.00312	0.00378
PIGU	0.13580	0.14544	-0.00965	-2.39704	-0.01754	-0.00176	0.01659
FAM216A	0.14626	0.15589	-0.00963	-2.05137	-0.01883	-0.00043	0.04032
ZNF250	0.11748	0.12706	-0.00958	-2.30432	-0.01772	-0.00143	0.02127
FAM104A	0.15083	0.16040	-0.00957	-2.18303	-0.01816	-0.00097	0.02911
NT5C3A	0.20804	0.21758	-0.00954	-1.97883	-0.01899	-0.00009	0.04793
CCDC9	0.08084	0.09037	-0.00954	-2.85439	-0.01609	-0.00299	0.00434
UNG	0.12683	0.13632	-0.00949	-2.40596	-0.01722	-0.00176	0.01619
FOXJ3	0.12830	0.13775	-0.00945	-2.22883	-0.01776	-0.00114	0.02590
ZNF781	0.11677	0.12621	-0.00944	-2.17966	-0.01793	-0.00095	0.02936
SCAF4	0.12878	0.13819	-0.00941	-1.98955	-0.01868	-0.00014	0.04673
PINX1	0.15283	0.16215	-0.00932	-2.01344	-0.01840	-0.00024	0.04416
DDX39B	0.10212	0.11141	-0.00929	-2.59025	-0.01633	-0.00226	0.00964
RNF216	0.18266	0.19191	-0.00925	-2.02033	-0.01823	-0.00027	0.04344
LMBR1L	0.17177	0.18097	-0.00919	-2.08197	-0.01785	-0.00054	0.03743
ZBTB43	0.15355	0.16272	-0.00917	-2.10529	-0.01771	-0.00063	0.03535
WIPI1	0.17018	0.17933	-0.00915	-2.08706	-0.01775	-0.00055	0.03697
IGF1R	0.08899	0.09809	-0.00910	-2.92129	-0.01521	-0.00299	0.00351
SMCR8	0.03143	0.04051	-0.00908	-3.98195	-0.01355	-0.00461	0.00007
MON1A	0.10817	0.11723	-0.00907	-2.78699	-0.01545	-0.00269	0.00535
RABIF	0.15820	0.16726	-0.00906	-2.18390	-0.01719	-0.00093	0.02905
TTI2	0.09540	0.10442	-0.00902	-2.34558	-0.01656	-0.00148	0.01906
CCDC144NL-AS1	0.09434	0.10335	-0.00900	-2.64680	-0.01567	-0.00233	0.00817
DENND4A	0.06169	0.07067	-0.00899	-3.00807	-0.01485	-0.00313	0.00265
DDX19B	0.13337	0.14233	-0.00895	-2.44571	-0.01613	-0.00177	0.01451
TRMT13	0.06302	0.07195	-0.00893	-3.34559	-0.01417	-0.00370	0.00083
ADAM10	0.13665	0.14558	-0.00893	-2.00112	-0.01768	-0.00018	0.04547
ALOX5AP	0.06846	0.07738	-0.00893	-2.34109	-0.01640	-0.00145	0.01929
FAM122B	0.08441	0.09333	-0.00892	-2.74219	-0.01530	-0.00254	0.00614
LINC01116	0.07098	0.07986	-0.00888	-2.87519	-0.01494	-0.00282	0.00407
PCCB	0.10127	0.11012	-0.00885	-2.55704	-0.01564	-0.00206	0.01061

RGS4	0.12445	0.13329	-0.00884	-2.01442	-0.01745	-0.00024	0.04405
NAB2	0.13164	0.14047	-0.00883	-1.97778	-0.01759	-0.00008	0.04804
HSCB	0.13253	0.14134	-0.00881	-2.23323	-0.01654	-0.00107	0.02561
LRCH4	0.12382	0.13259	-0.00877	-2.29654	-0.01625	-0.00128	0.02171
OSR1	0.08721	0.09598	-0.00877	-2.13732	-0.01681	-0.00072	0.03265
RP2	0.04878	0.05755	-0.00877	-3.39932	-0.01382	-0.00371	0.00068
CBLB	0.08341	0.09216	-0.00874	-2.19037	-0.01657	-0.00092	0.02857
PEX7	0.07549	0.08423	-0.00873	-2.72308	-0.01502	-0.00245	0.00650
CHM	0.08159	0.09032	-0.00873	-2.23349	-0.01639	-0.00107	0.02559
PEX11B	0.11001	0.11870	-0.00869	-2.37810	-0.01586	-0.00153	0.01746
GNPTAB	0.08637	0.09505	-0.00868	-2.61212	-0.01519	-0.00216	0.00904
ZDHHC17	0.13699	0.14566	-0.00867	-2.01842	-0.01709	-0.00025	0.04364
ZNF786	0.03783	0.04643	-0.00860	-3.50077	-0.01342	-0.00379	0.00047
GAREM1	0.04724	0.05583	-0.00859	-3.29871	-0.01370	-0.00349	0.00098
MEGF9	0.09241	0.10099	-0.00858	-2.81349	-0.01455	-0.00260	0.00493
APAF1	0.02005	0.02862	-0.00857	-3.46560	-0.01341	-0.00372	0.00054
MTR	0.08941	0.09788	-0.00848	-2.31881	-0.01564	-0.00131	0.02047
RNASEL	0.08677	0.09523	-0.00846	-2.77250	-0.01445	-0.00248	0.00560
TEX9	0.04286	0.05129	-0.00843	-2.77938	-0.01438	-0.00248	0.00548
ALDH1L1	0.08592	0.09431	-0.00839	-2.16451	-0.01599	-0.00079	0.03050
INPP5B	0.08769	0.09606	-0.00838	-2.38358	-0.01526	-0.00149	0.01721
C3orf18	0.10261	0.11098	-0.00836	-2.40430	-0.01518	-0.00154	0.01626
PRKCA	0.04042	0.04878	-0.00836	-2.98427	-0.01385	-0.00287	0.00287
CEMIP	0.01754	0.02588	-0.00834	-3.46771	-0.01305	-0.00362	0.00053
GK	0.04966	0.05798	-0.00832	-3.31872	-0.01323	-0.00340	0.00092
TTC39B	0.09658	0.10489	-0.00831	-2.55427	-0.01469	-0.00193	0.01069
SETD7	0.11922	0.12753	-0.00831	-2.03426	-0.01632	-0.00030	0.04201
TXNDC5	0.04110	0.04940	-0.00830	-3.66280	-0.01275	-0.00386	0.00025
CASK	0.08790	0.09617	-0.00827	-2.35712	-0.01515	-0.00139	0.01848
PCDHB10	0.04996	0.05822	-0.00826	-3.25887	-0.01323	-0.00329	0.00113
ATG4C	0.10538	0.11363	-0.00825	-2.36819	-0.01508	-0.00142	0.01794
YTHDF3	0.12915	0.13738	-0.00823	-2.09177	-0.01595	-0.00052	0.03654

GHDC	0.07997	0.08819	-0.00821	-2.49462	-0.01467	-0.00176	0.01266
SMARCD1	0.09757	0.10578	-0.00821	-2.19375	-0.01556	-0.00087	0.02833
GSG1L	0.06828	0.07644	-0.00817	-2.64144	-0.01423	-0.00210	0.00830
TMEM130	0.11057	0.11873	-0.00816	-2.13153	-0.01567	-0.00065	0.03313
MTMR12	0.03106	0.03922	-0.00815	-3.73682	-0.01243	-0.00388	0.00019
TXNRD2	0.12748	0.13563	-0.00815	-2.18823	-0.01545	-0.00085	0.02873
ZNF451	0.11495	0.12309	-0.00814	-2.06031	-0.01589	-0.00039	0.03945
AGO3	0.14416	0.15230	-0.00814	-2.00362	-0.01610	-0.00017	0.04520
LINC01006	0.03484	0.04297	-0.00813	-3.29130	-0.01297	-0.00329	0.00101
OTUD6B	0.07023	0.07835	-0.00813	-2.28924	-0.01508	-0.00117	0.02213
CBFA2T2	0.04282	0.05089	-0.00807	-3.02935	-0.01330	-0.00285	0.00247
ZNF720	0.07620	0.08428	-0.00807	-2.77730	-0.01377	-0.00237	0.00552
MGME1	0.10683	0.11488	-0.00806	-2.15504	-0.01539	-0.00073	0.03124
VPS18	0.09670	0.10475	-0.00805	-2.27852	-0.01497	-0.00112	0.02277
DHX38	0.13973	0.14772	-0.00799	-2.02056	-0.01575	-0.00024	0.04341
PPM1L	0.08697	0.09497	-0.00799	-2.27733	-0.01488	-0.00111	0.02284
PPIL1	0.14232	0.15025	-0.00793	-2.02157	-0.01562	-0.00024	0.04331
SOS1	0.08682	0.09473	-0.00791	-2.46152	-0.01420	-0.00161	0.01389
SYBU	0.08309	0.09099	-0.00790	-2.61875	-0.01382	-0.00199	0.00887
TRIM16	0.08475	0.09264	-0.00789	-2.52682	-0.01401	-0.00177	0.01156
MOSPD2	0.06326	0.07109	-0.00783	-2.51823	-0.01392	-0.00173	0.01185
BIVM	0.06319	0.07101	-0.00782	-2.57313	-0.01379	-0.00186	0.01013
YTHDF1	0.09094	0.09866	-0.00772	-2.10704	-0.01491	-0.00054	0.03520
SETD9	0.11981	0.12753	-0.00772	-2.04344	-0.01512	-0.00031	0.04110
FBXO30	0.09713	0.10484	-0.00771	-2.35739	-0.01412	-0.00130	0.01847
NUDT8	0.04822	0.05588	-0.00767	-2.79904	-0.01304	-0.00230	0.00516
LMO3	0.11182	0.11948	-0.00767	-2.08396	-0.01488	-0.00045	0.03725
USP42	0.09979	0.10745	-0.00767	-2.45496	-0.01379	-0.00154	0.01415
RUBCN	0.08334	0.09100	-0.00766	-2.75380	-0.01312	-0.00221	0.00593
AGFG2	0.05778	0.06543	-0.00765	-2.95986	-0.01272	-0.00258	0.00310
NOP14-AS1	0.07339	0.08103	-0.00764	-2.60566	-0.01340	-0.00189	0.00921
KAT2A	0.11493	0.12258	-0.00764	-2.21652	-0.01441	-0.00088	0.02673

CLCN7	0.08466	0.09229	-0.00763	-2.30897	-0.01411	-0.00115	0.02101
ICA1L	0.08820	0.09582	-0.00763	-2.18498	-0.01447	-0.00078	0.02897
C2CD2	0.10702	0.11463	-0.00761	-2.09650	-0.01473	-0.00049	0.03612
ZMAT1	0.09967	0.10719	-0.00752	-2.06009	-0.01467	-0.00036	0.03948
ZNRF1	0.06038	0.06789	-0.00751	-2.41665	-0.01360	-0.00142	0.01572
ADCYAP1	0.03177	0.03927	-0.00750	-3.12722	-0.01221	-0.00280	0.00178
TTC9C	0.10671	0.11421	-0.00750	-2.27046	-0.01398	-0.00102	0.02325
RPUSD2	0.09150	0.09899	-0.00749	-2.36017	-0.01371	-0.00127	0.01833
ACSM5	0.04714	0.05462	-0.00748	-2.99292	-0.01237	-0.00258	0.00279
CDK2	0.08085	0.08832	-0.00747	-2.03316	-0.01468	-0.00027	0.04212
SLC27A1	0.07181	0.07927	-0.00746	-2.24206	-0.01398	-0.00094	0.02503
PITRM1	0.08740	0.09484	-0.00745	-2.23571	-0.01398	-0.00092	0.02544
N6AMT1	0.03232	0.03971	-0.00738	-3.38602	-0.01166	-0.00311	0.00072
PGM3	0.11452	0.12187	-0.00735	-2.01085	-0.01451	-0.00018	0.04443
COA7	0.07076	0.07808	-0.00733	-2.47908	-0.01312	-0.00153	0.01323
DISP1	0.02557	0.03289	-0.00732	-3.54139	-0.01137	-0.00327	0.00040
MTHFSD	0.05307	0.06038	-0.00730	-2.59424	-0.01282	-0.00178	0.00953
ALDH18A1	0.08730	0.09459	-0.00729	-2.01190	-0.01439	-0.00019	0.04432
ZNF425	0.06688	0.07413	-0.00726	-2.56002	-0.01282	-0.00170	0.01051
FAM24B	0.03955	0.04680	-0.00725	-3.12189	-0.01181	-0.00270	0.00181
SMOC1	0.03059	0.03778	-0.00719	-2.47109	-0.01289	-0.00148	0.01352
STPG1	0.02541	0.03258	-0.00717	-3.54458	-0.01114	-0.00321	0.00040
NAV1	0.04255	0.04970	-0.00714	-2.26875	-0.01332	-0.00097	0.02335
JDP2	0.02623	0.03336	-0.00712	-3.64939	-0.01095	-0.00330	0.00027
ACOX3	0.10746	0.11457	-0.00711	-2.02946	-0.01398	-0.00024	0.04250
SNX29	0.05280	0.05989	-0.00709	-2.58819	-0.01246	-0.00172	0.00969
VPS13D	0.08686	0.09394	-0.00708	-2.32932	-0.01303	-0.00112	0.01991
FERMT3	0.04796	0.05503	-0.00707	-2.60093	-0.01240	-0.00174	0.00934
PUSL1	0.08233	0.08939	-0.00706	-2.22432	-0.01329	-0.00084	0.02620
SLC25A28	0.05633	0.06334	-0.00702	-2.79256	-0.01194	-0.00209	0.00526
TJAP1	0.09205	0.09907	-0.00702	-2.08056	-0.01363	-0.00040	0.03756
CLK2	0.03449	0.04150	-0.00701	-2.87790	-0.01178	-0.00223	0.00403

SGPL1	0.08935	0.09634	-0.00699	-2.30563	-0.01294	-0.00105	0.02120
CDC42EP2	0.06925	0.07623	-0.00698	-2.12465	-0.01343	-0.00054	0.03370
HYKK	0.05297	0.05991	-0.00694	-2.57580	-0.01223	-0.00166	0.01005
HIST1H1E	0.02279	0.02970	-0.00691	-3.45332	-0.01084	-0.00299	0.00056
ZNF345	0.06654	0.07339	-0.00685	-2.62200	-0.01198	-0.00173	0.00879
NSMAF	0.08581	0.09265	-0.00684	-2.28854	-0.01270	-0.00098	0.02217
BAK1	0.09489	0.10169	-0.00680	-1.96633	-0.01358	-0.00002	0.04935
C17orf51	0.06860	0.07538	-0.00678	-2.56389	-0.01196	-0.00159	0.01040
CREB3L1	0.03465	0.04143	-0.00677	-2.04326	-0.01327	-0.00027	0.04111
DYNC2H1	0.05350	0.06027	-0.00677	-2.23381	-0.01271	-0.00083	0.02557
PAK1	0.10873	0.11546	-0.00673	-1.98365	-0.01339	-0.00008	0.04739
FICD	0.06253	0.06925	-0.00672	-2.43547	-0.01213	-0.00131	0.01493
KANSL1L	0.03871	0.04542	-0.00671	-2.76229	-0.01148	-0.00195	0.00577
ZCCHC3	0.05505	0.06170	-0.00665	-2.28546	-0.01236	-0.00094	0.02235
TP53I3	0.05898	0.06563	-0.00664	-2.00319	-0.01315	-0.00014	0.04525
ENPP5	0.06833	0.07496	-0.00663	-2.20116	-0.01253	-0.00072	0.02780
FBXO38	0.07807	0.08466	-0.00658	-2.05361	-0.01286	-0.00030	0.04010
SBF2	0.06885	0.07540	-0.00656	-2.27625	-0.01221	-0.00091	0.02290
POLR1E	0.08004	0.08659	-0.00655	-2.04475	-0.01283	-0.00027	0.04097
ZNF616	0.01769	0.02422	-0.00653	-3.51332	-0.01018	-0.00289	0.00045
MAN2A1	0.06418	0.07071	-0.00653	-2.04509	-0.01279	-0.00027	0.04093
F2RL1	0.01757	0.02409	-0.00652	-2.78826	-0.01110	-0.00193	0.00533
AGRN	0.04721	0.05368	-0.00647	-2.66726	-0.01123	-0.00171	0.00769
MAP4K3	0.05322	0.05969	-0.00647	-2.20575	-0.01222	-0.00072	0.02748
CENPP	0.04794	0.05441	-0.00647	-2.49697	-0.01155	-0.00139	0.01258
IGIP	0.03725	0.04371	-0.00646	-2.87864	-0.01085	-0.00206	0.00402
KCTD2	0.02836	0.03480	-0.00644	-2.95027	-0.01071	-0.00216	0.00320
SIK2	0.04520	0.05163	-0.00643	-2.56141	-0.01136	-0.00151	0.01047
ZNF429	0.07601	0.08244	-0.00643	-2.03572	-0.01262	-0.00024	0.04187
CACFD1	0.02611	0.03254	-0.00643	-3.10714	-0.01048	-0.00237	0.00191
C9orf116	0.01558	0.02195	-0.00637	-3.58478	-0.00985	-0.00289	0.00034
LIG4	0.06712	0.07347	-0.00635	-2.10648	-0.01227	-0.00044	0.03525

FOXC2-AS1	0.02396	0.03031	-0.00634	-3.07570	-0.01038	-0.00230	0.00212
IFFO2	0.04722	0.05355	-0.00633	-2.82339	-0.01073	-0.00193	0.00478
SPOCK1	0.03396	0.04028	-0.00632	-2.47164	-0.01133	-0.00131	0.01350
TBC1D14	0.07619	0.08249	-0.00630	-2.04603	-0.01234	-0.00026	0.04084
MADD	0.05077	0.05706	-0.00628	-2.49277	-0.01123	-0.00134	0.01273
SRRD	0.04933	0.05552	-0.00620	-2.50605	-0.01105	-0.00135	0.01226
LAMA2	0.04902	0.05519	-0.00617	-2.09659	-0.01194	-0.00040	0.03611
RIPK4	0.01415	0.02031	-0.00616	-3.43274	-0.00968	-0.00264	0.00061
PPARA	0.08522	0.09137	-0.00616	-2.27012	-0.01148	-0.00084	0.02327
KCNJ3	0.06179	0.06794	-0.00614	-2.18945	-0.01165	-0.00064	0.02864
ATR	0.03591	0.04205	-0.00614	-2.37092	-0.01121	-0.00106	0.01781
SCYL3	0.05906	0.06519	-0.00613	-2.53337	-0.01087	-0.00138	0.01135
SLC6A9	0.04966	0.05577	-0.00611	-2.16694	-0.01164	-0.00058	0.03032
GAS8	0.06340	0.06951	-0.00611	-2.08679	-0.01185	-0.00037	0.03699
MYOC	0.03899	0.04508	-0.00610	-2.33463	-0.01122	-0.00098	0.01963
WDR27	0.01847	0.02453	-0.00606	-3.37595	-0.00958	-0.00254	0.00074
HARBI1	0.07098	0.07702	-0.00605	-1.96160	-0.01209	0.00000	0.04990
A1BG-AS1	0.06046	0.06649	-0.00603	-2.06810	-0.01175	-0.00031	0.03872
PVT1	0.01570	0.02173	-0.00603	-3.42440	-0.00948	-0.00258	0.00062
ACKR2	0.01243	0.01843	-0.00600	-3.47502	-0.00939	-0.00262	0.00052
UNC5B-AS1	0.04995	0.05590	-0.00595	-2.19383	-0.01127	-0.00063	0.02832
MASP1	0.04993	0.05588	-0.00595	-1.98023	-0.01183	-0.00006	0.04777
TARS2	0.05604	0.06198	-0.00594	-2.21664	-0.01118	-0.00069	0.02672
TMEM143	0.07735	0.08328	-0.00592	-1.98574	-0.01177	-0.00007	0.04715
ATP11B	0.04455	0.05045	-0.00590	-2.53219	-0.01048	-0.00133	0.01139
EIF1AD	0.07097	0.07677	-0.00581	-2.00090	-0.01150	-0.00012	0.04549
ZDHHC2	0.05785	0.06365	-0.00580	-2.00362	-0.01148	-0.00012	0.04520
CHIC1	0.05048	0.05628	-0.00580	-2.40839	-0.01052	-0.00108	0.01608
WDR53	0.04320	0.04897	-0.00577	-2.50696	-0.01028	-0.00126	0.01223
TDRP	0.03629	0.04206	-0.00577	-2.77201	-0.00985	-0.00169	0.00561
GZMK	0.01669	0.02241	-0.00572	-3.04989	-0.00940	-0.00204	0.00231
LINC00853	0.03315	0.03883	-0.00568	-2.48642	-0.01015	-0.00120	0.01296

ZKSCAN4	0.02926	0.03493	-0.00567	-3.01738	-0.00935	-0.00198	0.00257
ZNF552	0.05013	0.05579	-0.00566	-2.16579	-0.01079	-0.00054	0.03041
CACNA1A	0.03630	0.04196	-0.00566	-2.27257	-0.01055	-0.00078	0.02312
SLC19A2	0.02770	0.03332	-0.00562	-2.70265	-0.00969	-0.00154	0.00692
ZNF16	0.05193	0.05750	-0.00557	-2.13553	-0.01068	-0.00046	0.03280
PIWIL1	0.01326	0.01880	-0.00553	-3.35513	-0.00877	-0.00230	0.00080
FAAP100	0.07547	0.08100	-0.00553	-2.16663	-0.01054	-0.00053	0.03034
SMIM13	0.03195	0.03747	-0.00552	-2.23806	-0.01036	-0.00068	0.02529
GXYLT1	0.05289	0.05840	-0.00552	-2.19554	-0.01044	-0.00059	0.02820
CAMK2N1	0.06920	0.07470	-0.00551	-1.96928	-0.01099	-0.00002	0.04901
ZIK1	0.05273	0.05823	-0.00550	-2.22918	-0.01034	-0.00066	0.02587
ZSCAN16	0.02932	0.03481	-0.00549	-3.02300	-0.00905	-0.00193	0.00252
ZNF169	0.01533	0.02081	-0.00547	-3.07688	-0.00896	-0.00198	0.00211
TMEM86A	0.04336	0.04883	-0.00547	-2.40675	-0.00992	-0.00101	0.01615
SLC31A1	0.07146	0.07692	-0.00545	-2.01893	-0.01075	-0.00016	0.04358
CASP8	0.02771	0.03316	-0.00545	-2.23814	-0.01022	-0.00067	0.02528
CYB5R2	0.04638	0.05183	-0.00544	-2.14209	-0.01043	-0.00046	0.03227
GHRL	0.03898	0.04441	-0.00543	-2.23897	-0.01019	-0.00067	0.02523
NMNAT1	0.02333	0.02875	-0.00542	-2.98770	-0.00897	-0.00186	0.00283
MMP25-AS1	0.02847	0.03388	-0.00540	-2.64576	-0.00941	-0.00140	0.00819
ANOS1	0.02566	0.03101	-0.00535	-2.52348	-0.00951	-0.00119	0.01167
GANC	0.04419	0.04952	-0.00533	-2.24561	-0.00998	-0.00068	0.02480
NAT14	0.02513	0.03045	-0.00533	-2.73589	-0.00914	-0.00151	0.00626
LINC01550	0.02225	0.02753	-0.00528	-2.65788	-0.00918	-0.00139	0.00790
VIM-AS1	0.04014	0.04543	-0.00528	-2.66804	-0.00916	-0.00140	0.00767
MAD2L1	0.03847	0.04375	-0.00527	-2.40666	-0.00957	-0.00098	0.01616
GTF2E1	0.01898	0.02424	-0.00527	-3.47002	-0.00825	-0.00229	0.00053
LRRN4CL	0.01965	0.02490	-0.00525	-2.98340	-0.00870	-0.00180	0.00287
PLXNA1	0.04124	0.04649	-0.00525	-2.21373	-0.00990	-0.00060	0.02692
EPB42	0.04341	0.04865	-0.00524	-2.12599	-0.01008	-0.00041	0.03359
ZNF334	0.03715	0.04239	-0.00523	-2.47120	-0.00939	-0.00108	0.01352
BRPF3	0.01443	0.01964	-0.00521	-3.05321	-0.00855	-0.00186	0.00228

PLD6	0.01598	0.02118	-0.00520	-2.72986	-0.00894	-0.00147	0.00637
PHKA1	0.01206	0.01723	-0.00517	-3.83052	-0.00781	-0.00252	0.00013
NFYA	0.02180	0.02694	-0.00514	-2.45002	-0.00926	-0.00103	0.01434
AURKC	0.01220	0.01734	-0.00514	-3.36312	-0.00814	-0.00214	0.00078
ZNF790	0.02904	0.03416	-0.00512	-2.79424	-0.00871	-0.00153	0.00523
NCBP1	0.02579	0.03090	-0.00512	-2.52548	-0.00909	-0.00114	0.01160
TBC1D9	0.05043	0.05553	-0.00510	-2.35924	-0.00934	-0.00086	0.01838
NBPF15	0.03413	0.03923	-0.00510	-1.99745	-0.01011	-0.00009	0.04587
UNC13C	0.03160	0.03669	-0.00509	-2.16381	-0.00970	-0.00048	0.03056
TTF2	0.04152	0.04660	-0.00508	-2.29783	-0.00942	-0.00075	0.02164
SLC31A2	0.04549	0.05056	-0.00507	-1.96503	-0.01014	-0.00001	0.04950
ZNF79	0.04356	0.04862	-0.00506	-2.12053	-0.00974	-0.00038	0.03404
SLC27A4	0.03824	0.04326	-0.00503	-2.39110	-0.00915	-0.00090	0.01686
RAVER2	0.03951	0.04451	-0.00500	-2.12792	-0.00961	-0.00039	0.03342
ZNF627	0.03408	0.03907	-0.00499	-2.48488	-0.00894	-0.00105	0.01301
CCDC144A	0.03747	0.04246	-0.00499	-2.14597	-0.00955	-0.00043	0.03195
PIFO	0.02666	0.03163	-0.00497	-2.51297	-0.00885	-0.00109	0.01202
USP35	0.01775	0.02270	-0.00495	-2.77426	-0.00845	-0.00145	0.00557
ZNF675	0.01275	0.01770	-0.00494	-2.83215	-0.00837	-0.00152	0.00465
PAXIP1-AS2	0.02968	0.03462	-0.00494	-2.28820	-0.00917	-0.00071	0.02219
CDKN2B	0.01739	0.02231	-0.00492	-2.48491	-0.00881	-0.00104	0.01301
COL5A3	0.02092	0.02582	-0.00490	-2.42171	-0.00887	-0.00093	0.01551
MCPH1-AS1	0.03160	0.03649	-0.00488	-2.35803	-0.00895	-0.00082	0.01844
LRP2BP	0.02187	0.02673	-0.00487	-2.94273	-0.00811	-0.00162	0.00328
TMEM184C	0.04791	0.05277	-0.00486	-2.20600	-0.00918	-0.00054	0.02746
PBX4	0.01123	0.01608	-0.00486	-2.48252	-0.00869	-0.00102	0.01310
OXSM	0.02895	0.03380	-0.00485	-2.21986	-0.00913	-0.00057	0.02650
GPR21	0.03032	0.03504	-0.00472	-2.31475	-0.00872	-0.00072	0.02069
NAPA-AS1	0.03613	0.04083	-0.00470	-2.20130	-0.00889	-0.00051	0.02779
CASS4	0.03037	0.03507	-0.00470	-2.30323	-0.00870	-0.00070	0.02133
ANLN	0.03057	0.03525	-0.00467	-2.29579	-0.00867	-0.00068	0.02176
SLC26A1	0.03065	0.03529	-0.00464	-2.27831	-0.00864	-0.00065	0.02278

LINC00467	0.02211	0.02673	-0.00462	-2.47942	-0.00827	-0.00097	0.01321
ZNF684	0.02030	0.02491	-0.00462	-2.67337	-0.00800	-0.00123	0.00755
KRT86	0.02616	0.03071	-0.00455	-2.41012	-0.00825	-0.00085	0.01601
DOCK3	0.03436	0.03889	-0.00453	-2.13723	-0.00868	-0.00037	0.03266
PNPO	0.03066	0.03513	-0.00447	-2.21253	-0.00843	-0.00051	0.02700
KATNAL2	0.03669	0.04114	-0.00445	-1.98772	-0.00884	-0.00006	0.04693
LRRC75B	0.02940	0.03383	-0.00444	-2.20156	-0.00839	-0.00049	0.02777
PRR7-AS1	0.00679	0.01117	-0.00438	-3.29682	-0.00698	-0.00177	0.00099
VHL	0.02859	0.03291	-0.00431	-2.16509	-0.00822	-0.00041	0.03046
ATXN7L2	0.00804	0.01235	-0.00431	-3.13219	-0.00701	-0.00161	0.00175
WASIR2	0.00674	0.01105	-0.00431	-3.24091	-0.00691	-0.00170	0.00120
NYNRIN	0.03656	0.04086	-0.00430	-2.01309	-0.00849	-0.00011	0.04419
TMEM232	0.00731	0.01160	-0.00429	-3.19318	-0.00693	-0.00166	0.00142
FBXL19	0.03660	0.04085	-0.00425	-1.97297	-0.00848	-0.00003	0.04859
CYP3A43	0.02633	0.03058	-0.00425	-2.15893	-0.00812	-0.00039	0.03093
GABBR1	0.03636	0.04059	-0.00423	-2.13790	-0.00810	-0.00035	0.03260
SNX25	0.02346	0.02767	-0.00421	-2.66675	-0.00730	-0.00111	0.00770
IQUB	0.00829	0.01246	-0.00417	-3.04181	-0.00685	-0.00148	0.00237
HS3ST3B1	0.03190	0.03605	-0.00415	-2.00192	-0.00822	-0.00009	0.04538
PRODH	0.01062	0.01475	-0.00413	-2.99104	-0.00685	-0.00142	0.00280
LINC01091	0.01028	0.01439	-0.00411	-2.76896	-0.00702	-0.00120	0.00566
TRAF3	0.03795	0.04203	-0.00409	-2.28554	-0.00759	-0.00058	0.02235
UBE2T	0.02390	0.02798	-0.00408	-2.42811	-0.00737	-0.00078	0.01524
MTRNR2L10	0.02772	0.03179	-0.00406	-2.03388	-0.00798	-0.00015	0.04205
RNASE6	0.02532	0.02931	-0.00399	-1.99620	-0.00790	-0.00007	0.04600
FUT10	0.01952	0.02349	-0.00397	-2.38043	-0.00724	-0.00070	0.01735
MCM2	0.01629	0.02022	-0.00393	-2.85818	-0.00663	-0.00123	0.00429
FREM1	0.01615	0.02008	-0.00393	-2.25710	-0.00735	-0.00052	0.02407
ASIC4	0.01764	0.02157	-0.00392	-2.44068	-0.00707	-0.00077	0.01472
LINC01473	0.01480	0.01872	-0.00392	-2.43313	-0.00708	-0.00076	0.01503
PDE4A	0.02387	0.02774	-0.00387	-1.96724	-0.00773	-0.00001	0.04925
VSNL1	0.01389	0.01771	-0.00382	-2.14082	-0.00731	-0.00032	0.03237

OLFM2	0.00877	0.01256	-0.00379	-2.67617	-0.00657	-0.00101	0.00749
MDGA1	0.00954	0.01333	-0.00379	-2.87528	-0.00638	-0.00121	0.00406
ARHGAP26	0.02792	0.03166	-0.00374	-2.02924	-0.00736	-0.00013	0.04252
SFXN2	0.02577	0.02943	-0.00366	-2.04609	-0.00716	-0.00015	0.04083
FBXL6	0.02914	0.03279	-0.00364	-1.97039	-0.00727	-0.00002	0.04888
GNRH2	0.01225	0.01582	-0.00357	-2.17143	-0.00679	-0.00035	0.02998
NCF4	0.02129	0.02483	-0.00354	-2.10354	-0.00684	-0.00024	0.03550
MIR133A1HG	0.02263	0.02616	-0.00353	-2.01222	-0.00698	-0.00009	0.04429
ZNF782	0.00968	0.01318	-0.00351	-2.70179	-0.00605	-0.00096	0.00694
SPATA21	0.01259	0.01610	-0.00350	-2.12266	-0.00674	-0.00027	0.03386
C1RL-AS1	0.01545	0.01895	-0.00350	-2.09097	-0.00678	-0.00022	0.03661
CDCP1	0.00922	0.01264	-0.00342	-3.14610	-0.00556	-0.00129	0.00167
KSR1	0.01014	0.01353	-0.00339	-2.31964	-0.00626	-0.00052	0.02043
RBM38	0.00522	0.00849	-0.00327	-3.49797	-0.00510	-0.00144	0.00048
PRR5L	0.01047	0.01368	-0.00322	-2.21832	-0.00606	-0.00037	0.02661
OR51E2	0.01593	0.01908	-0.00315	-2.17129	-0.00600	-0.00031	0.02999
ERAP2	0.02900	0.03214	-0.00315	-1.97420	-0.00628	-0.00002	0.04845
TMEM158	0.00907	0.01212	-0.00305	-2.08284	-0.00592	-0.00018	0.03735
ALG11	0.01304	0.01608	-0.00304	-2.14201	-0.00582	-0.00026	0.03227
LPAR4	0.01063	0.01367	-0.00303	-2.09960	-0.00586	-0.00020	0.03585
TIGD4	0.00598	0.00879	-0.00281	-2.49750	-0.00502	-0.00060	0.01256
ZXDB	0.01557	0.01835	-0.00279	-2.05403	-0.00545	-0.00013	0.04006
RIN1	0.01476	0.01754	-0.00278	-2.26148	-0.00519	-0.00037	0.02380
EPPK1	0.01457	0.01734	-0.00277	-2.18853	-0.00525	-0.00029	0.02871
MRLN	0.00960	0.01235	-0.00275	-2.06895	-0.00536	-0.00014	0.03864
NLK	0.01750	0.02025	-0.00274	-1.98967	-0.00545	-0.00004	0.04672
LINC00174	0.00722	0.00997	-0.00274	-2.30576	-0.00508	-0.00041	0.02119
CTSG	0.00882	0.01152	-0.00271	-2.23393	-0.00508	-0.00033	0.02556
DLX1	0.00466	0.00736	-0.00271	-2.80329	-0.00460	-0.00081	0.00509
GREM2	0.00466	0.00736	-0.00270	-2.79623	-0.00459	-0.00081	0.00520
TTC30A	0.01372	0.01642	-0.00270	-2.11653	-0.00520	-0.00020	0.03438
FAM13A-AS1	0.00480	0.00750	-0.00270	-2.75128	-0.00462	-0.00077	0.00597

ANKRD53	0.01263	0.01528	-0.00264	-2.44781	-0.00476	-0.00053	0.01443
PCDHB6	0.01151	0.01411	-0.00260	-2.00276	-0.00514	-0.00005	0.04529
LINC00894	0.01477	0.01736	-0.00259	-1.97692	-0.00516	-0.00002	0.04814
TBX19	0.00625	0.00881	-0.00256	-2.34720	-0.00469	-0.00042	0.01898
DEPDC7	0.00529	0.00781	-0.00252	-2.57887	-0.00443	-0.00060	0.00996
FAM184A	0.01642	0.01887	-0.00245	-2.05659	-0.00478	-0.00011	0.03981
PLB1	0.00944	0.01178	-0.00234	-2.16876	-0.00446	-0.00022	0.03018
TMEM147-AS1	0.01344	0.01573	-0.00229	-2.12385	-0.00441	-0.00018	0.03376
SRP14-AS1	0.01016	0.01243	-0.00227	-2.10134	-0.00439	-0.00015	0.03569
ZNF556	0.00774	0.00999	-0.00225	-2.10585	-0.00435	-0.00016	0.03530
YJEFN3	0.01372	0.01592	-0.00220	-2.04879	-0.00431	-0.00009	0.04057
CLHC1	0.00992	0.01195	-0.00204	-2.11435	-0.00392	-0.00015	0.03457
EGFL8	0.00755	0.00955	-0.00200	-2.07747	-0.00389	-0.00011	0.03784
IL1R2	0.00456	0.00647	-0.00191	-3.00938	-0.00316	-0.00067	0.00264
SEC31B	0.00381	0.00565	-0.00184	-3.02211	-0.00303	-0.00065	0.00253
FBXL7	0.00895	0.01079	-0.00184	-2.13039	-0.00353	-0.00015	0.03322
PLA2G7	0.00587	0.00758	-0.00171	-2.06492	-0.00333	-0.00009	0.03902
ZNF492	0.00747	0.00914	-0.00167	-2.04025	-0.00328	-0.00007	0.04141
MIR193BHG	0.00825	0.00991	-0.00166	-2.13324	-0.00318	-0.00013	0.03299
EFHB	0.00676	0.00838	-0.00161	-2.16819	-0.00307	-0.00015	0.03022
TESPA1	0.00151	0.00309	-0.00159	-2.62905	-0.00277	-0.00040	0.00861
GPR173	0.00265	0.00419	-0.00154	-2.25603	-0.00288	-0.00020	0.02414
DISP2	0.00132	0.00268	-0.00136	-2.72056	-0.00234	-0.00038	0.00655
ATG9B	0.00135	0.00267	-0.00132	-2.65635	-0.00230	-0.00035	0.00794
P2RX5	0.00261	0.00389	-0.00129	-2.29069	-0.00239	-0.00019	0.02205
ADAMTS17	0.00321	0.00448	-0.00127	-2.06455	-0.00248	-0.00006	0.03905
CSF3R	0.00148	0.00274	-0.00126	-2.25048	-0.00236	-0.00016	0.02449
TIGD2	0.00143	0.00265	-0.00122	-2.50079	-0.00218	-0.00026	0.01244
CD2	0.00241	0.00361	-0.00120	-2.03590	-0.00235	-0.00004	0.04185
ZNF410	0.00250	0.00361	-0.00111	-2.31932	-0.00205	-0.00017	0.02044
TMIGD2	0.00025	0.00133	-0.00107	-2.54700	-0.00190	-0.00025	0.01091
HSD17B13	0.00240	0.00344	-0.00104	-2.63640	-0.00181	-0.00027	0.00842

ENDOU	0.00142	0.00243	-0.00101	-2.30664	-0.00187	-0.00015	0.02114
HIST1H2BE	0.00383	0.00483	-0.00100	-2.11188	-0.00194	-0.00007	0.03478
LINC01515	0.00349	0.00440	-0.00090	-2.31258	-0.00167	-0.00014	0.02081
CCDC158	0.00066	0.00155	-0.00089	-3.56567	-0.00138	-0.00040	0.00037
LINC00971	0.00234	0.00321	-0.00087	-2.37332	-0.00159	-0.00015	0.01769
SPRED3	0.00114	0.00199	-0.00086	-1.96146	-0.00172	0.00000	0.04992
PRTN3	0.00019	0.00103	-0.00084	-2.55157	-0.00148	-0.00019	0.01077
BRINP2	0.00178	0.00261	-0.00083	-2.04213	-0.00162	-0.00003	0.04123
ROPN1B	0.00178	0.00261	-0.00083	-2.04213	-0.00162	-0.00003	0.04123
MIR202HG	0.00051	0.00131	-0.00081	-2.33069	-0.00149	-0.00013	0.01984
RAPGEFL1	0.00088	0.00167	-0.00079	-2.99088	-0.00131	-0.00027	0.00280
C15orf48	0.00188	0.00266	-0.00079	-2.14713	-0.00150	-0.00007	0.03186
TNFSF8	0.00135	0.00213	-0.00078	-2.09086	-0.00152	-0.00005	0.03662
CXorf65	0.00131	0.00209	-0.00078	-2.08845	-0.00151	-0.00005	0.03684
ARHGAP11B	0.00014	0.00069	-0.00055	-3.10259	-0.00089	-0.00020	0.00194
ZNF610	0.00006	0.00040	-0.00034	-2.31215	-0.00063	-0.00005	0.02084
CACTIN-AS1	0.00010	0.00040	-0.00030	-2.61157	-0.00052	-0.00007	0.00906
RGS9BP	0.00025	0.00052	-0.00027	-2.05006	-0.00052	-0.00001	0.04044
CNIH2	0.00009	0.00032	-0.00023	-2.14299	-0.00044	-0.00002	0.03219
DAW1	0.00011	0.00033	-0.00022	-2.06065	-0.00043	-0.00001	0.03942
LINC00571	0.00003	0.00022	-0.00019	-2.24469	-0.00036	-0.00002	0.02486
GTSE1	0.00047	0.00066	-0.00019	-2.37194	-0.00035	-0.00003	0.01776
PGM5-AS1	0.00047	0.00066	-0.00019	-2.37194	-0.00035	-0.00003	0.01776
RAB40A	0.00047	0.00066	-0.00019	-2.37194	-0.00035	-0.00003	0.01776
WIF1	0.00006	0.00023	-0.00017	-1.97041	-0.00035	0.00000	0.04888
GNAS-AS1	0.00001	0.00013	-0.00011	-2.39345	-0.00020	-0.00002	0.01675
EPHB1	0.00001	0.00013	-0.00011	-2.39345	-0.00020	-0.00002	0.01675
TFR2	0.00001	0.00013	-0.00011	-2.39345	-0.00020	-0.00002	0.01675
MYL3	0.00004	0.00002	0.00002	2.02784	0.00000	0.00005	0.04266
LEFTY1	0.00011	0.00000	0.00011	2.18968	0.00001	0.00022	0.02862
KCNH4	0.00011	0.00000	0.00011	2.18968	0.00001	0.00022	0.02862
LINC00470	0.00011	0.00000	0.00011	2.18968	0.00001	0.00022	0.02862

BRINP3	0.00014	0.00000	0.00013	2.28936	0.00002	0.00025	0.02213
WNT9B	0.00014	0.00000	0.00014	1.98114	0.00000	0.00027	0.04767
PCDHGA8	0.00018	0.00002	0.00015	1.98314	0.00000	0.00031	0.04744
DAGLA	0.00018	0.00003	0.00016	1.98756	0.00000	0.00031	0.04695
SERPIND1	0.00020	0.00000	0.00020	2.01275	0.00001	0.00040	0.04423
ZMYND12	0.00029	0.00007	0.00021	2.95054	0.00007	0.00036	0.00320
SNTG2	0.00026	0.00003	0.00023	2.02905	0.00001	0.00046	0.04254
SULT1C2	0.00035	0.00012	0.00024	2.17608	0.00002	0.00045	0.02963
PCSK6	0.00025	0.00000	0.00025	2.35059	0.00004	0.00046	0.01881
TDGF1	0.00027	0.00001	0.00026	2.02074	0.00001	0.00051	0.04339
IGF2BP3	0.00035	0.00007	0.00029	2.06232	0.00001	0.00056	0.03926
PAX3	0.00043	0.00012	0.00031	2.19470	0.00003	0.00058	0.02826
HOXA-AS2	0.00048	0.00015	0.00033	2.14859	0.00003	0.00064	0.03175
ZNF69	0.00064	0.00029	0.00036	2.40870	0.00007	0.00065	0.01607
IKBKE	0.00084	0.00048	0.00036	2.13304	0.00003	0.00069	0.03300
ADAM11	0.00084	0.00048	0.00036	2.13304	0.00003	0.00069	0.03300
MATK	0.00046	0.00009	0.00037	2.18731	0.00004	0.00070	0.02880
ISM1	0.00048	0.00007	0.00041	2.44871	0.00008	0.00073	0.01439
FBXO15	0.00078	0.00030	0.00048	2.45260	0.00010	0.00087	0.01424
DCST2	0.00058	0.00009	0.00049	2.33293	0.00008	0.00090	0.01972
NPAS4	0.00106	0.00057	0.00049	2.03489	0.00002	0.00097	0.04195
VPS9D1-AS1	0.00106	0.00057	0.00049	2.03489	0.00002	0.00097	0.04195
ITGAL	0.00117	0.00065	0.00051	2.05805	0.00002	0.00100	0.03967
CCDC36	0.00117	0.00065	0.00052	2.51802	0.00012	0.00093	0.01185
HOXA3	0.00107	0.00051	0.00055	2.76830	0.00016	0.00095	0.00567
STAC2	0.00075	0.00017	0.00057	2.61580	0.00014	0.00100	0.00895
RNF125	0.00067	0.00006	0.00060	2.98105	0.00021	0.00100	0.00290
CAPN11	0.00077	0.00017	0.00060	2.78939	0.00018	0.00103	0.00531
ROPN1L	0.00067	0.00006	0.00061	2.29969	0.00009	0.00114	0.02153
LY9	0.00088	0.00024	0.00065	2.44056	0.00013	0.00116	0.01472
TRPA1	0.00160	0.00093	0.00067	2.44809	0.00013	0.00121	0.01442
BRICD5	0.00112	0.00043	0.00069	2.57177	0.00016	0.00121	0.01017

HIST1H2BC	0.00135	0.00066	0.00069	2.35585	0.00012	0.00127	0.01854
WWTR1-AS1	0.00173	0.00104	0.00069	1.99667	0.00001	0.00138	0.04595
FBN2	0.00127	0.00054	0.00073	2.06631	0.00004	0.00143	0.03888
ZMYND15	0.00146	0.00072	0.00074	2.10862	0.00005	0.00142	0.03506
USHBP1	0.00131	0.00056	0.00075	2.15745	0.00007	0.00143	0.03105
IL4I1	0.00099	0.00021	0.00078	2.02117	0.00002	0.00154	0.04335
ITGA2B	0.00092	0.00013	0.00079	2.20215	0.00009	0.00149	0.02773
GINS3	0.00195	0.00115	0.00080	2.09937	0.00005	0.00155	0.03587
PDE6B	0.00143	0.00062	0.00081	2.17787	0.00008	0.00153	0.02949
CORIN	0.00092	0.00002	0.00090	2.73913	0.00025	0.00154	0.00620
OPRK1	0.00092	0.00002	0.00090	2.73913	0.00025	0.00154	0.00620
CELSR3	0.00160	0.00069	0.00090	2.15570	0.00008	0.00173	0.03119
IL20RA	0.00195	0.00104	0.00091	2.14668	0.00008	0.00174	0.03190
IL31RA	0.00274	0.00182	0.00092	2.23069	0.00011	0.00173	0.02577
KLRD1	0.00220	0.00126	0.00094	2.01741	0.00003	0.00185	0.04374
MAB21L1	0.00115	0.00021	0.00094	2.35239	0.00016	0.00173	0.01872
VIPR1-AS1	0.00290	0.00193	0.00097	1.98359	0.00001	0.00193	0.04739
WDR17	0.00200	0.00101	0.00100	2.75593	0.00029	0.00171	0.00589
KCNK7	0.00300	0.00200	0.00100	2.11026	0.00007	0.00193	0.03492
CCL1	0.00119	0.00017	0.00102	2.42994	0.00020	0.00184	0.01516
KCNJ5	0.00110	0.00008	0.00102	2.92555	0.00034	0.00171	0.00346
EPCAM	0.00172	0.00069	0.00103	2.06408	0.00005	0.00201	0.03910
PYDC1	0.00115	0.00011	0.00104	3.01197	0.00036	0.00171	0.00262
MCOLN2	0.00262	0.00157	0.00105	1.96619	0.00000	0.00209	0.04937
DSP	0.00216	0.00111	0.00105	1.99618	0.00002	0.00208	0.04600
PRPH2	0.00295	0.00190	0.00105	1.97127	0.00001	0.00210	0.04878
NPIPA5	0.00299	0.00193	0.00106	2.18508	0.00011	0.00201	0.02896
IL20RB	0.00177	0.00071	0.00106	2.17266	0.00010	0.00203	0.02988
ANKRD2	0.00170	0.00063	0.00107	2.49626	0.00023	0.00191	0.01260
EXOC3L2	0.00250	0.00140	0.00109	1.99991	0.00002	0.00217	0.04560
XPNPEP2	0.00192	0.00077	0.00116	2.75936	0.00033	0.00198	0.00583
REEP6	0.00407	0.00291	0.00116	2.10315	0.00008	0.00224	0.03553

TTLL9	0.00141	0.00023	0.00117	3.10106	0.00043	0.00192	0.00195
BCAN	0.00352	0.00234	0.00118	2.39247	0.00021	0.00214	0.01680
NFATC2	0.00430	0.00312	0.00118	1.97889	0.00001	0.00234	0.04792
PCDH12	0.00235	0.00116	0.00119	2.46060	0.00024	0.00214	0.01393
WFIKKN2	0.00331	0.00212	0.00119	2.06113	0.00006	0.00233	0.03938
B3GNT5	0.00470	0.00348	0.00122	1.97931	0.00001	0.00242	0.04787
RAPGEF4	0.00274	0.00152	0.00122	2.06968	0.00006	0.00238	0.03857
ALDH1L1-AS1	0.00222	0.00097	0.00125	2.35691	0.00021	0.00228	0.01849
LRRIQ1	0.00317	0.00187	0.00131	2.42488	0.00025	0.00236	0.01537
CHDH	0.00220	0.00089	0.00131	2.36694	0.00022	0.00239	0.01800
LINC01465	0.00180	0.00048	0.00132	2.98476	0.00045	0.00219	0.00286
LINC00504	0.00429	0.00296	0.00133	2.03455	0.00005	0.00260	0.04198
NAV3	0.00257	0.00122	0.00134	2.37130	0.00023	0.00245	0.01779
CTXN1	0.00468	0.00334	0.00135	2.02537	0.00004	0.00265	0.04292
PNLIPRP1	0.00587	0.00452	0.00135	2.03418	0.00005	0.00265	0.04202
RNASEH2B-AS1	0.00262	0.00126	0.00135	2.41326	0.00025	0.00245	0.01587
CRACR2A	0.00246	0.00109	0.00137	2.34671	0.00023	0.00252	0.01900
HOXB-AS3	0.00264	0.00126	0.00137	2.44735	0.00027	0.00247	0.01445
TNKS2-AS1	0.00529	0.00391	0.00138	2.00072	0.00003	0.00273	0.04551
TMEM52	0.00453	0.00313	0.00140	2.20362	0.00015	0.00264	0.02763
TSPAN18	0.00399	0.00259	0.00140	2.09942	0.00009	0.00271	0.03586
KCP	0.00371	0.00225	0.00146	2.03185	0.00005	0.00286	0.04226
FAM71F2	0.00290	0.00142	0.00148	2.92741	0.00049	0.00247	0.00344
FRAT1	0.00415	0.00266	0.00148	2.23866	0.00018	0.00278	0.02525
SYK	0.00354	0.00204	0.00150	2.05600	0.00007	0.00294	0.03987
DANT2	0.00369	0.00217	0.00152	2.07611	0.00008	0.00295	0.03797
SMIM2-AS1	0.00493	0.00340	0.00153	2.05292	0.00007	0.00299	0.04017
KIF20A	0.00733	0.00579	0.00153	2.04956	0.00007	0.00300	0.04049
DIAPH3	0.00839	0.00684	0.00154	1.96548	0.00000	0.00308	0.04945
RAB11FIP3	0.00460	0.00302	0.00158	2.38126	0.00028	0.00288	0.01731
CCT6B	0.00778	0.00619	0.00159	2.08580	0.00010	0.00308	0.03708
ITIH1	0.00758	0.00597	0.00161	2.05114	0.00007	0.00314	0.04034

TFEC	0.01025	0.00864	0.00161	2.14595	0.00014	0.00308	0.03196
LRRC61	0.00508	0.00345	0.00162	2.09292	0.00010	0.00314	0.03644
EFNA4	0.00689	0.00526	0.00163	2.26400	0.00022	0.00304	0.02364
ARHGEF19	0.00342	0.00179	0.00163	2.28380	0.00023	0.00303	0.02245
TNNT3	0.00535	0.00371	0.00163	2.00021	0.00003	0.00323	0.04557
HIST1H2AH	0.00262	0.00097	0.00165	2.75593	0.00047	0.00282	0.00589
ACAD11	0.00401	0.00234	0.00167	2.28782	0.00024	0.00310	0.02222
TUB	0.00776	0.00608	0.00168	2.24107	0.00021	0.00315	0.02509
TPX2	0.00343	0.00175	0.00168	2.41618	0.00032	0.00304	0.01574
USP3-AS1	0.00624	0.00454	0.00170	1.98174	0.00002	0.00339	0.04760
LINC01186	0.01219	0.01045	0.00173	1.97873	0.00002	0.00345	0.04794
FGF10-AS1	0.00334	0.00159	0.00175	2.75734	0.00051	0.00300	0.00586
PLEKHA7	0.00374	0.00192	0.00181	2.02054	0.00005	0.00357	0.04342
HIST1H4E	0.00721	0.00539	0.00182	1.96817	0.00001	0.00363	0.04914
TRIM55	0.00770	0.00587	0.00183	2.03737	0.00007	0.00359	0.04170
NHLRC4	0.00600	0.00417	0.00184	1.96852	0.00001	0.00367	0.04910
CFHR3	0.00557	0.00373	0.00184	2.33235	0.00029	0.00339	0.01975
CHI3L2	0.00749	0.00564	0.00185	2.46185	0.00038	0.00332	0.01388
SLC38A5	0.00880	0.00694	0.00186	1.98938	0.00003	0.00370	0.04675
VPREB3	0.00645	0.00456	0.00189	1.97255	0.00001	0.00377	0.04864
ITPKB	0.00706	0.00515	0.00190	2.17319	0.00019	0.00362	0.02984
CAPN15	0.00887	0.00695	0.00191	2.06014	0.00009	0.00374	0.03947
OXCT2	0.00562	0.00369	0.00194	2.03895	0.00007	0.00380	0.04154
TINCR	0.00563	0.00368	0.00195	3.05529	0.00070	0.00320	0.00227
ARIH2OS	0.00961	0.00759	0.00202	2.20933	0.00023	0.00382	0.02723
MIF-AS1	0.00715	0.00512	0.00203	2.39976	0.00037	0.00369	0.01647
CCNF	0.00476	0.00273	0.00203	2.96620	0.00069	0.00337	0.00304
PRICKLE4	0.00495	0.00290	0.00204	2.72177	0.00057	0.00351	0.00653
ZKSCAN8	0.00700	0.00493	0.00207	2.63561	0.00053	0.00361	0.00844
GXYLT2	0.00518	0.00310	0.00207	2.19891	0.00022	0.00392	0.02796
MBLAC1	0.01193	0.00984	0.00209	2.13256	0.00017	0.00401	0.03304
FOXP2	0.00713	0.00504	0.00210	2.06629	0.00011	0.00409	0.03889

CNIH3	0.00696	0.00485	0.00211	2.22430	0.00025	0.00397	0.02620
TUBB4A	0.00583	0.00370	0.00212	2.21984	0.00025	0.00400	0.02650
FBXO45	0.01116	0.00903	0.00213	2.05713	0.00010	0.00415	0.03976
MYO7B	0.00883	0.00666	0.00217	2.53576	0.00049	0.00385	0.01127
PCDHGA5	0.00645	0.00425	0.00220	2.14089	0.00019	0.00422	0.03236
LAMB3	0.00456	0.00234	0.00222	2.95318	0.00075	0.00369	0.00317
BEGAIN	0.00409	0.00187	0.00222	2.60128	0.00055	0.00390	0.00933
CD3D	0.00860	0.00637	0.00223	2.02676	0.00007	0.00438	0.04277
TEP1	0.00724	0.00500	0.00223	2.04982	0.00010	0.00437	0.04047
LINC01544	0.00337	0.00113	0.00223	3.24037	0.00088	0.00358	0.00121
INTS7	0.01119	0.00895	0.00224	2.19996	0.00024	0.00423	0.02789
STRBP	0.00960	0.00736	0.00225	2.08577	0.00013	0.00436	0.03708
IPMK	0.00764	0.00539	0.00225	2.42018	0.00043	0.00407	0.01557
CYP4F11	0.00822	0.00597	0.00225	2.09373	0.00014	0.00436	0.03637
ZNF763	0.00923	0.00697	0.00226	2.10695	0.00016	0.00436	0.03520
SPN	0.00337	0.00107	0.00229	2.69405	0.00062	0.00397	0.00710
CAHM	0.00835	0.00603	0.00232	2.05800	0.00011	0.00453	0.03968
ZNF81	0.00703	0.00471	0.00232	2.65083	0.00060	0.00403	0.00807
GATA5	0.00802	0.00570	0.00232	2.14194	0.00020	0.00445	0.03228
TMEM256-PLSCR3	0.00657	0.00424	0.00232	2.05520	0.00011	0.00454	0.03995
PAMR1	0.00694	0.00461	0.00233	1.99190	0.00004	0.00461	0.04647
NUDT11	0.01179	0.00941	0.00238	1.98735	0.00003	0.00473	0.04697
ZNF530	0.00791	0.00552	0.00239	2.29736	0.00035	0.00444	0.02167
P2RX7	0.01011	0.00771	0.00239	2.13841	0.00020	0.00459	0.03256
ATAD2B	0.01015	0.00773	0.00241	2.41528	0.00045	0.00437	0.01578
IGSF10	0.00741	0.00499	0.00243	2.43350	0.00047	0.00438	0.01501
F2RL3	0.00708	0.00463	0.00244	1.96531	0.00001	0.00488	0.04947
IGSF6	0.01451	0.01206	0.00244	2.06036	0.00012	0.00477	0.03945
FITM1	0.00932	0.00684	0.00248	2.97952	0.00085	0.00411	0.00291
GPHA2	0.00642	0.00393	0.00249	2.30561	0.00037	0.00460	0.02120
ZNF337-AS1	0.00705	0.00456	0.00249	2.17727	0.00025	0.00474	0.02954
WNT2B	0.01181	0.00929	0.00252	1.98249	0.00003	0.00502	0.04752

CCDC181	0.01517	0.01263	0.00255	2.28882	0.00036	0.00473	0.02216
ADM5	0.00680	0.00423	0.00257	2.46159	0.00052	0.00461	0.01389
DLEU1	0.01134	0.00876	0.00258	2.03895	0.00010	0.00505	0.04154
FSCN2	0.00864	0.00606	0.00258	2.07343	0.00014	0.00502	0.03822
HID1	0.00848	0.00589	0.00259	2.39617	0.00047	0.00471	0.01663
DLGAP1-AS2	0.01700	0.01439	0.00261	2.03393	0.00009	0.00512	0.04205
MSX1	0.00821	0.00557	0.00264	2.57736	0.00063	0.00464	0.01000
SKP2	0.01126	0.00861	0.00265	2.07353	0.00014	0.00516	0.03821
C9orf50	0.00930	0.00665	0.00265	2.28548	0.00038	0.00492	0.02235
CEP19	0.01052	0.00781	0.00271	2.49344	0.00058	0.00484	0.01270
SHC2	0.00985	0.00713	0.00272	2.13680	0.00022	0.00521	0.03269
CD247	0.01775	0.01502	0.00273	2.38023	0.00048	0.00498	0.01736
NDST2	0.02414	0.02139	0.00275	1.98328	0.00003	0.00547	0.04743
ZNF519	0.02290	0.02013	0.00277	2.00403	0.00006	0.00548	0.04516
C12orf54	0.00823	0.00544	0.00279	2.24100	0.00035	0.00523	0.02510
TSNARE1	0.01058	0.00779	0.00279	2.36291	0.00048	0.00511	0.01819
ACP5	0.01172	0.00892	0.00280	2.23127	0.00034	0.00527	0.02574
HIP1R	0.00928	0.00643	0.00285	2.63468	0.00073	0.00496	0.00846
MIATNB	0.01992	0.01706	0.00286	2.36095	0.00048	0.00524	0.01829
TBX1	0.00720	0.00434	0.00286	2.38358	0.00051	0.00522	0.01721
MPP3	0.01876	0.01590	0.00286	1.96701	0.00001	0.00572	0.04927
MCF2L	0.02406	0.02118	0.00288	2.02505	0.00009	0.00567	0.04295
DUSP5	0.00971	0.00680	0.00291	2.59878	0.00071	0.00510	0.00940
CA9	0.00767	0.00474	0.00293	2.58456	0.00071	0.00516	0.00980
KIAA0408	0.01566	0.01273	0.00294	2.03157	0.00010	0.00577	0.04228
PHOSPHO2	0.02838	0.02544	0.00294	2.07734	0.00017	0.00572	0.03785
ADPRH	0.01639	0.01343	0.00296	2.09778	0.00019	0.00572	0.03601
GPT2	0.01419	0.01121	0.00297	2.45066	0.00059	0.00535	0.01432
FBF1	0.00784	0.00484	0.00300	2.66085	0.00079	0.00521	0.00784
WBP1	0.02713	0.02411	0.00302	2.28942	0.00043	0.00561	0.02212
CD86	0.02034	0.01731	0.00303	2.21261	0.00034	0.00571	0.02700
PARD6G	0.02503	0.02199	0.00304	2.09748	0.00020	0.00588	0.03603

LINC00865	0.01086	0.00782	0.00304	2.68629	0.00082	0.00526	0.00726
NTM	0.01288	0.00982	0.00305	2.33042	0.00048	0.00562	0.01985
ATRN	0.01497	0.01191	0.00306	2.48487	0.00064	0.00547	0.01301
KIAA1211L	0.01264	0.00957	0.00306	2.71690	0.00085	0.00527	0.00663
FAM180A	0.02839	0.02530	0.00309	2.16685	0.00029	0.00588	0.03032
GNG2	0.00968	0.00659	0.00309	2.31647	0.00047	0.00570	0.02060
SYPL2	0.02230	0.01915	0.00315	2.64765	0.00082	0.00549	0.00815
TRIM68	0.02922	0.02606	0.00315	2.21357	0.00036	0.00595	0.02693
RAB38	0.01456	0.01138	0.00318	2.45985	0.00064	0.00571	0.01395
FGR	0.00965	0.00646	0.00319	2.26478	0.00043	0.00595	0.02360
ADRB2	0.01673	0.01354	0.00319	2.09760	0.00021	0.00617	0.03602
DENND2A	0.01317	0.00997	0.00319	3.39829	0.00135	0.00504	0.00069
MAG	0.00577	0.00255	0.00321	2.42466	0.00061	0.00581	0.01538
ACSM3	0.01224	0.00901	0.00323	2.15131	0.00029	0.00617	0.03153
ZNF674	0.01483	0.01159	0.00323	2.12778	0.00025	0.00621	0.03344
NAGS	0.02682	0.02358	0.00324	2.10853	0.00023	0.00625	0.03507
MTBP	0.00774	0.00448	0.00326	3.02039	0.00115	0.00538	0.00255
ZBTB33	0.01829	0.01498	0.00332	2.30781	0.00050	0.00614	0.02108
MX2	0.01668	0.01335	0.00334	2.24262	0.00042	0.00625	0.02499
SCD	0.00857	0.00523	0.00334	2.71395	0.00093	0.00575	0.00669
LINC01355	0.02585	0.02249	0.00337	2.10217	0.00023	0.00651	0.03562
PRX	0.00837	0.00499	0.00338	2.28859	0.00048	0.00628	0.02217
CHEK2	0.01188	0.00849	0.00339	2.20401	0.00037	0.00640	0.02760
KLRB1	0.00816	0.00473	0.00344	3.16907	0.00131	0.00556	0.00154
ADCY4	0.00884	0.00541	0.00344	2.76476	0.00100	0.00587	0.00573
RHBDF2	0.02566	0.02216	0.00349	2.03158	0.00012	0.00687	0.04228
MAP7	0.01674	0.01324	0.00351	2.32884	0.00055	0.00646	0.01993
MMP16	0.01630	0.01279	0.00351	2.04806	0.00015	0.00687	0.04064
PIK3R4	0.01837	0.01484	0.00353	2.03926	0.00014	0.00693	0.04151
CCL26	0.01939	0.01585	0.00354	2.06840	0.00018	0.00690	0.03869
ATAD5	0.01708	0.01354	0.00354	2.04164	0.00014	0.00695	0.04127
TLN2	0.02363	0.02009	0.00354	2.40422	0.00065	0.00644	0.01627

POLR1A	0.01889	0.01531	0.00358	3.14777	0.00135	0.00581	0.00166
NUDT17	0.02542	0.02182	0.00360	1.98963	0.00005	0.00715	0.04672
UPF1	0.02140	0.01777	0.00363	2.07061	0.00019	0.00707	0.03848
ZNF300	0.01878	0.01511	0.00367	2.75935	0.00106	0.00627	0.00583
EPHB4	0.01474	0.01108	0.00367	2.23969	0.00046	0.00688	0.02518
ZNF780A	0.02233	0.01866	0.00367	2.24122	0.00046	0.00688	0.02508
SCX	0.03157	0.02788	0.00370	2.11436	0.00027	0.00713	0.03457
ZNF318	0.03307	0.02936	0.00371	2.18469	0.00038	0.00705	0.02899
ZC3H12C	0.02597	0.02225	0.00372	2.15004	0.00033	0.00711	0.03163
AHCYL2	0.01195	0.00823	0.00372	2.56585	0.00088	0.00656	0.01034
SH3RF3	0.02095	0.01722	0.00373	2.27817	0.00052	0.00694	0.02279
WDR35	0.02925	0.02552	0.00373	2.17236	0.00036	0.00710	0.02991
ARHGEF39	0.02213	0.01840	0.00374	2.39671	0.00068	0.00679	0.01660
ACHE	0.01907	0.01531	0.00376	2.35883	0.00063	0.00688	0.01840
LINC00909	0.02966	0.02584	0.00382	2.15943	0.00035	0.00729	0.03090
ACVR2A	0.02173	0.01791	0.00382	2.22154	0.00045	0.00720	0.02639
NPIPA1	0.02361	0.01977	0.00384	2.27106	0.00053	0.00716	0.02321
IGFBP7-AS1	0.03884	0.03498	0.00386	2.06840	0.00020	0.00751	0.03869
AGAP6	0.02470	0.02083	0.00387	2.07463	0.00021	0.00753	0.03810
C14orf93	0.01230	0.00843	0.00387	3.73892	0.00184	0.00591	0.00019
KIAA0513	0.03372	0.02985	0.00388	2.20618	0.00043	0.00732	0.02745
GALNT15	0.01236	0.00846	0.00390	3.34287	0.00161	0.00618	0.00084
TMEM79	0.02528	0.02139	0.00390	2.60465	0.00096	0.00683	0.00924
PCGF3	0.02777	0.02383	0.00394	2.04361	0.00016	0.00772	0.04108
SMURF1	0.03391	0.02994	0.00396	2.09557	0.00025	0.00767	0.03620
LINC01133	0.01133	0.00730	0.00402	2.61200	0.00100	0.00705	0.00905
ANKRD26	0.03066	0.02662	0.00404	2.31411	0.00062	0.00746	0.02073
TMOD2	0.04620	0.04212	0.00408	2.03938	0.00016	0.00800	0.04150
C9orf40	0.03585	0.03174	0.00412	2.12755	0.00032	0.00791	0.03345
AP1AR	0.02687	0.02274	0.00413	2.10467	0.00028	0.00797	0.03540
CHADL	0.01002	0.00587	0.00415	2.87149	0.00132	0.00699	0.00411
LRRC34	0.02937	0.02515	0.00422	2.00908	0.00010	0.00834	0.04462

RASSF8-AS1	0.04559	0.04136	0.00423	2.07047	0.00022	0.00823	0.03849
NGEF	0.01895	0.01466	0.00429	2.55040	0.00099	0.00759	0.01081
ZNF746	0.03672	0.03235	0.00437	2.18108	0.00044	0.00829	0.02925
CDYL	0.02224	0.01787	0.00437	2.76711	0.00127	0.00747	0.00569
BAIAP2	0.02126	0.01689	0.00437	2.37609	0.00076	0.00798	0.01756
CLASP1	0.03696	0.03258	0.00438	2.34468	0.00072	0.00804	0.01911
CDA	0.01775	0.01336	0.00440	2.07453	0.00024	0.00855	0.03811
ROBO3	0.02690	0.02250	0.00440	2.02376	0.00014	0.00866	0.04308
ZXDC	0.02995	0.02553	0.00442	2.28997	0.00064	0.00820	0.02209
ZMYM3	0.04350	0.03908	0.00443	2.09859	0.00029	0.00857	0.03594
THBS4	0.01338	0.00892	0.00445	2.58788	0.00108	0.00783	0.00970
MRC1	0.02156	0.01710	0.00446	2.01171	0.00011	0.00880	0.04434
RASSF8	0.02281	0.01832	0.00449	2.59626	0.00110	0.00788	0.00947
PLPP6	0.03473	0.03023	0.00450	2.65653	0.00118	0.00782	0.00794
RIC1	0.02628	0.02177	0.00451	2.27204	0.00062	0.00840	0.02315
SMO	0.01359	0.00907	0.00453	3.62044	0.00207	0.00698	0.00030
GAD1	0.05280	0.04827	0.00453	1.96982	0.00002	0.00904	0.04895
SLC2A13	0.03111	0.02657	0.00454	2.13517	0.00037	0.00871	0.03283
SGCD	0.05121	0.04663	0.00458	2.18021	0.00046	0.00870	0.02932
TMC6	0.04036	0.03578	0.00458	2.11539	0.00033	0.00883	0.03448
GRASP	0.02010	0.01552	0.00459	2.38966	0.00082	0.00835	0.01692
CD24	0.01223	0.00764	0.00459	2.78338	0.00136	0.00782	0.00541
ASAP3	0.02419	0.01959	0.00460	2.42406	0.00088	0.00832	0.01541
UCK2	0.01713	0.01250	0.00464	2.88853	0.00149	0.00778	0.00390
NLRX1	0.03189	0.02721	0.00469	2.10449	0.00032	0.00905	0.03542
TYK2	0.03987	0.03518	0.00469	2.26843	0.00064	0.00874	0.02337
PARVB	0.03185	0.02715	0.00469	2.13130	0.00038	0.00901	0.03315
MPHOSPH9	0.04436	0.03965	0.00470	2.07042	0.00025	0.00916	0.03850
FYN	0.03244	0.02770	0.00475	2.01841	0.00014	0.00936	0.04364
PYCR1	0.01767	0.01287	0.00480	2.58847	0.00116	0.00843	0.00969
ZNF784	0.03091	0.02607	0.00484	2.52828	0.00109	0.00859	0.01151
TNFRSF1B	0.03320	0.02835	0.00486	2.39840	0.00089	0.00883	0.01653

RAB30	0.03565	0.03079	0.00486	2.26708	0.00066	0.00906	0.02346
LOXL3	0.03271	0.02785	0.00487	2.12383	0.00037	0.00936	0.03377
ZNF395	0.02718	0.02231	0.00487	2.76058	0.00141	0.00833	0.00580
CEP112	0.02651	0.02163	0.00487	2.56072	0.00114	0.00860	0.01049
PCED1A	0.02389	0.01901	0.00488	2.61298	0.00122	0.00854	0.00902
GNA13	0.04255	0.03767	0.00488	2.00587	0.00011	0.00966	0.04496
SPRED2	0.03784	0.03294	0.00490	2.37098	0.00085	0.00895	0.01780
PTAR1	0.04187	0.03696	0.00492	2.01025	0.00012	0.00971	0.04449
STRN	0.04534	0.04041	0.00493	2.35724	0.00083	0.00903	0.01847
NIPSNAP3B	0.02480	0.01981	0.00499	2.85666	0.00157	0.00842	0.00431
SLC16A9	0.04958	0.04458	0.00500	2.19616	0.00054	0.00946	0.02816
COLCA2	0.01899	0.01398	0.00501	3.27764	0.00201	0.00801	0.00106
GPM6A	0.01885	0.01380	0.00506	2.26130	0.00067	0.00944	0.02381
TCEAL5	0.06010	0.05503	0.00507	2.18799	0.00053	0.00961	0.02875
CDCA4	0.03988	0.03480	0.00507	2.12640	0.00040	0.00975	0.03355
ZFYVE28	0.02248	0.01740	0.00508	2.98040	0.00174	0.00842	0.00290
SLC9B2	0.03538	0.03027	0.00511	2.45575	0.00103	0.00918	0.01412
VIPR2	0.05257	0.04745	0.00511	2.28792	0.00073	0.00949	0.02221
DUSP16	0.02608	0.02094	0.00514	3.22742	0.00202	0.00827	0.00126
LURAP1	0.04322	0.03802	0.00519	2.65088	0.00135	0.00903	0.00807
COX15	0.03939	0.03420	0.00520	2.27433	0.00072	0.00967	0.02302
ABHD6	0.03584	0.03058	0.00526	2.53448	0.00119	0.00933	0.01131
CADM4	0.03979	0.03452	0.00526	2.11821	0.00039	0.01014	0.03424
TMEM161B	0.02481	0.01953	0.00528	2.81700	0.00161	0.00896	0.00488
CSNK1G1	0.02174	0.01645	0.00529	3.25633	0.00210	0.00847	0.00114
GAL3ST4	0.05260	0.04731	0.00529	2.16279	0.00049	0.01009	0.03064
RAPGEF1	0.02272	0.01743	0.00529	3.19881	0.00205	0.00853	0.00139
CEP170	0.04803	0.04273	0.00530	2.10455	0.00036	0.01023	0.03541
MAST2	0.05413	0.04883	0.00530	2.23032	0.00064	0.00996	0.02580
C4orf36	0.03489	0.02958	0.00531	2.30209	0.00079	0.00984	0.02140
WDTC1	0.07018	0.06486	0.00532	2.01039	0.00013	0.01052	0.04448
ZBED5-AS1	0.03780	0.03245	0.00535	2.39087	0.00096	0.00974	0.01687

PRMT7	0.02976	0.02441	0.00535	2.93118	0.00177	0.00893	0.00340
KRBA1	0.04979	0.04442	0.00537	2.60988	0.00133	0.00940	0.00910
DTX2	0.04305	0.03766	0.00539	2.29103	0.00078	0.01001	0.02203
ZNF514	0.02767	0.02226	0.00541	3.12861	0.00202	0.00880	0.00177
SSH1	0.04070	0.03525	0.00545	2.17080	0.00053	0.01038	0.03002
GAB2	0.06126	0.05581	0.00545	2.28475	0.00077	0.01013	0.02240
EFHD2	0.01876	0.01331	0.00545	2.98051	0.00187	0.00904	0.00290
ABHD17B	0.08000	0.07454	0.00546	1.99735	0.00010	0.01082	0.04588
HCFC1	0.05108	0.04560	0.00548	2.36375	0.00094	0.01003	0.01815
MCC	0.01908	0.01357	0.00552	3.36683	0.00230	0.00873	0.00077
TICAM1	0.07544	0.06988	0.00556	2.08855	0.00034	0.01077	0.03683
ZSCAN9	0.06518	0.05959	0.00559	2.18759	0.00058	0.01060	0.02878
DNMT3A	0.05002	0.04443	0.00559	2.02036	0.00016	0.01101	0.04343
F8	0.02650	0.02088	0.00563	2.74601	0.00161	0.00964	0.00607
TSTD2	0.04465	0.03901	0.00564	2.71500	0.00157	0.00972	0.00667
NKD1	0.08263	0.07698	0.00565	2.02361	0.00018	0.01112	0.04310
ZDHHC8	0.03457	0.02891	0.00566	2.19674	0.00061	0.01071	0.02811
LINC00672	0.05199	0.04633	0.00566	2.03982	0.00022	0.01110	0.04145
TMEM54	0.04855	0.04287	0.00568	2.10310	0.00038	0.01098	0.03554
MCPH1	0.07004	0.06436	0.00569	2.01202	0.00014	0.01123	0.04431
ZNF599	0.04369	0.03797	0.00573	2.63994	0.00147	0.00998	0.00833
MED9	0.04387	0.03814	0.00573	2.17468	0.00056	0.01090	0.02973
SLC35C1	0.03805	0.03228	0.00577	2.28261	0.00081	0.01073	0.02252
HCG18	0.07304	0.06724	0.00580	2.14893	0.00051	0.01110	0.03172
PLBD1	0.02682	0.02100	0.00582	3.03051	0.00206	0.00959	0.00246
CCNK	0.05361	0.04778	0.00582	2.13707	0.00048	0.01117	0.03267
SPHK1	0.04487	0.03904	0.00583	2.09314	0.00037	0.01130	0.03642
TNIK	0.04203	0.03619	0.00584	2.82917	0.00179	0.00989	0.00470
ZMIZ1	0.03508	0.02923	0.00585	2.25068	0.00075	0.01095	0.02448
SOX18	0.03047	0.02461	0.00586	2.11361	0.00042	0.01130	0.03463
MPP5	0.05199	0.04611	0.00589	2.31715	0.00091	0.01087	0.02056
POLR2J2	0.06277	0.05688	0.00589	2.09969	0.00039	0.01139	0.03584

SMARCD2	0.06043	0.05453	0.00590	2.33014	0.00094	0.01086	0.01986
TSPAN17	0.05247	0.04650	0.00597	2.56065	0.00140	0.01055	0.01050
RBP7	0.04537	0.03935	0.00602	1.97536	0.00004	0.01199	0.04832
TRIM2	0.05517	0.04912	0.00605	2.36781	0.00104	0.01106	0.01796
ALKBH1	0.03843	0.03236	0.00607	2.52384	0.00135	0.01078	0.01166
ZC3H7B	0.06714	0.06107	0.00607	2.16505	0.00057	0.01157	0.03046
TFF3	0.03903	0.03290	0.00614	2.38028	0.00108	0.01119	0.01736
RAD51D	0.03669	0.03055	0.00614	2.76431	0.00179	0.01050	0.00574
RNF170	0.07350	0.06735	0.00615	2.12925	0.00049	0.01181	0.03331
PQLC1	0.03869	0.03254	0.00615	2.52976	0.00138	0.01091	0.01146
GPR183	0.07467	0.06851	0.00615	2.06164	0.00030	0.01200	0.03933
KDELC1	0.02286	0.01670	0.00616	3.36366	0.00257	0.00975	0.00078
CCDC157	0.01931	0.01315	0.00616	3.56548	0.00277	0.00955	0.00037
GSTO2	0.06054	0.05436	0.00618	2.17253	0.00060	0.01176	0.02989
LRIF1	0.07277	0.06659	0.00618	2.00624	0.00014	0.01222	0.04492
MTIF2	0.08245	0.07623	0.00622	1.97799	0.00005	0.01239	0.04802
ZNF554	0.03938	0.03316	0.00622	2.91114	0.00203	0.01042	0.00363
FANCL	0.06692	0.06069	0.00623	2.29633	0.00091	0.01154	0.02173
SOX13	0.03381	0.02757	0.00624	2.99020	0.00215	0.01032	0.00281
DOK1	0.07981	0.07355	0.00626	2.16247	0.00058	0.01193	0.03066
MED1	0.04542	0.03915	0.00627	2.84335	0.00195	0.01059	0.00449
SLX4IP	0.06918	0.06291	0.00628	2.18741	0.00065	0.01190	0.02879
ATM	0.08581	0.07952	0.00629	2.33317	0.00100	0.01157	0.01970
TIMM8A	0.04137	0.03507	0.00630	2.93383	0.00209	0.01052	0.00337
RANGAP1	0.07994	0.07361	0.00633	2.18975	0.00066	0.01199	0.02862
CARD8	0.07599	0.06965	0.00634	2.33548	0.00102	0.01166	0.01958
CRKL	0.05290	0.04653	0.00637	2.69363	0.00173	0.01101	0.00711
COX6A2	0.06036	0.05394	0.00642	1.97831	0.00006	0.01279	0.04798
FEN1	0.08322	0.07674	0.00647	1.96146	0.00000	0.01294	0.04992
PIGO	0.05394	0.04745	0.00649	2.80510	0.00195	0.01103	0.00506
KRI1	0.09657	0.09007	0.00650	2.03885	0.00025	0.01275	0.04155
TGFBR3	0.05618	0.04967	0.00651	2.08336	0.00038	0.01263	0.03730

RELN	0.02337	0.01685	0.00652	3.31639	0.00266	0.01037	0.00092
DOCK6	0.04627	0.03975	0.00652	2.22707	0.00078	0.01226	0.02602
GNS	0.07422	0.06769	0.00653	2.23415	0.00080	0.01226	0.02555
MBP	0.04823	0.04169	0.00654	2.21091	0.00074	0.01234	0.02712
CLN8	0.07543	0.06887	0.00655	2.25140	0.00085	0.01226	0.02443
RAB3D	0.05647	0.04988	0.00659	2.45441	0.00133	0.01186	0.01417
TMEM97	0.05258	0.04598	0.00660	3.18580	0.00254	0.01066	0.00146
TRPC6	0.06849	0.06187	0.00662	2.37613	0.00116	0.01208	0.01756
CDK13	0.05377	0.04715	0.00662	3.27223	0.00265	0.01059	0.00108
PCDHB5	0.06828	0.06166	0.00662	2.41582	0.00125	0.01200	0.01576
ACER3	0.05097	0.04433	0.00664	2.86075	0.00209	0.01119	0.00426
PPFIA2	0.06087	0.05422	0.00664	2.90247	0.00216	0.01113	0.00373
MANBA	0.09121	0.08453	0.00668	2.05967	0.00032	0.01303	0.03952
POM121C	0.04233	0.03564	0.00669	2.61752	0.00168	0.01171	0.00890
CCDC28B	0.06447	0.05776	0.00671	2.14924	0.00059	0.01283	0.03169
SMG1	0.09709	0.09037	0.00672	1.98517	0.00008	0.01336	0.04722
ICAM2	0.04079	0.03407	0.00673	2.18569	0.00069	0.01277	0.02891
TSHZ2	0.03751	0.03077	0.00674	2.15668	0.00061	0.01288	0.03111
LRBA	0.06757	0.06082	0.00675	2.52090	0.00150	0.01200	0.01176
HMG20A	0.07501	0.06822	0.00679	2.01827	0.00019	0.01339	0.04365
CD53	0.06388	0.05708	0.00680	2.32721	0.00107	0.01254	0.02002
ZNF615	0.03650	0.02964	0.00685	3.16143	0.00260	0.01110	0.00159
CARNS1	0.07212	0.06523	0.00690	2.65356	0.00180	0.01199	0.00801
PLPPR2	0.09776	0.09086	0.00690	2.38020	0.00122	0.01259	0.01736
LRRC14	0.05715	0.05021	0.00695	2.73176	0.00196	0.01193	0.00634
OSBP	0.10487	0.09792	0.00695	2.19637	0.00075	0.01316	0.02814
TLCD2	0.06711	0.06015	0.00696	2.80293	0.00209	0.01182	0.00510
FAN1	0.06327	0.05630	0.00696	2.75545	0.00201	0.01192	0.00590
AIFM1	0.09735	0.09039	0.00696	2.38712	0.00124	0.01268	0.01704
ELMOD2	0.10237	0.09540	0.00697	1.97542	0.00005	0.01388	0.04831
SPIDR	0.07534	0.06829	0.00705	2.56717	0.00167	0.01243	0.01030
DIAPH1	0.12423	0.11715	0.00708	2.06829	0.00037	0.01379	0.03870

EIF4E3	0.07998	0.07289	0.00709	2.30254	0.00105	0.01312	0.02137
MGAT5	0.05745	0.05035	0.00710	2.27103	0.00097	0.01322	0.02322
RNF166	0.07416	0.06704	0.00712	2.56053	0.00167	0.01257	0.01050
CA5B	0.05264	0.04550	0.00715	2.96174	0.00241	0.01188	0.00308
TBC1D25	0.09830	0.09114	0.00716	2.44769	0.00143	0.01290	0.01443
IKZF4	0.06021	0.05304	0.00717	2.65280	0.00187	0.01247	0.00802
ZNF76	0.11996	0.11277	0.00719	1.99285	0.00012	0.01426	0.04637
LRCH3	0.09739	0.09020	0.00719	2.12066	0.00054	0.01384	0.03403
PID1	0.08478	0.07755	0.00723	2.04870	0.00031	0.01415	0.04058
COQ5	0.12646	0.11922	0.00725	2.02288	0.00022	0.01428	0.04317
TESK1	0.05528	0.04799	0.00730	3.20640	0.00283	0.01176	0.00136
EIF2AK3	0.05834	0.05102	0.00732	2.41371	0.00137	0.01327	0.01585
PDE8A	0.03457	0.02725	0.00732	3.20282	0.00284	0.01181	0.00138
INTS5	0.09530	0.08797	0.00733	2.37413	0.00128	0.01338	0.01765
HIF3A	0.09602	0.08868	0.00734	2.35254	0.00122	0.01346	0.01871
POMGNT2	0.11557	0.10822	0.00735	2.06647	0.00038	0.01432	0.03887
SPRTN	0.12145	0.11410	0.00735	2.01270	0.00019	0.01451	0.04424
FGD4	0.09547	0.08809	0.00738	2.28982	0.00106	0.01370	0.02210
CFI	0.06940	0.06194	0.00747	2.31400	0.00114	0.01379	0.02073
ACTR8	0.10521	0.09772	0.00749	2.27556	0.00104	0.01395	0.02294
LYSMD3	0.08350	0.07597	0.00753	2.38322	0.00133	0.01372	0.01722
HS3ST1	0.03712	0.02957	0.00756	3.30564	0.00307	0.01204	0.00096
IGF2	0.06138	0.05382	0.00756	2.20614	0.00084	0.01427	0.02745
STAT5A	0.12977	0.12220	0.00757	2.03224	0.00027	0.01488	0.04222
TP53	0.12670	0.11905	0.00766	1.97321	0.00005	0.01526	0.04856
RNASEH1-AS1	0.09784	0.09016	0.00768	2.12774	0.00060	0.01476	0.03344
CLDN12	0.11411	0.10642	0.00769	2.11970	0.00058	0.01480	0.03411
CDK17	0.10396	0.09623	0.00773	1.99306	0.00013	0.01534	0.04634
TRAF4	0.07475	0.06699	0.00776	2.10205	0.00052	0.01500	0.03563
FRS2	0.04769	0.03989	0.00780	3.77306	0.00375	0.01186	0.00016
GABPB1-AS1	0.09791	0.09007	0.00784	2.00549	0.00017	0.01550	0.04500
MTERF2	0.09829	0.09045	0.00784	2.17401	0.00077	0.01491	0.02978

ATXN1	0.05880	0.05092	0.00788	3.37697	0.00330	0.01245	0.00074
ZNF91	0.10467	0.09676	0.00791	2.26008	0.00105	0.01477	0.02389
FOXO1	0.10234	0.09443	0.00791	2.20663	0.00088	0.01495	0.02741
MAML2	0.05072	0.04281	0.00792	2.51548	0.00175	0.01409	0.01194
ZNF562	0.09909	0.09113	0.00796	2.26845	0.00108	0.01483	0.02337
ABHD5	0.05213	0.04417	0.00796	2.99546	0.00275	0.01317	0.00276
TMEM100	0.03169	0.02372	0.00797	2.41156	0.00149	0.01446	0.01594
LINC01184	0.12028	0.11229	0.00798	2.39576	0.00145	0.01452	0.01665
MYO9B	0.08410	0.07611	0.00799	2.36693	0.00137	0.01461	0.01800
RABL6	0.11770	0.10965	0.00805	2.23947	0.00100	0.01509	0.02520
APOL2	0.10892	0.10087	0.00805	2.13782	0.00067	0.01544	0.03261
TMTC1	0.06150	0.05344	0.00806	3.31981	0.00330	0.01282	0.00091
MRPL46	0.15068	0.14261	0.00806	2.07293	0.00044	0.01569	0.03826
WSB2	0.12536	0.11729	0.00806	2.06194	0.00040	0.01573	0.03930
LRTM1	0.03337	0.02528	0.00809	3.04352	0.00288	0.01330	0.00236
POGLUT1	0.12625	0.11812	0.00813	2.11483	0.00059	0.01567	0.03453
HTRA3	0.03396	0.02582	0.00814	2.92688	0.00269	0.01359	0.00345
STMN3	0.07200	0.06383	0.00817	2.36958	0.00141	0.01492	0.01787
GJC1	0.04766	0.03947	0.00819	3.08192	0.00298	0.01340	0.00208
SLC39A11	0.07156	0.06333	0.00822	2.63812	0.00211	0.01434	0.00838
UQCRHL	0.14438	0.13613	0.00825	2.03622	0.00031	0.01619	0.04182
FASTKD2	0.09094	0.08269	0.00825	2.44821	0.00164	0.01486	0.01441
SEMA3C	0.05998	0.05170	0.00828	2.42086	0.00157	0.01499	0.01554
MTA3	0.07932	0.07102	0.00829	2.63092	0.00211	0.01447	0.00856
EZH1	0.10946	0.10113	0.00833	2.23581	0.00102	0.01563	0.02544
RDH5	0.04058	0.03224	0.00835	3.57165	0.00376	0.01293	0.00036
NPHP3	0.08874	0.08037	0.00838	2.32395	0.00131	0.01544	0.02019
LDB2	0.06462	0.05624	0.00838	2.16525	0.00079	0.01596	0.03045
MCEE	0.09710	0.08871	0.00839	2.27297	0.00115	0.01563	0.02310
FCF1	0.11517	0.10672	0.00845	2.19818	0.00091	0.01598	0.02801
MOK	0.10193	0.09346	0.00847	2.33494	0.00136	0.01558	0.01961
NOC3L	0.08897	0.08048	0.00848	2.39243	0.00153	0.01544	0.01680

TMEM186	0.10659	0.09803	0.00856	2.47144	0.00177	0.01536	0.01351
CLEC14A	0.04712	0.03852	0.00860	2.38368	0.00153	0.01567	0.01720
HILPDA	0.08672	0.07806	0.00866	2.70094	0.00237	0.01494	0.00695
CBFB	0.10832	0.09966	0.00866	1.98243	0.00009	0.01722	0.04752
DECR2	0.11980	0.11114	0.00866	2.54618	0.00199	0.01533	0.01094
ZADH2	0.05073	0.04207	0.00866	2.06181	0.00042	0.01690	0.03931
ARHGAP5-AS1	0.11291	0.10425	0.00866	2.45298	0.00174	0.01559	0.01422
ZDHHC1	0.06007	0.05139	0.00868	2.87822	0.00277	0.01459	0.00403
SNHG21	0.07967	0.07091	0.00876	2.42667	0.00168	0.01584	0.01530
CHERP	0.14312	0.13434	0.00878	2.35564	0.00147	0.01609	0.01855
ME3	0.12569	0.11690	0.00879	2.17233	0.00086	0.01672	0.02991
LPCAT4	0.07261	0.06381	0.00880	3.04236	0.00313	0.01448	0.00237
AMT	0.05899	0.05018	0.00881	3.98117	0.00447	0.01315	0.00007
HDAC11	0.10829	0.09944	0.00885	2.68271	0.00238	0.01532	0.00734
TOM1L2	0.09787	0.08900	0.00887	2.04251	0.00036	0.01739	0.04119
NEDD9	0.12047	0.11157	0.00890	2.13331	0.00072	0.01708	0.03298
ZNF830	0.19192	0.18300	0.00892	2.00147	0.00018	0.01765	0.04543
KHDRBS3	0.09031	0.08138	0.00893	2.64073	0.00230	0.01556	0.00832
NAAA	0.11477	0.10573	0.00904	1.99917	0.00017	0.01790	0.04568
MAPK10	0.10745	0.09840	0.00905	2.50619	0.00197	0.01613	0.01226
PHRF1	0.03320	0.02414	0.00906	4.80184	0.00536	0.01277	0.00000
BAZ2B	0.12423	0.11503	0.00920	2.17031	0.00089	0.01752	0.03006
TOR3A	0.14567	0.13646	0.00921	2.12922	0.00073	0.01770	0.03332
TMEM218	0.12425	0.11502	0.00922	2.07939	0.00053	0.01792	0.03767
LZTS2	0.16802	0.15875	0.00927	2.03351	0.00033	0.01821	0.04209
UBAP2L	0.12649	0.11706	0.00943	2.24735	0.00120	0.01765	0.02469
LDLRAD3	0.07590	0.06646	0.00944	2.04378	0.00038	0.01849	0.04106
SLN	0.02554	0.01609	0.00944	3.00614	0.00328	0.01560	0.00267
HUWE1	0.15717	0.14769	0.00948	2.28915	0.00136	0.01760	0.02214
KLF3	0.11191	0.10237	0.00954	2.41158	0.00178	0.01729	0.01594
MPDU1	0.11446	0.10488	0.00958	2.20127	0.00105	0.01811	0.02779
STAM	0.10309	0.09348	0.00961	1.97844	0.00009	0.01914	0.04797

FADD	0.16079	0.15111	0.00968	2.37068	0.00167	0.01769	0.01782
UBTD1	0.10717	0.09746	0.00971	2.75154	0.00279	0.01662	0.00597
KPNA1	0.15284	0.14310	0.00974	1.98683	0.00013	0.01935	0.04703
FLJ20021	0.09579	0.08603	0.00975	2.26751	0.00132	0.01819	0.02343
GALK2	0.17251	0.16275	0.00976	2.14299	0.00083	0.01868	0.03219
ZNF558	0.07534	0.06554	0.00981	2.27967	0.00137	0.01824	0.02270
ZNF322	0.13232	0.12248	0.00984	2.56301	0.00231	0.01737	0.01042
NUP107	0.11111	0.10123	0.00987	2.27601	0.00137	0.01838	0.02292
POLR3F	0.13100	0.12111	0.00988	2.47478	0.00205	0.01771	0.01339
RALA	0.15521	0.14532	0.00990	2.49539	0.00212	0.01767	0.01263
BAG5	0.14441	0.13449	0.00992	2.36995	0.00171	0.01813	0.01785
SLC2A4RG	0.14867	0.13874	0.00993	2.24221	0.00125	0.01862	0.02502
MAP3K7	0.20613	0.19614	0.00999	2.01838	0.00029	0.01969	0.04364
FBXW7	0.09624	0.08625	0.00999	2.00207	0.00021	0.01977	0.04537
PDE1B	0.15154	0.14152	0.01002	2.21232	0.00114	0.01891	0.02702
SERPINB9	0.05490	0.04485	0.01005	3.17548	0.00384	0.01626	0.00151
B3GLCT	0.10047	0.09042	0.01005	1.97637	0.00008	0.02002	0.04820
TNFSF10	0.04011	0.03004	0.01007	2.62259	0.00254	0.01760	0.00877
MAPKAPK5	0.11405	0.10395	0.01010	2.35045	0.00168	0.01853	0.01881
MARCKSL1	0.07340	0.06316	0.01024	2.14239	0.00087	0.01962	0.03224
TMEM246	0.18900	0.17873	0.01027	2.12115	0.00078	0.01977	0.03399
C12orf49	0.11892	0.10862	0.01030	2.59356	0.00251	0.01809	0.00954
NKIRAS2	0.21246	0.20213	0.01033	1.97751	0.00009	0.02058	0.04808
IVD	0.26133	0.25098	0.01035	1.99566	0.00018	0.02052	0.04606
PDE6D	0.15170	0.14128	0.01043	2.04542	0.00043	0.02042	0.04090
MRPL45	0.26475	0.25431	0.01044	1.98768	0.00014	0.02073	0.04694
NECAP1	0.13507	0.12461	0.01046	2.07129	0.00056	0.02036	0.03842
WWP2	0.19649	0.18603	0.01046	2.01222	0.00027	0.02065	0.04428
CHCHD6	0.12821	0.11773	0.01048	2.99411	0.00362	0.01735	0.00277
TSC22D4	0.20528	0.19479	0.01048	2.06651	0.00054	0.02043	0.03886
KALRN	0.16465	0.15416	0.01049	2.08715	0.00064	0.02034	0.03696
TRUB2	0.16696	0.15647	0.01049	2.25124	0.00135	0.01963	0.02444

POLR2A	0.13511	0.12460	0.01051	2.44747	0.00209	0.01892	0.01444
EPHX2	0.13467	0.12411	0.01056	2.66701	0.00280	0.01832	0.00769
RHOQ	0.20101	0.19044	0.01056	2.16125	0.00098	0.02015	0.03075
HEPH	0.20371	0.19311	0.01060	2.03972	0.00041	0.02079	0.04147
OSMR	0.09657	0.08592	0.01065	2.32909	0.00168	0.01961	0.01992
TFDP2	0.18700	0.17634	0.01066	2.07069	0.00057	0.02075	0.03847
PRKAA2	0.17508	0.16441	0.01067	2.05682	0.00050	0.02084	0.03979
ZNF75A	0.13851	0.12784	0.01067	2.37279	0.00185	0.01949	0.01772
AFMID	0.13776	0.12706	0.01070	2.56887	0.00253	0.01886	0.01025
SRGAP2C	0.09793	0.08719	0.01074	2.41591	0.00202	0.01946	0.01575
IGF1	0.04147	0.03072	0.01074	2.64389	0.00278	0.01871	0.00824
SENP5	0.18543	0.17462	0.01081	1.97644	0.00009	0.02153	0.04820
GTF2IRD2B	0.10237	0.09155	0.01082	2.83486	0.00334	0.01830	0.00461
ACIN1	0.22506	0.21419	0.01087	2.12511	0.00084	0.02089	0.03366
ATG4A	0.10917	0.09822	0.01096	3.17037	0.00418	0.01773	0.00154
BPHL	0.09038	0.07942	0.01096	3.61143	0.00501	0.01692	0.00031
FTX	0.10206	0.09106	0.01100	3.06906	0.00397	0.01803	0.00217
IPO8	0.08499	0.07398	0.01101	2.39077	0.00198	0.02005	0.01687
INIP	0.12871	0.11769	0.01102	2.97346	0.00375	0.01829	0.00297
UBE2R2	0.22546	0.21444	0.01103	2.05654	0.00051	0.02154	0.03982
RRAS2	0.19789	0.18686	0.01103	2.32718	0.00174	0.02033	0.02002
KLHDC4	0.17020	0.15913	0.01107	2.51963	0.00246	0.01968	0.01180
FUZ	0.14521	0.13411	0.01110	2.72198	0.00310	0.01909	0.00653
CEP63	0.23186	0.22076	0.01111	2.07410	0.00061	0.02160	0.03815
SKA2	0.28710	0.27599	0.01112	2.02861	0.00037	0.02186	0.04259
RPP21	0.12303	0.11166	0.01137	2.51843	0.00252	0.02022	0.01184
MT1F	0.17352	0.16208	0.01144	1.99125	0.00018	0.02270	0.04654
HDHD3	0.16766	0.15620	0.01147	2.57784	0.00274	0.02019	0.00999
UHMK1	0.13450	0.12302	0.01148	2.21367	0.00131	0.02165	0.02693
E2F6	0.11647	0.10496	0.01151	2.18673	0.00119	0.02184	0.02884
NF1	0.15298	0.14143	0.01154	2.38868	0.00207	0.02102	0.01697
TNRC6A	0.25824	0.24663	0.01162	1.99358	0.00019	0.02304	0.04629

WDR82	0.20976	0.19813	0.01163	2.14559	0.00100	0.02226	0.03199
CHMP4C	0.16128	0.14957	0.01172	2.19283	0.00124	0.02219	0.02840
ZNF800	0.16315	0.15144	0.01172	2.47070	0.00242	0.02102	0.01354
TMEM189	0.14262	0.13085	0.01177	3.14202	0.00443	0.01912	0.00169
VWF	0.08447	0.07265	0.01182	1.99289	0.00019	0.02345	0.04636
FAM8A1	0.09821	0.08638	0.01183	2.10262	0.00080	0.02286	0.03558
WARS2	0.12025	0.10832	0.01194	2.36851	0.00206	0.02182	0.01792
MAP3K5	0.10651	0.09454	0.01197	2.31807	0.00184	0.02209	0.02051
MORC4	0.10197	0.08995	0.01203	2.04235	0.00048	0.02357	0.04120
ELAC2	0.18970	0.17755	0.01215	2.59964	0.00299	0.02131	0.00938
KMT5A	0.16813	0.15597	0.01216	2.32774	0.00192	0.02240	0.01999
NSMCE2	0.22087	0.20871	0.01217	2.05930	0.00058	0.02375	0.03955
IFT46	0.17453	0.16236	0.01217	2.48752	0.00258	0.02176	0.01292
TRIM28	0.13280	0.12062	0.01218	2.65825	0.00320	0.02117	0.00790
PNO1	0.26170	0.24946	0.01224	2.20694	0.00137	0.02311	0.02739
RNF213	0.15776	0.14547	0.01229	2.14722	0.00107	0.02350	0.03186
HDAC7	0.19224	0.17995	0.01229	2.15930	0.00113	0.02345	0.03091
HLA-DRB5	0.13361	0.12131	0.01230	2.31102	0.00186	0.02274	0.02090
IRF3	0.24619	0.23387	0.01232	2.20535	0.00137	0.02327	0.02750
CAMK2D	0.16831	0.15587	0.01244	2.97914	0.00425	0.02063	0.00291
CFAP20	0.21067	0.19821	0.01246	2.61475	0.00312	0.02180	0.00897
DR1	0.22332	0.21078	0.01254	2.24207	0.00157	0.02351	0.02503
PNPT1	0.13332	0.12076	0.01257	2.68136	0.00338	0.02176	0.00737
ALKBH3	0.20217	0.18956	0.01261	2.49431	0.00270	0.02253	0.01267
USP1	0.27668	0.26406	0.01262	2.14286	0.00107	0.02417	0.03220
KIAA2026	0.14981	0.13718	0.01263	2.59232	0.00308	0.02219	0.00958
NACA2	0.20708	0.19444	0.01264	2.10914	0.00089	0.02438	0.03501
ANKRD35	0.15643	0.14376	0.01267	2.79852	0.00379	0.02154	0.00517
FBXW2	0.29227	0.27958	0.01269	1.97878	0.00012	0.02526	0.04793
KCNK15	0.18293	0.17013	0.01281	2.06443	0.00064	0.02497	0.03906
RNF121	0.13281	0.11998	0.01283	2.25021	0.00165	0.02400	0.02451
NUPR2	0.11105	0.09811	0.01294	3.31629	0.00529	0.02058	0.00092

CLN3	0.21032	0.19736	0.01296	2.00855	0.00031	0.02561	0.04467
AP1G1	0.17573	0.16274	0.01299	2.84535	0.00404	0.02194	0.00447
SSH3	0.19239	0.17923	0.01316	2.40978	0.00245	0.02388	0.01602
HNRNPLL	0.24345	0.23028	0.01317	2.26024	0.00175	0.02460	0.02388
TPD52	0.15851	0.14529	0.01322	2.73408	0.00374	0.02270	0.00629
SMAD9	0.24394	0.23070	0.01324	1.96432	0.00002	0.02646	0.04958
TBL2	0.20701	0.19376	0.01325	2.91044	0.00432	0.02218	0.00364
PDCD7	0.22738	0.21411	0.01327	1.98205	0.00014	0.02641	0.04756
MGST1	0.05676	0.04344	0.01332	3.26117	0.00531	0.02132	0.00112
TSPAN12	0.19408	0.18075	0.01333	2.16335	0.00125	0.02541	0.03059
DNAJC9	0.20767	0.19425	0.01342	2.32548	0.00210	0.02473	0.02011
PUM3	0.17170	0.15828	0.01343	2.88526	0.00430	0.02255	0.00394
DPH7	0.19627	0.18283	0.01344	2.52906	0.00302	0.02386	0.01149
POLR2H	0.22708	0.21346	0.01362	2.38963	0.00244	0.02479	0.01693
TMPO	0.12239	0.10877	0.01362	2.85354	0.00426	0.02299	0.00435
NFASC	0.16522	0.15159	0.01363	3.05722	0.00489	0.02237	0.00225
SLC7A2	0.21299	0.19931	0.01367	2.02366	0.00043	0.02692	0.04309
XPC	0.27963	0.26588	0.01375	2.11537	0.00101	0.02650	0.03448
SCAMP3	0.21241	0.19866	0.01375	2.09907	0.00091	0.02660	0.03589
OGDH	0.17558	0.16175	0.01383	2.42949	0.00267	0.02499	0.01518
CCND3	0.30980	0.29591	0.01390	2.33083	0.00221	0.02559	0.01983
BPGM	0.20718	0.19319	0.01398	2.49969	0.00302	0.02495	0.01248
C12orf29	0.24223	0.22814	0.01409	2.43464	0.00274	0.02544	0.01496
POLR2M	0.32782	0.31368	0.01414	2.08365	0.00083	0.02744	0.03728
CDIP1	0.13587	0.12165	0.01422	3.36356	0.00593	0.02252	0.00078
C1orf109	0.13507	0.12084	0.01423	1.96449	0.00003	0.02843	0.04956
SYNJ2BP	0.38827	0.37404	0.01423	2.13265	0.00115	0.02732	0.03303
SLC43A3	0.13946	0.12516	0.01430	3.17399	0.00547	0.02313	0.00152
CDV3	0.26702	0.25263	0.01439	2.56284	0.00338	0.02540	0.01043
TTC14	0.36336	0.34893	0.01443	1.98325	0.00016	0.02871	0.04743
TM9SF3	0.32038	0.30595	0.01444	2.08679	0.00087	0.02800	0.03699
FOSL2	0.29867	0.28413	0.01454	2.03418	0.00052	0.02855	0.04202

GMPR	0.37027	0.35573	0.01454	1.97503	0.00011	0.02897	0.04836
PIGP	0.37879	0.36423	0.01456	2.08796	0.00089	0.02824	0.03688
TGFB3	0.25482	0.24024	0.01458	2.10997	0.00103	0.02813	0.03494
MDK	0.12899	0.11440	0.01459	2.13667	0.00120	0.02798	0.03271
IST1	0.43248	0.41788	0.01460	1.99003	0.00021	0.02898	0.04668
MAP3K11	0.07824	0.06363	0.01461	3.67536	0.00681	0.02240	0.00024
TRIP10	0.42834	0.41370	0.01464	2.06350	0.00073	0.02855	0.03915
ZNF83	0.19594	0.18130	0.01464	2.58805	0.00355	0.02573	0.00970
UTP6	0.20598	0.19122	0.01476	2.40315	0.00272	0.02681	0.01631
IL10RB	0.16546	0.15069	0.01477	3.61661	0.00676	0.02278	0.00030
WARS	0.14349	0.12870	0.01479	2.97166	0.00503	0.02455	0.00299
NPRL3	0.29707	0.28227	0.01480	2.14054	0.00124	0.02835	0.03239
COMMD5	0.30662	0.29173	0.01488	2.03958	0.00058	0.02919	0.04148
NAPRT	0.29759	0.28260	0.01499	2.66325	0.00396	0.02603	0.00778
ETFDH	0.25552	0.24050	0.01502	2.59288	0.00366	0.02638	0.00956
TMA16	0.29732	0.28213	0.01519	2.30518	0.00227	0.02812	0.02122
SECISBP2	0.24105	0.22572	0.01534	2.49077	0.00326	0.02741	0.01280
RPA3	0.39286	0.37750	0.01536	2.02876	0.00051	0.03020	0.04257
SDR39U1	0.37251	0.35715	0.01536	2.32178	0.00239	0.02833	0.02031
ITFG2	0.11063	0.09523	0.01540	3.72705	0.00730	0.02350	0.00020
ALG5	0.38884	0.37330	0.01554	2.20633	0.00173	0.02934	0.02744
CBY1	0.37621	0.36052	0.01568	2.25519	0.00205	0.02932	0.02419
HGSNAT	0.11856	0.10285	0.01571	3.79666	0.00760	0.02382	0.00015
NAA50	0.27513	0.25926	0.01587	2.42060	0.00301	0.02872	0.01555
SLC30A9	0.21709	0.20102	0.01607	3.12747	0.00599	0.02614	0.00178
SDHAF2	0.30919	0.29310	0.01609	2.48163	0.00338	0.02880	0.01313
AATF	0.40174	0.38561	0.01613	1.98342	0.00018	0.03208	0.04741
CPSF7	0.33471	0.31854	0.01617	2.40233	0.00297	0.02937	0.01635
GPS1	0.38318	0.36700	0.01618	2.19168	0.00170	0.03066	0.02848
GSK3B	0.15776	0.14155	0.01621	3.11903	0.00602	0.02640	0.00183
NR4A2	0.25327	0.23699	0.01628	1.96339	0.00002	0.03254	0.04969
PLEC	0.25545	0.23907	0.01637	2.38195	0.00290	0.02985	0.01728

MRPL3	0.57032	0.55370	0.01662	2.03256	0.00059	0.03265	0.04218
ABI2	0.25813	0.24144	0.01669	2.30605	0.00250	0.03089	0.02118
PRKG1	0.49388	0.47712	0.01675	2.13187	0.00134	0.03216	0.03310
IMMP2L	0.45182	0.43496	0.01686	2.13360	0.00137	0.03236	0.03296
TXN2	0.60350	0.58657	0.01693	1.97609	0.00013	0.03372	0.04824
CMAS	0.38786	0.37071	0.01714	2.03239	0.00060	0.03368	0.04220
UBE2G2	0.31413	0.29685	0.01728	2.52493	0.00386	0.03070	0.01162
PTRHD1	0.19651	0.17917	0.01734	3.09383	0.00635	0.02833	0.00199
CLSTN1	0.23572	0.21834	0.01738	2.81736	0.00528	0.02947	0.00487
VPS25	0.34982	0.33233	0.01749	2.67606	0.00467	0.03030	0.00749
MUM1	0.44839	0.43090	0.01749	2.17407	0.00172	0.03326	0.02978
OFD1	0.17568	0.15817	0.01751	4.52486	0.00992	0.02510	0.00001
COA4	0.46167	0.44406	0.01761	2.05418	0.00080	0.03442	0.04004
CNP	0.35751	0.33984	0.01767	2.35451	0.00296	0.03239	0.01861
SWAP70	0.35480	0.33706	0.01774	1.97540	0.00013	0.03535	0.04831
GNAI1	0.29155	0.27367	0.01788	2.69323	0.00486	0.03089	0.00712
DECR1	0.29298	0.27500	0.01798	2.65629	0.00471	0.03125	0.00794
KMT2C	0.19488	0.17682	0.01807	2.97210	0.00615	0.02999	0.00298
CLIP1	0.41816	0.39995	0.01821	2.50468	0.00396	0.03247	0.01231
TIAL1	0.31207	0.29384	0.01822	2.57188	0.00433	0.03212	0.01016
ATG5	0.22490	0.20659	0.01831	2.74190	0.00522	0.03140	0.00614
GSPT1	0.31517	0.29679	0.01838	2.40734	0.00341	0.03336	0.01613
NOLC1	0.20613	0.18767	0.01846	2.73383	0.00522	0.03169	0.00630
WDR34	0.23140	0.21290	0.01849	2.58674	0.00448	0.03251	0.00974
PYGM	0.17128	0.15267	0.01861	3.86862	0.00918	0.02804	0.00011
TCEAL7	0.33677	0.31806	0.01871	2.41344	0.00351	0.03391	0.01586
KRAS	0.21329	0.19446	0.01883	2.16662	0.00179	0.03588	0.03034
FXYD6	0.22812	0.20926	0.01886	2.16966	0.00182	0.03591	0.03011
TADA3	0.53542	0.51654	0.01887	2.18611	0.00195	0.03580	0.02888
WFS1	0.58202	0.56299	0.01903	2.04993	0.00083	0.03722	0.04046
TLE1	0.40672	0.38755	0.01916	2.48929	0.00407	0.03426	0.01285
OXA1L	0.46201	0.44281	0.01921	2.22754	0.00230	0.03611	0.02598

SNAP47	0.19540	0.17615	0.01925	3.33348	0.00793	0.03057	0.00087
ILVBL	0.28489	0.26556	0.01933	2.16650	0.00184	0.03683	0.03035
ARMC10	0.34297	0.32338	0.01959	2.58211	0.00471	0.03447	0.00987
HLA-F	0.28356	0.26383	0.01973	2.54215	0.00451	0.03494	0.01107
MRPS2	0.32296	0.30323	0.01973	2.42283	0.00376	0.03570	0.01546
SMARCA1	0.39352	0.37355	0.01998	2.24779	0.00255	0.03740	0.02466
ANGPT1	0.26947	0.24942	0.02005	2.21713	0.00232	0.03778	0.02669
AHI1	0.37681	0.35669	0.02012	2.49304	0.00429	0.03594	0.01272
CKLF	0.27644	0.25629	0.02016	2.35453	0.00337	0.03694	0.01861
EGLN2	0.54546	0.52522	0.02024	2.14032	0.00170	0.03878	0.03241
MX1	0.20649	0.18612	0.02037	3.92280	0.01019	0.03056	0.00009
IFI27	0.17379	0.15322	0.02057	2.18890	0.00214	0.03899	0.02868
TULP3	0.32093	0.30029	0.02064	2.04139	0.00082	0.04046	0.04130
PTN	0.19116	0.17043	0.02074	2.85552	0.00650	0.03497	0.00433
GPN3	0.47237	0.45156	0.02081	2.42471	0.00398	0.03763	0.01538
SZRD1	0.61539	0.59445	0.02095	2.06910	0.00110	0.04079	0.03862
PFKL	0.63864	0.61767	0.02097	2.13395	0.00170	0.04023	0.03293
SNAPC5	0.48151	0.46054	0.02097	2.68095	0.00563	0.03631	0.00738
PFDN6	0.45251	0.43130	0.02121	2.89651	0.00685	0.03557	0.00380
PTOV1	0.72868	0.70726	0.02142	2.06487	0.00108	0.04176	0.03902
RABGGTB	0.40952	0.38789	0.02162	2.79921	0.00648	0.03677	0.00516
NMD3	0.49616	0.47418	0.02198	2.30662	0.00330	0.04067	0.02114
SOX4	0.39165	0.36966	0.02199	2.25950	0.00291	0.04108	0.02392
COPS4	0.63747	0.61546	0.02201	2.15380	0.00197	0.04204	0.03133
SMIM12	0.87360	0.85150	0.02210	1.99157	0.00034	0.04386	0.04651
MED4	0.99536	0.97321	0.02215	2.01119	0.00056	0.04375	0.04439
TMEM128	0.22355	0.20114	0.02241	3.40700	0.00951	0.03531	0.00067
ATG3	0.56067	0.53801	0.02267	2.61391	0.00566	0.03967	0.00900
PDE5A	0.38142	0.35864	0.02279	2.49114	0.00485	0.04072	0.01279
CLDN5	0.10329	0.08043	0.02286	2.97116	0.00777	0.03794	0.00299
JAK1	0.55756	0.53450	0.02306	2.14258	0.00196	0.04416	0.03223
MFF	0.65735	0.63412	0.02323	2.40389	0.00428	0.04217	0.01628

GTF3C6	0.84866	0.82531	0.02335	1.97492	0.00017	0.04653	0.04837
SRP68	0.61118	0.58732	0.02386	2.15245	0.00212	0.04559	0.03144
SCAF11	0.74198	0.71802	0.02397	2.10435	0.00164	0.04630	0.03543
NOP56	0.74974	0.72566	0.02408	2.15329	0.00215	0.04600	0.03137
DTWD1	0.36265	0.33856	0.02409	2.40111	0.00442	0.04376	0.01641
DARS	0.88833	0.86418	0.02414	2.12599	0.00188	0.04641	0.03358
CSNK1D	0.44511	0.42081	0.02430	2.98073	0.00832	0.04029	0.00290
CFDP1	0.75062	0.72615	0.02447	2.70371	0.00672	0.04221	0.00690
UBE2E2	0.59393	0.56924	0.02469	2.30528	0.00369	0.04568	0.02122
NEMF	0.57656	0.55170	0.02487	2.62187	0.00627	0.04346	0.00879
FAM32A	0.60717	0.58197	0.02519	2.04388	0.00102	0.04936	0.04105
FGD5-AS1	0.47454	0.44920	0.02534	3.00439	0.00880	0.04188	0.00268
PHF14	0.56725	0.54176	0.02549	2.63964	0.00656	0.04443	0.00834
HDAC2	0.71496	0.68927	0.02569	2.26083	0.00341	0.04797	0.02384
MPHOSPH10	0.29261	0.26685	0.02575	3.82344	0.01255	0.03896	0.00013
PLSCR1	0.33606	0.31015	0.02590	2.47497	0.00538	0.04643	0.01338
PAIP1	0.81356	0.78751	0.02604	2.18328	0.00265	0.04943	0.02909
DDA1	0.39511	0.36903	0.02608	3.01847	0.00914	0.04302	0.00256
STK25	0.45578	0.42954	0.02625	3.17389	0.01003	0.04246	0.00152
KAT5	0.61448	0.58823	0.02625	2.72528	0.00736	0.04514	0.00646
NT5C3B	0.76367	0.73716	0.02651	2.19229	0.00280	0.05023	0.02843
MAP7D1	0.91396	0.88738	0.02658	1.97694	0.00022	0.05294	0.04814
MAGED1	0.49034	0.46356	0.02678	3.26527	0.01070	0.04287	0.00111
CD2BP2	0.55123	0.52438	0.02685	2.60304	0.00663	0.04708	0.00929
PHB2	1.00825	0.98137	0.02688	2.05746	0.00126	0.05249	0.03973
NDUFB5	0.83567	0.80807	0.02760	2.42439	0.00528	0.04992	0.01539
MAT2A	0.48023	0.45181	0.02842	2.82528	0.00870	0.04815	0.00475
HEXB	0.75541	0.72674	0.02867	2.35166	0.00477	0.05258	0.01875
RAB18	1.01028	0.98151	0.02876	2.31199	0.00437	0.05316	0.02084
PSMD7	0.77278	0.74364	0.02914	2.60284	0.00719	0.05109	0.00929
PSMG2	1.02873	0.99937	0.02936	2.21496	0.00337	0.05534	0.02684
GORASP2	0.56840	0.53902	0.02938	2.20039	0.00320	0.05556	0.02785

RBM25	0.96625	0.93687	0.02938	2.08157	0.00171	0.05706	0.03747
CCL21	0.30879	0.27814	0.03066	2.01077	0.00076	0.06055	0.04444
HSPA9	1.19093	1.16027	0.03066	2.09880	0.00202	0.05930	0.03592
RNASE1	0.35054	0.31956	0.03097	2.45158	0.00620	0.05575	0.01428
CDS2	0.60287	0.57180	0.03107	3.49814	0.01365	0.04848	0.00048
GLO1	0.89935	0.86825	0.03109	2.00766	0.00073	0.06146	0.04477
TOMM40	0.36070	0.32960	0.03111	3.47285	0.01354	0.04867	0.00052
AIMP1	0.93684	0.90570	0.03114	2.36635	0.00534	0.05694	0.01803
CTSC	0.31328	0.28211	0.03118	2.26356	0.00417	0.05818	0.02367
EBAG9	0.31275	0.28132	0.03143	4.77416	0.01852	0.04434	0.00000
AHSA1	0.72320	0.69174	0.03146	2.99688	0.01088	0.05204	0.00275
EIF6	1.09816	1.06668	0.03148	2.17953	0.00316	0.05980	0.02937
MRPS36	0.84488	0.81280	0.03208	2.55003	0.00741	0.05675	0.01082
TMEM50B	0.66713	0.63496	0.03217	2.31485	0.00492	0.05942	0.02069
EHD2	1.15353	1.12075	0.03278	2.28175	0.00461	0.06095	0.02257
HLA-DPA1	0.83959	0.80680	0.03280	2.30563	0.00491	0.06069	0.02120
PSMC4	0.74407	0.71109	0.03298	2.98488	0.01132	0.05465	0.00286
SLC41A3	0.32268	0.28875	0.03393	4.88085	0.02030	0.04756	0.00000
NDUFA8	0.62906	0.59509	0.03397	3.16336	0.01291	0.05502	0.00158
PLCG2	0.54907	0.51479	0.03428	2.12837	0.00270	0.06586	0.03339
ST6GALNAC6	1.08196	1.04739	0.03457	2.49802	0.00743	0.06170	0.01254
NDUFS4	1.56418	1.52945	0.03472	2.05248	0.00155	0.06790	0.04021
CHMP4A	0.92468	0.88960	0.03508	2.62559	0.00888	0.06127	0.00869
SERPINF1	0.46337	0.42815	0.03522	2.67206	0.00938	0.06107	0.00758
MRPS26	0.72746	0.69115	0.03632	3.32125	0.01488	0.05776	0.00091
PET100	1.26326	1.22671	0.03655	2.43402	0.00711	0.06599	0.01499
SEC23A	0.66388	0.62660	0.03728	3.38999	0.01572	0.05884	0.00071
HLA-DRB1	0.74137	0.70391	0.03746	2.57350	0.00892	0.06600	0.01011
KDELR1	1.01501	0.97744	0.03756	2.39362	0.00679	0.06834	0.01674
PIN1	1.51354	1.47506	0.03848	2.34554	0.00631	0.07064	0.01906
NUTF2	1.00824	0.96962	0.03862	3.13662	0.01448	0.06276	0.00173
BUD31	1.19842	1.15901	0.03941	2.30319	0.00586	0.07296	0.02134

TCEAL3	1.54555	1.50613	0.03942	1.97097	0.00020	0.07864	0.04882
PRDX3	1.41096	1.37147	0.03949	2.48483	0.00833	0.07065	0.01302
MMADHC	1.22204	1.18151	0.04053	2.77316	0.01187	0.06919	0.00559
TMEM165	1.02839	0.98771	0.04069	2.58673	0.00985	0.07153	0.00974
SNRPB2	1.80070	1.75985	0.04085	2.12244	0.00311	0.07860	0.03388
PCMT1	1.10476	1.06387	0.04089	2.69546	0.01115	0.07064	0.00707
UBXN4	0.81775	0.77679	0.04096	2.74218	0.01167	0.07024	0.00614
UXT	1.31621	1.27460	0.04161	2.39920	0.00760	0.07562	0.01649
SNRPB	1.67022	1.62797	0.04225	2.25783	0.00556	0.07894	0.02403
NDUFV1	1.14451	1.10088	0.04363	3.01207	0.01523	0.07204	0.00262
RNF7	1.62057	1.57650	0.04408	2.35324	0.00735	0.08080	0.01867
RNPS1	1.06916	1.02450	0.04466	2.69721	0.01219	0.07713	0.00703
DCTN2	1.52485	1.47740	0.04745	2.36622	0.00813	0.08677	0.01803
COA3	0.86460	0.81707	0.04752	3.78504	0.02291	0.07214	0.00016
NR2F2	0.95066	0.90242	0.04823	3.10537	0.01778	0.07869	0.00192
C14orf119	0.53716	0.48879	0.04837	5.14384	0.02993	0.06681	0.00000
PPIG	1.50163	1.45280	0.04884	3.04094	0.01735	0.08033	0.00238
SERP1	1.16676	1.11767	0.04909	2.64316	0.01268	0.08551	0.00826
PGK1	2.34545	2.29065	0.05480	2.29984	0.00808	0.10152	0.02152
MRPS21	1.75279	1.69662	0.05618	2.97735	0.01918	0.09317	0.00293
RBM8A	1.37981	1.32198	0.05783	3.19338	0.02232	0.09334	0.00142
ZFYVE21	1.67675	1.61528	0.06146	3.24613	0.02434	0.09859	0.00118
LDHB	3.72983	3.66768	0.06215	2.16802	0.00594	0.11835	0.03023
EPS8	1.74574	1.68326	0.06248	2.28474	0.00886	0.11611	0.02240
EIF4H	1.72136	1.65730	0.06406	3.12755	0.02390	0.10422	0.00178
SNX3	3.73046	3.66605	0.06441	2.01535	0.00174	0.12707	0.04396
TBCA	3.05524	2.98878	0.06647	2.39819	0.01212	0.12081	0.01654
TMBIM6	3.50191	3.43349	0.06842	2.23617	0.00843	0.12842	0.02541
ARPC3	2.79980	2.72909	0.07070	2.59523	0.01729	0.12412	0.00950
OAZ2	2.04775	1.97134	0.07641	2.77145	0.02235	0.13047	0.00561
SEC61B	2.67409	2.59618	0.07791	2.47220	0.01612	0.13970	0.01348
APOE	1.88857	1.80186	0.08671	2.32910	0.01371	0.15970	0.01992

COX6C	8.08626	7.96714	0.11913	2.18398	0.01218	0.22608	0.02904
UQCR11	6.49554	6.36583	0.12971	2.51016	0.02839	0.23103	0.01212
DDX5	8.60654	8.42733	0.17921	2.37051	0.03098	0.32744	0.01783
RPS20	18.31942	18.12138	0.19805	2.24399	0.02500	0.37109	0.02491
HNRNPA1	12.37161	12.15761	0.21400	2.36998	0.03695	0.39105	0.01785
NPM1	10.25655	10.03965	0.21690	2.64500	0.05611	0.37769	0.00821
CFL1	13.25354	13.02221	0.23133	3.32224	0.09480	0.36785	0.00090
HSP90AB1	10.13270	9.89253	0.24017	2.68210	0.06459	0.41575	0.00736
YBX1	14.39208	14.13839	0.25369	3.27040	0.10159	0.40579	0.00109
RPL8	30.56312	30.25907	0.30405	2.25422	0.03958	0.56852	0.02425
RPS7	22.92202	22.59555	0.32647	3.01037	0.11383	0.53911	0.00263
RPS3A	36.08535	35.70302	0.38233	2.11483	0.02786	0.73681	0.03453
RPS3	30.53291	30.13703	0.39588	2.47043	0.08167	0.71008	0.01355
ACTG1	24.17112	23.75092	0.42020	2.55391	0.09759	0.74281	0.01070
RPS27A	43.52303	43.02586	0.49717	2.24392	0.06274	0.93160	0.02491
RPS2	32.49222	31.96756	0.52466	1.98194	0.00561	1.04371	0.04758
RPL7	44.32903	43.57727	0.75176	3.23644	0.29632	1.20720	0.00122
EEF1A1	87.91344	86.90372	1.00972	2.81461	0.30632	1.71312	0.00492

Supplemental Table 7. Gene set enrichment analyses for genes upregulated in low CD163 SMCs using the Jensen tissues ontology

Term	P-value	Adjusted P-value	Old P-value	Old Adjusted P-value	Odds Ratio	Combined Score
Bone	5.65E-32	2.09E-29	0	0	14.31731	1030.15362
Eye	4.44E-27	8.22E-25	0	0	10.45516	634.40705
Olfactory bulb	6.69E-27	8.25E-25	0	0	9.51953	573.72968
Intestine	5.16E-25	4.77E-23	0	0	9.10083	508.95374
Thymus	1.24E-24	9.17E-23	0	0	8.67287	477.42298
Spinal ganglion	1.39E-23	8.31E-22	0	0	8.03279	422.76456

Pancreatic islet	1.57E-23	8.31E-22	0	0	8.16381	428.65562
Parenchyma	2.18E-23	1.01E-21	0	0	8.16989	426.31937
Ophthalmic nerve	4.81E-22	1.98E-20	0	0	7.48509	367.40806
Small intestine	4.50E-21	1.66E-19	0	0	7.51853	352.24997
Trigeminal ganglion	4.98E-19	1.68E-17	0	0	6.30795	265.83704
Prefrontal cortex	1.71E-18	5.29E-17	0	0	6.22283	254.56133
Caudate nucleus	2.19E-18	6.23E-17	0	0	6.07800	247.15320
Thalamus	3.14E-18	8.30E-17	0	0	6.02938	243.00004
Adrenal cortex	4.52E-18	1.11E-16	0	0	5.97041	238.45205
Pons	5.68E-17	1.31E-15	0	0	5.62172	210.29453
Bronchial epithelial cell	8.55E-17	1.86E-15	0	0	5.54509	205.15818
Pineal gland	1.26E-16	2.58E-15	0	0	5.57548	204.13996
Gut	6.87E-16	1.34E-14	0	0	5.84944	204.22540
Superior cervical ganglion	8.87E-16	1.64E-14	0	0	5.22234	180.99725
Amygdala	1.53E-15	2.69E-14	0	0	5.14945	175.67647
Vascular system	1.86E-15	3.12E-14	0	0	8.66831	294.02650
Medulla oblongata	3.01E-15	4.85E-14	0	0	5.06992	169.51896
Adipose tissue	3.43E-15	5.29E-14	0	0	12.17175	405.39117
Muscle	3.87E-15	5.73E-14	0	0	6.25300	207.50344
Spinal cord	9.38E-14	1.33E-12	0	0	7.59904	227.95272
Monocyte	1.40E-13	1.92E-12	0	0	4.82575	142.83167
Cingulate cortex	2.74E-13	3.62E-12	0	0	4.47381	129.41168
Globus pallidus	3.19E-13	4.08E-12	0	0	4.58968	132.05567
Cerebral peduncle	5.16E-13	6.37E-12	0	0	4.39123	124.23561
Retina	5.37E-13	6.41E-12	0	0	6.46819	182.74288
Embryonic brain	6.75E-13	7.80E-12	0	0	4.35671	122.09571
Subthalamic nucleus	1.38E-12	1.55E-11	0	0	4.31656	117.87460
Hypothalamus	5.69E-12	6.19E-11	0	0	8.51631	220.51356
Thyroid gland	6.76E-12	7.15E-11	0	0	11.11382	285.84618
Heart	2.22E-11	2.28E-10	0	0	13.10142	321.41433
Blood	7.30E-11	7.30E-10	0	0	3.76839	87.95457

Uterus	1.01E-10	9.85E-10	0	0	6.47783	149.08420
Parietal lobe	1.22E-10	1.16E-09	0	0	4.30423	98.23880
Brain	2.63E-10	2.38E-09	0	0	6.75490	149.00804
Occipital lobe	2.64E-10	2.38E-09	0	0	4.19827	92.59017
Ascites	2.75E-10	2.43E-09	0	0	6.31057	138.91372
Nerve	3.48E-10	2.99E-09	0	0	20.59197	448.47956
Erythroid cell	6.24E-10	5.25E-09	0	0	3.59695	76.23832
Dendritic cell	9.93E-10	8.17E-09	0	0	3.46322	71.79336
Cytotoxic T-lymphocyte	1.55E-09	1.25E-08	0	0	3.42993	69.56669
Bladder	4.71E-09	3.71E-08	0	0	8.12458	155.77482
Frontal lobe	7.49E-09	5.78E-08	0	0	4.08503	76.42837
Helper T-lymphocyte	8.02E-09	6.06E-08	0	0	3.24296	60.45245

Supplemental Table 8. Gene set enrichment analyses for genes upregulated in low CD163 SMCs using the Mouse gene atlas ontology

Term	P-value	Adjusted P-value	Old P-value	Old Adjusted P-value	Odds Ratio	Combined Score
Osteoblast day14	3.1E-09	1.8E-07	0	0	10.29429	201.85093
Osteoblast day21	7.8E-08	2.3E-06	0	0	9.70751	158.90136
Uterus	7.2E-05	1.2E-03	0	0	9.44612	90.07944
Umbilical cord	7.9E-05	1.2E-03	0	0	7.45728	70.45591
Lung	3.8E-04	4.4E-03	0	0	4.96856	39.18126
Osteoblast day5	1.8E-03	1.8E-02	0	0	6.17365	39.06785
Macrophage peri LPS thio 0hrs	8.2E-03	6.9E-02	0	0	3.63559	17.45591
3T3-L1	1.3E-02	9.3E-02	0	0	4.69200	20.54354
Nih 3T3	3.7E-02	2.4E-01	0	0	3.33667	11.03718
C3H 10T1 2	4.2E-02	2.5E-01	0	0	4.06024	12.89492
MEF	6.2E-02	3.3E-01	0	0	2.78876	7.75941
Bladder	7.3E-02	3.6E-01	0	0	3.20785	8.41083

Kidney	1.4E-01	5.8E-01	0	0	1.87498	3.68296
Iris	1.4E-01	5.8E-01	0	0	3.11167	6.10069
Placenta	1.5E-01	5.8E-01	0	0	2.01671	3.86889
Macrophage peri LPS thio 1hrs	1.7E-01	6.0E-01	0	0	1.73191	3.01880
Hypothalamus	1.8E-01	6.0E-01	0	0	2.09130	3.57306
Bone	1.8E-01	6.0E-01	0	0	2.62667	4.46805
Intestine small	2.0E-01	6.1E-01	0	0	1.77161	2.85161
Ciliary bodies	2.1E-01	6.1E-01	0	0	1.94934	3.06539
Eyecup	2.3E-01	6.4E-01	0	0	1.49745	2.22057
Adipose white	2.6E-01	6.4E-01	0	0	2.06227	2.78848
Macrophage peri LPS thio 7hrs	2.7E-01	6.4E-01	0	0	1.45473	1.89135
Skeletal muscle	2.8E-01	6.4E-01	0	0	1.44832	1.86793
Dorsal root ganglia	2.8E-01	6.4E-01	0	0	1.51830	1.93347
Intestine large	2.8E-01	6.4E-01	0	0	1.64505	2.07860
Heart	3.1E-01	6.8E-01	0	0	1.44360	1.69120
Adrenal gland	3.4E-01	7.2E-01	0	0	1.66798	1.78660
Pancreas	4.2E-01	8.3E-01	0	0	1.22175	1.05611

Supplemental Table 9. Gene set enrichment analyses for genes upregulated in low CD163 SMCs using the PanglaoDB ontology

Term	P-value	Adjusted P-value	Old P-value	Old Adjusted P-value	Odds Ratio	Combined Score
Fibroblasts	1.62E-19	1.61E-17	0	0	23.51206	1017.24868
Chondrocytes	1.89E-17	9.36E-16	0	0	29.31752	1128.91531
Stromal Cells	4.47E-17	1.48E-15	0	0	32.42464	1220.64104
Myofibroblasts	2.37E-15	5.86E-14	0	0	34.42689	1159.39644
Pancreatic Stellate Cells	9.57E-15	1.89E-13	0	0	30.54568	986.02848
Osteoblasts	3.06E-14	5.05E-13	0	0	23.24824	723.43715

Hepatic Stellate Cells	9.43E-14	1.33E-12	0	0	25.12859	753.65632
Pulmonary Vascular Smooth Muscle Cells	2.17E-12	2.69E-11	0	0	28.14347	755.77477
Pericytes	1.58E-11	1.74E-10	0	0	19.05763	473.98147
Peritubular Myoid Cells	1.84E-11	1.82E-10	0	0	22.69725	561.05979
Airway Smooth Muscle Cells	6.01E-11	5.41E-10	0	0	25.01199	588.67455
Juxtaglomerular Cells	8.95E-11	7.38E-10	0	0	23.93138	553.69991
Mesangial Cells	1.81E-10	1.38E-09	0	0	18.03285	404.55220
Smooth Muscle Cells	7.50E-10	5.30E-09	0	0	15.61946	328.18667
Adipocytes	9.08E-08	5.99E-07	0	0	11.06798	179.45934
Endothelial Cells (Aorta)	3.45E-07	2.14E-06	0	0	11.20739	166.75613
Endothelial Cells	3.94E-07	2.30E-06	0	0	9.36250	138.06000
Leydig Cells	6.17E-07	3.39E-06	0	0	12.68244	181.33727
Vascular Smooth Muscle Cells	7.90E-07	4.11E-06	0	0	15.69690	220.57017
Mesothelial Cells	7.42E-06	3.67E-05	0	0	10.97652	129.64310
Loop of Henle Cells	8.50E-06	3.82E-05	0	0	10.73967	125.39226
Müller Cells	8.50E-06	3.82E-05	0	0	10.73967	125.39226
Adipocyte Progenitor Cells	2.30E-05	9.89E-05	0	0	11.71471	125.11987
Melanocytes	5.21E-05	2.15E-04	0	0	10.04521	99.05991
Mast Cells	1.68E-04	6.66E-04	0	0	6.56517	57.05191
Meningeal Cells	2.68E-04	9.82E-04	0	0	9.57012	78.71369
Myoepithelial Cells	2.68E-04	9.82E-04	0	0	9.57012	78.71369
Myoblasts	4.08E-04	0.001444065	0	0	8.69527	67.85100
Satellite Cells	6.39E-04	0.002182342	0	0	7.84654	57.71271

References

1. Otsuka F, et al. Community-based statins and advanced carotid plaque: Role of CD163 positive macrophages in lipoprotein-associated phospholipase A(2) activity in atherosclerotic plaque. *Atherosclerosis.* 2017;267:78-89.
2. Golubeva YG, et al. Optimizing Frozen Sample Preparation for Laser Microdissection: Assessment of CryoJane Tape-Transfer System®. *PLoS One.* 2013;8(6):e66854.
3. Virmani R, et al. Lessons from sudden coronary death: a comprehensive morphological classification scheme for atherosclerotic lesions. *Arterioscler Thromb Vasc Biol.* 2000;20(5):1262-1275.
4. Finn AV, et al. Hemoglobin directs macrophage differentiation and prevents foam cell formation in human atherosclerotic plaques. *J Am Coll Cardiol.* 2012;59(2):166-177.
5. Akahori H, et al. CD163 interacts with TWEAK to regulate tissue regeneration after ischaemic injury. *Nat Commun.* 2015;6:7792.
6. Tziakas DN, et al. Lysed Erythrocyte Membranes Promote Vascular Calcification. *Circulation.* 2019;139(17):2032-2048.
7. Saeed O, et al. Pharmacological suppression of hepcidin increases macrophage cholesterol efflux and reduces foam cell formation and atherosclerosis. *Arterioscler Thromb Vasc Biol.* 2012;32(2):299-307.
8. Villa-Bellosta R, et al. Alternatively activated macrophages exhibit an anticalcifying activity dependent on extracellular ATP/pyrophosphate metabolism. *Am J Physiol Cell Physiol.* 2016;310(10):C788-799.

9. Guo L, et al. CD163+ macrophages promote angiogenesis and vascular permeability accompanied by inflammation in atherosclerosis. *J Clin Invest.* 2018;128(3):1106-1124.

10. Wirka RC, et al. Atheroprotective roles of smooth muscle cell phenotypic modulation and the TCF21 disease gene as revealed by single-cell analysis. *Nat Med.* 2019;25(8):1280-1289.

11. Stuart T, et al. Comprehensive Integration of Single-Cell Data. *Cell.* 2019;177(7):1888-1902.e1821.

12. Turner AW, et al. Single-nucleus chromatin accessibility profiling highlights regulatory mechanisms of coronary artery disease risk. *Nat Genet.* 2022;54(6):804-816.

13. Granja JM, et al. ArchR is a scalable software package for integrative single-cell chromatin accessibility analysis. *Nat Genet.* 2021;53(3):403-411.

14. Chen EY, et al. Enrichr: interactive and collaborative HTML5 gene list enrichment analysis tool. *BMC Bioinformatics.* 2013;14:128.

15. Keenan AB, et al. ChEA3: transcription factor enrichment analysis by orthogonal omics integration. *Nucleic Acids Res.* 2019;47(W1):W212-w224.

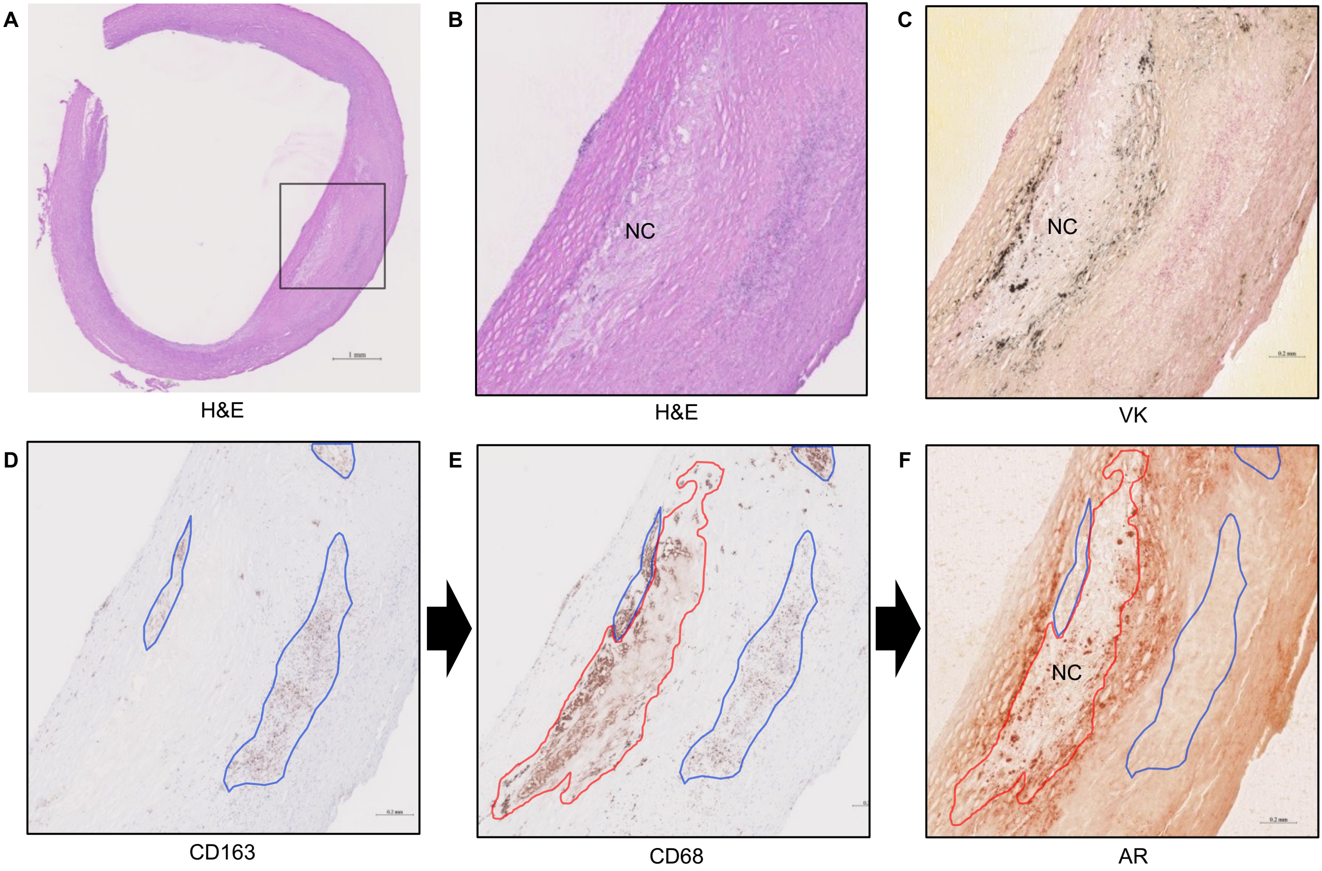

Supplemental Figure 1. Outline of analysis for the amount of calcification in the spatial area of CD163$^+$/CD68$^+$ and CD163$^-$/CD68$^+$ in human atheroma of carotid artery (frozen CEA specimens)

Surgically removed carotid artery pathologic sections from 62-yo male patient. **A**: Low-power H&E image of fibroatheroma. **B-F**: High-power image of H&E (**B**), VK (**C**), CD163 (**D**) and CD68 (**E**) immunostaining, and AR (**F**) images of the corresponding black rectangular field in Image **A**. To determine the spatial distribution of CD163, CD68, and calcification, CD163 positive areas were digitally traced (bordered by blue line [**D**]) in CD163 IHC images (1st step). The CD163 positive area was digitally overlaid on an adjacent CD68 IHC image (2nd step). Subsequently, CD68 positive but CD163 negative area was digitally traced (bordered by red line [**E**]) in CD68 IHC image (3rd step). Both blue border (CD163$^+$/CD68$^+$) and red (CD163$^-$/CD68$^+$) border areas were digitally overlaid on an adjacent AR image (4th step). Finally, AR positive areas in blue and red zones were determined by HALO digital software (5th step). Total 70 advanced atheroma sections obtained from 32 patients were applied for this analysis. AR=Alizarin Red, CEA=carotid endarterectomy, IHC=immunohistochemistry, NC=necrotic core, VK=Von Kossa.

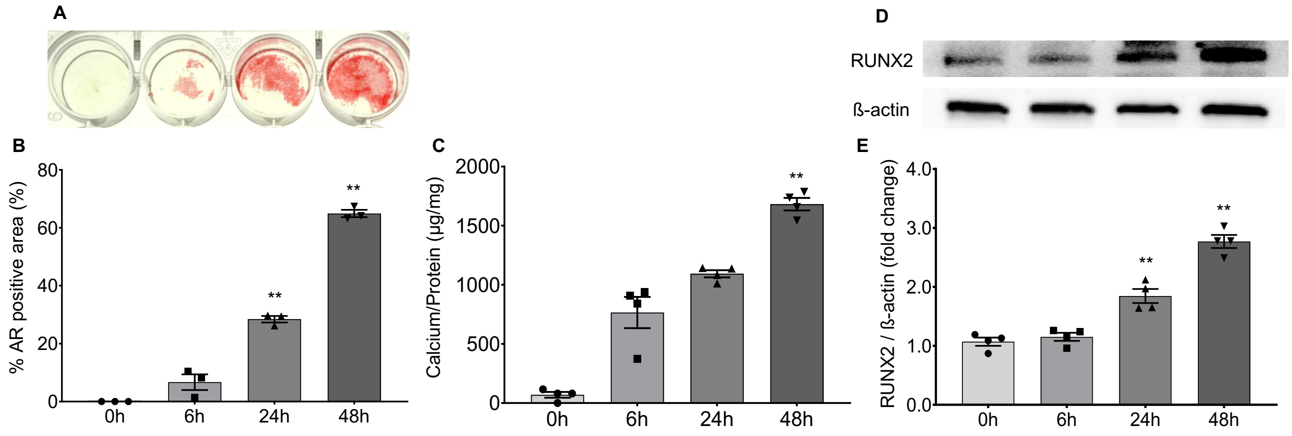

Supplemental Figure 2. Time dependent effect of OS media on HASMC calcification in vitro

A-**B**: HASMC were seeded in 24-well culture plates, grown to confluence, and cultivated for 6, 24, and 48 hours. Representative image of AR staining of HASMC (**A**) and summary of % AR positive areas for each time point after exposure to culture media with OS is shown (**B**) (n=3 in each).

C: Summary of calcium amount in samples of each time point examined by colorimetric assay adjusted for protein content (n=4 in each).

D-**E**: Representative western blot images of RUNX2 and β-actin from protein samples extracted at each time point (**D**). Summary of densitometry analysis is shown in **E** (n=4 in each).

** p<0.01 vs 0h.

Results are presented as the mean±standard error and ANOVA followed by post-hoc Tukey's test was conducted for statistical analysis (**B**-**C**, **E**). Data normality was tested by Shapiro-Wilk test. All experiments were performed at least three times to confirm the reproducibility.

AR=Alizarin Red, HASMC=human aortic smooth muscle cell, OS=osteogenic components supplementation, RUNX2=runt-related transcription factor 2.

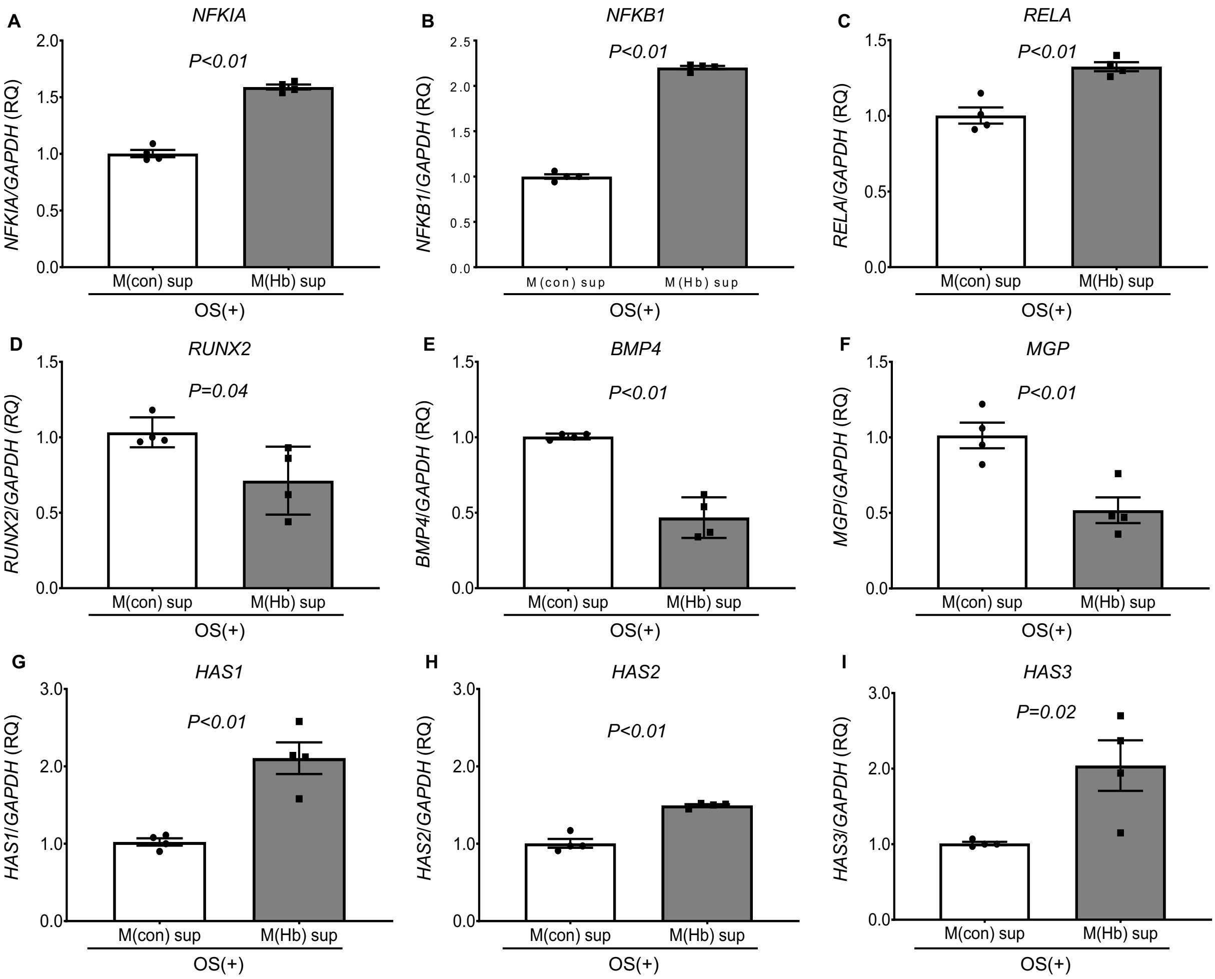

Supplemental Figure 3. Validation results of RT-PCR in RNA samples extracted from M(con) and M(Hb) plus OS exposed HASMC

HASMCs were treated with M(con) or M(Hb) + OS supernatant for 24 h. Real-time RT–PCR analysis was performed in order to validate the result of microarray analysis (Figure 4) including the expression levels of *NFKIA, NFKB1, RELA, RUNX2, BMP4, MGP, HAS1, HAS2, HAS3*. Data were normalized with *GAPDH* expression. Results are presented as the mean±standard error and t-test was conducted for statistical analysis (n=3 per group). Data normality was tested by Shapiro-Wilk test. All experiments were performed at least three times to confirm the reproducibility.

M(con)sup=control macrophage supernatant, M(Hb)sup=HH-differentiated macrophage supernatant, OS=osteogenic components supplementation, RQ=relative quantification.

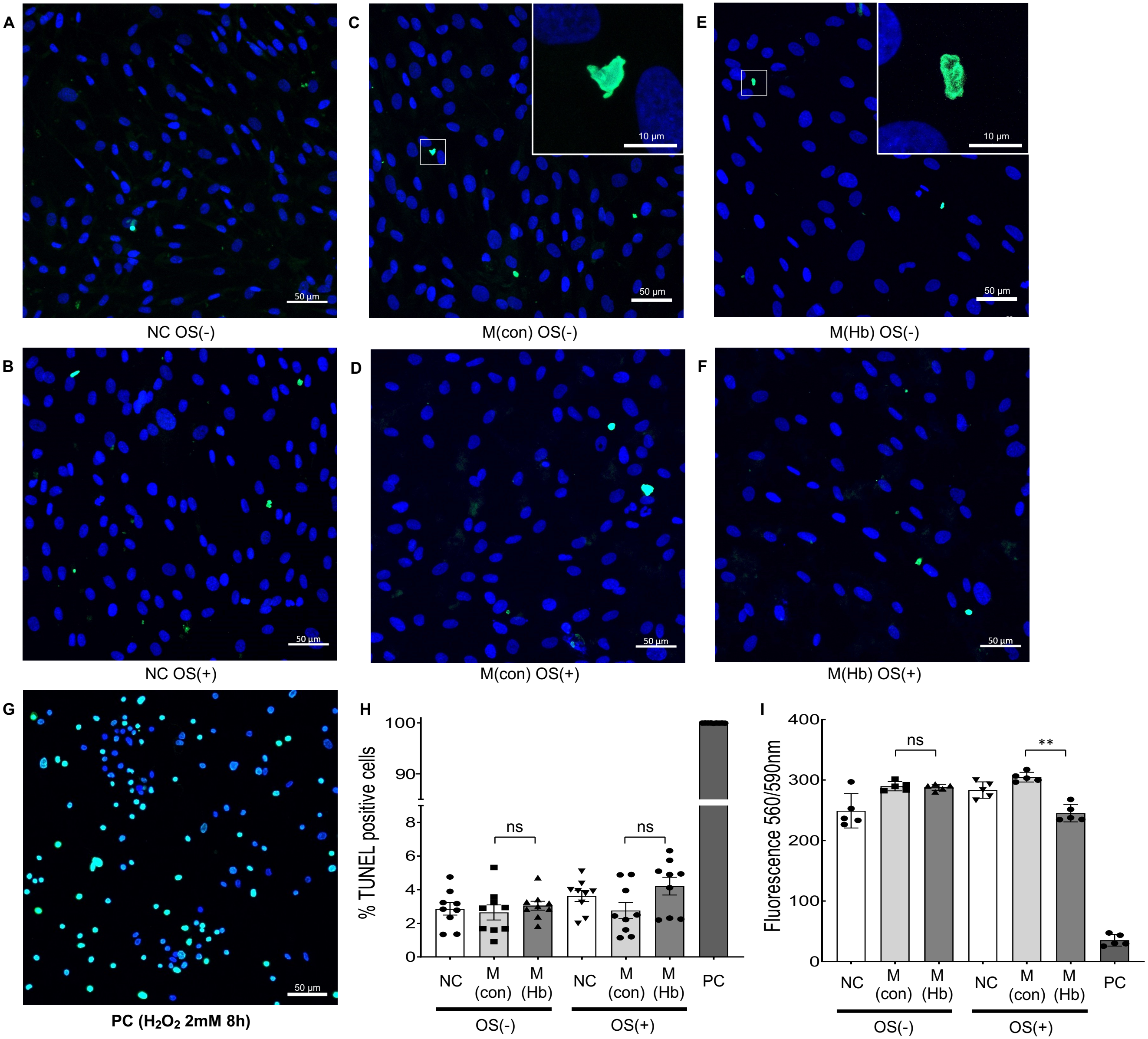

Supplemental Figure 4. The effect of M(con) and M(Hb) supernatant on the cell apoptosis and viability of HASMC

A-**G**: Representative fluorescent microscopic images of terminal deoxynucleotidyl transferase-mediated dUTP nickend-labeling (TUNEL) staining of HASMC cultured with normal growth media (negative control; NC) with or without OS (**A**; OS(-), **B**; OS(+)), M(con) with or without OS (**C**; OS(-), **D**; OS(+)), or M(Hb) supernatant with or without OS (**E**; OS(-), **F**; OS(+)) for 48 hours. For positive control (PC), HASMCs were exposed to culture media containing 2mM of H_2O_2 for 8 hours (**G**). DNA fragmentation in apoptotic cells were detected by fluorescein-12-dUTP (green). Nucleus were counterstained by DAPI (blue). Small inset on upper-right in **C** and **E** represent higher magnification of TUNEL positive cells in the white rectangular field of each group. Scale bars indicate 50μm in low power or 10μm in high power fields. **H**: Summarized data of TUNEL positive cells. Bars indicate % of TUNEL positive cells in each condition. The values represent the mean ± standard error from 3 independent experiments, which include 3 different fields involving >100 cells in each (overall n=9 per group). **I**: Summarized data from PrestoBlue HS cell viability assay (n=5 per group) in the same experimental condition as TUNEL assay.

The experiment was performed three times to confirm the reproducibility. The values represent the mean ± standard error. ANOVA followed by post-hoc Tukey's test was applied. Data normality was tested by Shapiro-Wilk test.

NC=negative control, PC=positive control, TUNEL=terminal deoxynucleotidyl transferase-mediated dUTP nick end-labeling, Others are as Supplemental Figure 3.

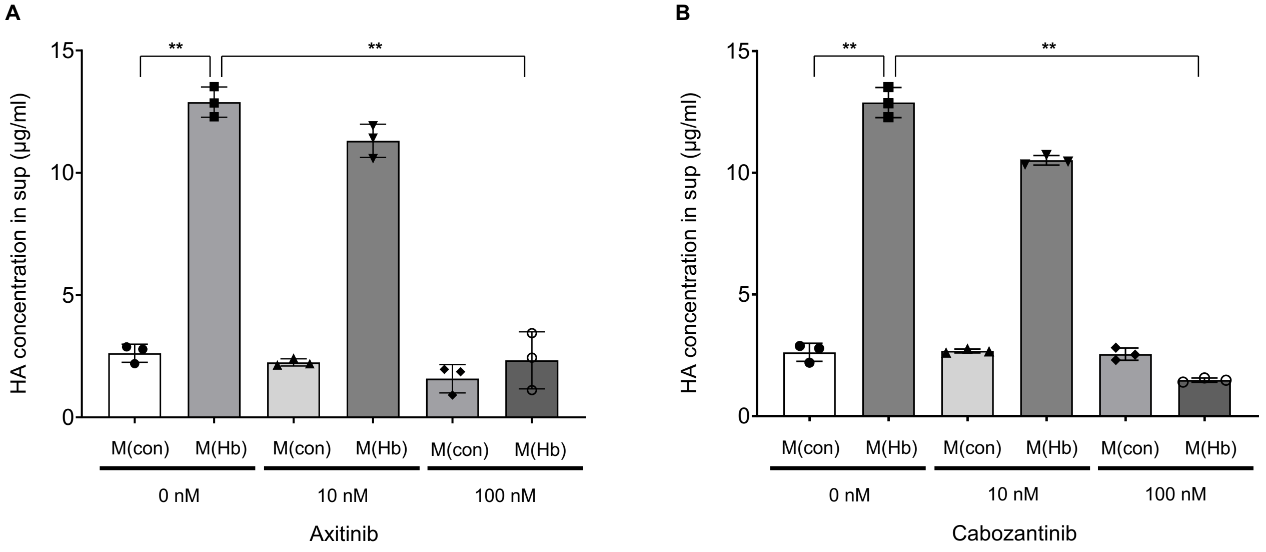

Supplemental Figure 5. Augmented hyaluronan synthesis in HASMC by M(Hb) supernatants exposure is attenuated by VEGF receptor antagonists
A-**B**: Summary of HA concentration in M(con) and M(Hb) supernatant post-exposing (24 hours) to HASMC (ELISA, n=3 per group) with VEGF receptor antagonist, Axitinib (**A**) or Cabozantinib (**B**), in different dose setting (0, 10, and 100nM). **P<0.01. Results are presented as the mean±standard error. ANOVA followed by post-hoc Tukey's test was applied. Data normality was tested by Shapiro-Wilk test. All experiments were performed at least three times to confirm the reproducibility. HA=hyaluronan, M(con)=control macrophage supernatant, M(Hb)=hemoglobin-haptoglobin complex-differentiated macrophage supernatant.

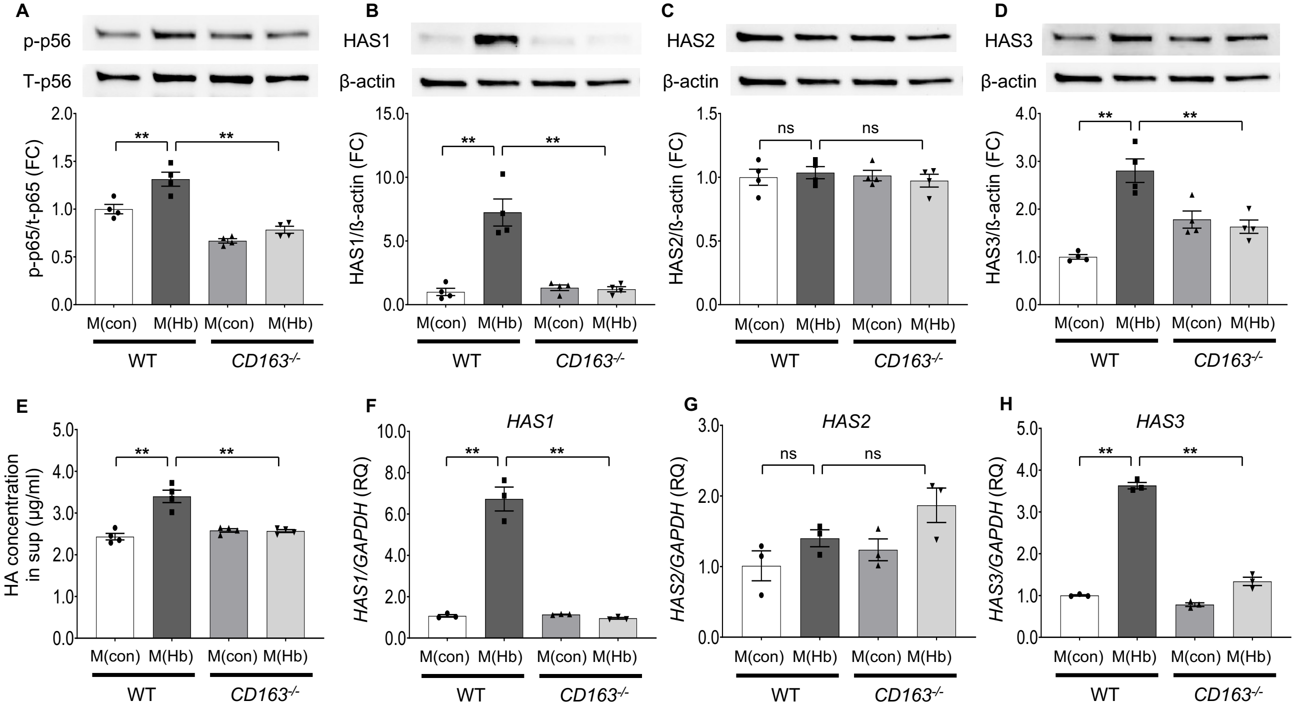

Supplemental Figure 6. Effect of peritoneal macrophage supernatant obtained from WT or *CD163*$^{-/-}$ mice on NFκB signaling and HA synthesis

Peritoneal macrophages are collected from WT or *CD163*$^{-/-}$ mice and cultured with or without mouse hemoglobin for 24 hours. The supernatant was exposed to HASMC for 24 hours. **A**-**D**: Immunoblotting of HASMC with quantitation of densitometry for phospho-p65 (Ser 536)/total-p65 (**A**), HAS1/β-actin (**B**), HAS2/β-actin (**C**), and HAS3/β-actin (**D**) in HASMC exposed to mouse peritoneal macrophage supernatant for 24 hours (1: WT M(con), 2: WT M(Hb), 3: *CD163*$^{-/-}$ M(con), and 4: *CD163*$^{-/-}$ M(Hb)) (n=4 per group). **E**: Summary of HA concentration in WT M(con), WT M(Hb), *CD163*$^{-/-}$ M(con), and *CD163*$^{-/-}$ M(Hb) supernatant exposed (24 hours) to HASMC (ELISA, n=4 per group). **F**-**H**: Result of real-time PCR analysis of HASMC which exposed to peritoneal macrophage supernatant for 24 hours, including expression levels of *HAS1* (**F**), *HAS2* (**G**), and *HAS3* (**H**). Data were normalized with *GAPDH* expression and presented as mean ± standard error (n=3 per group). Results are presented as the mean ± standard error and ANOVA followed by post-hoc Tukey's test was applied for statistical analysis. Data normality was tested by Shapiro-Wilk test. All experiments were performed at least three times to confirm the reproducibility.

CD163$^{-/-}$ M(con)=*CD163*$^{-/-}$ macrophage control supernatant, *CD163*$^{-/-}$ M(Hb)=Hb-stimulated *CD163*$^{-/-}$ macrophage supernatant, FC=fold changes, RQ=relative quantification, WT=wild type, WT M(con)=WT macrophage control supernatant, WT M(Hb)=Hb-stimulated WT macrophage supernatant

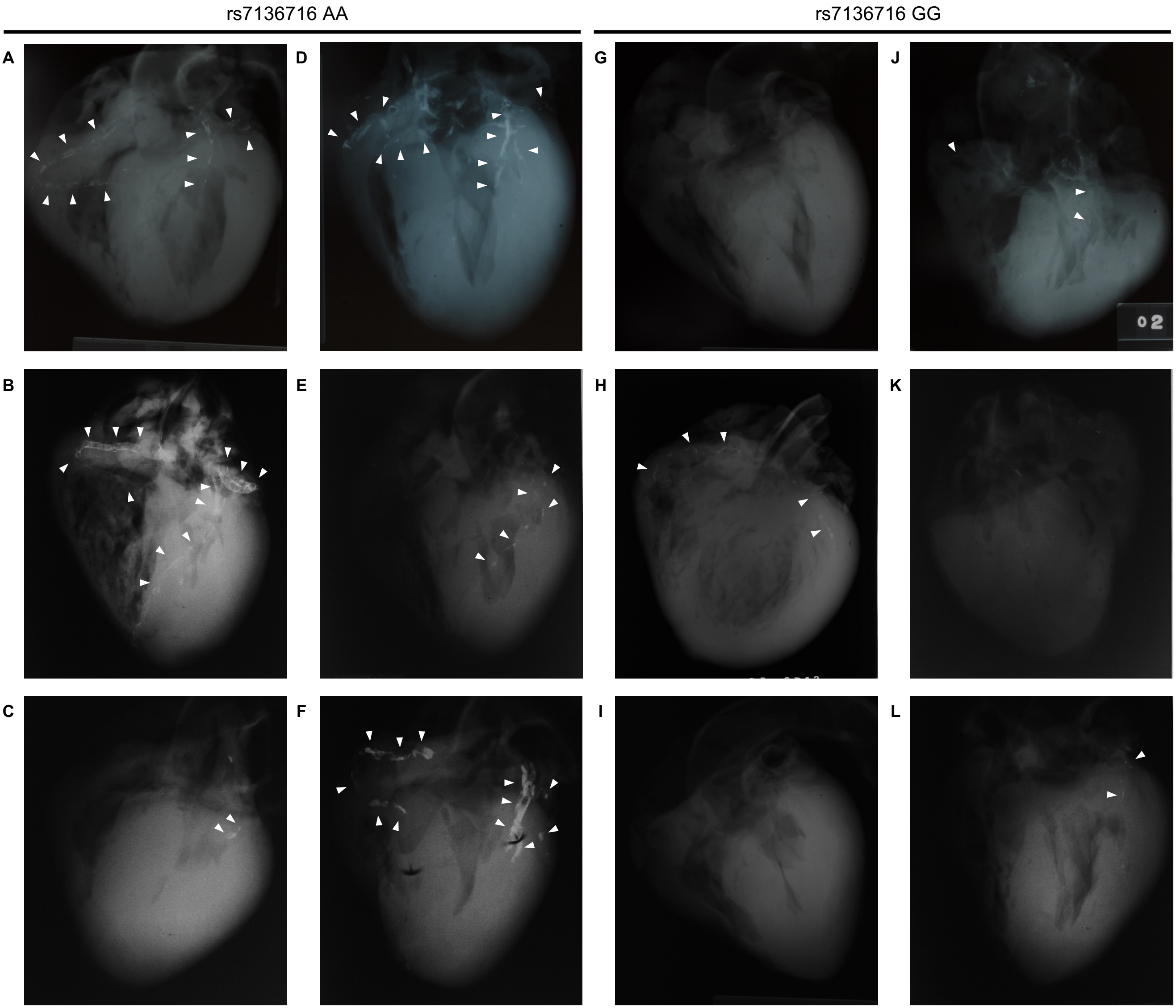

Supplemental Figure 7. Representative radiographs of autopsy hearts of sudden cardiac death cases of African American victims with major AA or minor GG genotype of rs7136716 SNPs

Radiographs of **A**-**F** are from carriers of major AA genotype and **G**-**L** are from carriers of minor GG genotype of rs7136716 SNPs. **A**: A heart with severe CAD from 41-year-old male without any cardiac risk factors. **B**: A heart with severe CAD from 61-year-old female with history of hypertension, diabetes, and CKD. **C**: A heart with severe CAD from 50-year-old female with history of hypertension. **D**: A heart with severe CAD from 78-year-old male with history of abdominal aortic aneurysm. **E**: A heart with severe CAD from 41-year-old male with history of OMI, hypertension, and diabetes. **F**: A heart with severe CAD from 47-year-old male with history of OMI. **G**: A heart with severe CAD from 44-year-old male with history of drug use without other cardiac risk factors. **H**: A heart with severe CAD from 62-year-old male with history of hypertension and valvular heart disease. **I**: A heart with severe CAD from 48-year-old male without known cardiac risk factors. **J**: A heart with severe CAD from 74-year-old male with history of diabetes. **K**: A heart with recent MI from 47-year-old female with history of hypertension and smoking. **L**: A heart with severe CAD from 46-year-old male with history of OMI. White arrowheads indicate visible coronary calcification on X-ray. CAD=coronary artery disease, CKD=chronic kidney disease, OMI=old myocardial infarction.

Conflict of interest: RV and AVF have received institutional research support from R01 HL141425, Leducq Foundation Grant, Amazon Web Services (AWS) COVID-19 Diagnostic Development Initiative Grant, 480 Biomedical, 4C Medical, 4Tech, Abbott Vascular, Ablative Solutions, Absorption Systems, Advanced NanoTherapies, Aerwave Medical, Alivas, Amgen, Asahi Medical, Aurios Medical, Avantec Vascular, BD, Biosensors, Biotronik, Biotyx Medical, Bolt Medical, Boston Scientific, Canon, Cardiac Implants, Cardiawave, CardioMech, Cardionomic, Celonova, Cerus Endovascular, Chansu Vascular Technologies, Children's National Hospital, Concept Medical, Cook Medical, Cooper Health, Cormaze, CRL, Croivalve, CSI, Dexcom, Edwards Lifesciences, Elucid Bioimaging, eLum Technologies, Emboline, Endotronix, Envision, Filterlex, Imperative Care, Innovalve, Innovative Cardiovascular Solutions, Intact Vascular, Interface Biologics, Intershunt Technologies, Invatin, Lahav, LimFlow, L&J Bio, Lutonix, Lyra Therapeutics, Mayo Clinic, Maywell, MD Start, MedAlliance, Medanex, Medtronic, Mercator, Microport, Microvention, Neovasc, Nephronyx, Nova Vascular, Nyra Medical, Occultech, Olympus, Ohio Health, OrbusNeich, Ossiso, Phenox, Pi-Cardia, Polares Medical, Polyvascular, Profusa, ProKidney, Protembis, Pulse Biosciences, Qool Therapeutics, Recombinetics, ReCor Medical, Regencor, Renata Medical, Restore Medical, Ripple Therapeutics, Rush University, Sanofi, Shockwave, SMT, SoundPipe, Spartan Micro, Spectrawave, Surmodics, Terumo, Jacobs Institute, Transmural Systems, Transverse Medical, TruLeaf, UCSF, University of Pittsburgh Medical Center, Vascudyne, Vesper, Vetex Medical, Whiteswell, W. L. Gore & Associates, and Xeltis. AVF has received honoraria from Abbott Vascular, Biosensors, Boston Scientific, Celonova, Cook Medical, CSI, Lutonix Bard, Sinomed, and Terumo and is a consultant to Amgen, Abbott Vascular, Boston Scientific, Celonova, Cook Medical, Lutonix Bard, and Sinomed. RV has received honoraria from Abbott Vascular, Biosensors, Boston Scientific, Celonova, Cook Medical, Cordis, CSI, Lutonix Bard, Medtronic, OrbusNeich, Sinomed, etics (Philips), ReCor Medical, Terumo, and W. L. Gore & Associates and is a consultant for BD, Celonova, Cook Medical, CSI, Edwards Lifesciences, Medtronic, OrbusNeich, ReCor Medical, Sinomed, Surmodics, Terumo, W. L. Gore & Associates, and Xeltis. LG is supported by R01 HL141425 and Leducq Foundation Grant. AS, MM, and KK are supported by Leducq Foundation Grant.
